# Environmental risk factors, protective factors, and biomarkers for amyotrophic lateral sclerosis: an umbrella review

**DOI:** 10.3389/fnagi.2025.1541779

**Published:** 2025-06-13

**Authors:** Qian Wu, Junyi Yang, Yuanjie Duan, Yumei Ma, Yue Zhang, Shutong Tan, Jinke Wang, Yaxin Wang, Binhui Liu, Jian Zhang, Xu Liu

**Affiliations:** ^1^Department of Neurology, First Affiliated Hospital of China Medical University, Shenyang, Liaoning, China; ^2^Key Laboratory of Cell Biology, Department of Cell Biology, National Health Commission of the People’s Republic of China, China Medical University, Shenyang, Liaoning, China; ^3^Key Laboratory of Medical Cell Biology, Ministry of Education of the People’s Republic of China, Shenyang, China; ^4^Key Laboratory of Neurological Disease Big Data of Liaoning Province, Shenyang, China; ^5^Shenyang Clinical Medical Research Center for Difficult and Serious Diseases of the Nervous System, Shenyang, China

**Keywords:** amyotrophic lateral sclerosis, environmental risk factors, biomarkers, meta-analysis, umbrella review

## Abstract

**Introduction:**

Amyotrophic lateral sclerosis (ALS) is a fatal neurodegenerative disease characterized by the rapid loss of motor neurons. Given the significant global economic impact of ALS, effective preventive measures are urgently needed to reduce the incidence of this devastating disease. Recent meta-analyses have explored potential links between environmental factors, biomarkers, and ALS occurrence. However, the findings of these studies have been inconsistent and controversial. Therefore, we present a comprehensive umbrella review of recent meta-analyses to systematically summarize the available epidemiological evidence and evaluate its credibility.

**Methods:**

A systematic search was conducted in PubMed and Embase from inception until 01 October 2024, to identify meta-analyses of observational studies examining associations between environmental risk factors, protective factors, biomarkers, and ALS susceptibility. For each meta-analysis, summary effect estimates, 95% confidence intervals (CIs), 95% prediction intervals, study heterogeneity, small study effects, and excess significance biases were calculated independently by two investigators. The methodological quality was evaluated using the AMSTAR 2 criteria. The strength of the epidemiological evidence was categorized into five levels based on predefined criteria.

**Results:**

Out of 1,902 articles identified, 43 met the inclusion criteria, resulting in 103 included meta-analyses. These analyses covered 46 environmental risk and protective factors (344,597 cases, 71,415,574 population) and 57 cerebrospinal fluid (CSF) and serum biomarkers (30,941 cases, 2,180,797 population). The evidence was classified as convincing (Class I) for the regular use of antihypertensive drugs (OR: 0.85, 95% CI: 0.81–0.88) and highly suggestive (Class II) for premorbid body mass index (OR: 0.97, 95% CI: 0.95 to 0.98), trauma (OR: 1.51, 95% CI: 1.32 to 1.73), CSF NFL levels (SMD: 2.06, 95% CI: 1.61 to 2.51), serum NFL levels (SMD: 1.57, 95% CI: 1.29 to 1.85), ferritin levels (SMD: 0.66, 95% CI: 0.50 to 0.83), and uric acid levels (SMD: −0.72; 95% CI: −0.98 to −0.46).

**Discussion:**

This umbrella review offers new insights into the epidemiological evidence regarding the associations between environmental factors, biomarkers, and ALS susceptibility. We aim for our study to enhance the understanding of the roles of environmental factors and biomarkers in ALS occurrence and assist clinicians in developing evidence-based prevention and control strategies.

## 1 Introduction

Amyotrophic lateral sclerosis (ALS) is a deadly neurodegenerative disease resulting from the deterioration of motor neurons in the brain and spinal cord. It is typically characterized by progressive muscle weakness and atrophy in adults (Feldman et al., 2022). Globally, the incidence and prevalence rates of ALS were estimated to be 1.59 and 4.42 per 100,000, respectively (Xu et al., 2020). Recent studies have indicated that the national cost associated with ALS ranges from $149 million to $1,329 million, imposing a significant financial burden on families and society (Achtert and Kerkemeyer, 2021). Moreover, most ALS patients lack effective treatment, and the median survival time from clinical symptoms to death is 20–48 months (Paulukonis et al., 2015). Therefore, it is crucial to investigate and develop more efficient strategies for the early detection and prevention of this devastating disease.

To date, the causes of ALS have been suggested to be multifactorial, with various genetic predispositions and environmental factors intricately linked to its development. While significant progress has been achieved in investigating the genetic factors associated with ALS (Rosen et al., 1993; Savage et al., 2019), the connection between environmental factors and biomarkers of ALS susceptibility remains unclear. Consequently, numerous systematic reviews and meta-analyses of observational studies have examined potential associations between diverse environmental factors, biomarkers, and ALS incidence. However, the consistency and conclusiveness of the epidemiological evidence from these meta-analyses have not always been consistent and conclusive.

Umbrella reviews are increasingly popular for summarizing and evaluating evidence from published meta-analyses. They play a crucial role in investigating relationships between environmental factors, biomarkers, and diseases such as inflammatory bowel diseases, autism spectrum disorder, and multiple sclerosis (Belbasis et al., 2015; Kim et al., 2019; Piovani et al., 2019). In Belbasis et al. (2016)conducted an umbrella review on ALS susceptibility, consolidating eight meta-analyses to explore potential associations between environmental factors and ALS. Numerous meta-analyses on ALS have been published, prompting us to conduct a comprehensive umbrella review of all recently published meta-analyses. This review aims to systematically summarize the epidemiological evidence available and assess its credibility. We anticipate that our umbrella review will assist healthcare professionals and policymakers in devising effective strategies for the early diagnosis and prevention of ALS.

## 2 Materials and methods

### 2.1 Search strategy and eligibility criteria

The systematic literature search for our umbrella review adhered to the Preferred Reporting Items for Systematic Reviews and Meta-Analyses (PRISMA) guidelines (Moher et al., 2009). We systematically searched the PubMed and Embase databases from their inception to 01 October 2024, using the following search strategy: (“amyotrophic lateral sclerosis” OR “motor neuron disease”) AND (“systematic review” OR “meta-analysis”). Besides the database searches, we conducted manual screening of the reference lists of the retrieved articles to guarantee comprehensive coverage of relevant studies.

### 2.2 Inclusion and exclusion criteria

Our umbrella review comprised meta-analyses examining the relationships between environmental risk factors, protective factors, and biomarkers of susceptibility to ALS. We considered meta-analyses from observational studies (cohort, case-control, and cross-sectional) focusing on high versus low exposure or dose-response relationships. Eligible meta-analyses are needed to provide sufficient data for calculating summary effect estimates, 95% confidence intervals (CIs), and 95% prediction intervals (PIs), as well as information on heterogeneity, small-study effects, and excess significance bias for further analysis. Only studies published in English were included, while meta-analyses based on randomized controlled trials were excluded. Our study specifically excluded research on prognostic factors and biomarkers related to ALS survival, focusing instead on factors associated with ALS occurrence. Studies lacking raw data for calculating summary risk estimates, 95% CIs, or 95% PIs, such as systematic reviews without meta-analyses, were excluded. Publications categorized as reviews, editorials, letters, or conference abstracts were disregarded due to the absence of original data, and duplicate publications were likewise excluded.

### 2.3 Data extraction and methodological quality assessment

Two researchers independently extracted the following information for each eligible meta-analysis: name of the first author; journal and publication year; original article retrieval time; environmental risk factors; protective factors or biomarkers of interest (ALS); quality appraisal tool; funding information; conflicts of interest; number of studies included; number of participants and cases; and study design of the original study. Additionally, we gathered the most adjusted effect estimates (odds ratio [OR], relative risk [RR], hazard ratio [HR], weighted mean difference [WMD], and standardized mean difference [SMD]) along with the corresponding 95% CIs from the original studies. The most common adjustment factors comprised age, sex, body mass index (BMI), education, physical activity, smoking, alcohol consumption, and occupation.

The methodological quality of the included meta-analyses was evaluated using the robust and validated AMSTAR-2 tool (a measurement tool to assess systematic reviews). This tool rigorously evaluated the risk of bias, including ratings of search quality, reporting, analytics, and transparency. The overall methodological quality of each eligible meta-analysis was graded as high, moderate, low, or critical low (Shea et al., 2017).

### 2.4 Statistical analysis

First, we recalculated summary effect sizes and their 95% CIs for each eligible meta-analysis using random- and fixed-effect models to assess associations between environmental factors and biomarkers and ALS risk. When reporting weighted mean difference (WMD) in the included meta-analyses, we converted it to SMD according to an established formula (Carvalho et al., 2020). We also computed the 95% PIs, the probability range within which we predicted the effect size of the association would lie for 95% of similar future studies (Higgins et al., 2009). Subsequently, we performed Cochran’s Q test and calculated the *I*^2^ statistic to further assess statistical heterogeneity among the original studies. A *p*-value < 0.10 and *I*^2^ > 50% were considered to indicate significant heterogeneity. Additionally, Egger’s test was applied to evaluate publication bias and small-study effects, with a *p*-value less than 0.10, which was judged to be significant evidence of small-study effects. Finally, we applied an excess significance test to investigate whether the observed number of statistically significant studies was greater than expected (Ioannidis and Trikalinos, 2007). All statistical analyses were performed using STATA version 15.0. A *p*-value less than 0.05 was considered statistically significant for all tests, except for heterogeneity, small-study effects, and excess significant bias.

### 2.5 Credibility of epidemiologic evidence

In line with previously published umbrella reviews (Belbasis et al., 2015; Kim et al., 2019; Kim et al., 2020; Kim et al., 2022), we categorized the epidemiologic evidence regarding the relationship between environmental factors and biomarkers with ALS susceptibility into five strength levels based on specific assessment criteria (Supplementary Table 1). Convincing evidence (Class I) necessitated highly significant associations (*P* < 10^–6^ by random effects model), over 1,000 ALS cases, the largest study reporting a statistically significant result (*P* < 0.05), a 95% PI excluding the null value, no substantial heterogeneity (*I*^2^ < 50%), absence of excess significance bias (*P* > 0.10), and no significant small study effects (*P* > 0.10). Highly suggestive evidence (Class II) required statistically significant associations (*P* < 10^–6^ by random effects model) with more than 1,000 ALS cases, and the primary study component showed a statistically significant result (*P* < 0.05). Suggestive evidence (Class III) mandated only *P* < 10^–3^ by random effects model and over 1,000 cases. All other associations with nominal significance (*P* < 0.05) were classified as weak (Class IV). Lastly, evidence was deemed non-significant if no significance threshold was met (*P* > 0.05). Furthermore, for associations rated as convincing or highly suggestive, we conducted sensitivity analyses exclusively on nested case-control and cohort studies to evaluate any changes in the robustness of the epidemiologic evidence.

## 3 Results

### 3.1 Study identification

**FIGURE 1:**
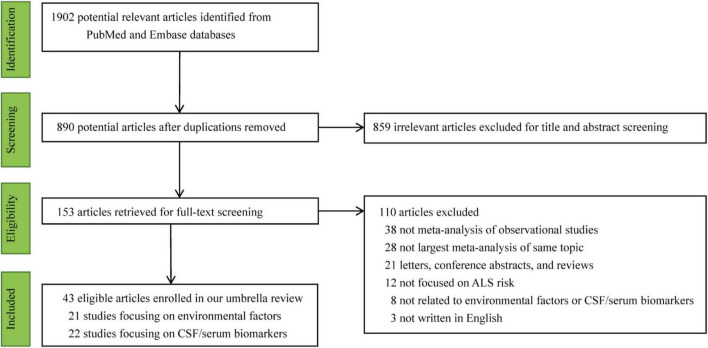
Flow diagram of literature search.

From database inception to 01 October 2024, we initially identified 1,902 articles by systematically searching PubMed and Embase. First, 890 repetitive and 859 irrelevant articles were excluded by reviewing the titles and abstracts. After full-text screening, 82 articles were excluded, comprising 38 non-meta-analyses of observational studies, 21 letters, conference abstracts, and reviews; 12 not focusing on ALS risk; 8 unrelated to environmental factors or cerebrospinal fluid (CSF)/serum biomarkers; and 3 not in English. To identify meta-analyses, the selection prioritized the meta-analysis with the most individual studies when multiple meta-analyses focused on the same association. In cases where multiple meta-analyses included the same largest number of studies, the most recently published meta-analysis was chosen. Subsequently, 28 articles were excluded due to the publication of a larger meta-analysis on the same topic. A list of all excluded publications is available in Supplementary Table 2. Therefore, 43 eligible articles published between 2013 and 2024 were included in this umbrella review. In total, 103 unique associations (46 potential environmental factors and 57 CSF/serum biomarkers) were extracted and are presented in Tables 1, 2 and Supplementary Tables 3, 4, respectively. The random-effects meta-analyses and corresponding funnel plots for all 103 associations are presented in Supplementary Datasheets 1, 2. The flow chart of the process of selecting eligible meta-analyses is presented in Figure 1.

**TABLE 1:** Characteristics of included meta-analyses evaluating associations between environmental factors and ALS risk.

Environmental factors	References	Effect metrics	Studies (*n*)	Subjects (*n*)	Cases (*n*)	Random effects model		The largest study SMD (95% CI)	Heterogeneity		Egger *p*-value	Excess significance *p*-value	95% PI
						OR/RR (95% CI)	*P*-value		*P*-value	*I* ^2^			
**Class I**													
Anti-hypertensives	Hu and Ji, 2022	OR	4	250,871	11,594	0.85 (0.81 to 0.88)	< 10^–6^	0.85 (0.81 to 0.88)	0.580	0.00%	0.756	1.000	0.78 to 0.93
**Class II**													
Trauma	Gu et al., 2021	OR	29	6,537,781	18,390	1.51 (1.32 to 1.73)	< 10^–6^	1.43 (1.33 to 1.54)	< 0.001	78.10%	0.657	1.000	0.87 to 2.63
Premorbid body mass index	Zeng et al., 2019	OR	11	5,314,782	5,673	0.97 (0.95 to 0.98)	< 10^–6^	0.98 (0.97 to 0.99)	0.017	52.50%	0.447	< 0.001	0.93 to 1.00
**Class III**													
Farming occupation	Kang et al., 2014	OR	10	2,621,006	9,338	1.42 (1.17 to 1.73)	< 0.001	1.20 (1.02 to 1.41)	0.080	41.70%	0.858	0.312	0.90 to 2.25
Pesticides	Kang et al., 2014	OR	15	3,732,028	9,534	1.44 (1.22 to 1.70)	< 0.001	1.20 (1.02 to 1.41)	0.048	41.30%	0.165	0.140	0.94 to 2.20
Head injury	Watanabe and Watanabe, 2017	OR	16	510,802	11,692	1.46 (1.20 to 1.74)	< 0.001	1.19 (0.88 to 1.61)	0.057	38.80%	0.933	0.790	0.88 to 2.39
Leisure time activity	Zheng et al., 2023	OR	8	10,569	3,327	1.08 (1.04 to 1.12)	< 0.001	1.07 (1.02 to 1.12)	0.519	0.00%	0.619	0.170	1.03 to 1.13
Anti-diabetes	Duan et al., 2023	OR	3	5,180	1,248	0.56 (0.41 to 0.78)	< 0.001	0.51 (0.43 to 0.60)	0.221	33.70%	0.525	0.043	0.02 to 12.68
Diabetes mellitus	Wannarong and Ungprasert, 2020	OR	11	5,410,951	11,961	0.68 (0.55 to 0.84)	< 0.001	0.98 (0.85 to 1.13)	< 0.001	81.10%	0.310	0.016	0.34 to 1.36
Kidney diseases	Duan et al., 2023	OR	3	136,375	11,735	0.84 (0.78 to 0.91)	< 0.001	0.84 (0.78 to 0.91)	0.650	0.00%	0.639	0.138	0.51 to 1.38
Smoking	Kim et al., 2024	OR	32	3,287,035	20,947	1.14 (1.06 to 1.23)	< 0.001	1.18 (1.07 to 1.30)	0.005	44.10%	0.531	0.557	0.88 to 1.47
Metals	Wang et al., 2014	OR	13	3,787	1,685	1.87 (1.51 to 2.33)	< 0.001	1.52 (0.95 to 2.42)	0.339	10.60%	0.040	0.418	1.29 to 2.72
**Class IV**													
Heavy metals	Duan et al., 2023	OR	7	7,285	1,311	1.80 (1.28 to 2.52)	0.001	1.45 (1.00 to 2.10)	0.083	46.40%	0.977	1.000	0.75 to 4.32
Lead	Meng et al., 2020	OR	12	25,872	4,246	1.46 (1.16 to 1.83)	0.001	1.07 (0.85 to 1.35)	0.019	51.80%	0.041	< 0.001	0.78 to 2.72
Annual PM2.5 exposure	Gong et al., 2023	OR	2	387,737	6,486	1.83 (1.01 to 3.35)	0.048	1.79 (1.00 to 3.39)	0.650	0.00%	NA	< 0.001	NA
Competitive organized sports	Blecher et al., 2019	RR	23	100,864	17,029	1.78 (1.11 to 2.86)	0.015	1.52 (1.03 to 2.25)	< 0.001	84.00%	0.789	0.581	0.23 to 13.78
Vigorous physical activity	Zheng et al., 2023	OR	17	9,088	2,849	1.26 (1.06 to 1.49)	0.008	1.03 (1.01 to 1.05)	< 0.001	74.50%	0.059	< 0.001	0.71 to 2.22
Occupational-related activity	Zheng et al., 2023	OR	12	12,277	4,025	1.14 (1.04 to 1.24)	0.005	1.06 (1.03 to 1.09)	< 0.001	77.90%	0.052	< 0.001	0.91 to 1.42
Unclassified physical activity	Zheng et al., 2023	OR	7	9,196	2,943	1.05 (1.02 to 1.09)	0.001	1.06 (1.04 to 1.09)	0.241	24.70%	0.517	0.016	0.99 to 1.12
Military personnel	Tai et al., 2017	OR	9	2,116,690	10,492	1.27 (1.06 to 1.54)	0.006	1.31 (1.09 to 1.58)	0.014	55.10%	0.748	0.761	0.77 to 2.16
Heavy physical work	Gunnarsson and Bodin, 2018	RR	9	494,435	2,097	1.89 (1.27 to 2.81)	0.002	0.95 (0.88 to 1.02)	< 0.001	87.20%	0.006	0.020	0.51 to 7.04
Chemicals	Gunnarsson and Bodin, 2018	RR	13	2,160,038	6,534	1.20 (1.06 to 1.35)	0.003	1.07 (0.93 to 1.23)	0.006	56.70%	0.060	0.002	0.85 to 1.68
Environmental and occupational solvents	Zhang and Zhou, 2023	OR	13	179,686	6,365	1.29 (1.08 to 1.55)	0.001	0.92 (0.77 to 1.09)	0.002	59.70%	0.159	0.035	0.78 to 2.19
ELF-MF	Jalilian et al., 2021	RR	27	11,207,625	22,673	1.20 (1.04 to 1.38)	0.008	1.09 (1.00 to 1.19)	< 0.001	66.30%	0.034	0.030	0.73 to 1.99
Stroke	Duan et al., 2023	OR	6	138,016	14,158	1.25 (1.06 to 1.47)	0.007	1.29 (1.15 to 1.44)	0.123	42.30%	0.782	0.057	0.67 to 2.35
ω-3 Polyunsaturated fatty acid intake	Fitzgerald et al., 2014	RR	5	1,056,837	994	0.66 (0.54 to 0.82)	< 0.001	0.64 (0.43 to 0.95)	0.923	0.00%	0.425	0.015	0.50 to 1.01
Carotenoids	Fitzgerald et al., 2013	RR	5	1,053,575	1,093	0.92 (0.87 to 0.97)	0.002	0.93 (0.86 to 1.01)	0.708	0.00%	0.623	0.475	0.86 to 0.98
Acetaminophen	Chang et al., 2020	OR	2	425,754	1,104	0.80 (0.67 to 0.96)	0.019	0.87 (0.71 to 1.07)	0.249	24.60%	NA	1.000	NA
Living in urban	Duan et al., 2023	OR	5	16,262	1,167	0.69 (0.48 to 1.00)	0.047	1.00 (0.83 to 1.20)	0.029	62.90%	0.121	0.023	0.01 to 63.81
**NS**													
Occupation in industry	Zhu et al., 2023	OR	3	866	264	1.24 (0.81 to 1.91)	0.328	1.48 (0.80 to 2.74)	0.665	0.00%	0.156	< 0.001	0.08 to 20.37
Annual PM10 exposure	Gong et al., 2023	OR	2	3,763	969	3.51 (0.63 to 19.36)	0.150	7.09 (0.79 to 64.83)	0.321	0.00%	NA	< 0.001	NA
Rural residence	Kang et al., 2014	OR	5	1,591	559	1.25 (0.84 to 1.87)	0.273	0.80 (0.54 to 1.18)	0.044	59.30%	0.635	0.219	0.35 to 4.51
Work with electricity	Gunnarsson and Bodin, 2018	RR	10	5,766,217	14,752	1.16 (1.00 to 1.36)	0.053	0.99 (0.90 to 1.09)	0.001	65.70%	0.235	< 0.001	0.74 to 1.82
Alcohol consumption	Duan et al., 2023	OR	11	12,110	4,430	1.02 (0.78 to 1.32)	0.895	0.72 (0.62 to 0.84)	< 0.001	84.40%	0.175	0.066	0.41 to 2.52
hypertension	Duan et al., 2023	OR	5	925,227	12,349	1.03 (0.98 to 1.08)	0.308	1.05 (1.01 to 1.10)	0.370	6.40%	0.290	1.000	0.88 to 1.19
NSAIDs	Duan et al., 2023	OR	3	31,753	1,880	1.08 (0.82 to 1.42)	0.581	1.04 (0.87 to 1.25)	0.007	17.70%	0.290	0.040	0.44 to 2.68
Welding	Gunnarsson and Bodin, 2018	RR	6	9,607,980	18,482	0.95 (0.70 to 1.29)	0.740	0.71 (0.57 to 0.89)	< 0.001	82.40%	0.154	1.000	0.34 to 2.61
Electric shocks	Jalilian et al., 2021	RR	8	2,273,307	14,036	0.97 (0.80 to 1.17)	0.754	0.73 (0.67 to 0.79)	< 0.001	80.50%	0.237	< 0.001	0.56 to 1.69
Statins	Hu and Ji, 2022	OR	10	3,588,732	27,698	0.91 (0.78 to 1.07)	0.166	0.87 (0.83 to 0.91)	< 0.001	92.00%	0.967	1.000	0.60 to 1.42
Aspirin	Chang et al., 2020	OR	3	793,588	1,548	0.94 (0.75 to 1.17)	0.583	1.04 (0.90 to 1.21)	0.077	61.10%	0.925	0.212	0.09 to 10.05
High vitamin diet	Duan et al., 2023	OR	3	1,171	409	0.95 (0.72 to 1.27)	0.740	1.08 (0.70 to 1.66)	0.723	0.00%	0.417	1.000	0.15 to 5.99
Sport-related activity	Zheng et al., 2023	OR	18	12,607	4,898	0.98 (0.76 to 1.26)	0.844	0.93 (0.72 to 1.20)	< 0.001	77.80%	0.419	0.002	0.37 to 2.58
AMI/IS	Duan et al., 2023	OR	6	162,136	16,371	0.96 (0.88 to 1.05)	0.412	0.96 (0.88 to 1.05)	0.606	0.00%	0.396	0.603	0.85 to 1.10
Cerebrovascular disease	Zhu et al., 2023	OR	2	51,293	1,238	0.99 (0.75 to 1.29)	0.928	1.12 (1.04 to 1.19)	0.004	88.10%	NA	0.513	NA
Occupation in service industry	Zhu et al., 2023	OR	2	580	205	0.47 (0.19 to 1.17)	0.105	0.33 (0.19 to 0.56)	0.129	56.60%	NA	0.005	NA
Coffee drinking	Duan et al., 2023	OR	3	960,249	1,819	0.80 (0.58 to 1.10)	0.169	0.98 (0.85 to 1.11)	0.005	81.20%	0.285	0.162	0.02 to 34.32

OR, odds ratio; RR, relative risk; CI, confidence intervals; PI, prediction interval; NA: not available; NS, not significant; ELF-MF, exposure to extremely-low frequency magnetic fields; NSAIDs, non-steroidal anti-inflammatory drugs; AMI/IS, acute myocardial infarction/ischemic stroke.

**TABLE 2:** Characteristics of included meta-analyses evaluating associations between biomarkers and ALS risk.

Biomarkers	References	Effect metrics	Studies (*n*)	Subjects (*n*)	Cases (*n*)	Random effects model		The largest study SMD/OR (95% CI)	Heterogeneity		Egger *p*-value	Excess significance *p*-value	95% PI
					SMD/OR (95% CI)	*P*-value		*P* value	*I* ^2^				
**Class II**													
CSF NFL	Sferruzza et al., 2022	SMD	23	2,887	1,901	2.06 (1.61 to 2.51)	< 10^–6^	0.87 (0.67 to 1.08)	< 0.001	94.80%	< 0.001	0.717	−0.16 to 4.28
Serum NFL	Sferruzza et al., 2022	SMD	15	1,781	1,074	1.57 (1.29 to 1.85)	< 10^–6^	1.45 (1.13 to 1.78)	< 0.001	81.80%	0.077	1.000	0.48 to 2.67
Serum ferritin	Cheng et al., 2021	SMD	9	2,880	1,661	0.66 (0.50 to 0.83)	< 10^–6^	0.46 (0.31 to 0.60)	< 0.001	70.80%	0.504	0.013	0.16 to 1.17
Serum uric acid	Zhang et al., 2018	SMD	8	2,559	1,168	−0.72 (−0.98 to −0.46)	< 10^–6^	−1.07 (−1.20 to −0.94)	< 0.001	87.90%	0.503	0.091	−1.58 to 0.14
**Class III**													
Serum transferrin	Wang et al., 2020	SMD	6	1,837	1,129	−0.27 (−0.39 to −0.16)	< 0.001	−0.30 (−0.44 to −0.17)	0.347	10.80%	0.844	1.000	−0.47 to −0.08
**Class IV**													
CSF CHIT1	Xu et al., 2024	SMD	6	798	580	1.92 (0.78 to 3.06)	< 0.001	0.92 (0.59 to 1.26)	< 0.001	96.50%	0.201	0.519	0.03 to 3.81
CSF cystatin C	Zhu et al., 2018	SMD	6	396	246	−1.40 (−2.43 to −0.36)	0.008	−0.29 (−0.64 to 0.06)	< 0.001	94.50%	0.079	0.018	−4.84 to 2.04
CSF TNF-α	Chen et al., 2018	SMD	6	318	175	0.36 (0.04 to 0.67)	0.028	0.66 (0.20 to 1.12)	0.095	46.70%	0.211	0.002	−0.44 to 1.15
CSF MIP-1α	Chen et al., 2018	SMD	6	490	292	0.90 (0.10 to 1.71)	0.028	2.09 (1.69 to 2.48)	< 0.001	93.60%	0.365	< 0.001	−1.81 to 3.61
CSF MCP-1	Chen et al., 2018	SMD	7	509	283	0.58 (0.40 to 0.75)	< 10^–6^	0.43 (0.10 to 0.75)	0.588	0.00%	0.745	0.450	0.35 to 0.80
CSF IL-17	Chen et al., 2018	SMD	5	267	151	0.74 (0.49 to 0.99)	< 10^–6^	0.71 (0.25 to 1.17)	0.631	0.00%	0.863	0.665	0.39 to 1.10
CSF IL-15	Chen et al., 2018	SMD	4	241	120	0.46 (0.03 to 0.88)	0.035	0.00 (−0.45 to −0.45)	0.067	58.00%	0.734	0.014	−0.80 to 1.71
CSF G-CSF	Chen et al., 2018	SMD	5	417	251	0.55 (0.35 to 0.76)	< 0.001	0.30 (−0.03 to 0.62)	0.374	5.70%	0.269	0.107	0.22 to 0.89
CSF IL-2	Chen et al., 2018	SMD	5	417	251	0.34 (0.02 to 0.66)	0.039	0.32 (−0.01 to 0.64)	0.050	57.90%	0.678	1.000	−0.55 to 1.22
CSF NFH	Xu et al., 2016	SMD	6	710	443	1.01 (0.54 to 1.49)	< 0.001	0.36 (0.08 to 0.64)	< 0.001	84.30%	0.007	0.006	−0.49 to 2.52
CSF TDP-43	Gambino et al., 2023	SMD	7	472	254	0.66 (0.23 to 1.10)	0.003	0.32 (−0.03 to 0.67)	< 0.001	79.10%	0.227	0.012	−0.71 to 2.03
CSF homocysteine	Hu and Wang, 2023	SMD	3	338	169	2.78 (0.61 to 4.95)	0.012	0.67 (0.17 to 1.17)	< 0.001	97.90%	0.110	1.000	−6.67 to 12.23
CSF t-tau	Thapa et al., 2023	SMD	7	1,100	634	1.76 (0.52 to 2.99)	0.005	−0.55 (−0.82 to −0.27)	< 0.001	98.50%	0.026	0.020	−2.56 to 6.07
CSF CHI3L1	Xu et al., 2024	SMD	5	556	369	3.16 (1.25 to 5.06)	0.001	0.85 (0.41 to 1.29)	< 0.001	97.40%	0.208	< 0.001	−3.37 to 9.68
Serum 8-OHdG	Wang et al., 2019	SMD	2	79	18	2.20 (0.56 to 3.83)	0.009	3.02 (2.14 to 3.90)	0.012	84.20%	NA	1.000	−15.22 to 19.61
Serum GSH	Wang et al., 2019	SMD	2	79	18	2.20 (0.56 to 3.83)	0.009	3.02 (2.14 to 3.90)	0.012	84.20%	NA	1.000	−15.22 to 19.61
Serum AOPP	Wang et al., 2019	SMD	2	280	147	0.56 (0.32 to 0.79)	< 0.001	0.54 (0.20 to 0.87)	0.876	0.00%	NA	1.000	−0.99 to 2.10
Serum MDA	Wang et al., 2019	SMD	5	243	123	1.17 (0.81 to 1.52)	< 10^–6^	0.97 (0.56 to 1.38)	0.216	30.80%	0.411	1.000	0.37 to 1.97
Serum lead	Farace et al., 2022	SMD	9	984	409	0.60 (0.12 to 1.07)	0.013	0.50 (0.30 to 0.70)	< 0.001	88.90%	0.567	1.000	−0.99 to 2.18
Serum TNF-α	Hu et al., 2017	SMD	12	822	456	0.66 (0.28 to 1.03)	0.001	0.50 (0.20 to 0.79)	< 0.001	82.70%	0.328	0.781	−0.67 to 1.98
Serum TNFR1	Hu et al., 2017	SMD	3	246	155	0.74 (0.47 to 1.01)	< 10^–6^	0.71 (0.33 to 1.09)	0.965	0.00%	0.995	1.000	0.15 to 1.33
Serum IL-1β	Hu et al., 2017	SMD	4	304	161	0.29 (0.01 to 0.58)	0.038	0.50 (0.20 to 0.79)	0.283	21.20%	0.500	0.627	−0.33 to 0.92
Serum IL-6	Hu et al., 2017	SMD	7	509	267	0.25 (0.07 to 0.43)	0.006	0.40 (0.11 to 0.69)	0.512	0.00%	0.808	0.434	0.03 to 0.47
Serum IL-8	Hu et al., 2017	SMD	6	434	242	0.45 (0.26 to 0.64)	< 0.001	0.50 (0.20 to 0.79)	0.471	0.00%	0.868	0.692	0.20 to 0.70
Serum IL-17	Gautam et al., 2023	SMD	2	79	50	0.64 (0.16 to 1.12)	0.009	0.78 (0.14 to 1.41)	0.517	0.00%	NA	1.000	−2.46 to 3.75
Serum VEGF	Hu et al., 2017	SMD	3	199	107	0.89 (0.29 to 1.49)	0.003	0.40 (0.01 to 0.79)	0.029	71.80%	0.368	0.044	−1.40 to 3.18
Serum FBG	Cheng et al., 2021	SMD	10	542	301	0.20 (0.01 to 0.40)	0.040	−0.02 (−0.30 to 0.27)	0.337	11.50%	0.066	0.404	−0.13 to 0.53
Serum CK	Cheng et al., 2021	SMD	5	2,072	229	0.74 (0.27 to 1.20)	0.002	0.72 (0.42 to 1.02)	0.034	61.70%	0.200	0.687	−0.53 to 2.00
Serum TSC	Cheng et al., 2021	SMD	3	1,344	858	0.23 (0.11 to 0.34)	< 0.001	0.20 (0.06 to 0.34)	0.758	0.00%	0.509	1.000	−0.03 to 0.48
Serum TIBC	Cheng et al., 2021	SMD	3	540	236	−0.25 (−0.43 to −0.07)	0.006	−0.16 (−0.42 to 0.09)	0.350	4.70%	0.141	0.490	−0.67 to 0.17
Serum creatinine	Liu et al., 2020b	SMD	5	1,920	983	−0.78 (−0.97 to −0.60)	< 10^–6^	−0.78 (−0.90 to −0.66)	0.031	62.40%	0.907	1.000	−1.30 to −0.27
Serum folic	Hu and Wang, 2023	SMD	3	338	169	2.78 (0.61 to 4.95)	0.012	0.67 (0.17 to 1.17)	< 0.001	97.90%	0.110	1.000	−6.67 to 12.23
Serum miR-206	Liu et al., 2023	SMD	5	211	110	0.76 (0.49 to 1.04)	< 10^–6^	0.63 (0.18 to 1.08)	0.494	0.00%	0.029	0.022	0.37 to 1.15
Serum miR-338-3p	Liu et al., 2023	SMD	3	252	139	0.47 (0.21 to 0.72)	< 0.001	0.34 (0.001 to 0.69)	0.538	0.00%	0.29	0.250	−0.09 to 1.03
Serum miR-133b	Liu et al., 2023	SMD	2	46	26	1.18 (0.56 to 1.79)	< 0.001	1.04 (0.22 to 1.87)	0.624	0.00%	NA	0.545	−2.81 to 5.17
Serum miR-133a	Liu et al., 2023	SMD	2	46	26	0.77 (0.18 to 1.36)	0.010	0.68 (−0.12 to 1.47)	0.729	0.00%	NA	1.000	−3.05 to 4.60
**NS**													
CSF VEGF	Chen et al., 2018	SMD	9	619	365	0.51 (−0.001 to 1.01)	0.051	1.93 (1.55 to 2.32)	< 0.001	88.30%	0.043	< 0.001	−1.26 to 2.28
CSF lead	Farace et al., 2022	SMD	6	244	114	0.51 (−0.01 to 1.03)	0.057	0.10 (−0.35 to 0.55)	0.002	72.90%	0.124	0.315	−1.06 to 2.08
CSF *p*-tau	Thapa et al., 2023	SMD	6	933	577	0.12 (−0.38 to 0.62)	0.645	0.82 (0.72 to 0.94)	< 0.001	94.80%	0.043	< 0.001	−1.55 to 1.78
Serum NFH	Xu et al., 2016	SMD	2	195	117	1.07 (−0.08 to 2.21)	0.068	0.53 (0.20 to 0.86)	0.005	87.50%	NA	0.531	−11.24 to 13.37
Serum iron	Wang et al., 2020	SMD	7	1,796	1,055	0.48 (−0.07 to 1.03)	0.086	1.20 (1.05 to 1.35)	< 0.001	95.40%	0.22	0.003	−1.38 to 2.34
Serum HDL	Cheng et al., 2021	SMD	14	7,045	2,674	−0.01 (−0.19 to 0.17)	0.909	−0.02 (−0.13 to 0.09)	< 0.001	87.70%	0.480	< 0.001	−0.66 to 0.63
Serum LDL	Liu et al., 2020a	SMD	6	2,990	1,495	−0.01 (−0.12 to 0.10)	0.907	0.08 (−0.03 to 0.18)	0.152	38.10%	0.987	0.602	−0.26 to 0.24
Serum TC	Liu et al., 2020a	SMD	6	2,981	1,495	−0.06 (−0.21 to 0.09)	0.453	0.06 (−0.05 to 0.17)	0.019	63.00%	0.952	0.121	−0.46 to 0.34
Serum TG	Liu et al., 2020a	SMD	8	3,727	1,918	−0.08 (−0.17 to 0.01)	0.080	−0.05 (−0.16 to 0.06)	0.194	29.30%	0.508	1.000	−0.27 to 0.11
Serum homocysteine	Hu and Wang, 2023	SMD	11	3,444	812	0.50 (0.04 to 0.97)	0.034	−0.03 (−0.21 to 0.14)	< 0.001	95.70%	0.223	< 0.001	−1.26 to 2.27
Serum vitamin B12	Hu and Wang, 2023	SMD	4	521	252	−0.00 (−0.20 to 0.19)	0.984	−0.16 (−0.39 to 0.08)	0.347	9.20%	0.345	1.000	−0.37 to 0.36
Serum galectin	Ramos-Martínez et al., 2022	SMD	2	180	70	0.30 (−0.04 to 0.63)	0.085	0.17 (−0.21 to 0.54)	0.283	13.20%	NA	1.000	−2.18 to 2.77
Serum selenium	Zhou et al., 2023	SMD	10	995	425	−0.27 (−1.09 to 0.55)	0.525	−2.08 (−2.34 to −1.82)	< 0.001	96.10%	0.281	< 0.001	−3.35 to 2.81
Serum vitamin D	Lanznaster et al., 2020	SMD	4	439	163	−0.75 (−1.61 to 0.12)	0.090	−1.75 (−2.07 to −1.42)	< 0.001	93.30%	0.598	0.450	−3.77 to 2.28
Serum ApoA1	Chalitsios et al., 2024	OR	2	1,062,073	1,514	0.77 (0.60 to 1.00)	0.053	0.82 (0.72 to 0.94)	0.250	24.40%	NA	1.000	NA
Serum ApoB	Chalitsios et al., 2024	OR	2	1,062,073	1,514	0.77 (0.60 to 1.00)	0.053	0.82 (0.72 to 0.94)	0.250	24.40%	NA	1.000	NA

CSF, cerebrospinal fluid; SMD, standardized mean difference; CI, confidence intervals; PI, prediction interval; NA, not available; NFL, neurofilaments light chain; TNF, tumor necrosis factor; MIP, macrophage inflammatory proteins; MCP, monocyte chemoattractant protein; IL, interleukin; G-CSF, granulocyte colony-stimulating factor; NFH, neurofilaments heavy chain; TDP-43, an RNA-binding protein; 8-OHdG, 8-hydroxyguanosine; GSH, glutathione; AOPP, Advanced Oxidation Protein Product; MDA, malondialdehyde; TNFR1, TNF receptor 1; VEGF, vascular endothelial growth factor; FBG, fasting blood glucose; CK, creatine kinase; TSC, transferrin saturation coefficient; TIBC, total iron binding capacity; miR, microRNA; HDL, high-density lipoprotein; LDL, low-density lipoprotein; TC, total cholesterol; TG, triglyceride; ApoA1, apolipoprotein A1; ApoB, apolipoprotein B; t-tau, total tau; *p*-tau, phosphorylated-tau; CHIT1, chitotriosidase; CHI3L1, chitinase 3-like 1.

### 3.2 Methodological quality assessment of meta-analyses

The AMSTAR 2 quality assessment tool was utilized to evaluate all 43 meta-analysis articles in our umbrella review. Among the potential environmental factors, 2 out of 21 meta-analyses (10%) were deemed high quality, 1 (5%) was rated as moderate quality, 6 (29%) were considered low quality, and 12 (57%) were classified as critically low quality (Supplementary Table 5). Of the 22 meta-analysis articles focusing on potential biomarkers, only 3 (14%) were rated as moderate, 9 (41%) as low, and 10 (45%) as critically low (Supplementary Table 5). Overall, most meta-analysis researchers did not register protocols before conducting the review (30 studies, 70%), which significantly impacts methodological quality. Consequently, we performed a supplementary analysis that did not consider the absence of a registered protocol as a critical flaw when reassessing the methodological quality of the included studies. The results of the supplementary analysis showed that AMSTAR 2 ratings were reclassified as high in 3 studies (7%), moderate in 10 studies (23%), and low (18 studies, 42%) or critically low (12 studies, 28%) in 43 studies (Supplementary Table 6).

### 3.3 Environmental risk and protective factors

The 46 associations between environmental factors and ALS susceptibility were based on 344,597 ALS cases, a total population of 71,415,574, a median of 4,664 ALS cases per meta-analysis (interquartile range 1,370–11,904, range 205–27,698), and a median of 215,279 subjects per meta-analysis (interquartile range 12,152–2,149,201, range 580–11,207,625). Among these meta-analyses, 42 were case-control studies, with 26 including cohort studies. The median number of study estimates was eight (interquartile range 3–12, range 2–29). The effect metrics used to evaluate the relationships between environmental factors and ALS risk were RR and OR. Twenty-nine of the 46 associations (63%) were statistically significant under the random effect model, with 13 (45%) having *P* < 10^–3^ and 3 (10%) having *P* < 10^–6^. Of these 29 statistically significant associations, 28 (97%) included more than 1,000 ALS cases, and 13 (45%) exhibited substantial heterogeneity (*I*^2^ > 50%). Additionally, 12 associations (41%) were statistically significant without small study effects or excess significance bias, and the 95% PI excluded the null value in 5 (17%) of the 29 associations.

**FIGURE 2:**
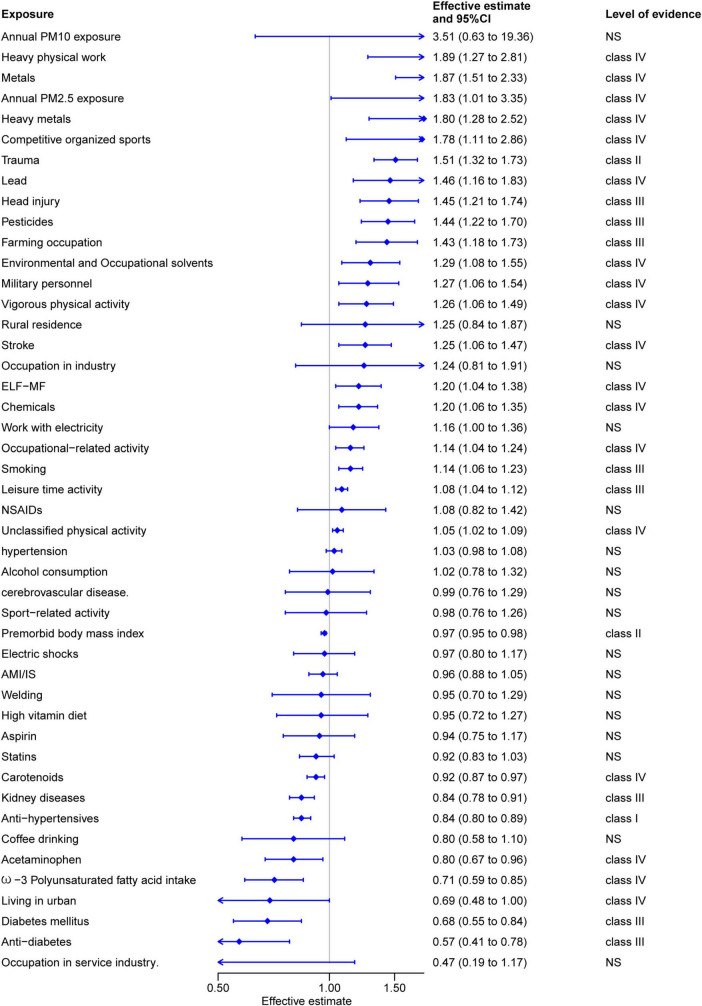
Summary estimates of environmental risk and protective factors for amyotrophic lateral sclerosis.

As shown in Figure 2, the summary effect size, along with its corresponding 95% CI, was calculated to assess the relationships between various environmental factors and ALS risk. Out of 46 associations of environmental risk/protective factors, the sole environmental protective factor classified as convincing evidence (Class I) was the regular use of antihypertensive drugs (OR: 0.85, 95% CI: 0.81 to 0.88) (Donohue et al., 2022). Furthermore, premorbid body mass index (OR: 0.97, 95% CI: 0.95 to 0.98) (Zeng et al., 2019) and trauma (OR: 1.51, 95% CI: 1.32 to 1.73) (Gu et al., 2021) were graded as highly suggestive evidence (class II) for environmental protective and risk factors, respectively. Nine environmental risk/protective factors were categorized as suggestive evidence (Class III), among which farming occupation (OR: 1.42, 95% CI: 1.17 to 1.73) (Kang et al., 2014), pesticides exposure (OR: 1.44, 95% CI: 1.22 to 1.70) (Kang et al., 2014), head injuries (OR: 1.46, 95% CI: 1.20 to 1.74) (Watanabe and Watanabe, 2017), leisure time activity (OR: 1.08, 95% CI: 1.04 to 1.12) (Zheng et al., 2023), and metals exposure (OR: 1.87, 95% CI: 1.51 to 2.33) (Wang et al., 2014) were identified as environmental risk factors. Conversely, anti-diabetes (OR: 0.56, 95% CI: 0.41 to 0.78) (Duan et al., 2023), diabetes mellitus (OR: 0.68, 95% CI: 0.55 to 0.84) (Wannarong and Ungprasert, 2020), smoking (OR: 1.14, 95% CI: 1.06 to 1.23) (Kim et al., 2024), and kidney diseases (OR: 0.84, 95% CI: 0.78 to 0.91) (Duan et al., 2023) were recognized as environmental protective factors. Additionally, 13 other risk factors and four protective factors were statistically significant but with weak evidence certainty (Figure 2). Ultimately, it was determined that the remaining 17 environmental factors had no significant impact on ALS (*P* > 0.05).

### 3.4 CSF and serum biomarkers

A total of 57 associations focusing on CSF/serum biomarkers with ALS susceptibility were based on 30,941 ALS cases, 2,180,797 individuals in the total population, a median of 254 ALS cases per meta-analysis (interquartile range 151–812, range 18–2,674), and a median of 509 subjects per meta-analysis (interquartile range 267–1,781, range 46–7,045). All these studies were cohort, case-control, or cross-sectional studies. The median number of study estimates in each meta-analysis was five (interquartile range 3–7, range 2–23). The effect metrics used to assess the association between various biomarkers and ALS risk were SMD and OR. Among 57 associations, 41 (72%) associations were nominally statistically significant at *P* < 0.05, 19 of 57 (33%) at *P* < 10^–3^, and 10 of 57 (18%) at *P* < 10^–6^. Among the 41 statistically significant associations, five (12%) enrolled more than 1,000 ALS cases, and 22 (54%) exhibited large heterogeneity (*I*^2^ > 50%). Additionally, 15 (37%) statistically significant associations suggested hints for small study effects or excessive significance bias. Lastly, in 13 (32%) associations with *P* < 0.05, the 95% PI excluded the null value.

**FIGURE 3:**
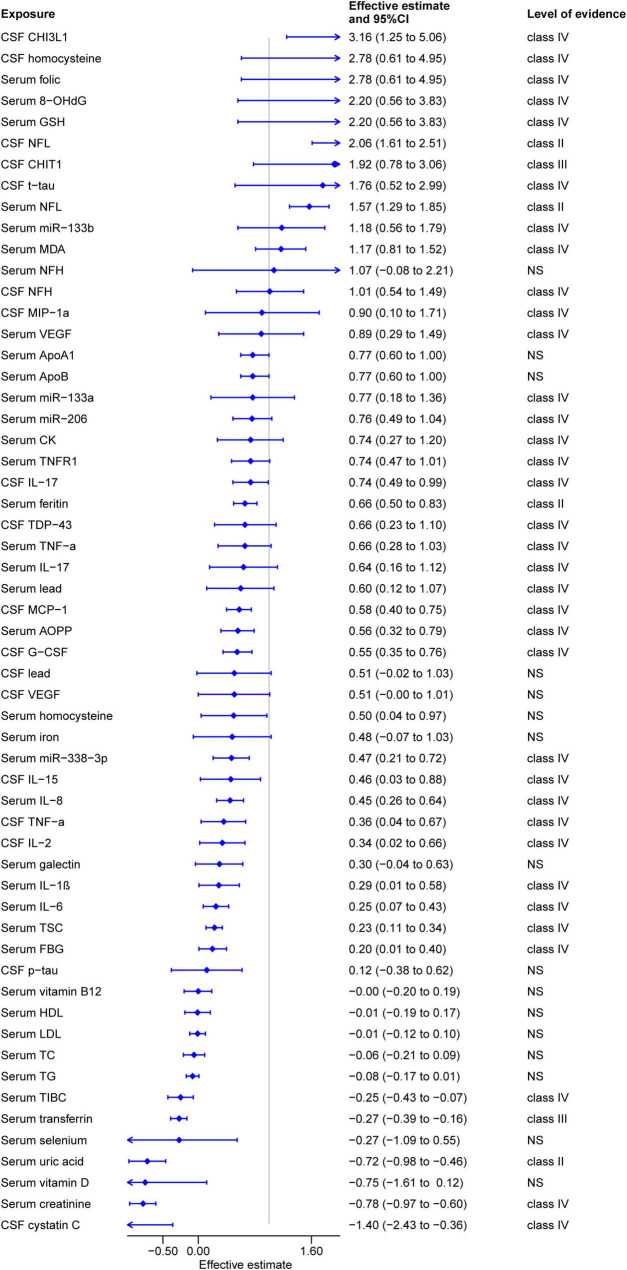
Summary estimates of cerebrospinal fluid and serum biomarkers for amyotrophic lateral sclerosis.

Among the 57 CSF/serum biomarker associations, 15 CSF and 26 serum biomarkers were significantly associated with ALS (Figure 3). Unfortunately, none of these biomarkers received a grade of convincing evidence (Class I). Three serums and one CSF biomarker exhibited highly suggestive evidence (Class II). Specifically, serum levels of neurofilament light chains (NFL) (SMD: 1.57, 95% CI: 1.29 to 1.85) (Sferruzza et al., 2022), CSF NFL levels (SMD: 2.06, 95% CI: 1.61 to 2.51) (Sferruzza et al., 2022), and serum ferritin levels (SMD: 0.66, 95% CI: 0.50 to 0.83) (Cheng et al., 2021) were significantly higher in ALS patients, while uric acid levels (SMD: −0.72; 95% CI: −0.98 to −0.46) (Wang et al., 2019) were lower in ALS patients compared to controls. Serum transferrin levels (SMD: −0.27; 95% CI: −0.39 to −0.16) (Wang et al., 2020) were classified as suggestive evidence (Class III). In contrast, 14 other CSF biomarkers were categorized as weak evidence (Class IV), including CSF NFH, TDP-43, TNF-α, MIP-1α, MCP-1, G-CSF, IL-2, IL-15, IL-17, cystatin C, CHIT1, CHI3L1, t-tau, and homocysteine levels. Similarly, 22 serum biomarkers were also considered weak evidence, encompassing serum 8-OHdG, GSH, AOPP, MDA, TNF-α, TNFR1, IL-1β, IL-6, IL-8, IL-17, VEGF, FBG, CK, TSC, TIBC, lead, creatinine, folic, miR-133a, miR-133b, miR-206, and miR-338–3p levels. Furthermore, no significant impact of the remaining 16 biomarkers on ALS was detected (*P* > 0.05).

### 3.5 Results of sensitivity analysis

TTo evaluate the robustness of the seven associations categorized as convincing or highly suggestive, we conducted sensitivity analyses exclusively utilizing cohort and nested case-control studies. The evidence supporting the link between trauma history and ALS risk, comprising four cohort studies and one nested case-control study, remained consistent (Class II, highly suggestive). Conversely, the association between premorbid body mass index and the onset of ALS was reclassified to suggestive evidence. Regrettably, the meta-analysis on antihypertensive drug usage incorporated solely one cohort study. Notably, investigations on CSF NFL, serum NFL, ferritin, and uric acid levels did not encompass any cohort or nested case-control studies. Consequently, a sensitivity analysis for these associations was infeasible.

## 4 Discussion

### 4.1 Principal findings

ALS is a fatal disease characterized by neuronal degeneration that has garnered significant attention from numerous scholars. An increasing number of meta-analyses have aimed to evaluate the reliability and certainty of epidemiological evidence regarding the association of various environmental factors and biomarkers with ALS incidence. However, the published data often present inconsistent or conflicting findings. Therefore, we conducted an umbrella review that systematically assessed all recently published meta-analyses investigating 103 potential associations between ALS and different environmental factors and biomarkers. We applied stringent criteria to evaluate the credibility of eligible meta-analyses. Seven factors or biomarkers were identified as providing convincing or highly suggestive evidence (trauma, use of antihypertensive drugs, premorbid body mass index, CSF NFL levels, serum NFL levels, ferritin levels, and uric acid levels), indicating their potential significance in the development of ALS. Among these factors, the use of antihypertensive drugs and premorbid body mass index were associated with a decreased risk of ALS, while trauma was linked to increased susceptibility. Moreover, elevated CSF NFL levels, serum NFL levels, and ferritin levels, as well as decreased serum uric acid levels, were observed in ALS patients compared to controls, suggesting their potential utility as reliable biomarkers for the onset and progression of ALS. Belbasis et al. (2016) conducted a similar umbrella review in 2016, consolidating eight meta-analyses to explore 11 associations between various environmental factors and ALS incidence. The previous study identified long-term occupational exposure to lead as a compelling environmental risk factor for ALS occurrence, with head injury being considered highly suggestive. In contrast, our study encompassed 43 meta-analyses involving 344,597 ALS cases and 71 million participants. Following a meticulous evaluation using robust methods, we reclassified the association of lead exposure and head injury with ALS risk as weak and suggestive evidence in our umbrella review.

### 4.2 Possible explanations

Antihypertensive medications can significantly decrease the incidence of ALS, which may be attributed to multiple mechanisms. First, hypertension has been linked to maladaptation of cerebral circulation, resulting in dysregulation of cerebral blood flow and disruption of the blood–brain barrier (BBB) (Ungvari et al., 2021). BBB disruption permits the entry of neurotoxic blood-derived debris, cells, and microbial pathogens into the brain, triggering inflammation and immune responses that activate various ALS pathways. BBB breakdown has been observed to directly cause early motor neuron impairment and dysfunction in ALS mouse models, and early preservation of BBB integrity can postpone the onset of motor neuron injury and degeneration (Winkler et al., 2014). Additionally, neuroimaging studies of living human brains and postmortem tissue analyses have confirmed BBB disruption in the initial states of ALS (Sweeney et al., 2018; Mirian et al., 2022). Hence, antihypertensive medications might lower the incidence of ALS by averting BBB dysfunction (Kucuk et al., 2002; Katsi et al., 2020). Second, the most commonly prescribed antihypertensive drugs, ACEIs, β-blockers, and CCBs, could reduce the risk of ALS through distinct neuroprotective mechanisms (Donohue et al., 2022). Specifically, ACEI might promote neuronal survival by scavenging free radicals and providing protection against glutamate-induced neurotoxicity (Ravati et al., 1999; Sengul et al., 2011). β-blockers could diminish neuroinflammation by modulating macrophages and microglia from pro-inflammatory to anti-inflammatory phenotypes, thereby contributing to neuroprotective effects (Lin et al., 2020). Moreover, calcium dysregulation can induce motor neuron degeneration by directly or indirectly impacting crucial proteins involved in ALS neurodegeneration, such as VAP-B, Matrin 3, and alsin (Leal and Gomes, 2015). Therefore, individuals with ALS and chronic hypertension may derive additional benefits beyond blood pressure reduction from regular use of antihypertensive medications, which should be recommended in forthcoming clinical practice.

In our umbrella review, a history of trauma, graded as highly suggestive, was linked to an increased risk of ALS. Trauma, particularly repeated trauma, contributes to a sustained low-level pro-inflammatory state in the body, resulting in the overexpression of various circulating inflammatory factors such as IL-1, IL-6, and TNF-α (Lenz et al., 2007). These pro-inflammatory cytokines can lead to excessive activation of microglia in the central nervous system (CNS), causing chronic neuroinflammation (Potgieter et al., 2015; Ritzel et al., 2015). Activated microglia also release significant amounts of pro-inflammatory factors, attracting more peripheral immune cells to migrate to the CNS (Huang et al., 2021). Given that neuroinflammation plays a crucial role in ALS pathogenesis, the close association between trauma and ALS susceptibility is understandable.

Traumatic brain injury (TBI), a prevalent form of trauma, could also increase the risk of developing ALS through various mechanisms. Anomalous phosphorylation and translocation of TDP-43 to the cytoplasm play crucial roles in ALS pathogenesis (Dewey et al., 2011; Correia et al., 2015). A prior autopsy study revealed TDP-43 inclusions in 61 out of 71 TBI cases across multiple brain regions such as the brainstem, basal ganglia, and diencephalon (Smith et al., 2013). Recent research has validated that TBI can trigger ALS-related TDP-43 pathological alterations in mouse models, possibly due to TBI-induced inflammation promoting NF-κB-mediated TDP-43 overexpression (McKee et al., 2009; McKee et al., 2013; Wiesner et al., 2018; Gao et al., 2022). Additionally, a study using an ALS fly model indicated that TBI may prompt stress granule formation in the brain, potentially leading to motor neuron degeneration (Anderson et al., 2018). In certain instances, TBI can directly compromise the BBB breakdown (Shlosberg et al., 2010). Specifically, localized head trauma can disrupt BBB regulation by damaging the endothelium of small blood vessels (Rodríguez-Baeza et al., 2003). It is widely recognized that the exacerbation of motor neuron damage resulting from BBB impairment is a significant factor in ALS pathogenesis (Garbuzova-Davis et al., 2011). Therefore, TBI could increase ALS susceptibility by affecting several crucial pathophysiological processes involved in ALS onset. Notably, our umbrella review encompassing a meta-analysis of 11,692 ALS cases has substantiated a statistically significant link between a history of head injury and ALS risk (OR 1.45, 95% CI 1.21 to 1.74). Unfortunately, the evidence level for this correlation was deemed suggestive, as the *p*-value of 8.0 × 10^–5^ was very close but did not reach 10^–6^.

In our study, highly suggestive evidence demonstrated that premorbid BMI was inversely associated with the risk of ALS. One possible explanation for this is that individuals susceptible to ALS are more likely to be in a hypermetabolic state (low BMI) (O’Reilly et al., 2013). Previous studies have shown that mice with ALS exhibit increased energy expenditure, skeletal muscle hypermetabolism, and reduced adipose tissue levels prior to symptom onset (Dupuis et al., 2004). A recent observational study showed that approximately 50% of patients were hypermetabolic at diagnosis, and up to 80% had no change in metabolic status during 2 years of follow-up, suggesting that in most cases, hypermetabolism may occur early during ALS (Bouteloup et al., 2009). Moreover, Mark et al. suggested that low-energy diets might render motor neurons vulnerable to degeneration. In contrast, high-energy diets could induce adaptive responses in neuronal populations, activate signaling pathways that promote plasticity and disease resistance, and initiate a neuroprotective response to energy stress (Mattson et al., 2007). These findings suggest that metabolic dysfunction plays a key role in the pathogenesis of ALS (Batulan et al., 2003; Sinclair, 2005; Perera and Turner, 2016). Another explanation is that BMI is closely related to type II diabetes (T2D), and T2D is involved in ALS occurrence, indicating that BMI may indirectly affect ALS susceptibility through the T2D pathway (Corbin et al., 2016; D’Ovidio et al., 2018). Mechanistically, higher blood glucose levels may act as unintentional compensation to meet the higher energy expenditure of damaged motor neurons (Fergani et al., 2007; Dupuis et al., 2011; Zhang et al., 2020). Additionally, the potential anti-inflammatory effects of metformin and sulphonylureas may suppress neuroinflammation in patients with ALS (Han et al., 2018). However, the exact mechanisms underlying the relationship between BMI and ALS are complex and should be interpreted cautiously.

Regarding robust serum biomarkers, circulating ferritin levels were significantly higher in patients with ALS than in healthy controls. One possible explanation is that the disruption of iron homeostasis may cause neuronal death, which plays an important role in the pathogenesis of ALS (Rathore et al., 2012; Kim and Connor, 2020; Obrador et al., 2020). The ferritin complex releases the stored iron by triggering autophagy (Gao et al., 2016; Hou et al., 2016). Subsequently, excessive iron may increase the generation of reactive oxygen species through the Fenton reaction, thus inducing ferroptosis and apoptosis due to the failure of redox control (Dixon et al., 2012; Conrad and Pratt, 2019). Interestingly, serum uric acid levels were reduced in patients with ALS compared with controls, suggesting a protective effect of uric acid on ALS. This can be explained by the following mechanism. First, uric acid can assist in scavenging superoxides by inhibiting the degradation of superoxide dismutase to reduce neurotoxicity, ultimately exerting neuroprotective effects (Kutzing and Firestein, 2008). Second, uric acid can also chelate iron, preventing increased free radical production to further reduce oxidative damage (Davies et al., 1986). Finally, uric acid has been demonstrated to protect neurons from damage by reducing glutamate toxicity (Du et al., 2007).

Similarly, CSF and serum NFL levels were significantly increased in patients with ALS compared to controls, which was rated as highly suggestive evidence. In multiple animal models of ALS, axonal damage precedes motor neuron death and symptom onset (Fischer and Glass, 2007). As a crucial structural component of axons, NFL is vital for maintaining normal axonal diameter and conduction velocity (Brettschneider et al., 2006). Additionally, NFL can be released into CSF and serum through axonal degeneration (Brettschneider et al., 2006). In our umbrella review, patients with ALS exhibited elevated CSF and serum NFL levels, reflecting extensive damage to motor neurons and axons and serving as important diagnostic markers (Bjornevik et al., 2021; Falzone et al., 2021). While both CSF and serum NFL levels offer potential diagnostic accuracy, serial lumbar puncture for monitoring NFL levels is significantly less practical than blood collection, making blood-borne NFL a more favorable surrogate marker for ALS patients (Puentes et al., 2014; McCombe et al., 2015).

### 4.3 Strengths and limitations

To the best of our knowledge, our umbrella review offers the most comprehensive and systematic evaluation of all published meta-analyses on environmental risk factors, protective factors, and biomarkers of ALS susceptibility. We applied stringent criteria to assess the methodological quality and strength of evidence in each eligible meta-analysis. Furthermore, we emphasized sensitivity analysis and biological plausibility to enhance the accuracy of evaluating each environmental factor and biomarker. However, it is important to note several limitations of this study. First, we focused solely on associations synthesized by published meta-analyses, potentially overlooking important relationships not yet evaluated using meta-analytic methods. Second, it is worth acknowledging that biomarker studies included relatively small sample sizes, ranging from 46 to 7,045 cases without longitudinal data. Therefore, there is a need for more multicenter, large-sample, prospective biomarker studies. Third, in our umbrella review, the AMSTAR 2 criteria revealed that among the 43 included meta-analyses, two (5%) were rated as high quality, four (9%) as moderate, 15 (35%) as low, and 22 (51%) as critically low quality. A significant factor contributing to these lower-quality ratings was the absence of protocol registration, noted in 30 (70%) of the included meta-analyses. While this methodological concern did not substantially alter our overall findings, future researchers are strongly encouraged to prospectively register study protocols and adhere closely to standardized reporting guidelines such as PRISMA and the Meta-analysis of Observational Studies in Epidemiology (MOOSE) to enhance transparency and reliability in this research field. Fourth, the original studies were susceptible to confounding biases in observational meta-analyses. To mitigate this issue, most original studies adjusted for known confounding factors to minimize their impact on the results. Our umbrella review extracted the fully adjusted effect estimates for further analysis. However, due to variations in the adjustment models across the original studies, we cannot completely rule out the possibility of residual confounding in some effect estimates, which could potentially distort the true effect sizes. Finally, out of the 70 statistically significant associations, 35 (50%) exhibited heterogeneity, which could compromise the robustness of our results despite most estimates demonstrating significant effects consistently. Therefore, it is advisable to exercise caution when interpreting these results.

## 5 Conclusion

In summary, we provide a comprehensive overview of 103 potential environmental risk factors, protective factors, and biomarkers for ALS susceptibility. Following rigorous criteria to evaluate the epidemiological evidence, seven factors and biomarkers show convincing or highly suggestive evidence, including trauma, use of antihypertensive drugs, premorbid body mass index, circulating NFL, ferritin, and uric acid levels, and CSF NFL levels. Further research is needed to enhance understanding of the complex mechanisms through which these factors impact ALS development.

## Data Availability

The original contributions presented in this study are included in this article/Supplementary material, further inquiries can be directed to the corresponding authors.
