# Supplementary material for: Environmental risk factors, protective factors, and biomarkers for amyotrophic lateral sclerosis: an umbrella review
Source: Front Aging Neurosci. 2025 Jun 13;17:1541779. doi: 10.3389/fnagi.2025.1541779 (PMC12202415; doi:10.3389/fnagi.2025.1541779)
Supplement: Supplementary file 1 [file Data_Sheet_1.pdf]

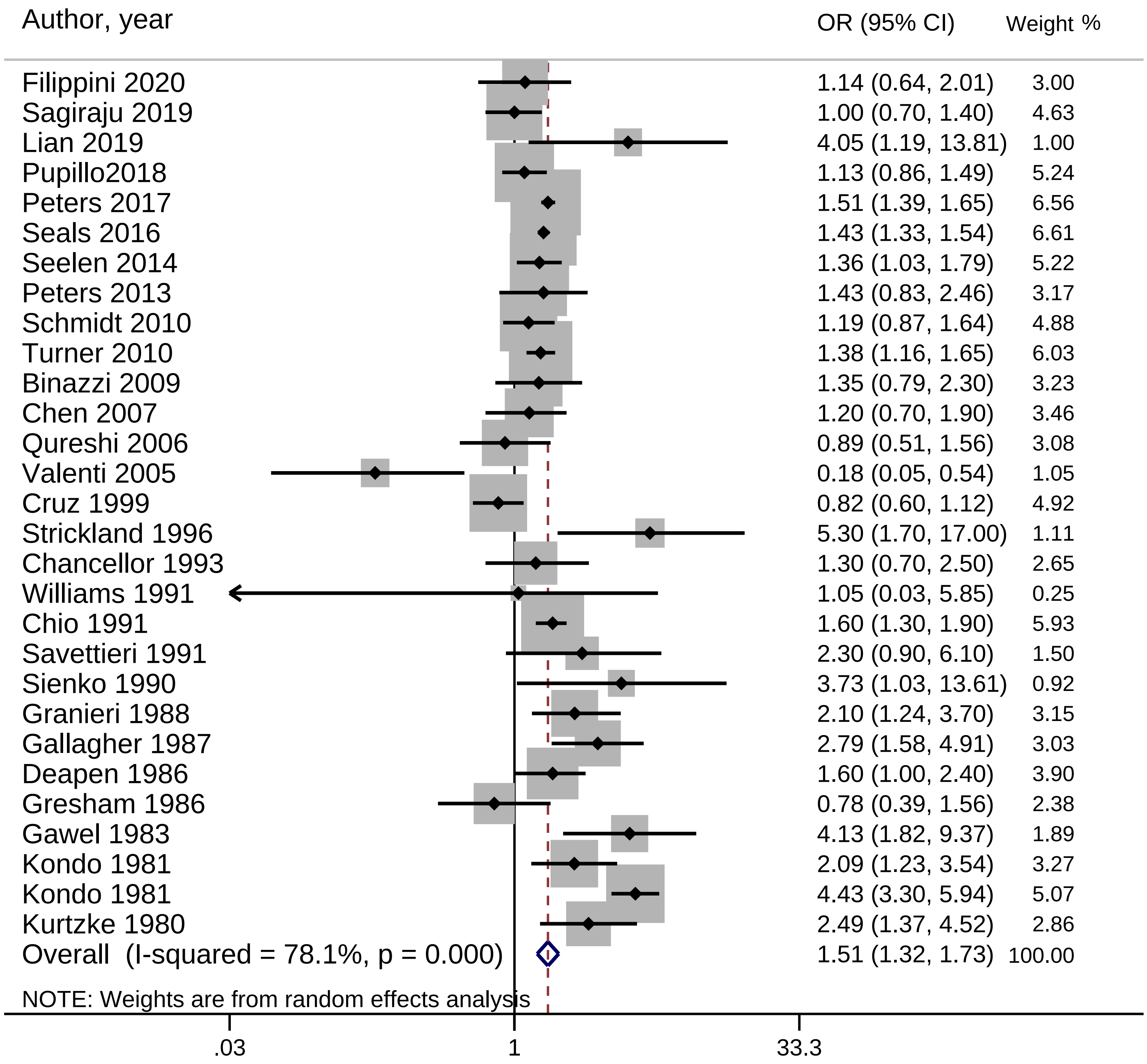

Figure S1. Random-effects meta-analysis of the association between trauma and ALS

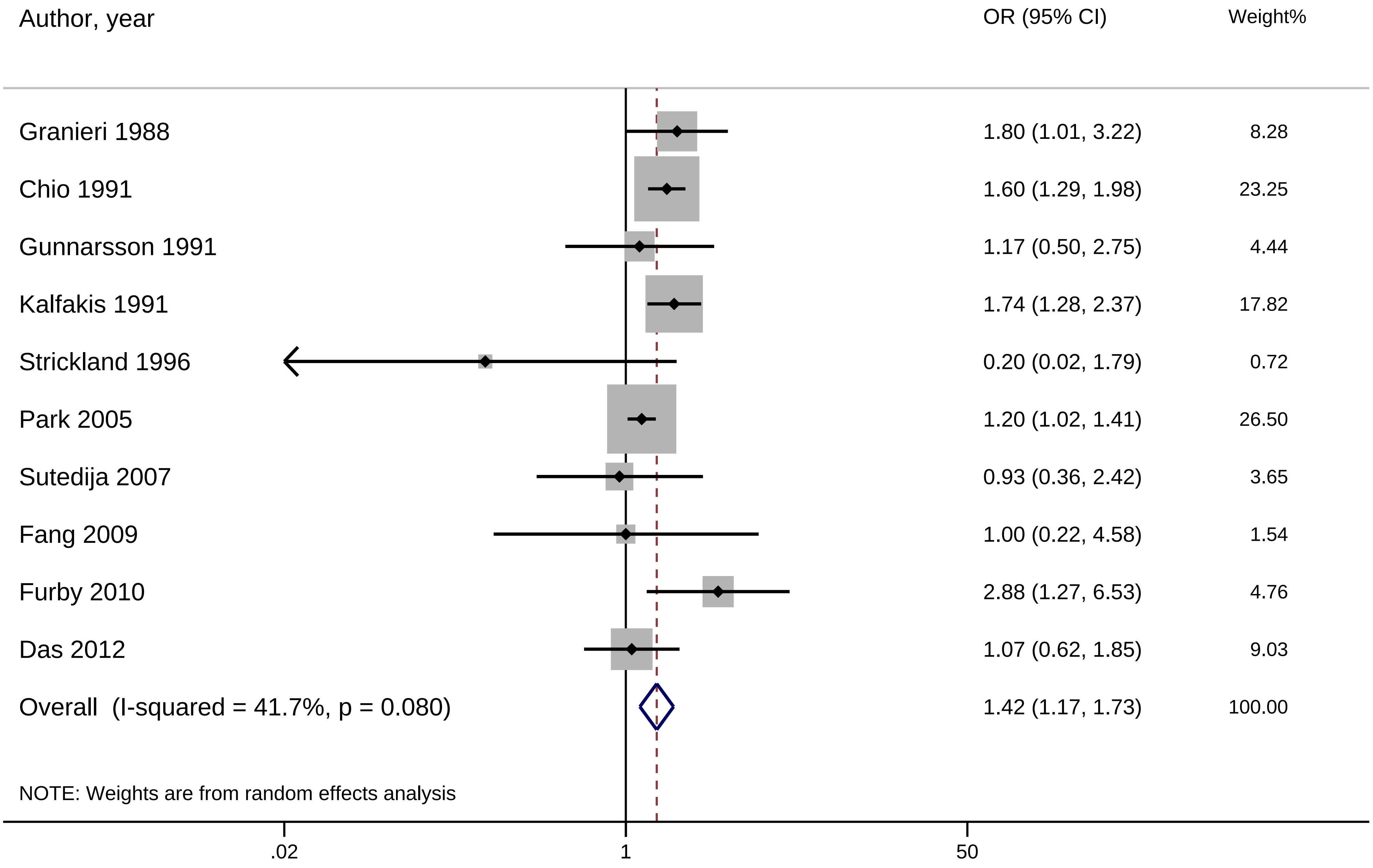

Figure S2. Random-effects meta-analysis of the association between farming occupation and ALS

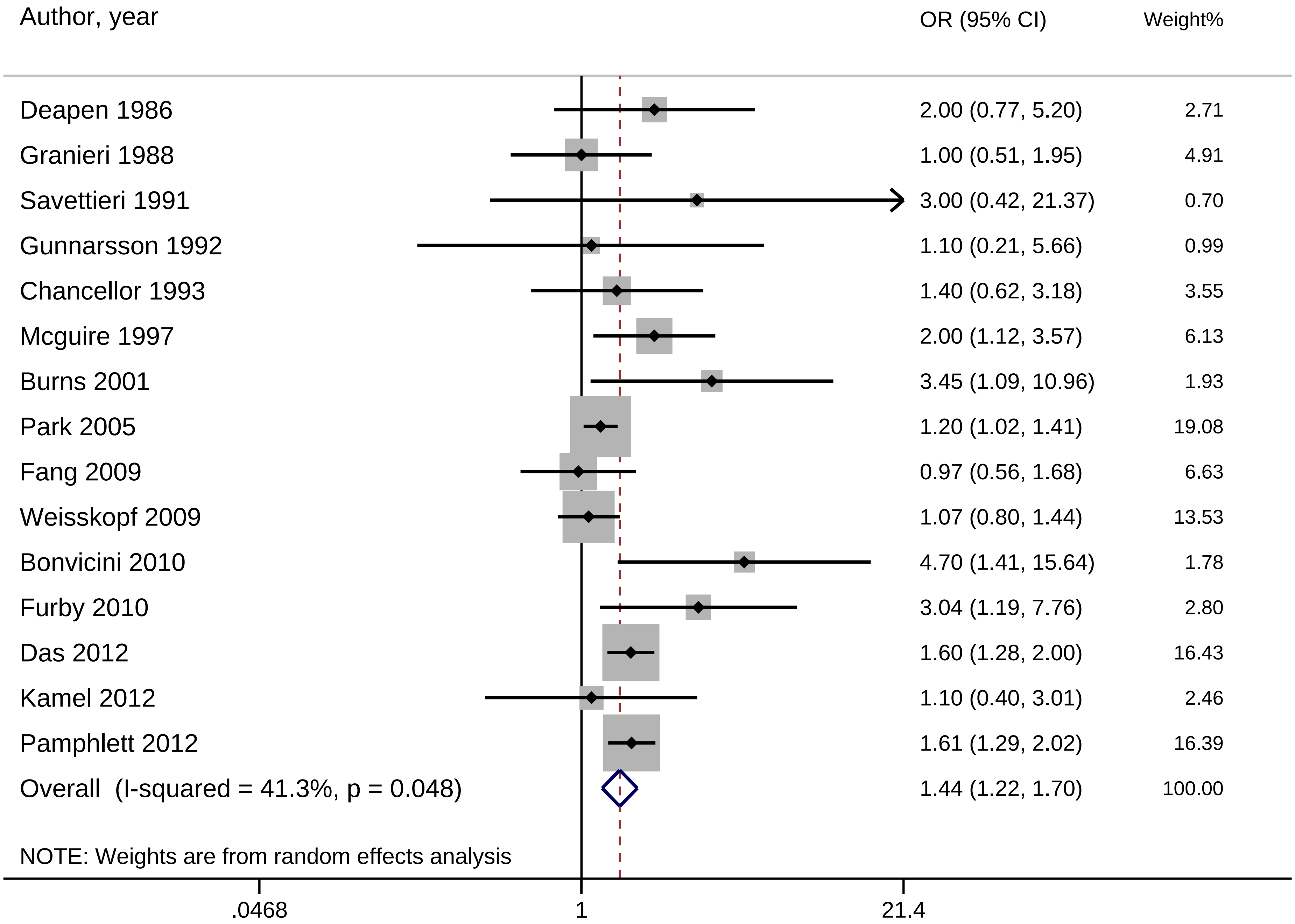

Figure S3. Random-effects meta-analysis of the association between pesticide and ALS

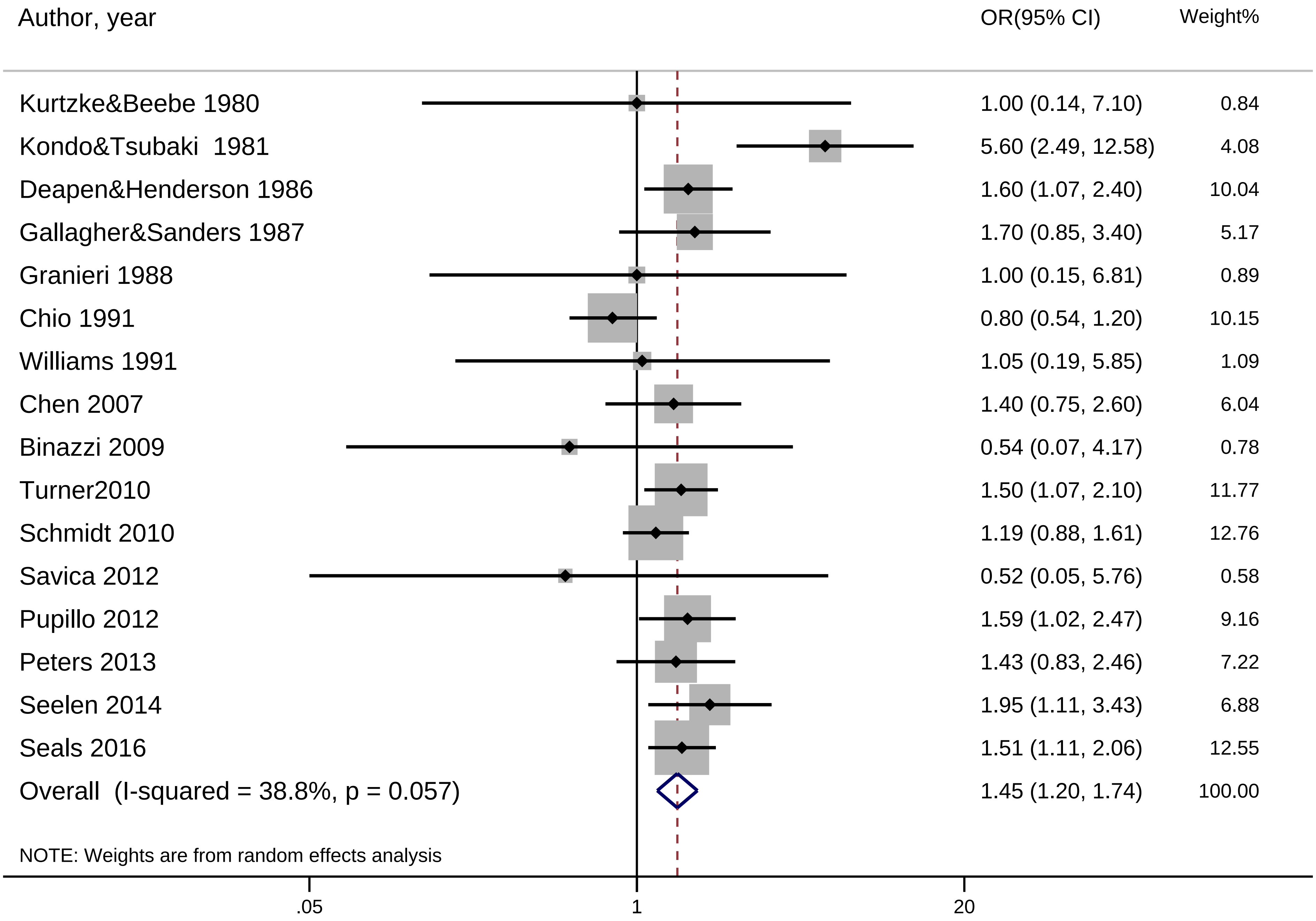

Figure S4. Random-effects meta-analysis of the association between head injury and ALS

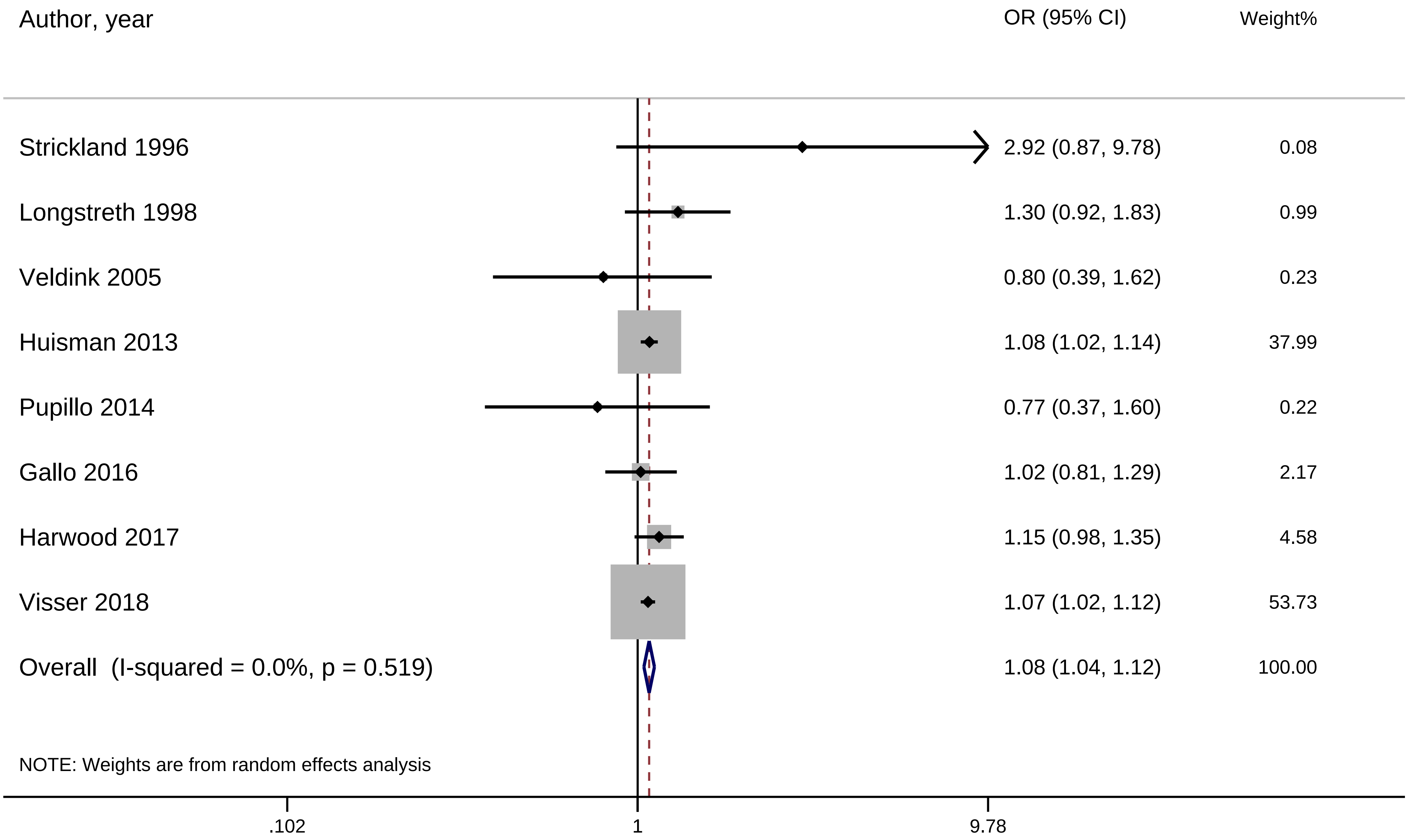

Figure S5. Random-effects meta-analysis of the association between leisure time activity and ALS

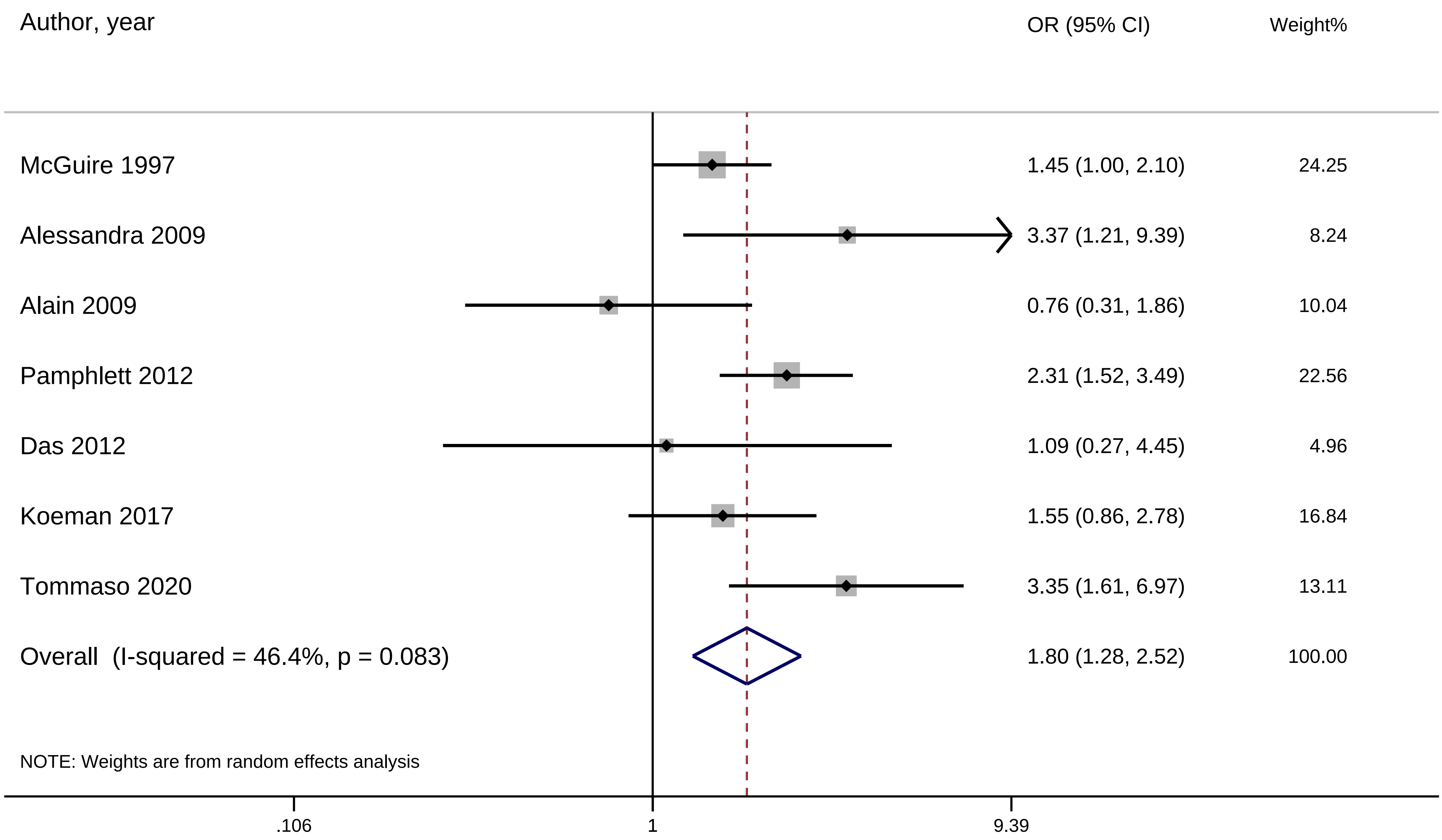

Figure S6. Random-effects of meta-analysis of the association between heavy metals and ALS

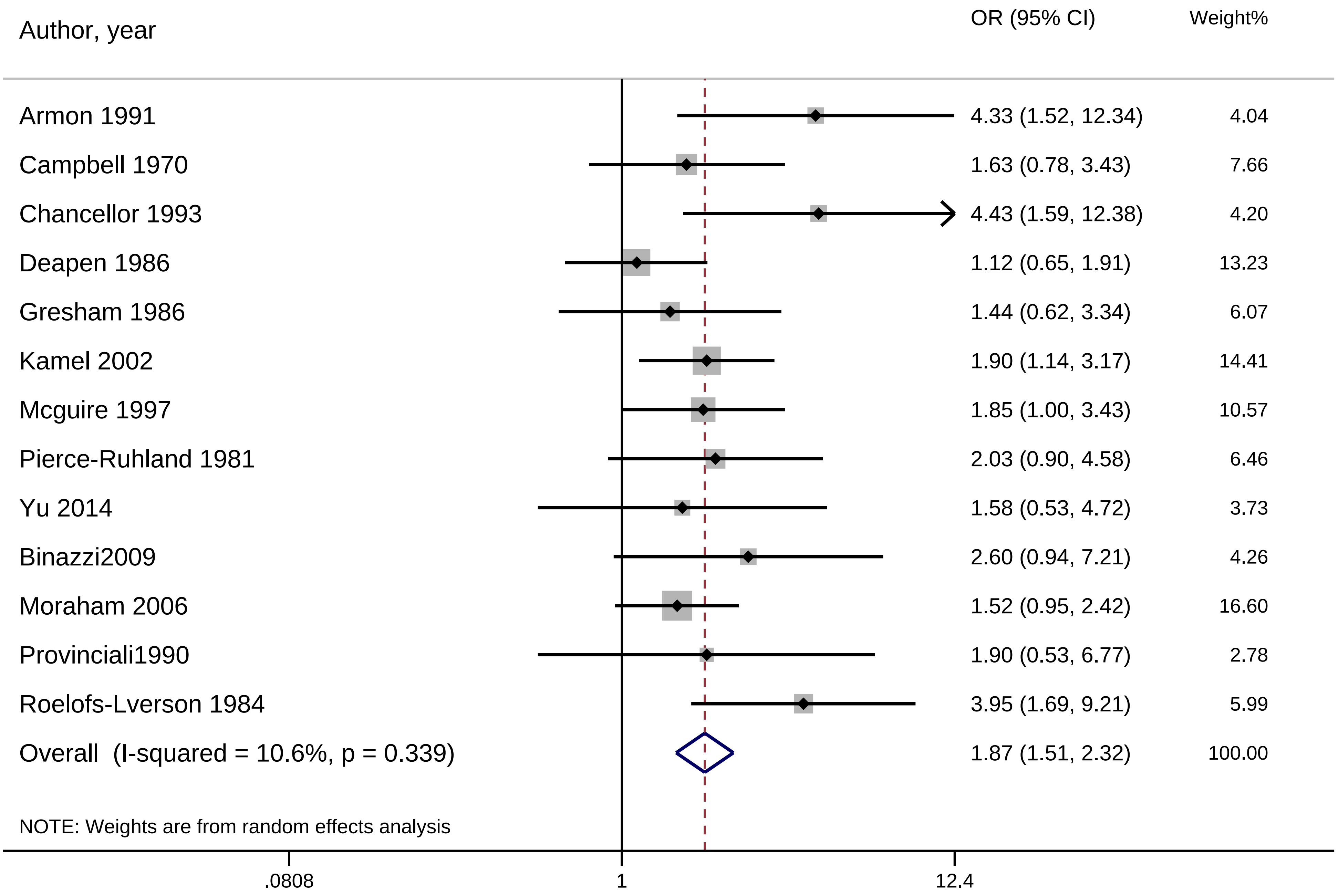

Figure S7. Random-effects meta-analysis of the association between metals and ALS

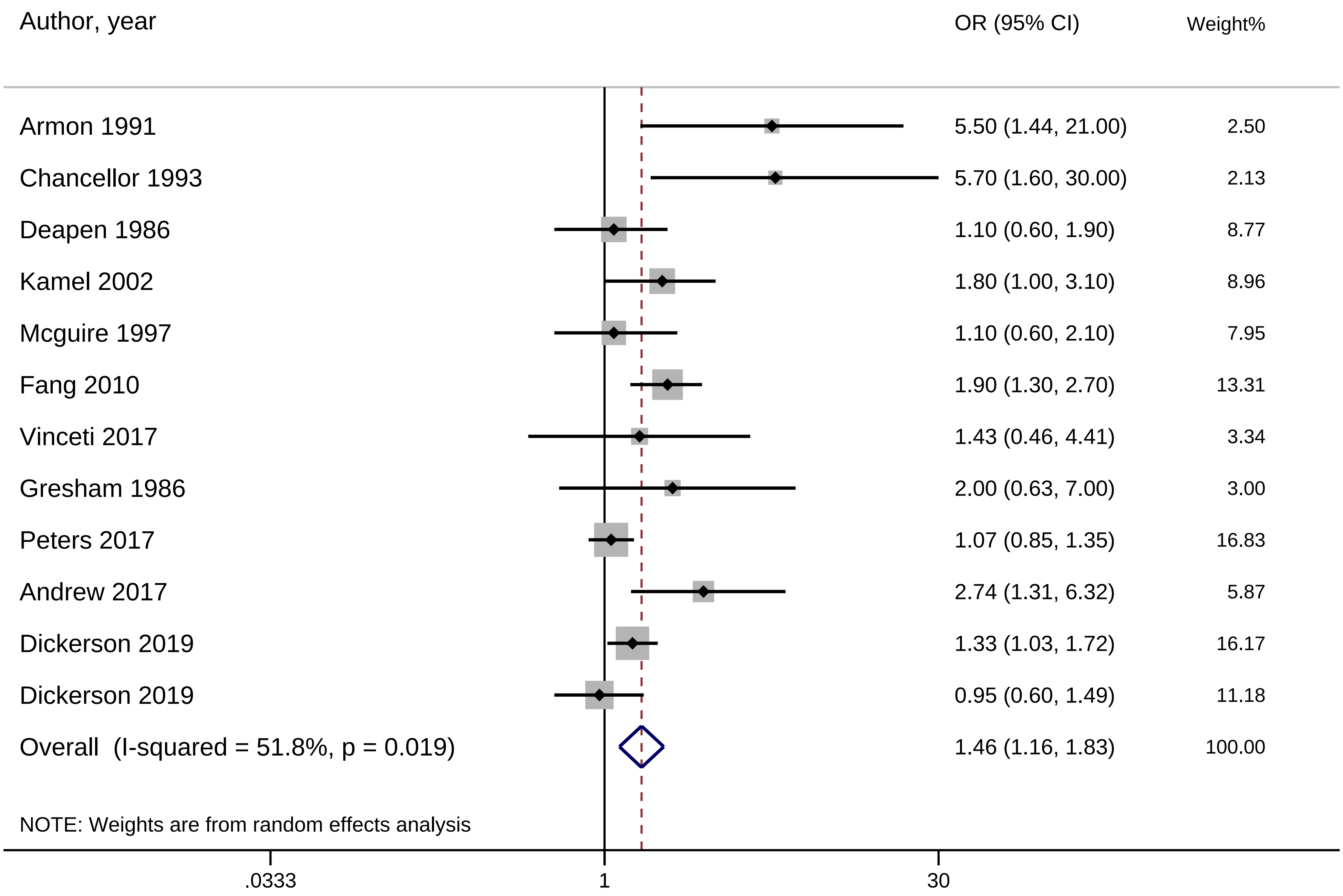

Figure S8. Random-effects meta-analysis of the association between lead and ALS

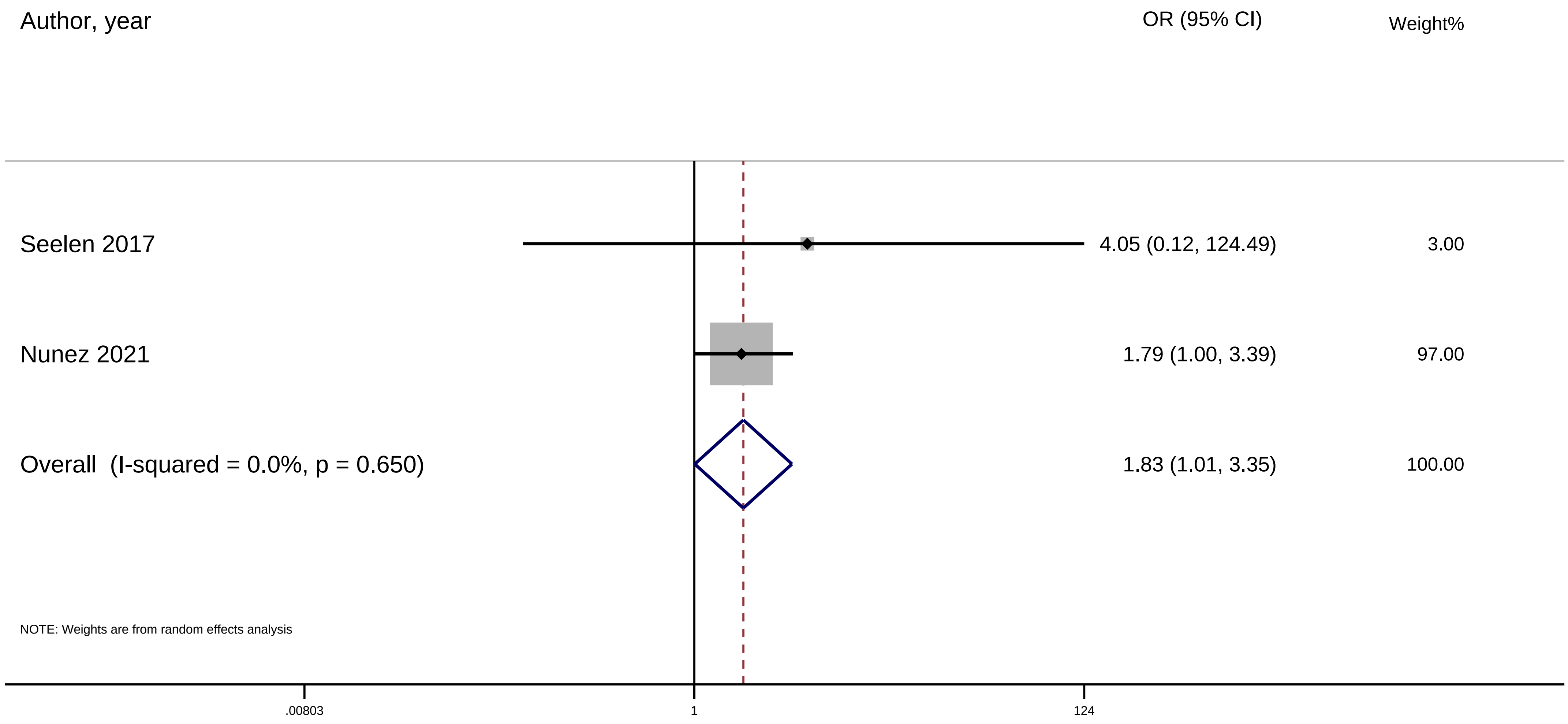

Figure S9. Funnel plot of meta-analysis of the association between annual PM2.5 exposure and ALS

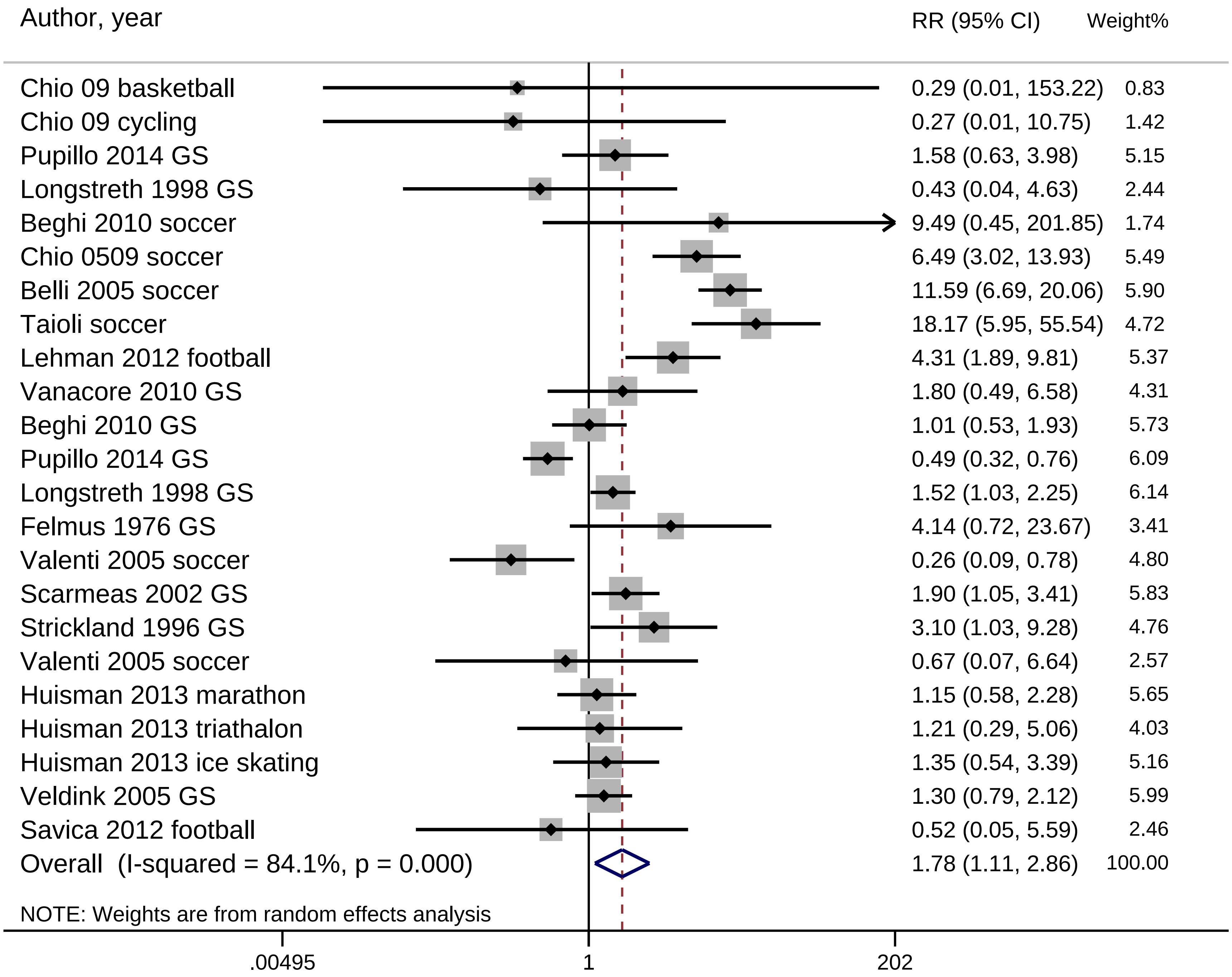

Figure S10. Random-effects meta-analysis of the association between competitive organized sports and ALS

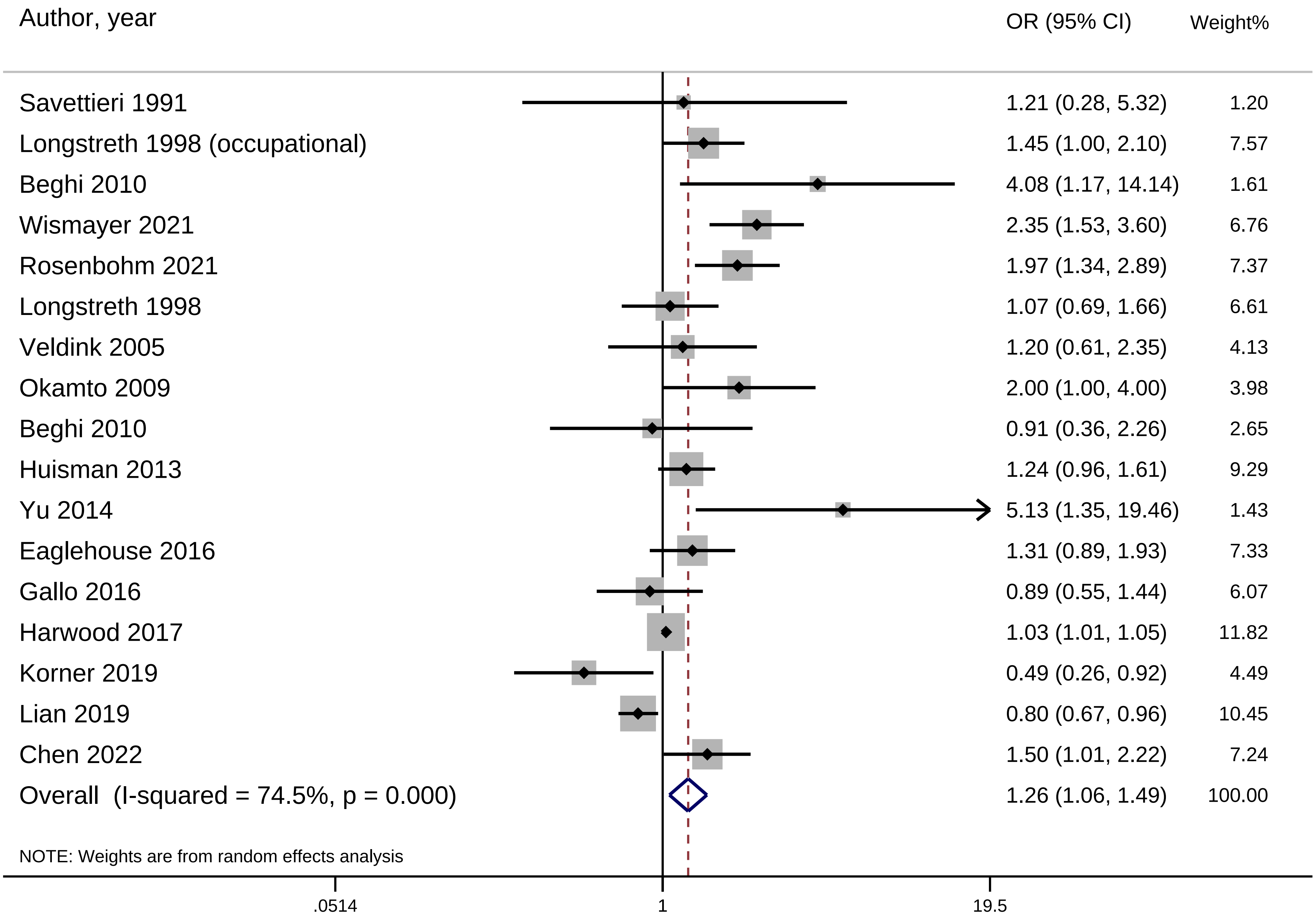

Figure S11. Random-effects meta-analysis of the association between vigorous physical activity and ALS

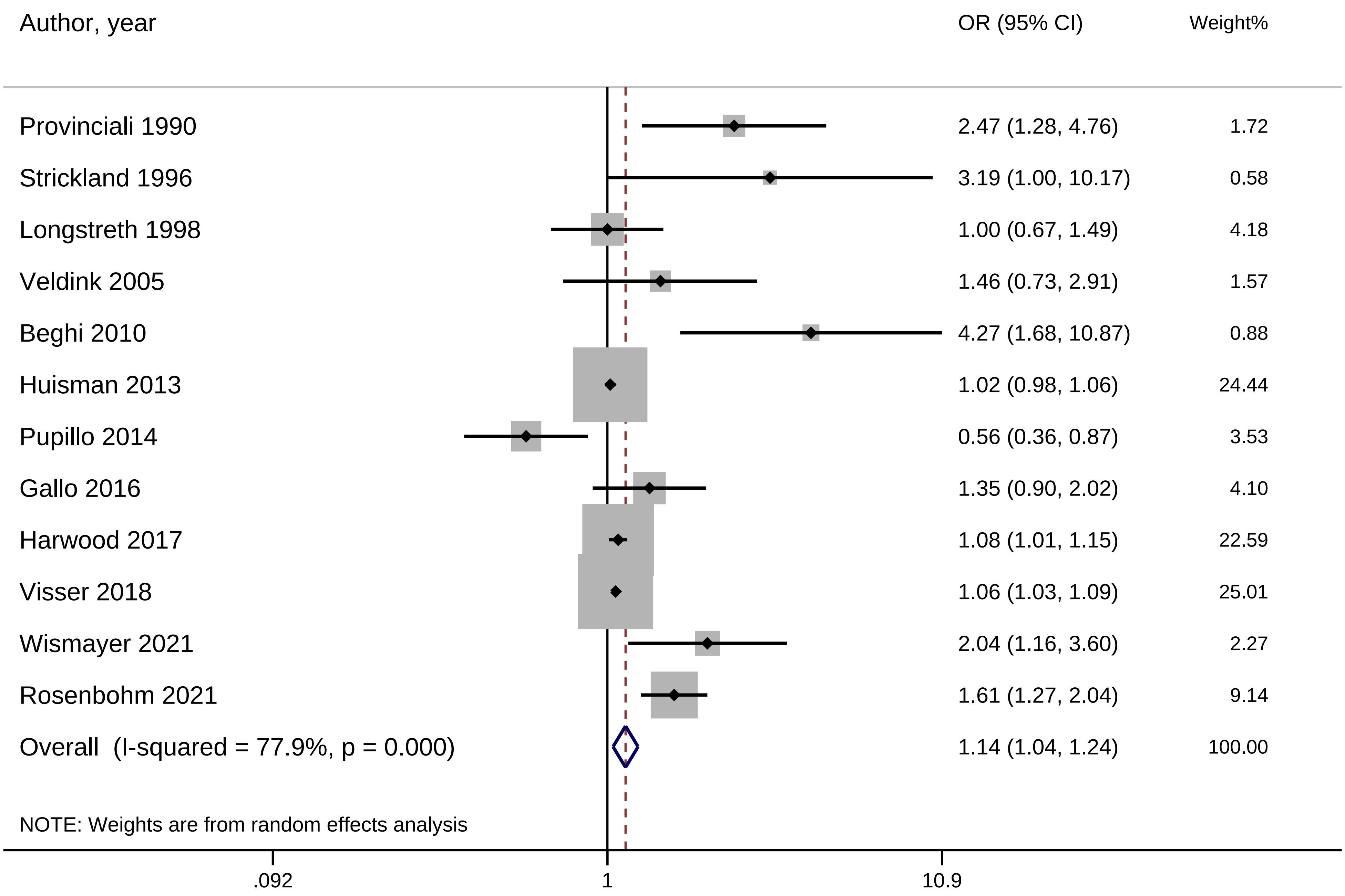

Figure S12. Funnel plot of meta-analysis of the association between occupational-related activity and ALS

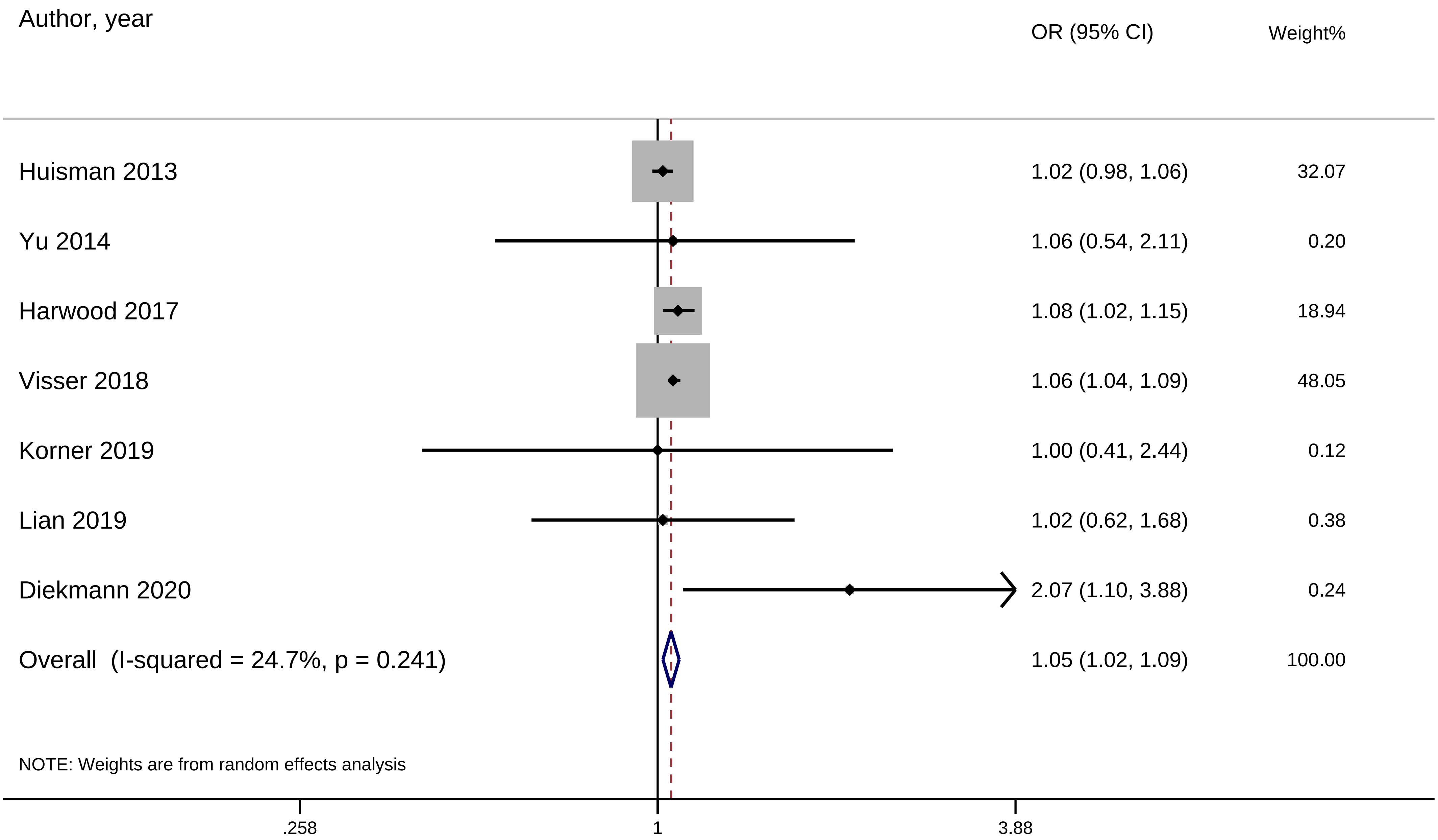

Figure S13. Random-effects meta-analysis of the association between unclassified physical activity and ALS

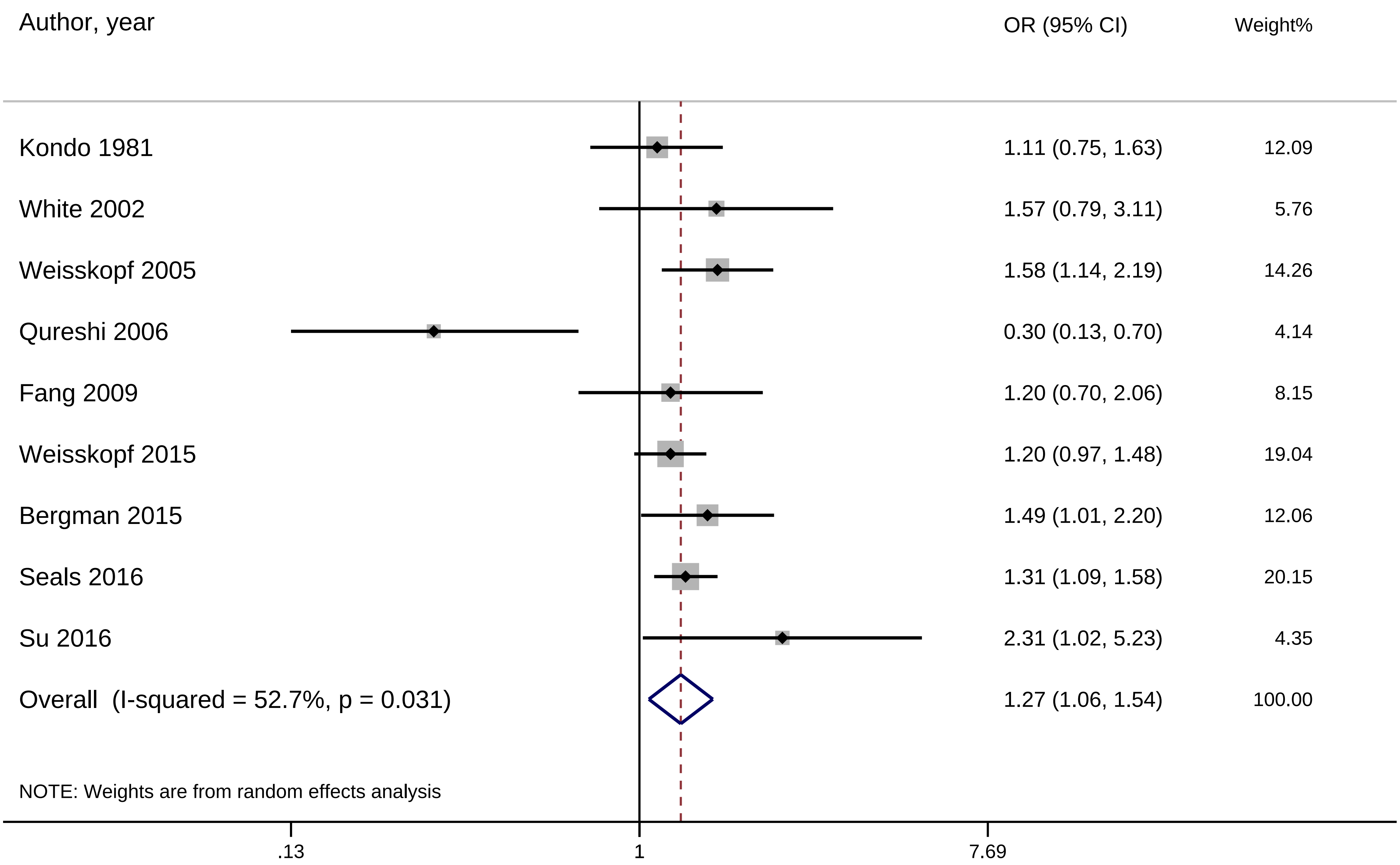

Figure S14. Random-effects meta-analysis of the association between military personnel and ALS

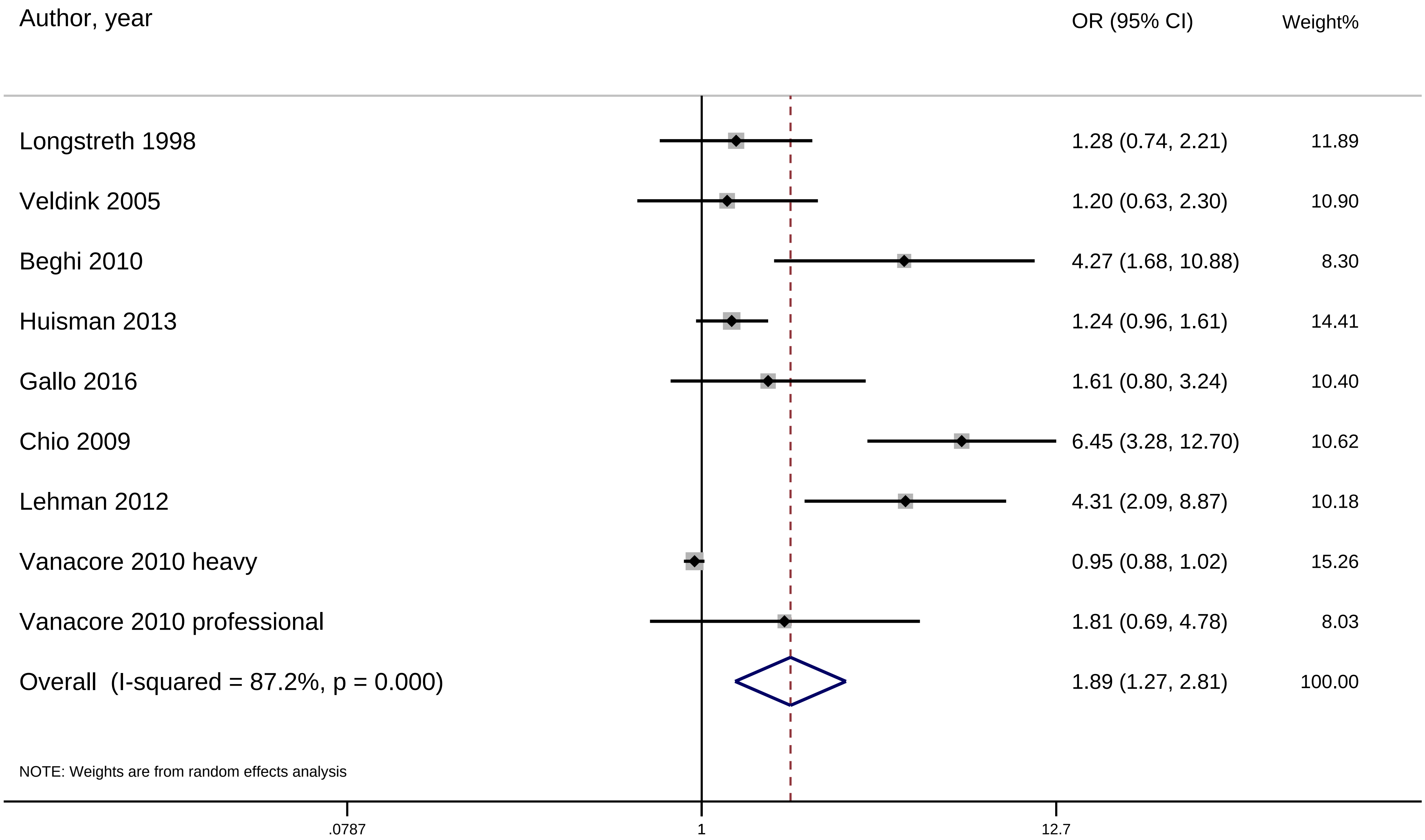

Figure S15. Random-effects meta-analysis of the association between heavy physical work and ALS

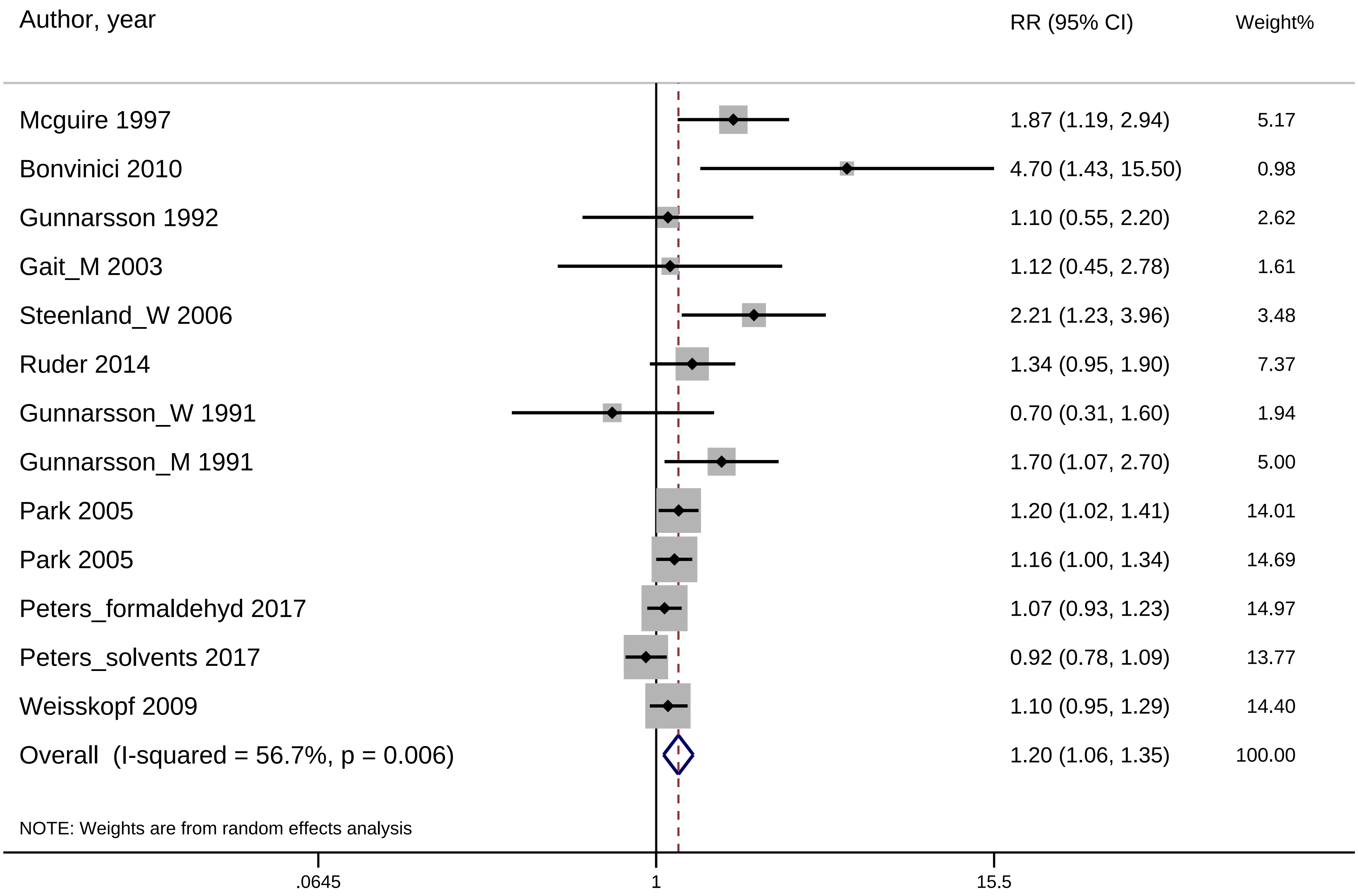

Figure S16. Random-effects meta-analysis of the association between chemicals and ALS

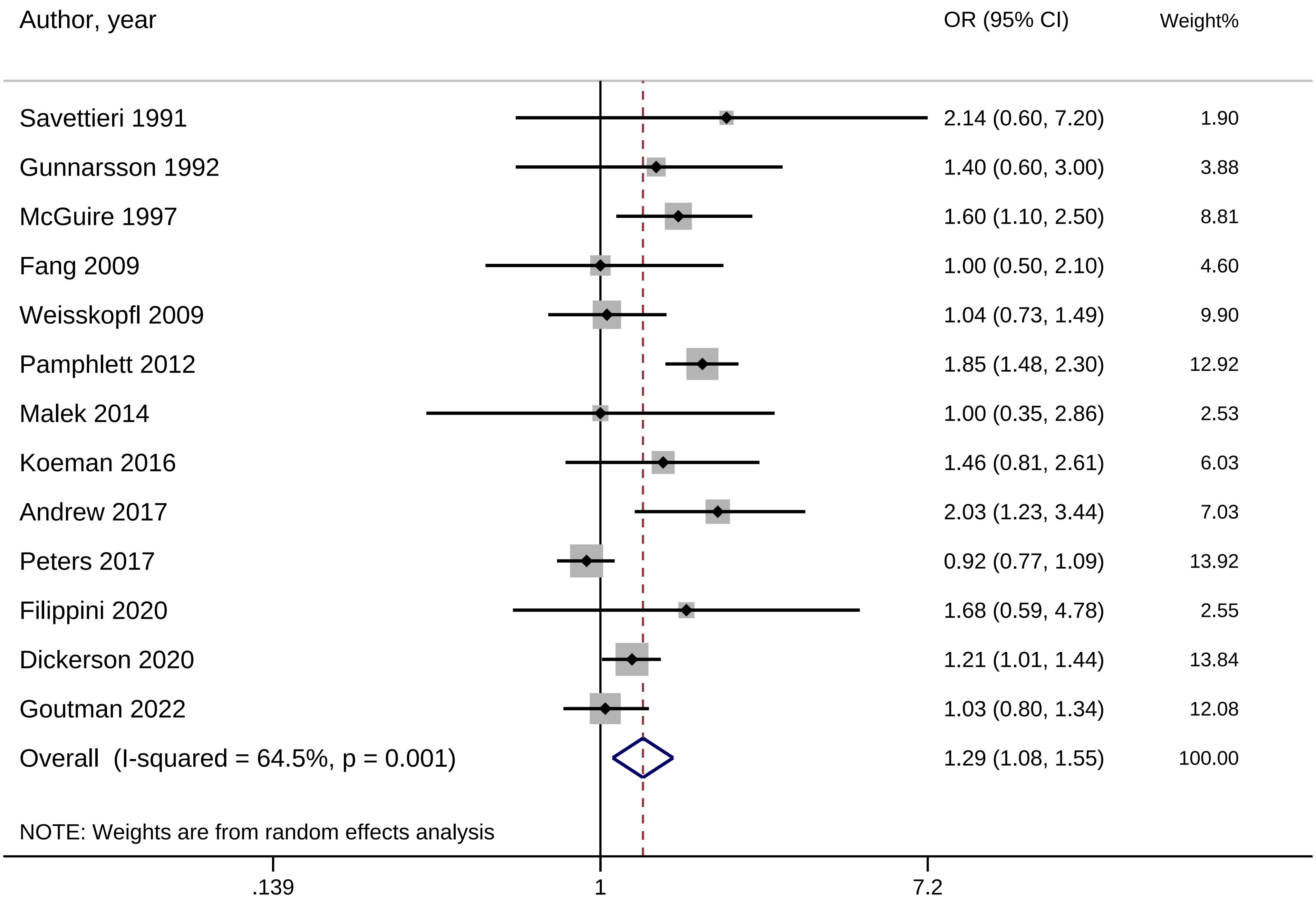

Figure S17. Random-effects meta-analysis of the association between environmental and occupational solvents and ALS

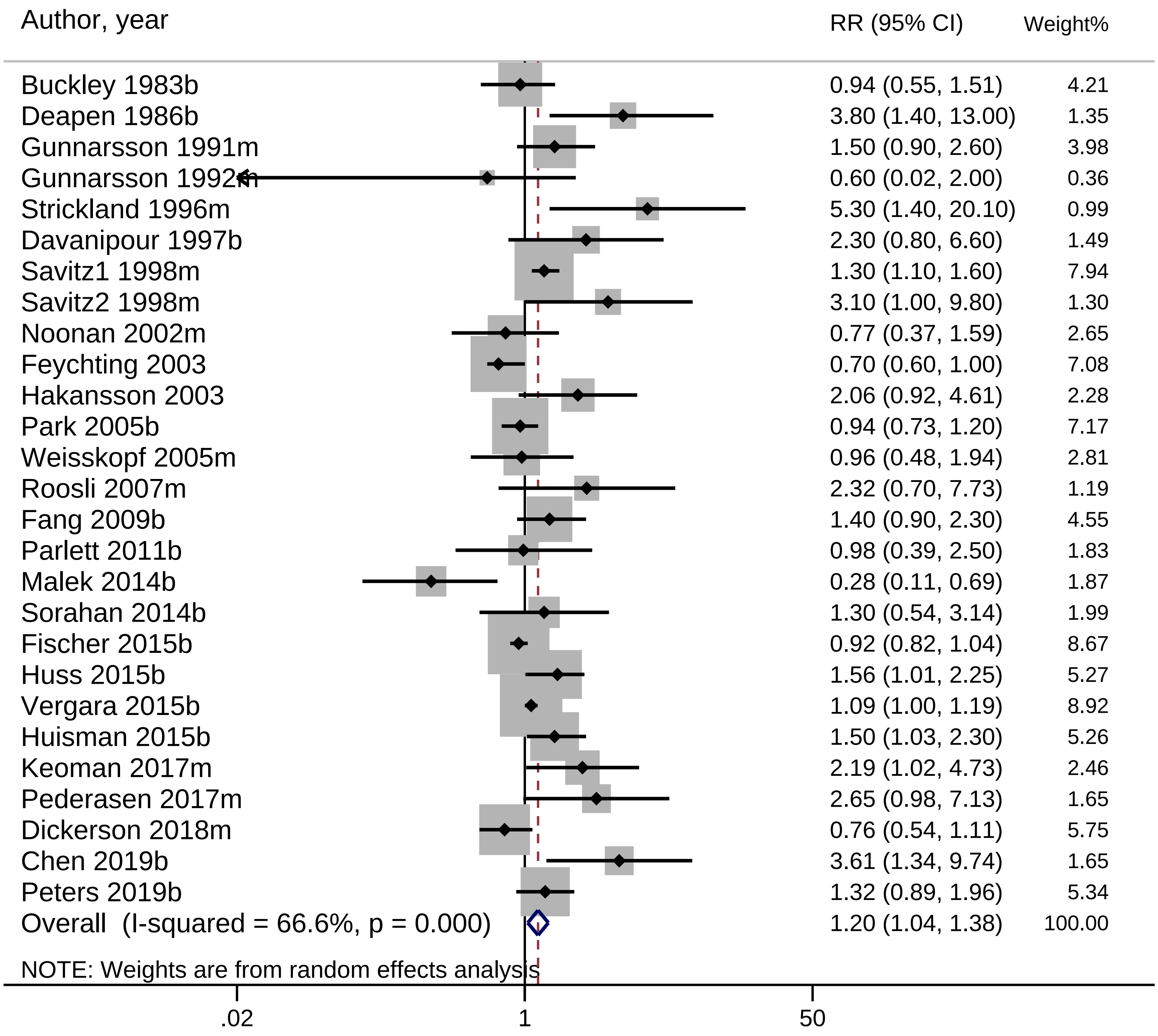

Figure S18. Random-effects meta-analysis of the association between extremely low frequency magnetic fields and ALS

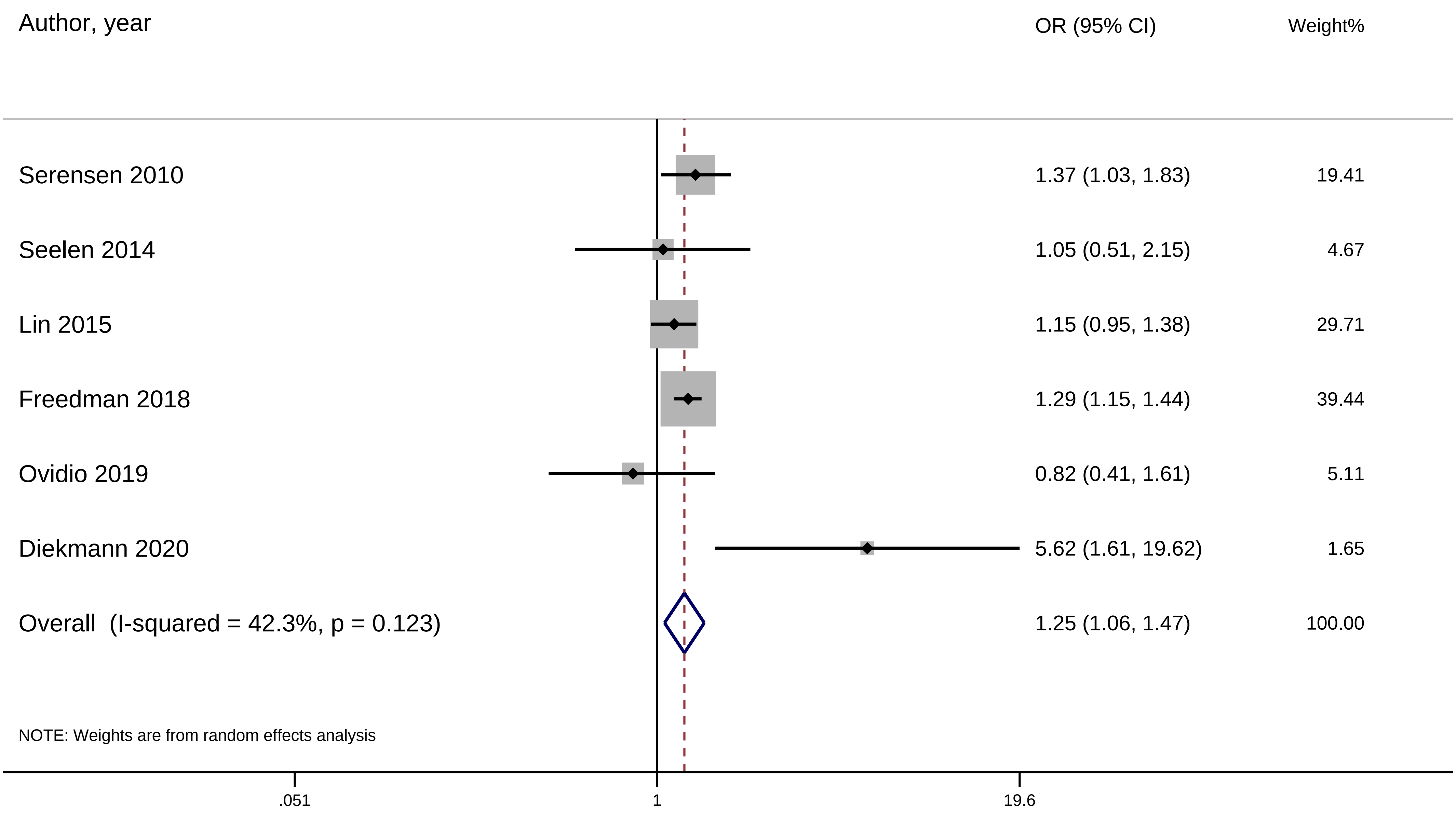

Figure S19. Funnel plot of meta-analysis of the association between stroke and ALS

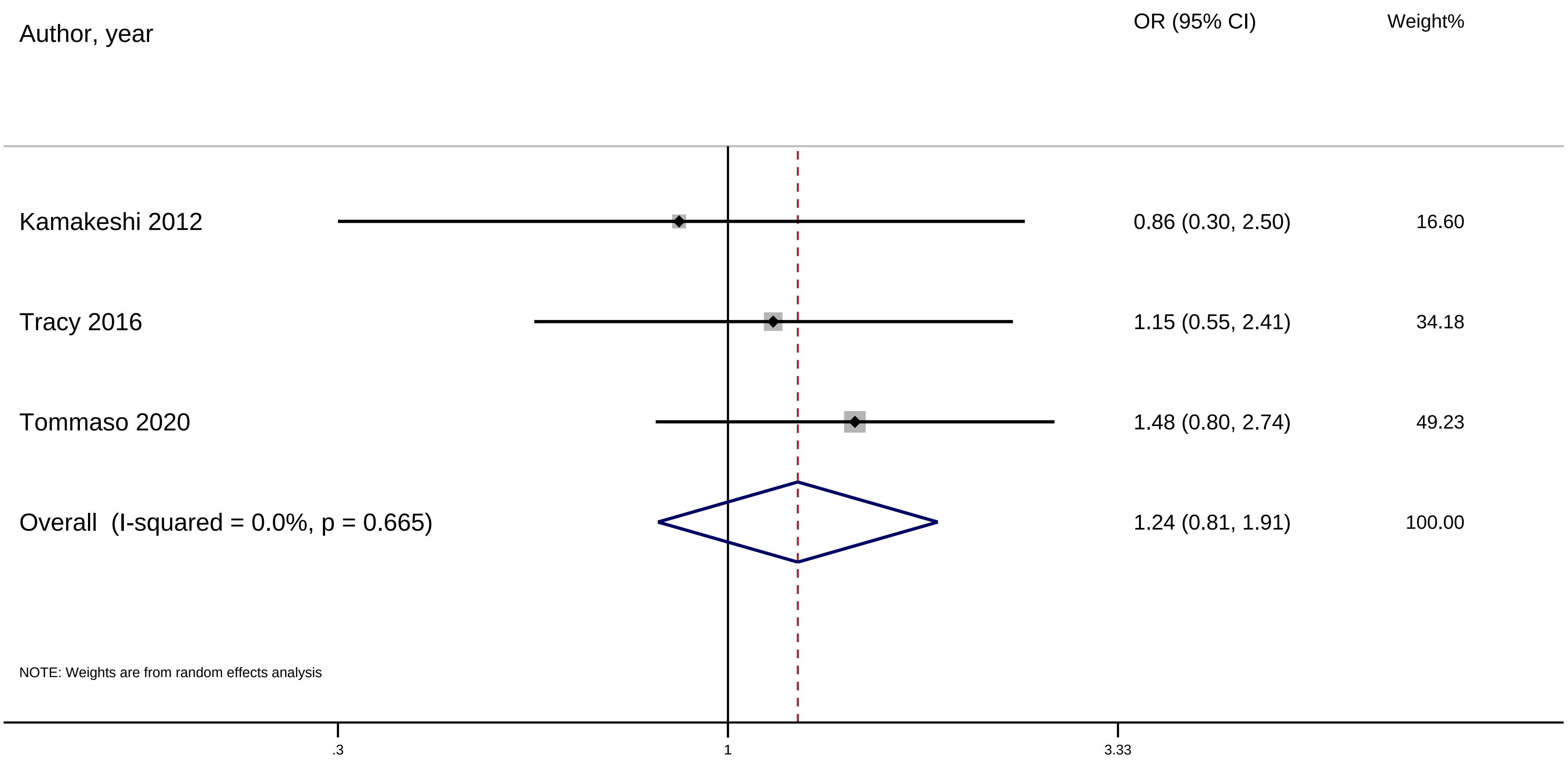

Figure S20. Random-effects meta-analysis of the association between occupation in industry and ALS

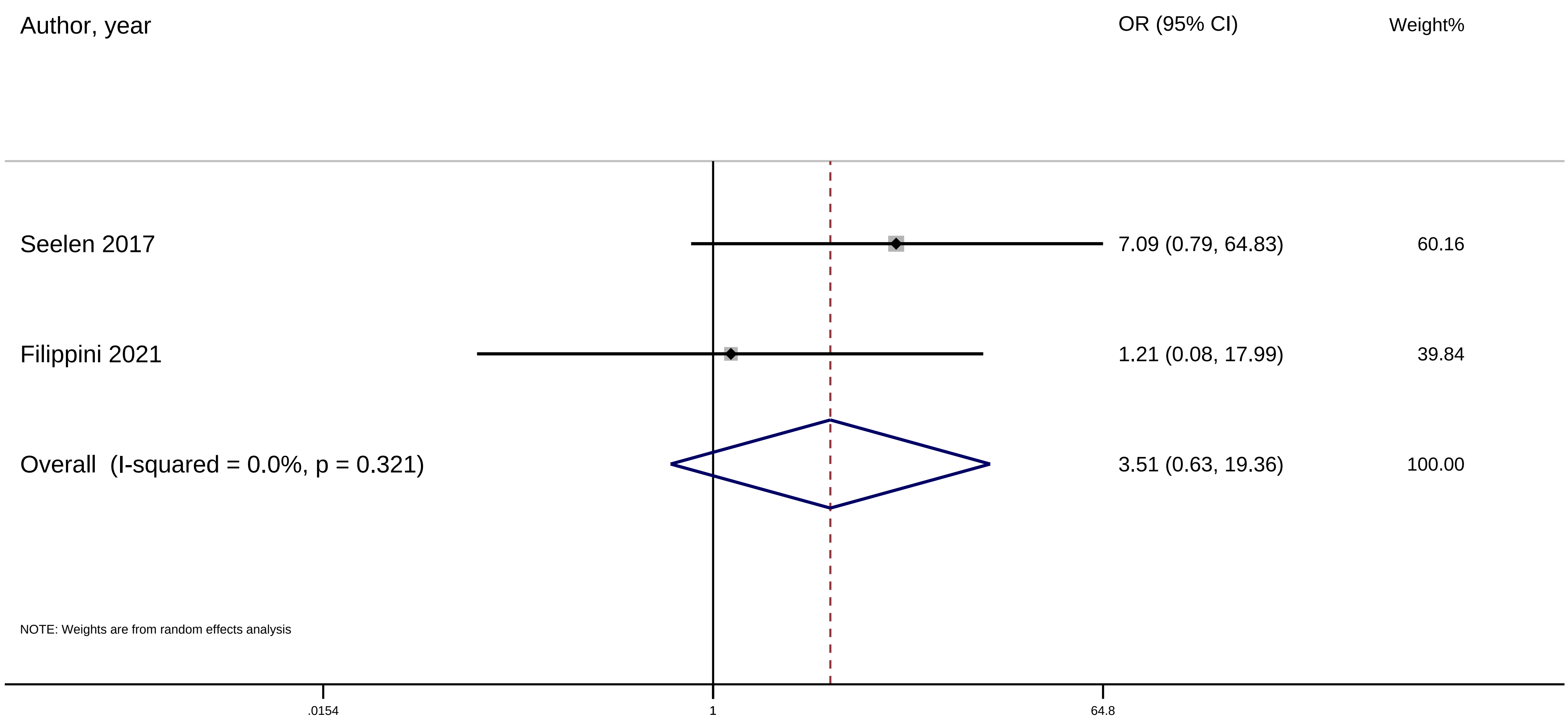

Figure S21. Random-effects meta-analysis of the association between annual PM10 exposure and ALS

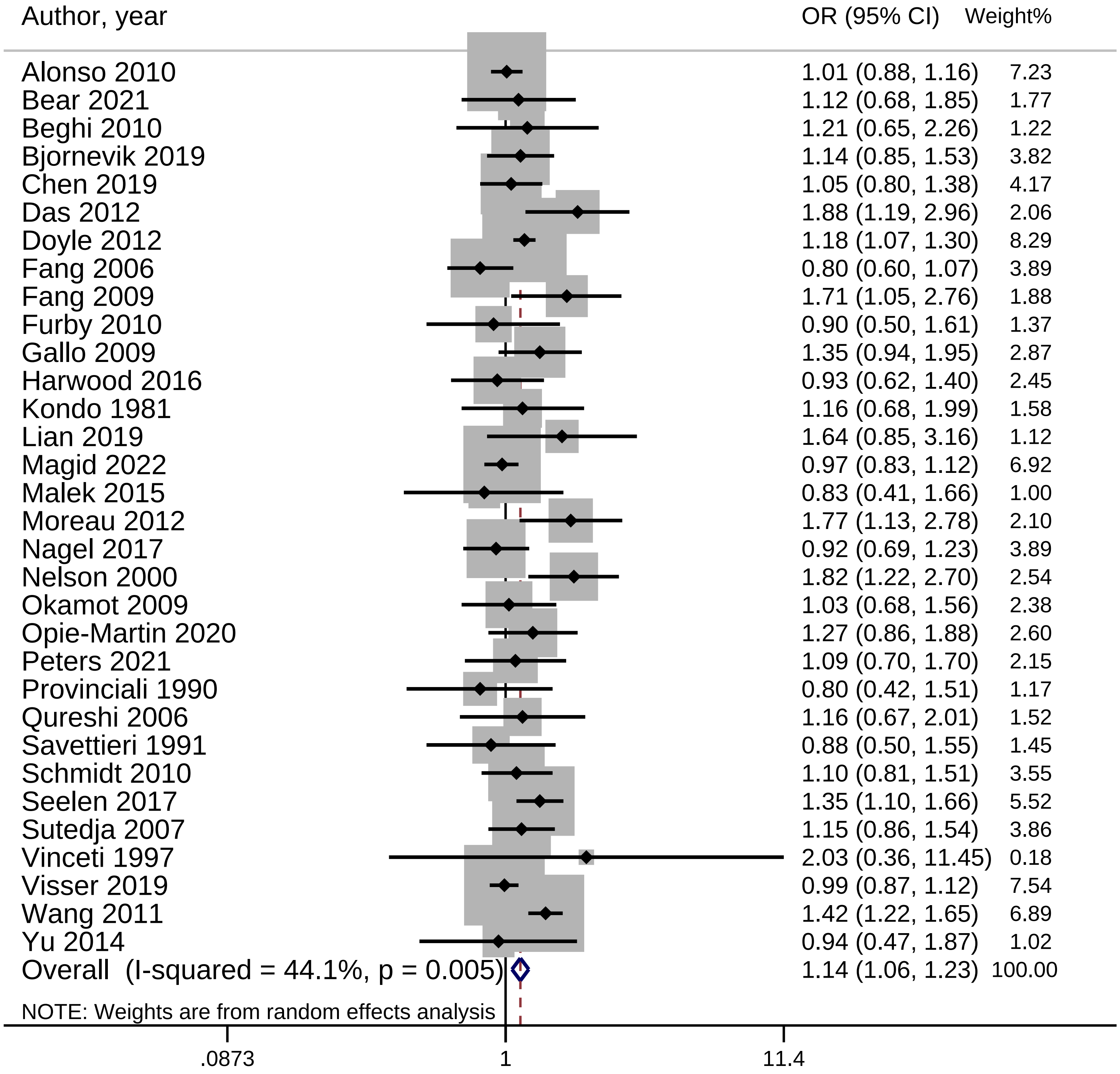

Figure S22. Random-effects meta-analysis of the association between smoking and ALS

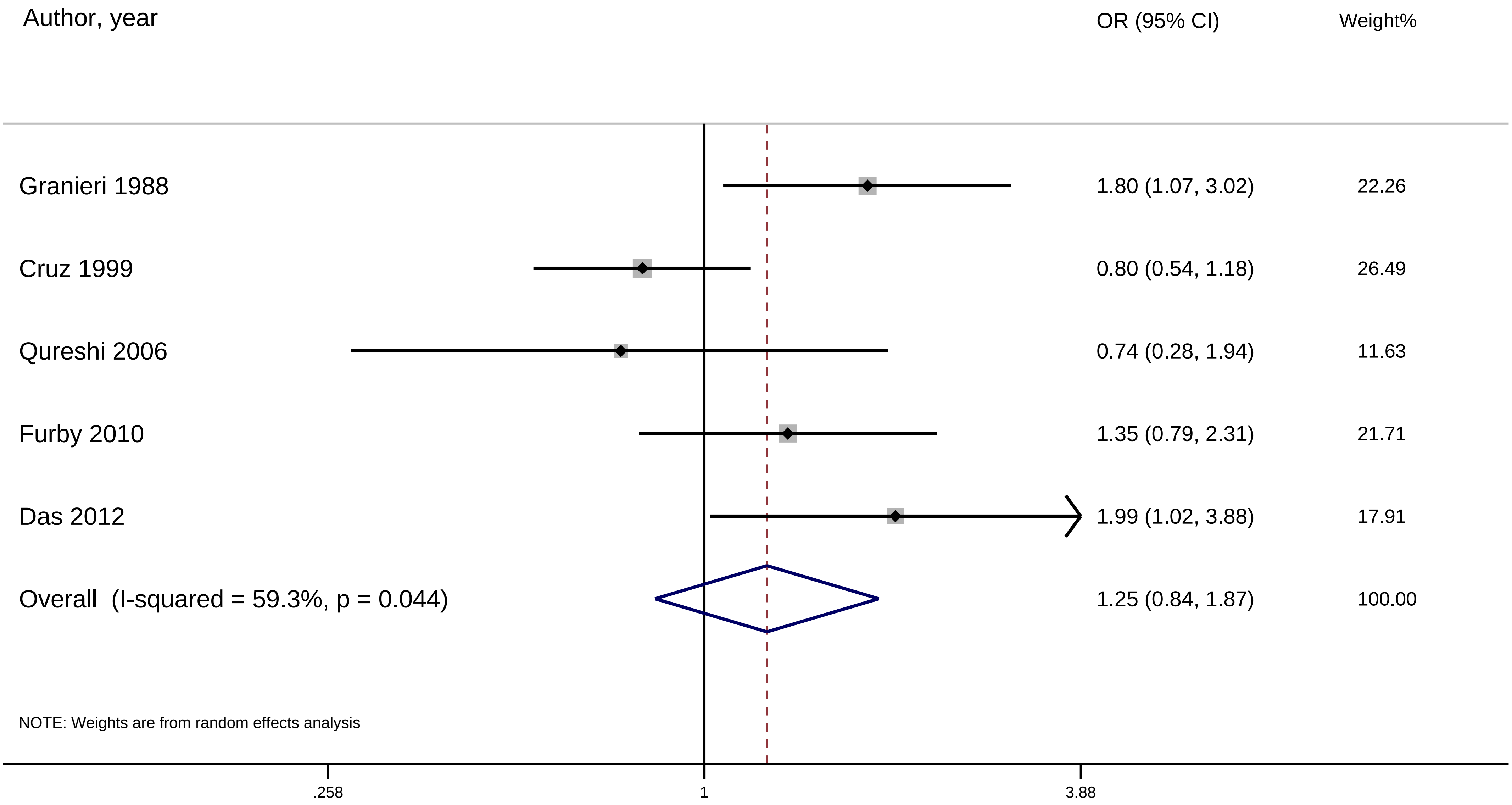

Figure S23. Random-effects meta-analysis of the association between rural residence and ALS

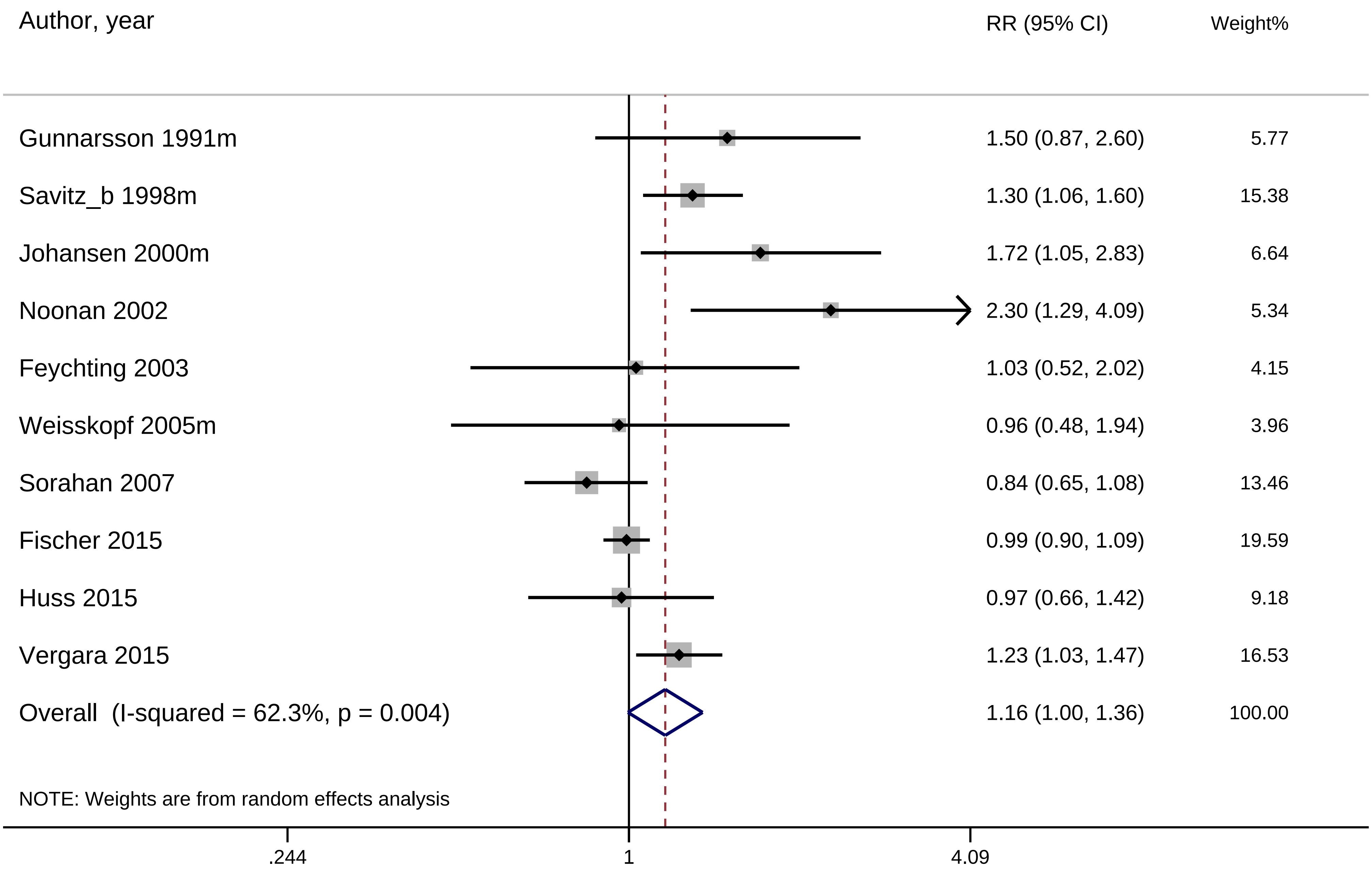

Figure S24. Random-effects meta-analysis of the association between work with electricity and ALS

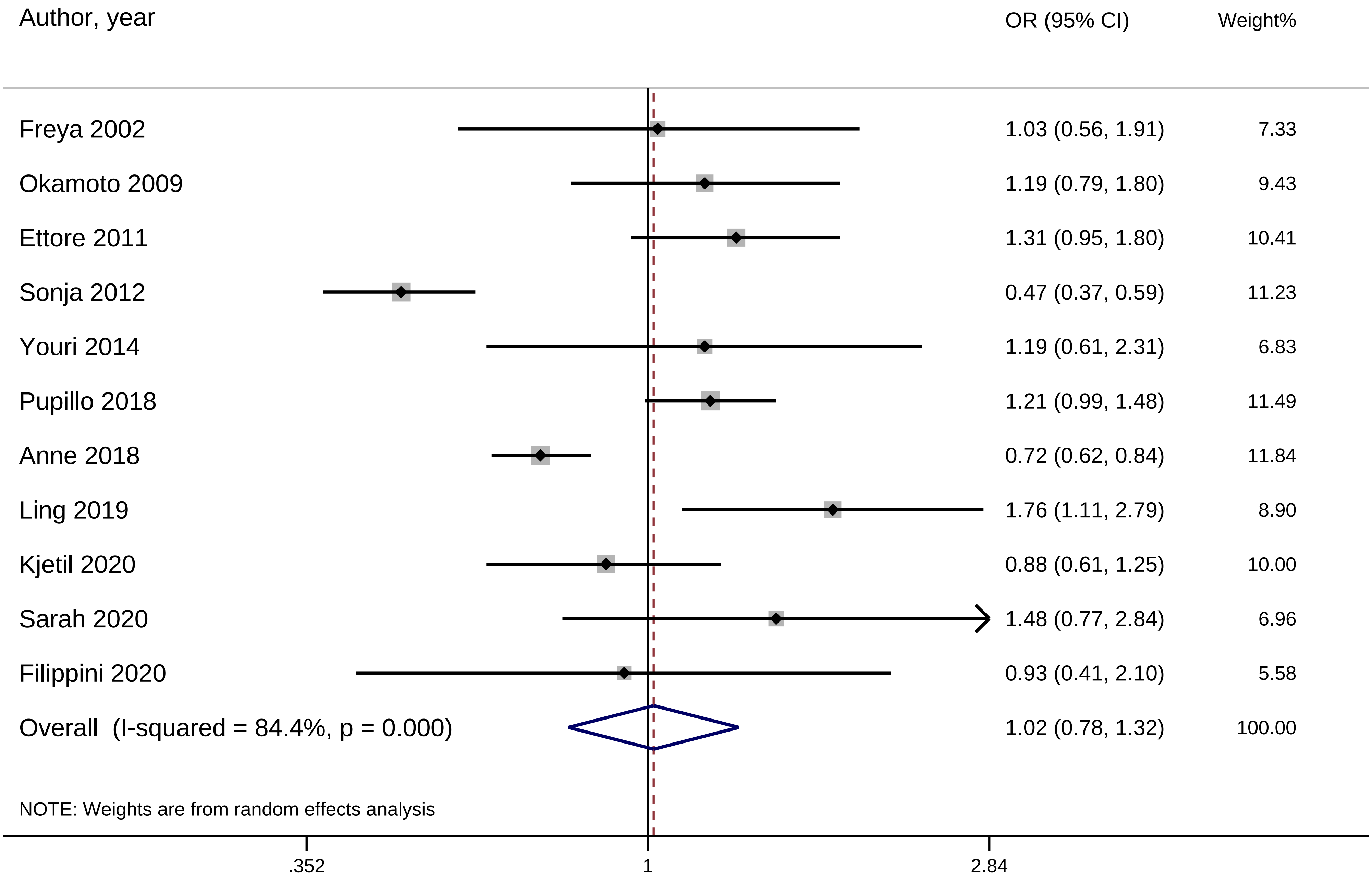

Figure S25. Random-effects meta-analysis of the association between alcohol consumption and ALS

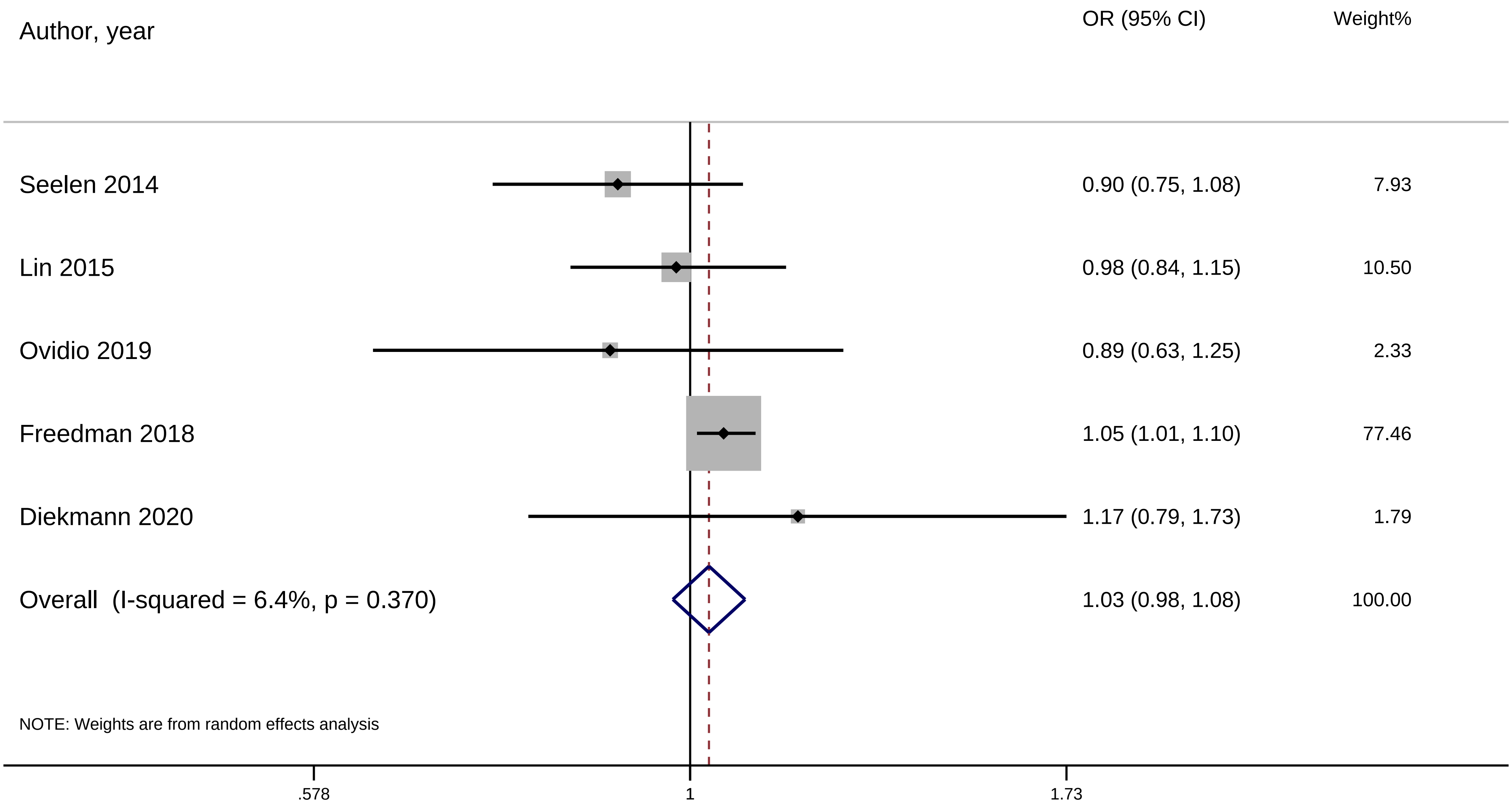

Figure S26. Random-effects meta-analysis of the association between hypertension and ALS

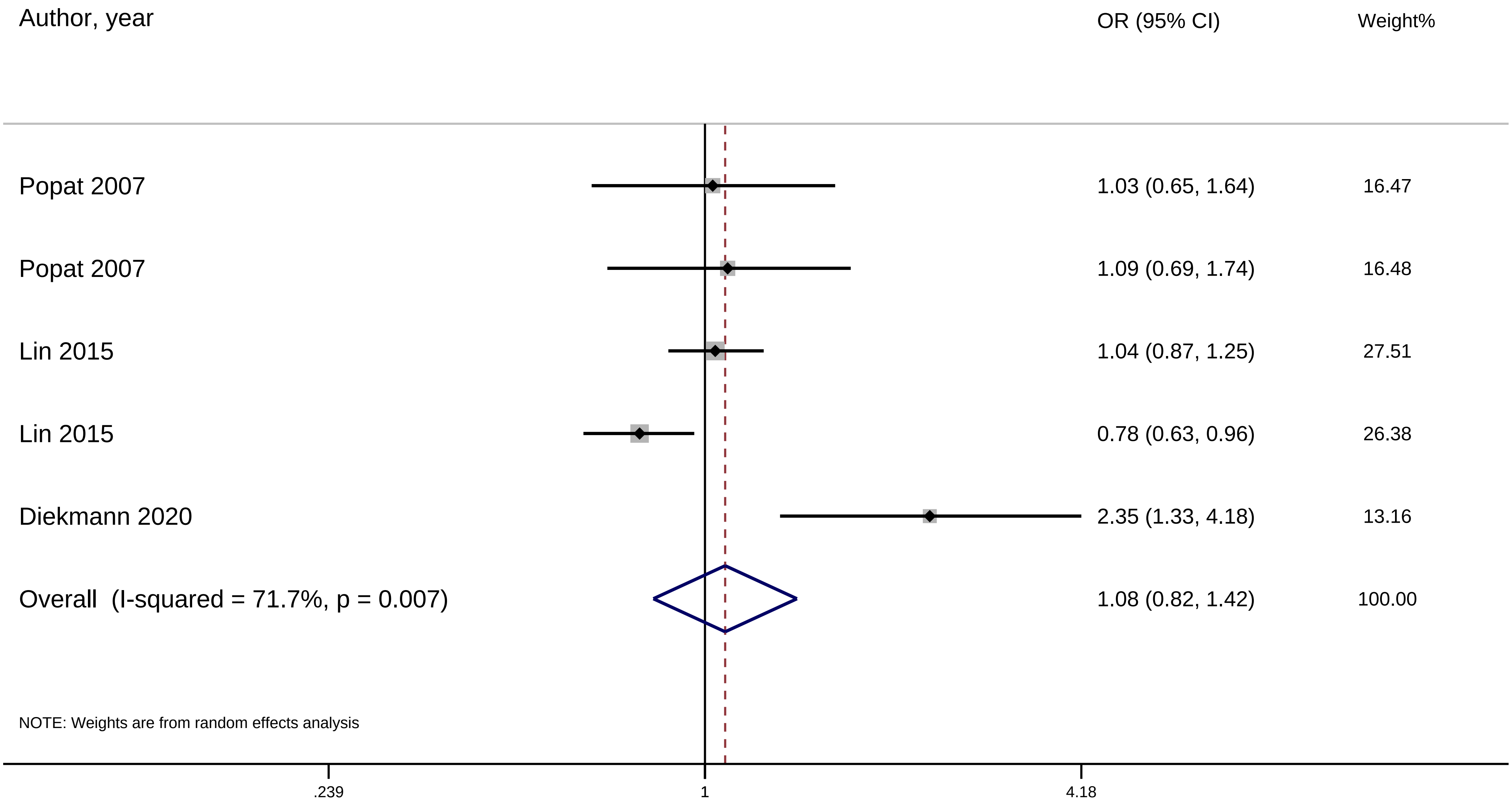

Figure S27. Random-effects meta-analysis of the association between NSAIDs and ALS

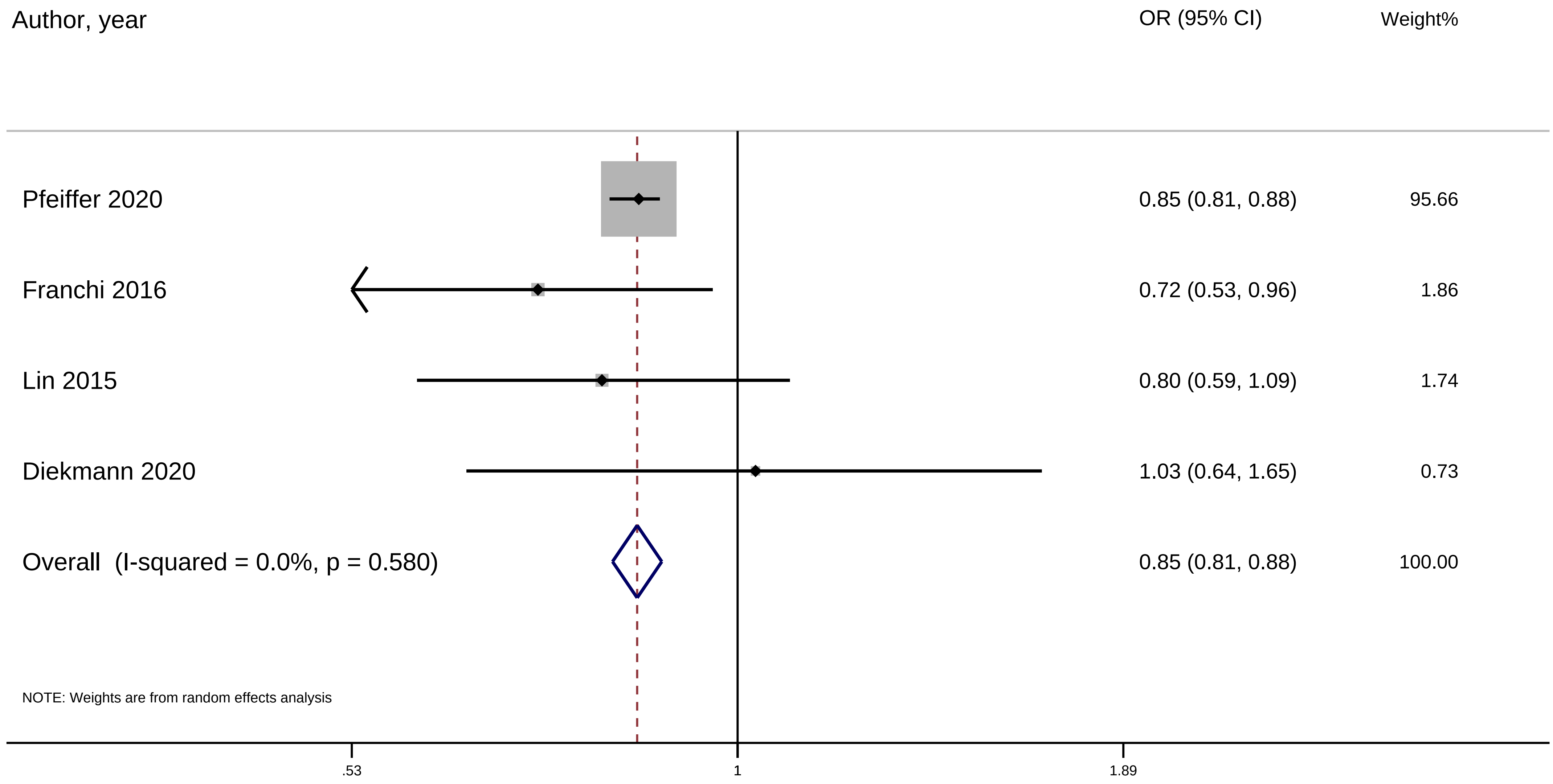

Figure S28. Random-effects meta-analysis of the association between anti-hypertensives and ALS

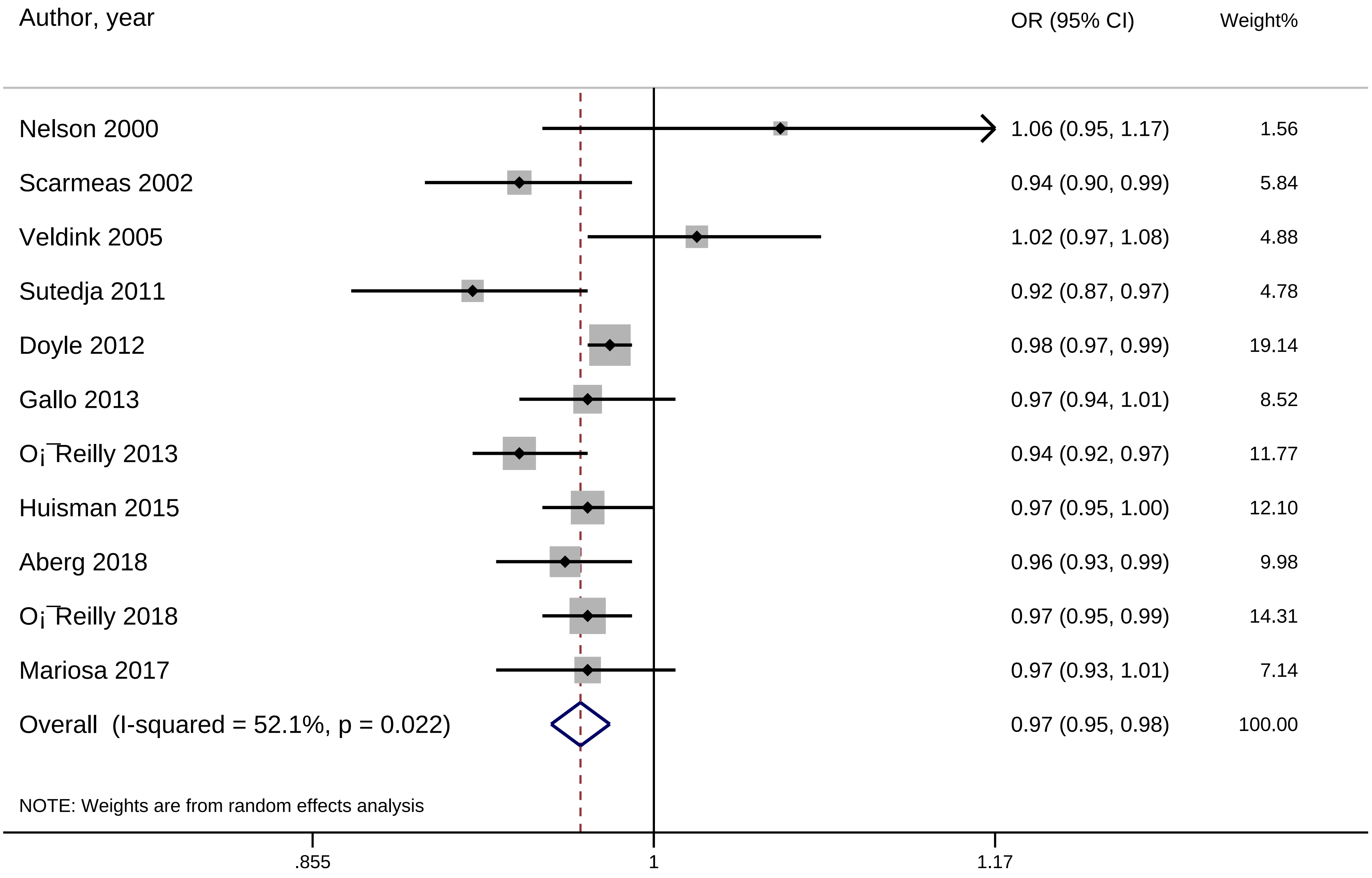

Figure S29. Random-effects meta-analysis of the association between premorbid body mass index and ALS

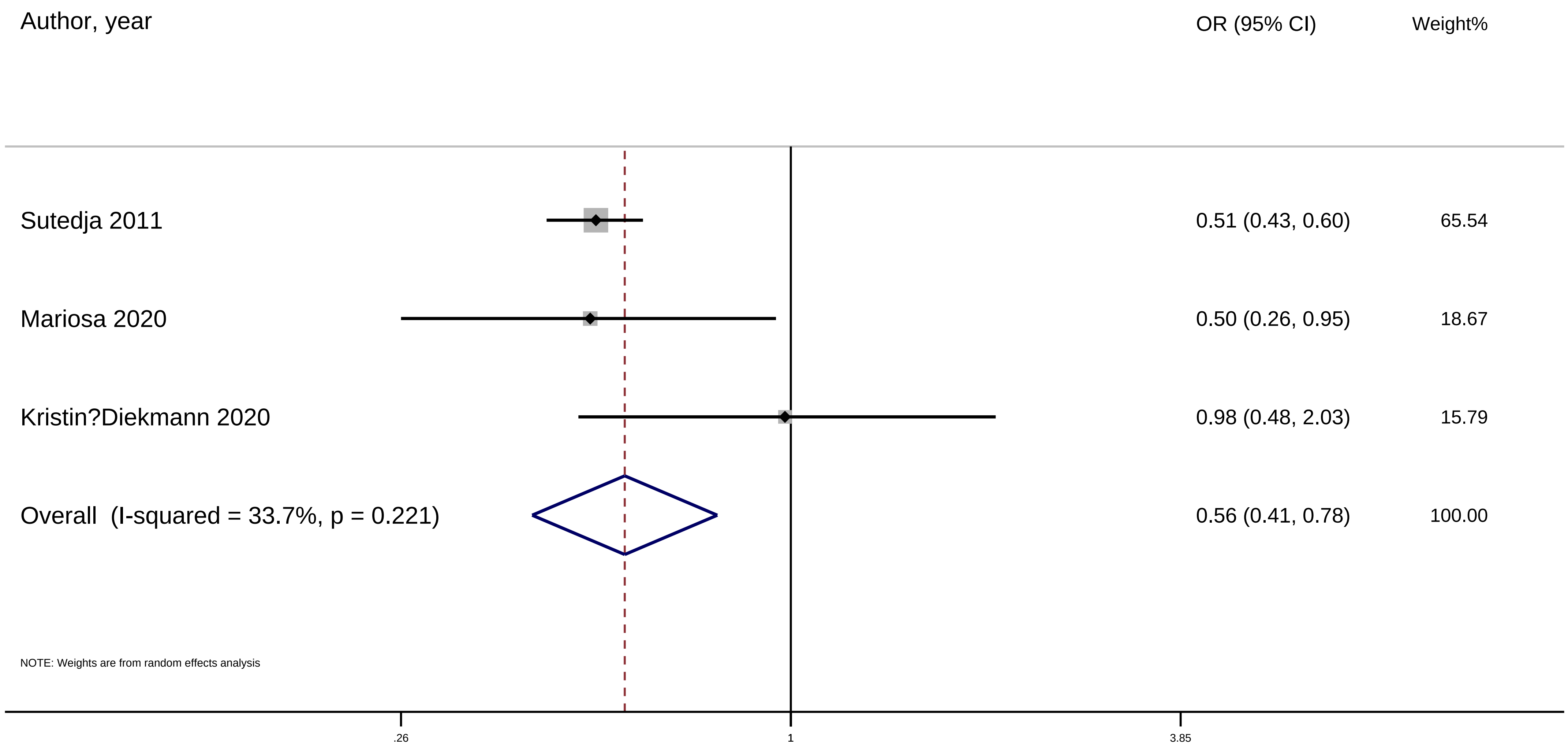

Figure S30. Random-effects meta-analysis of the association between anti-diabetes and ALS

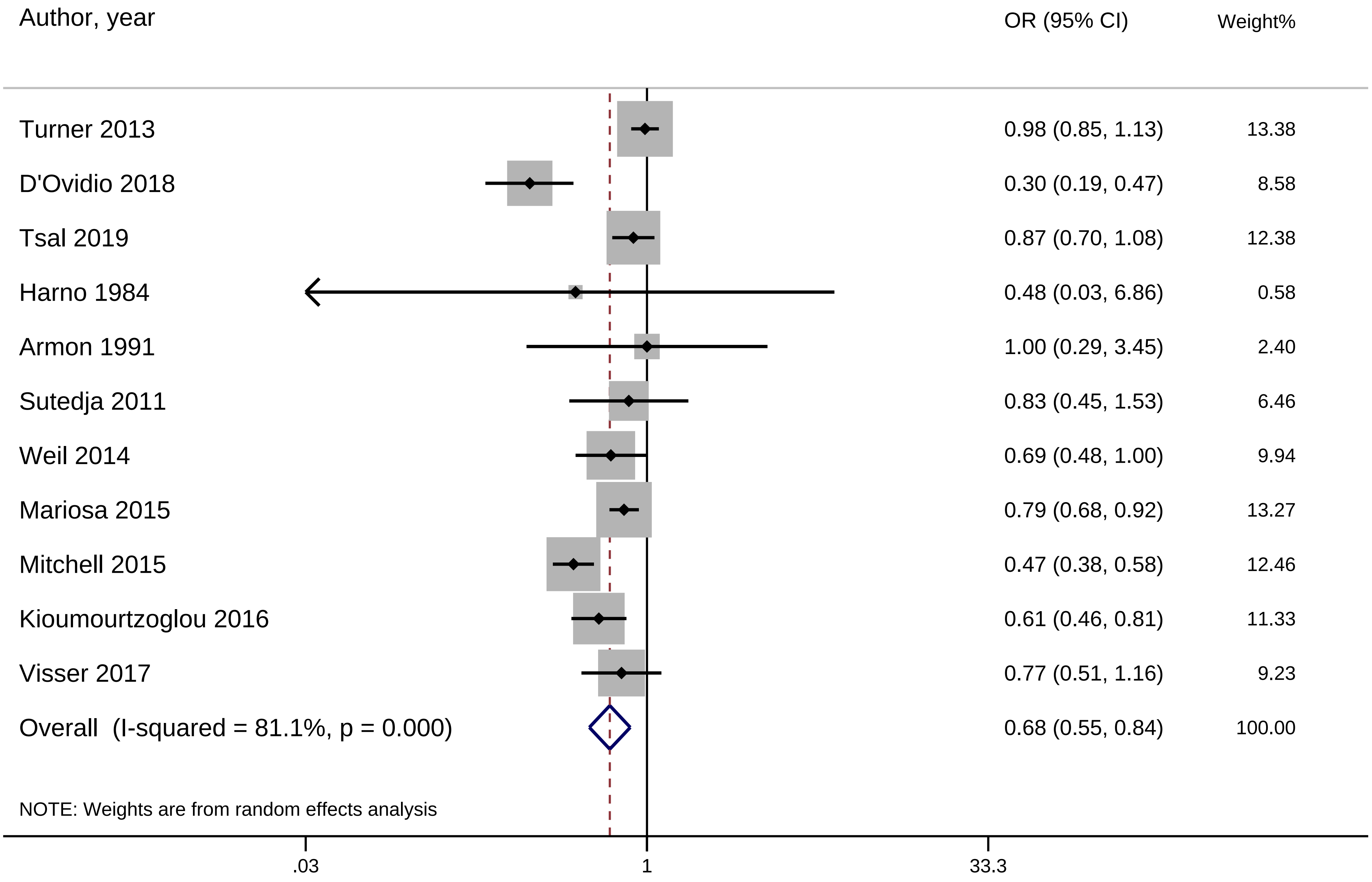

Figure S31. Random-effects meta-analysis of the association between diabetes mellitus and ALS

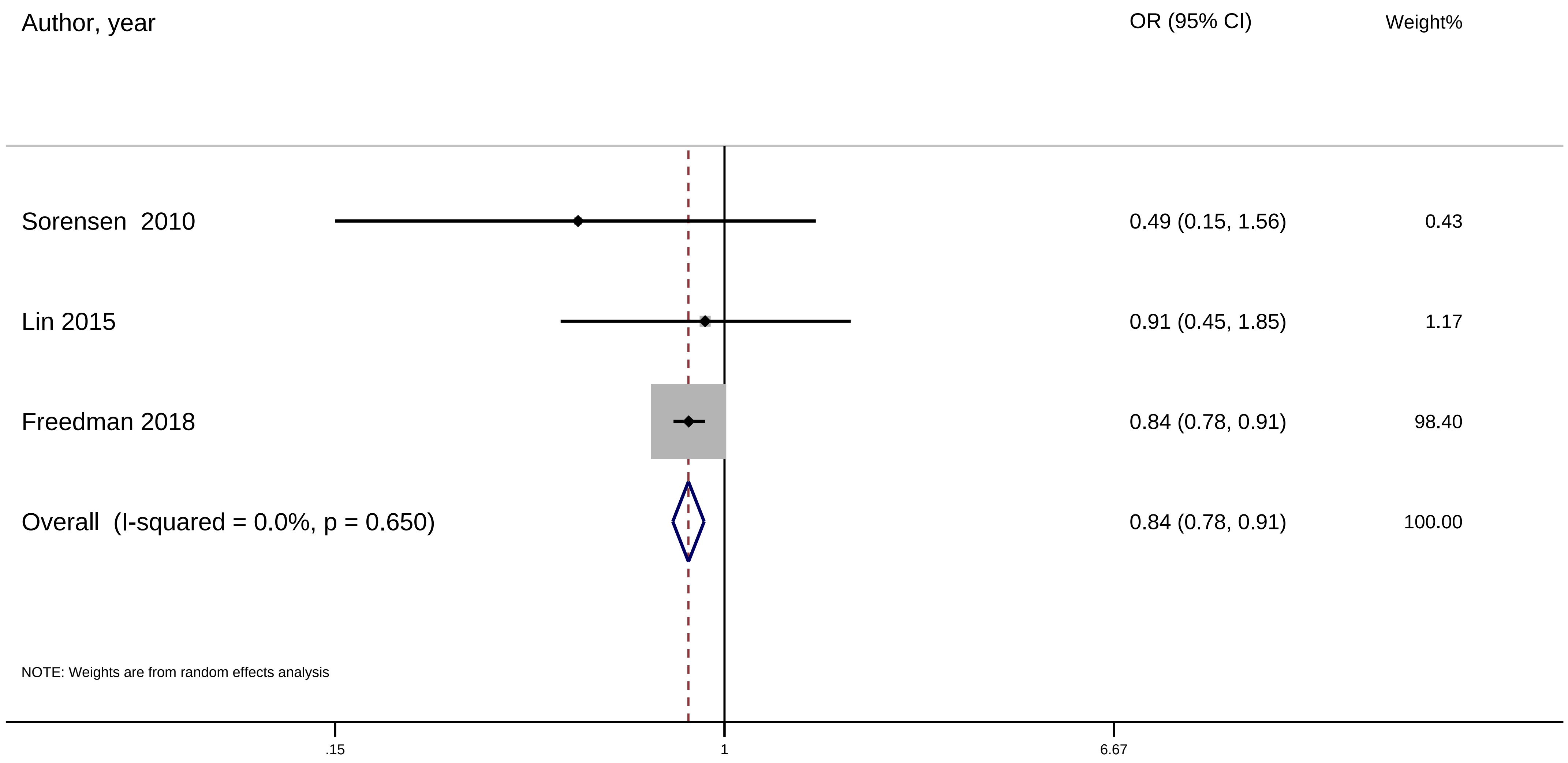

Figure S32. Random-effects meta-analysis of the association between kidney diseases and ALS

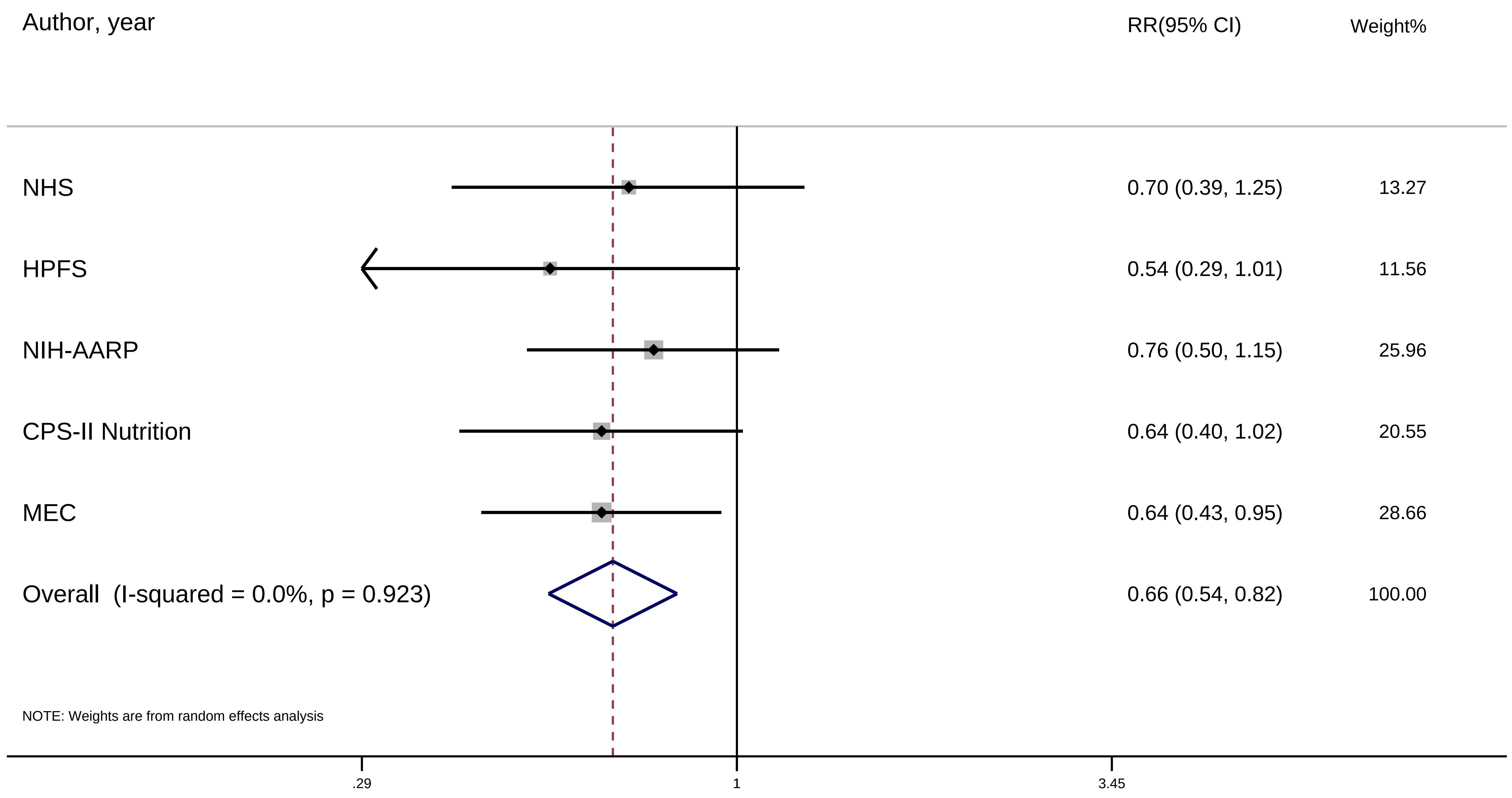

Figure S33. Random-effects meta-analysis of the association between ω-3 polyunsaturated fatty acid intake and ALS

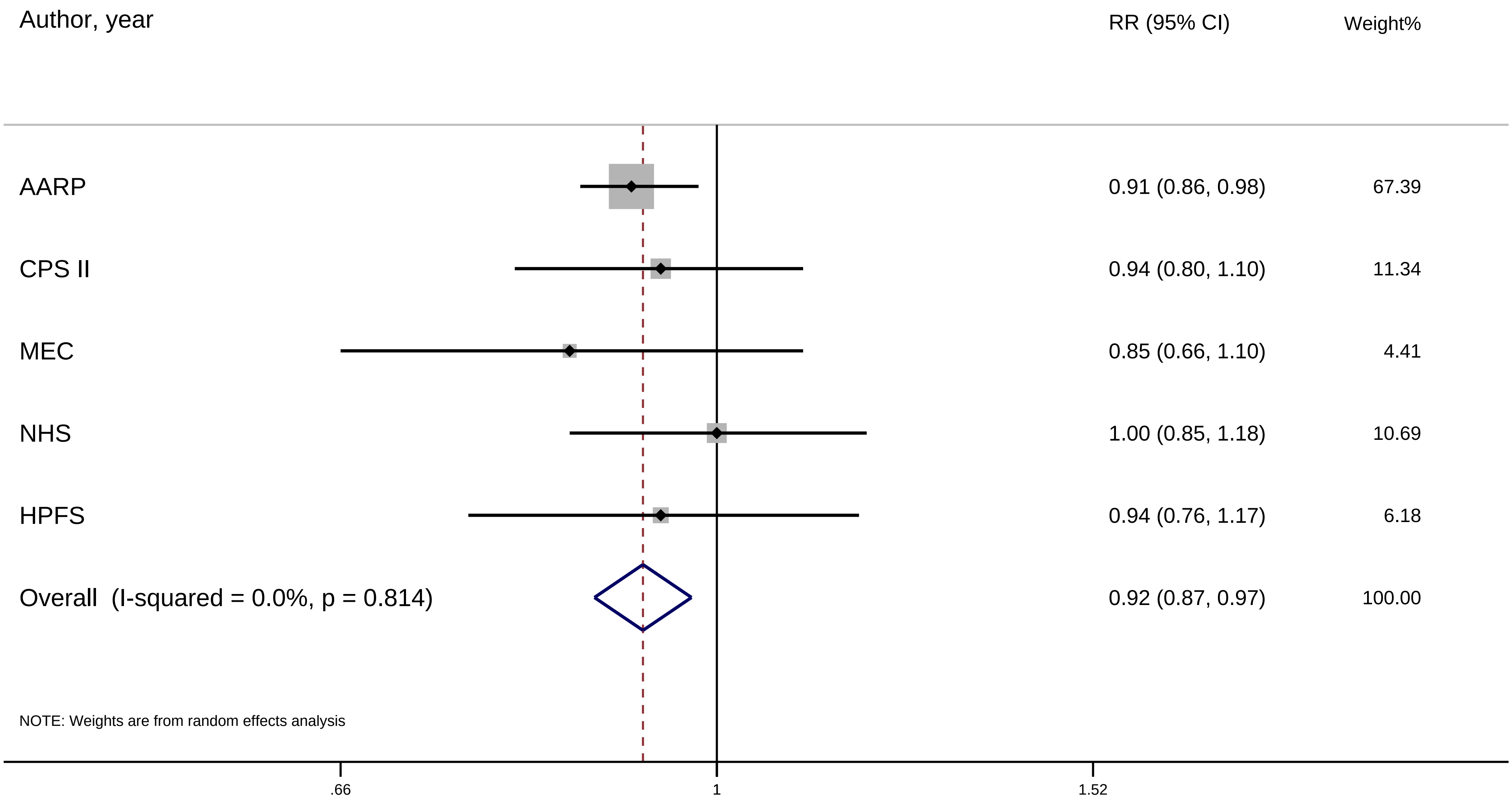

Figure S34. Random-effects meta-analysis of the association between carotenoids intake and ALS

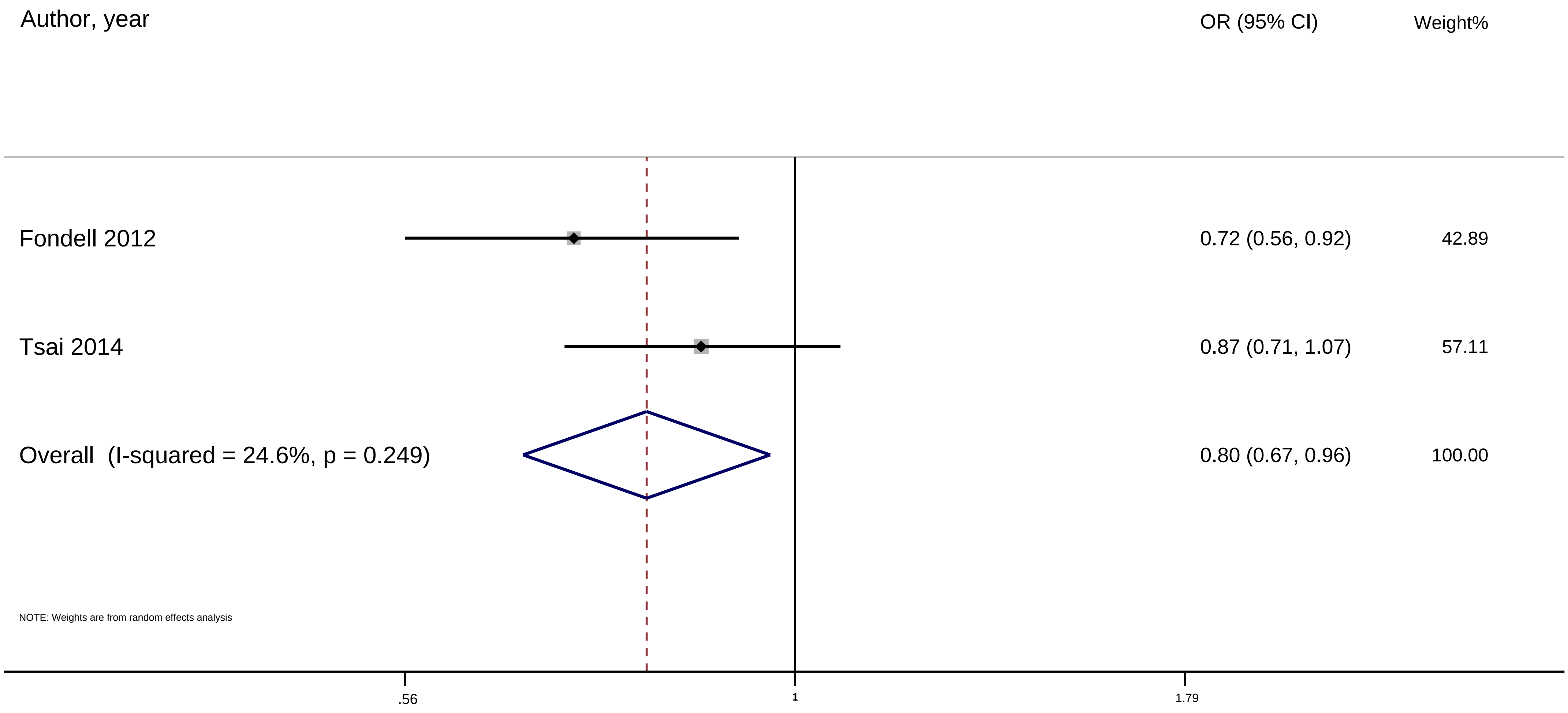

Figure S35. Random-effects meta-analysis of the association between acetaminophen and ALS

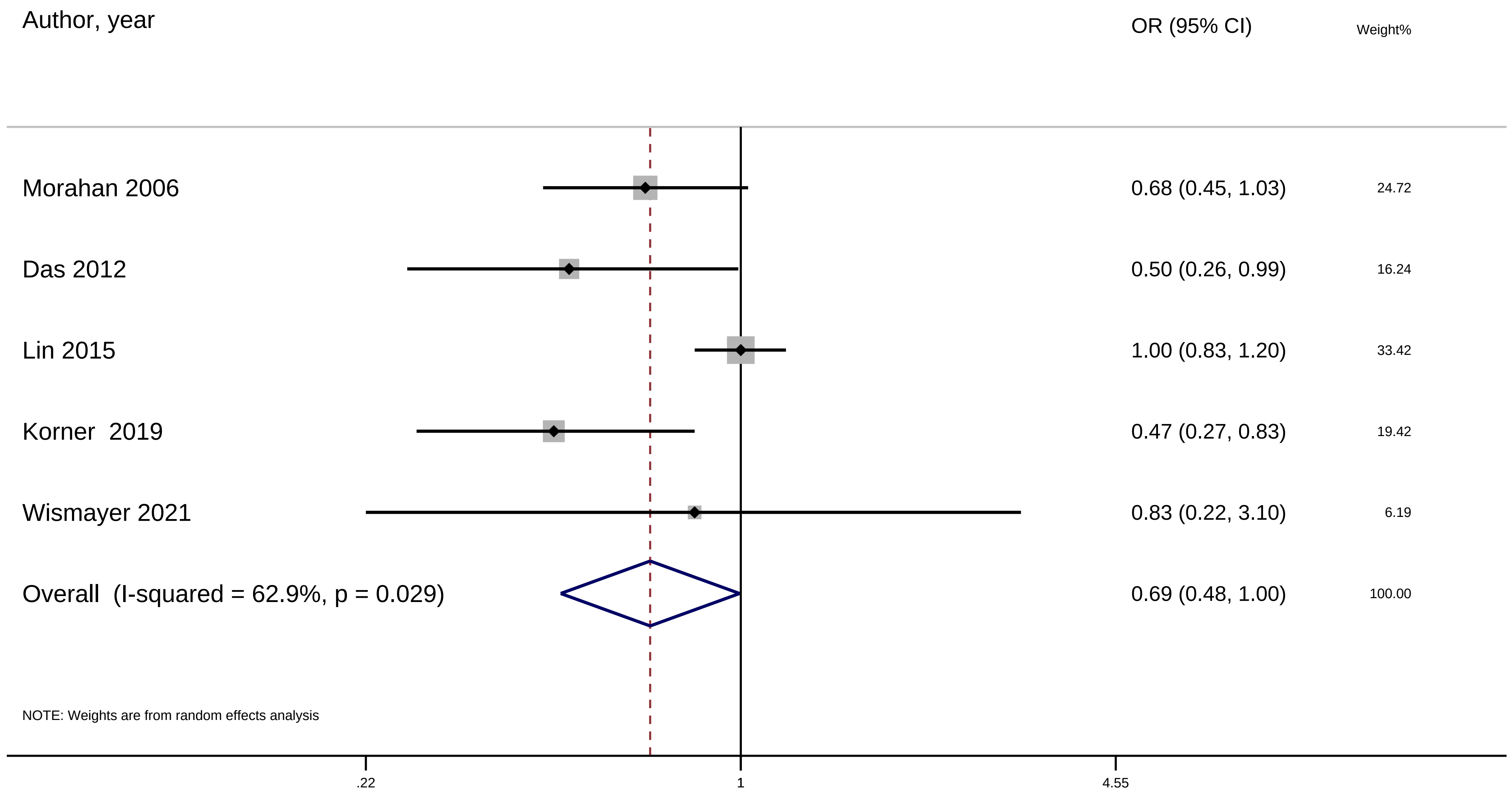

Figure S36. Random-effects meta-analysis of the association between living in urban and ALS

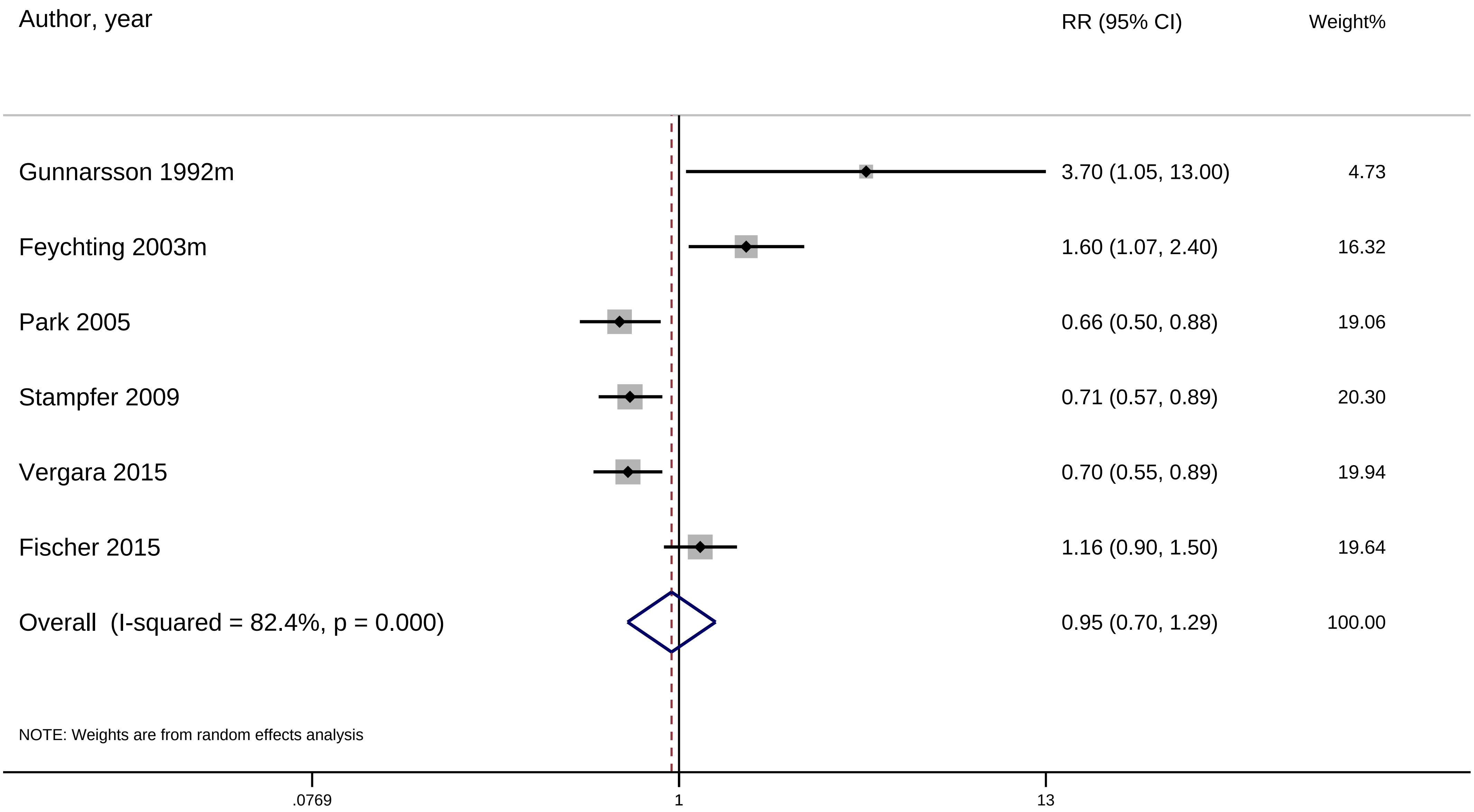

Figure S37. Random-effects meta-analysis of the association between welding and ALS

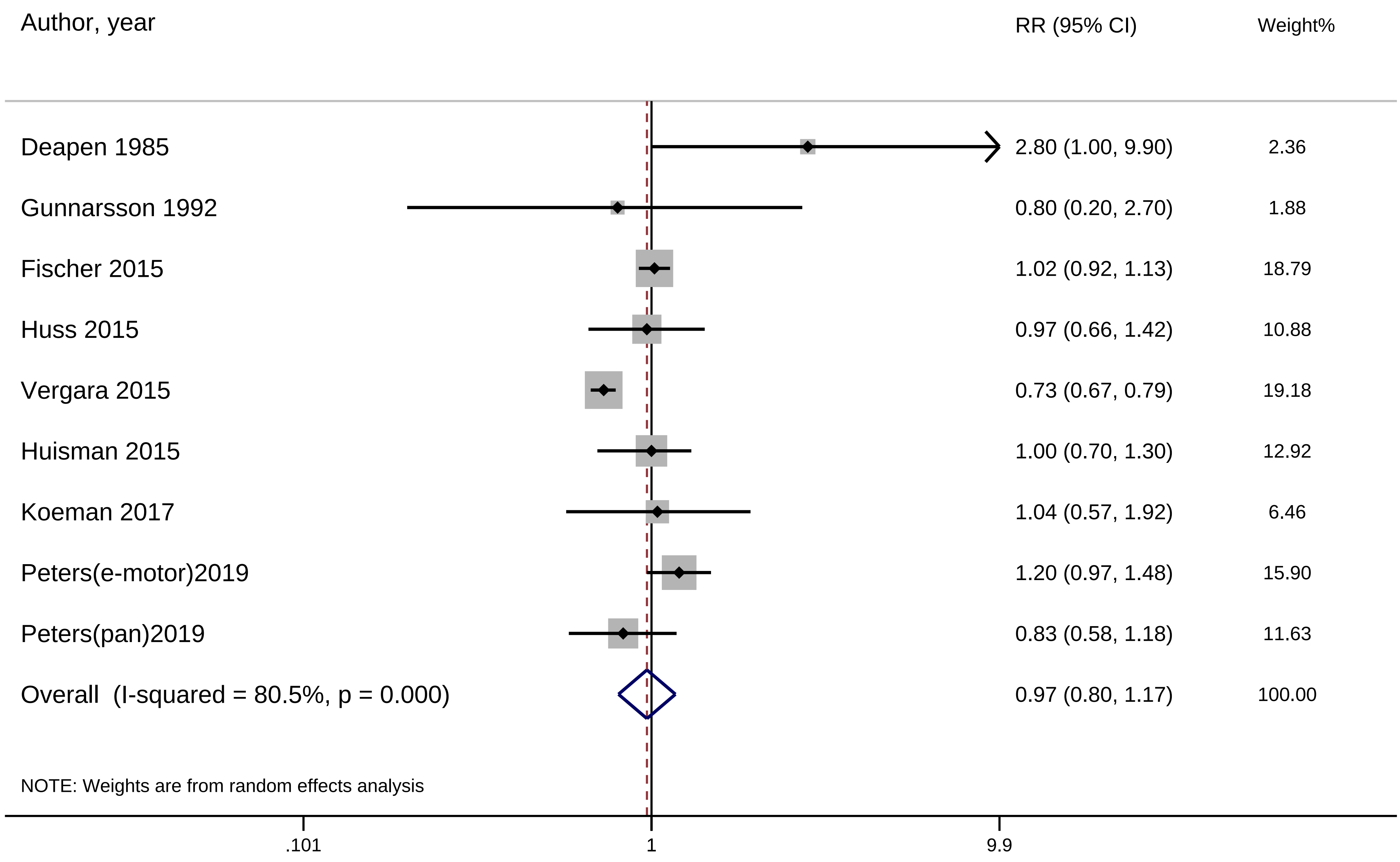

Figure S38. Random-effects meta-analysis of the association between electric shocks and ALS

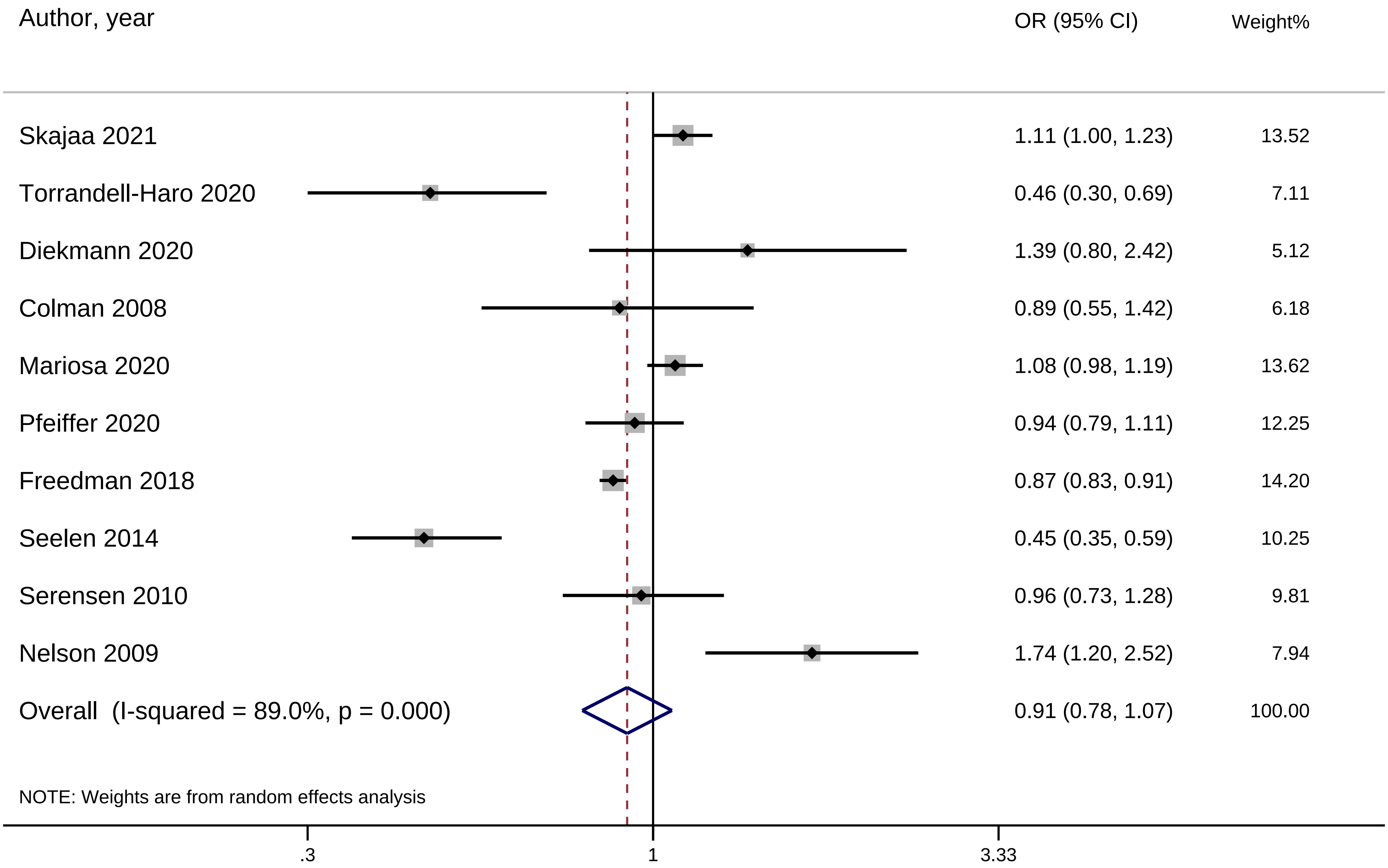

Figure S39. Random-effects meta-analysis of the association between statin and ALS

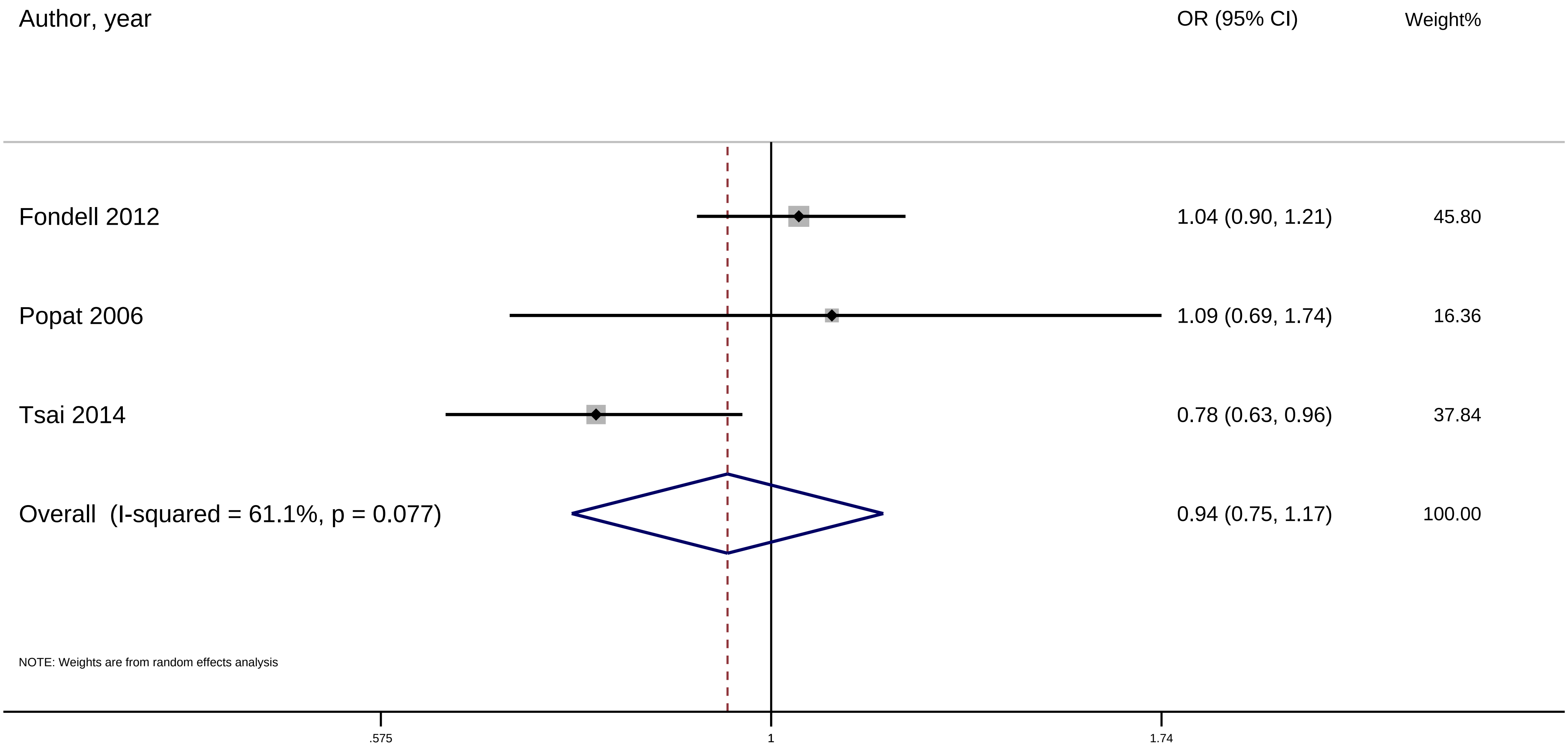

Figure S40. Random-effects meta-analysis of the association between aspirin and ALS

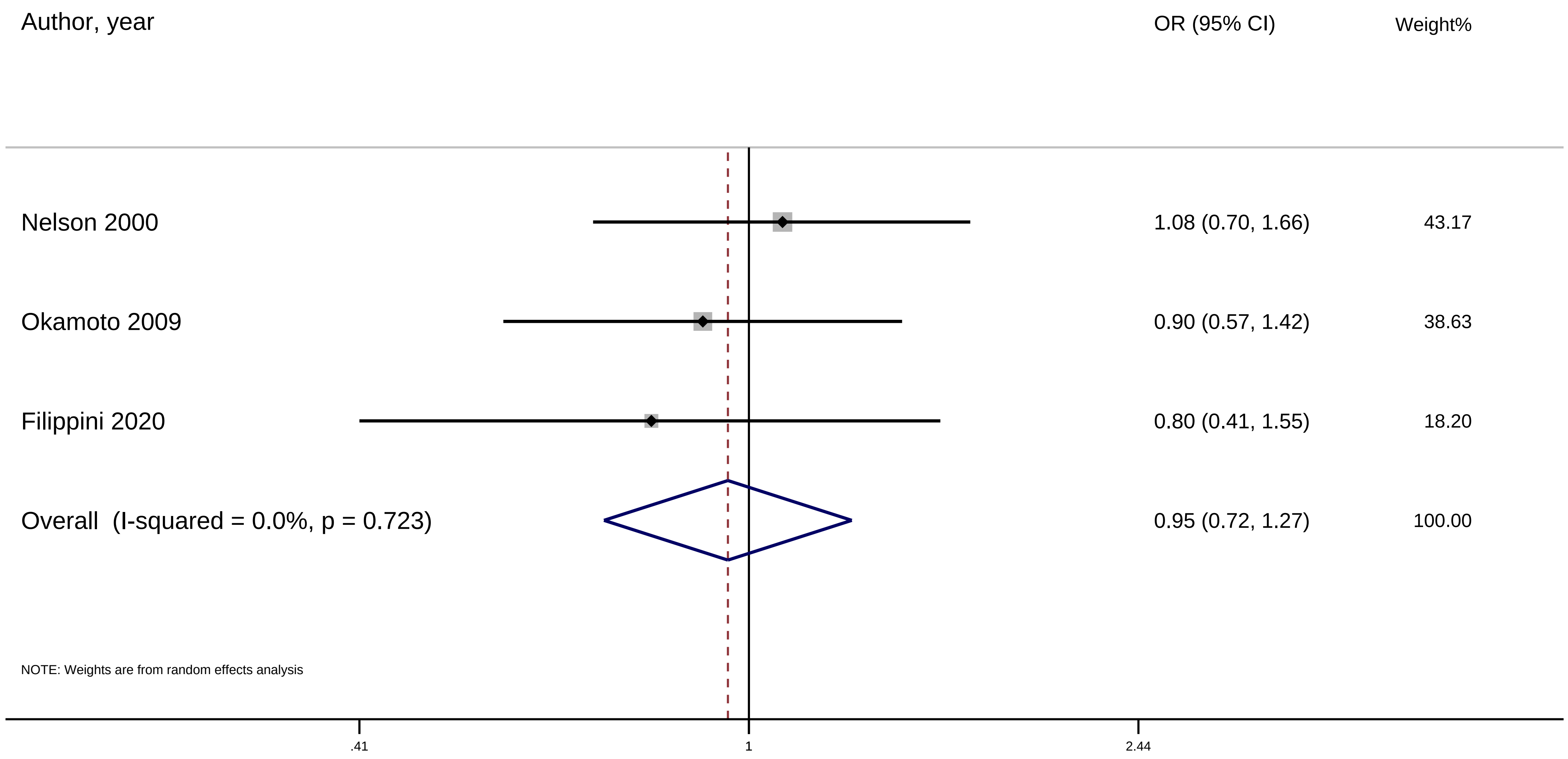

Figure S41. Random-effects meta-analysis of the association between high vitamin diet and ALS

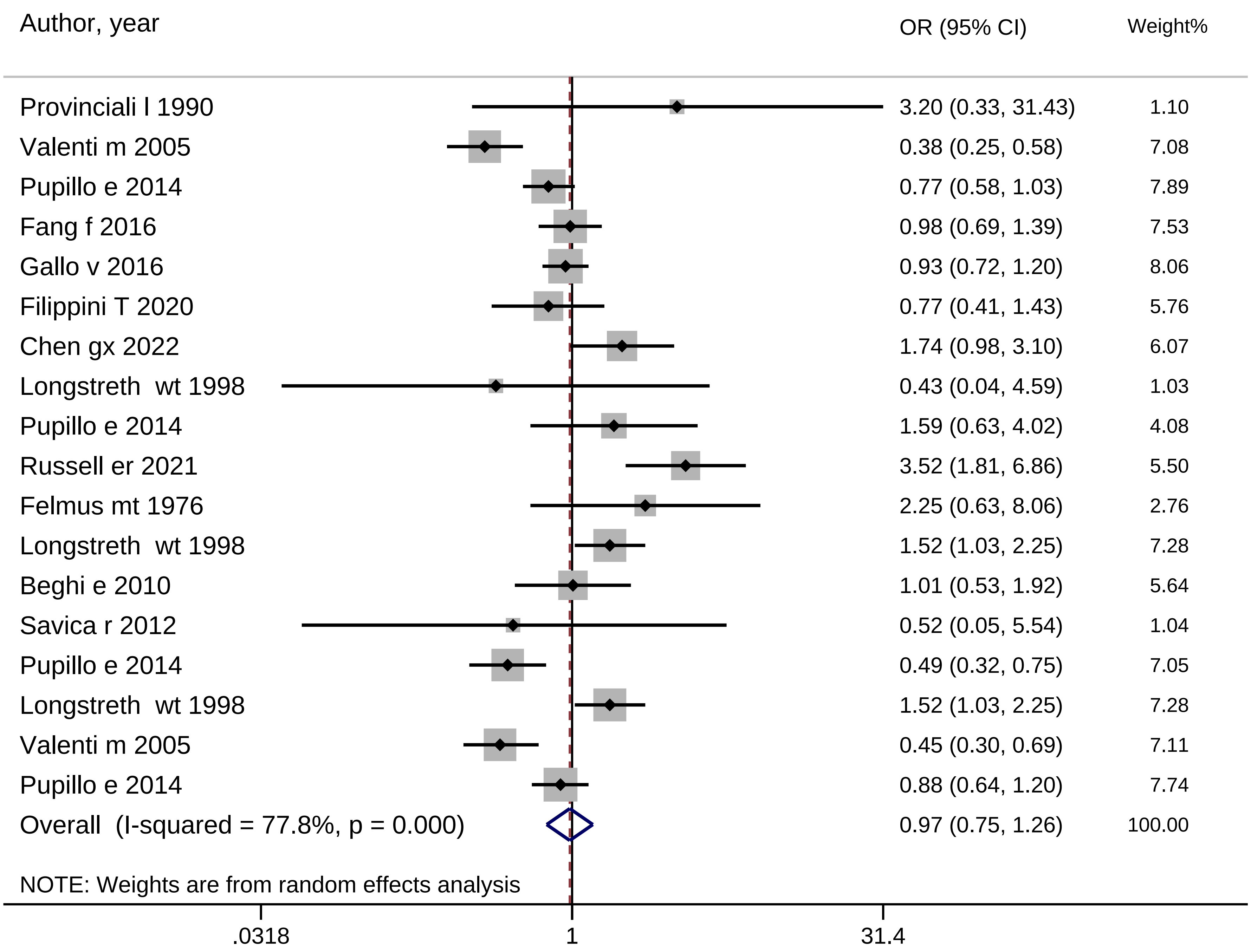

Figure S42. Random-effects meta-analysis of the association between sport-related activity and ALS

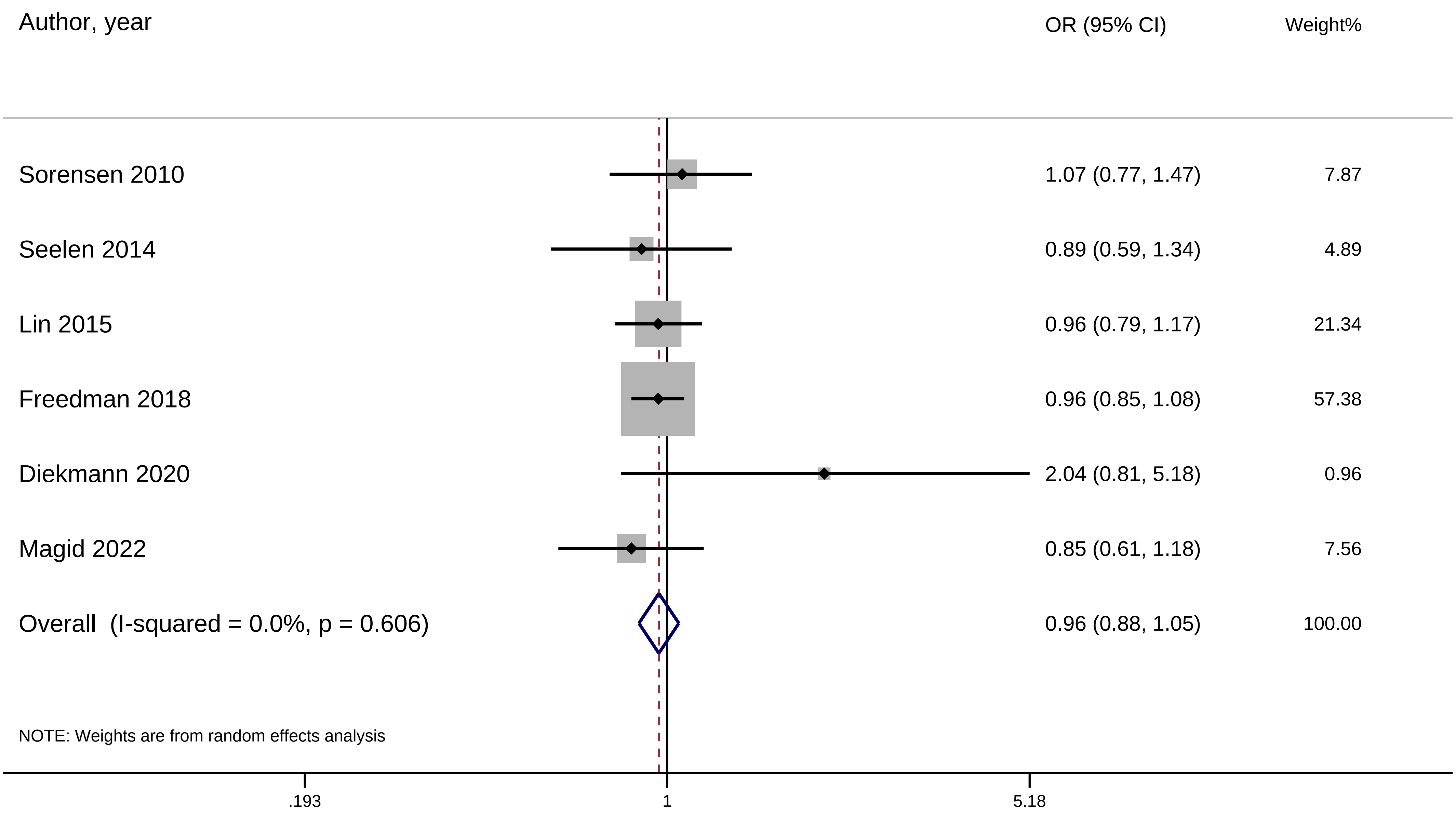

Figure S43. Random-effects meta-analysis of the association between AMI/IS and ALS

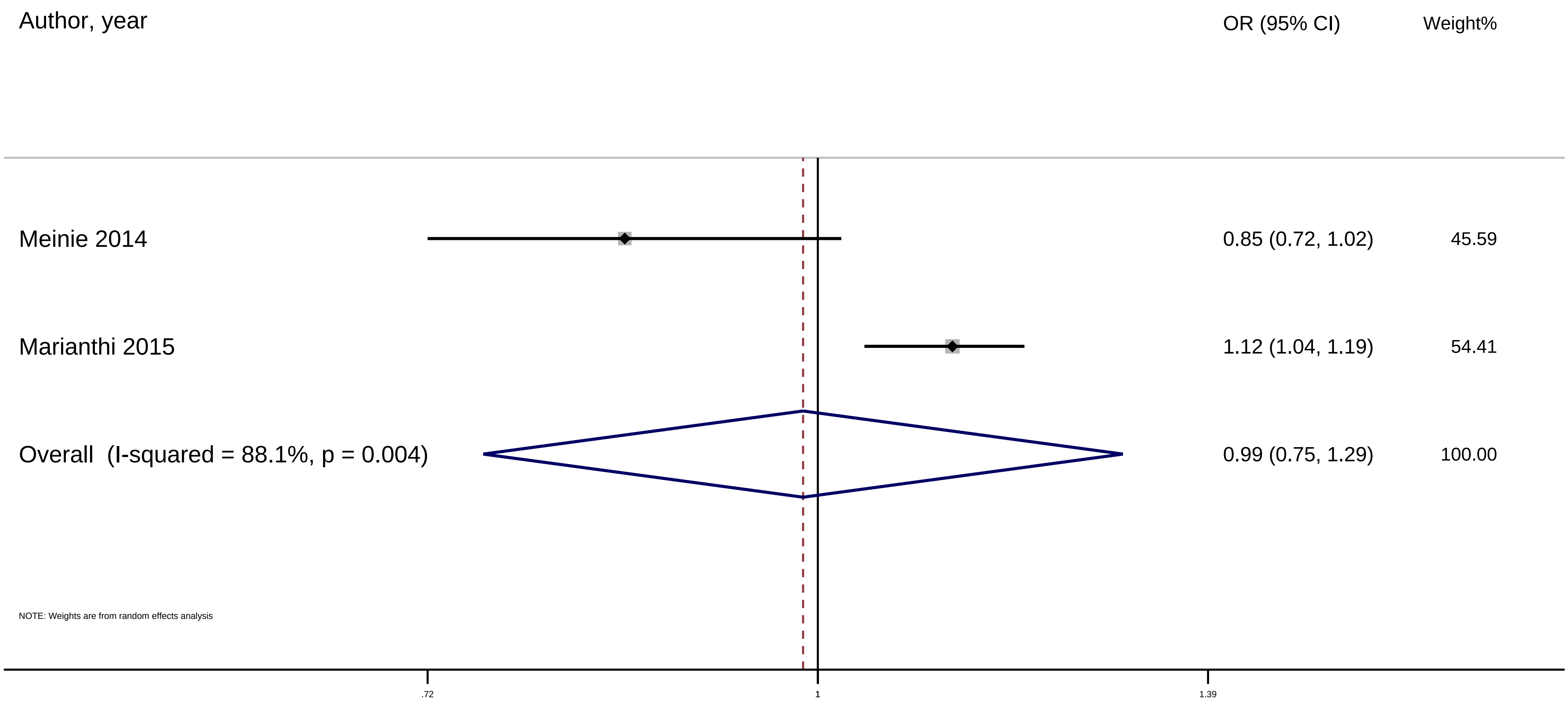

Figure S44. Random-effects meta-analysis of the association between cerebrovascular disease and ALS

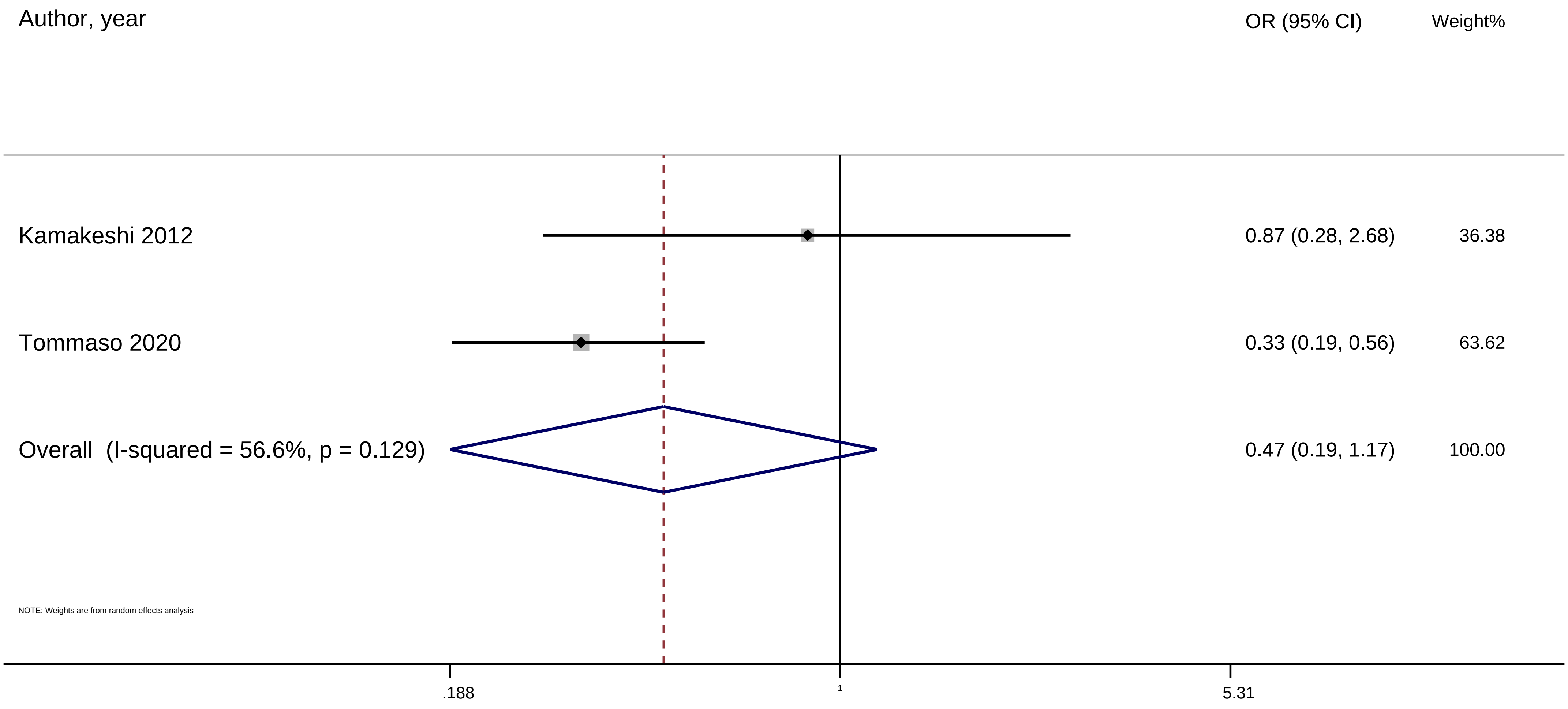

Figure S45. Random-effects meta-analysis of the association between occupation in service industry and ALS

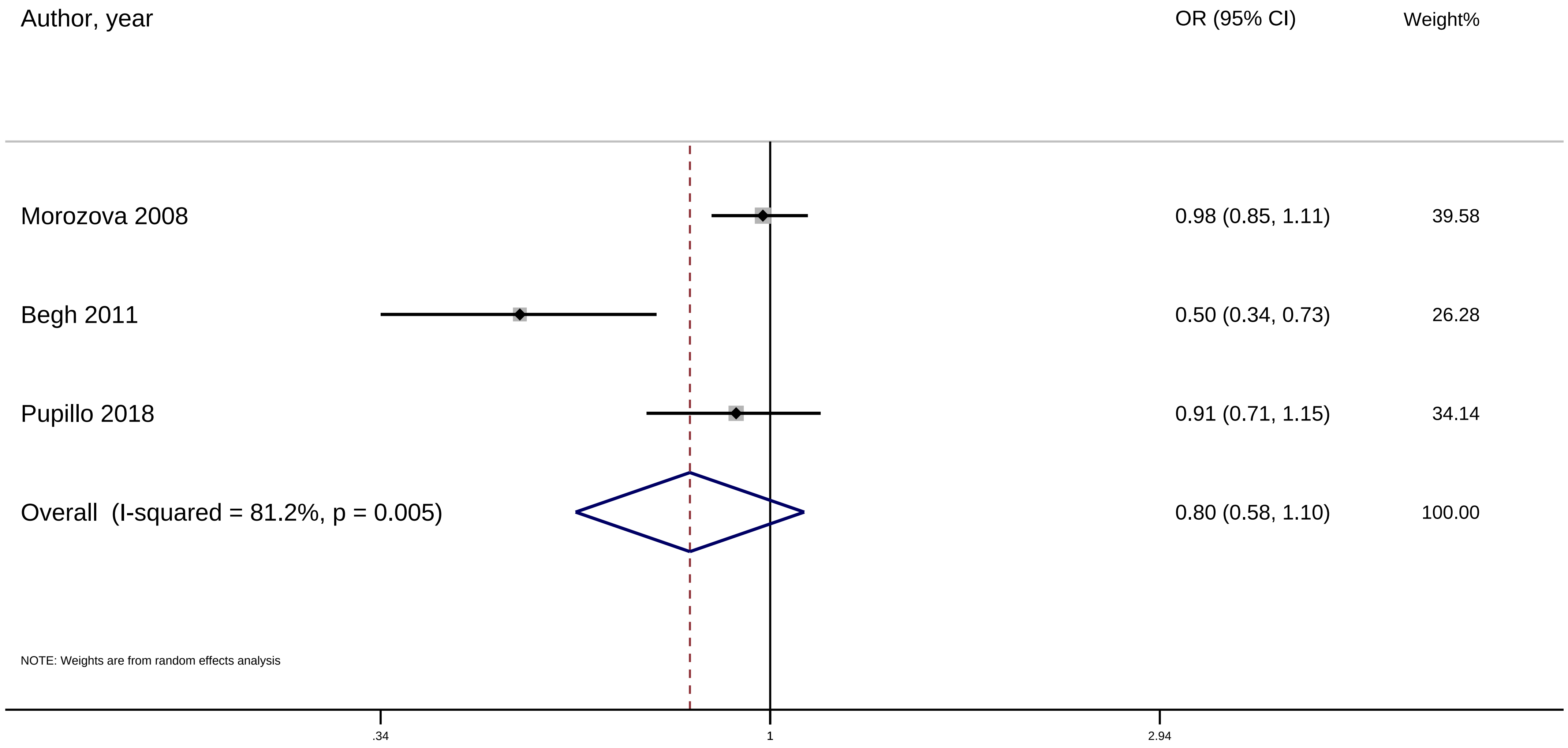

Figure S46. Random-effects meta-analysis of the association between coffee consumption and ALS

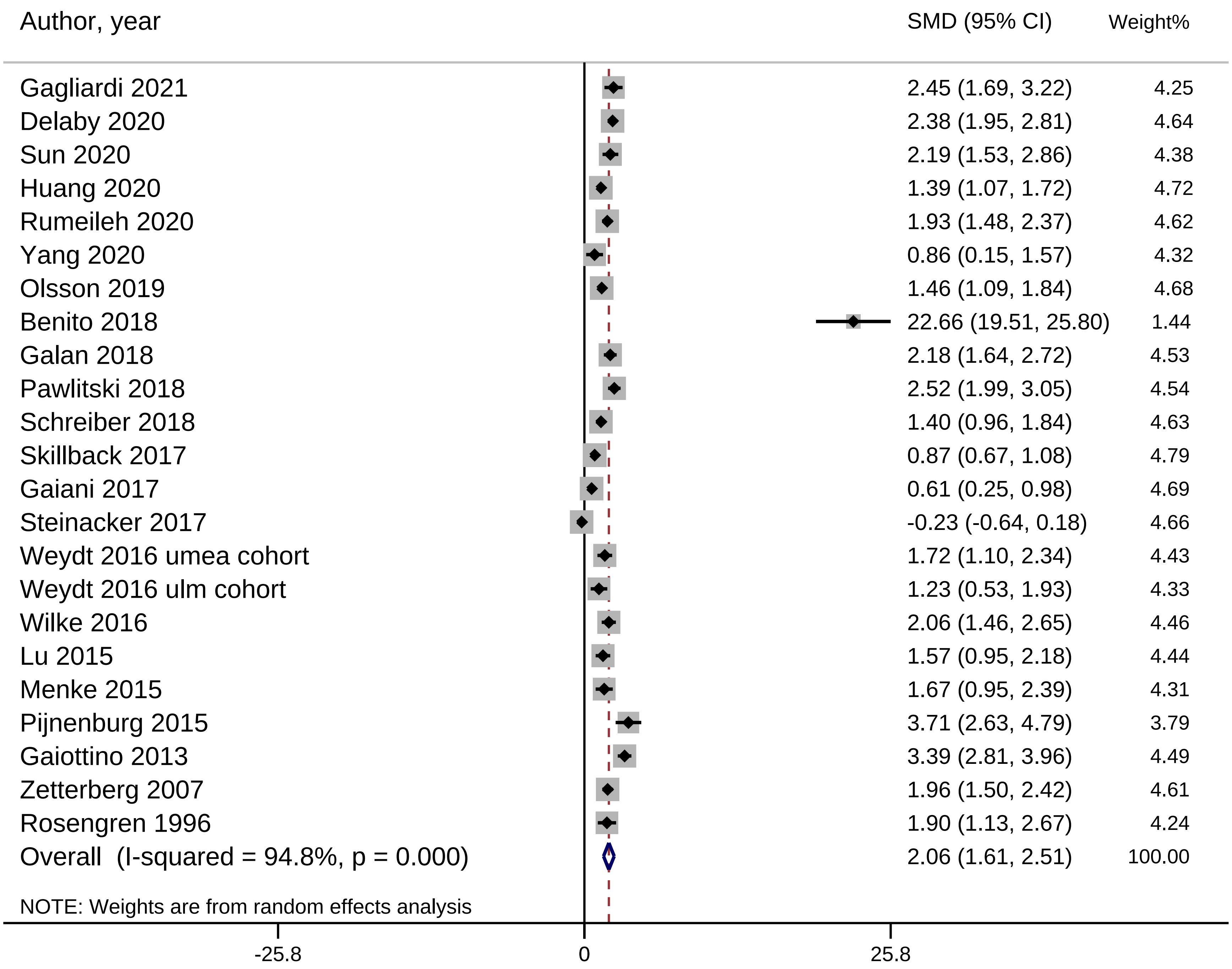

Figure S47. Random-effects meta-analysis of the association between CSF NFL level and ALS

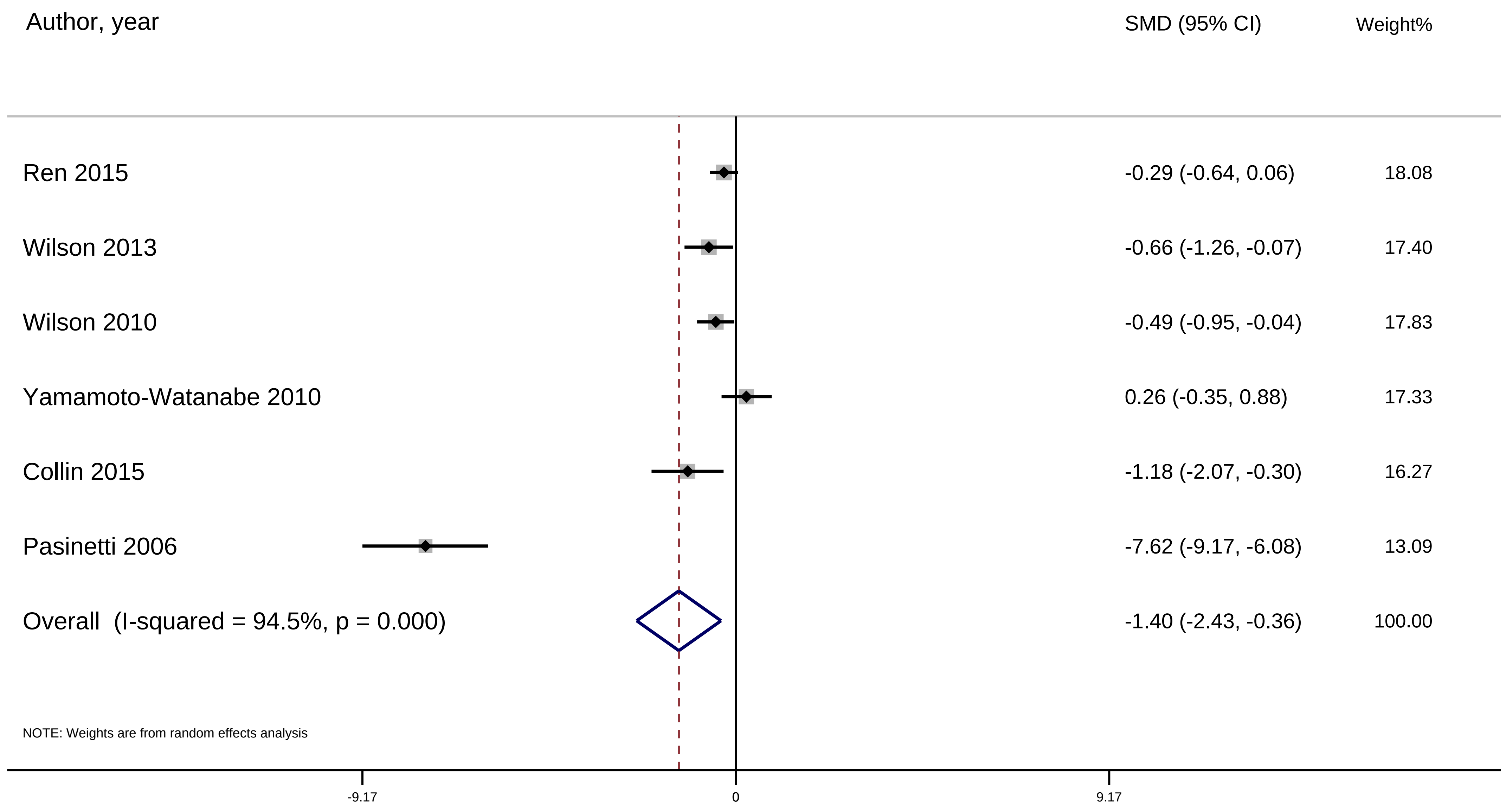

Figure S48. Funnel plot of meta-analysis of the association between CSF Cystatin C level and ALS

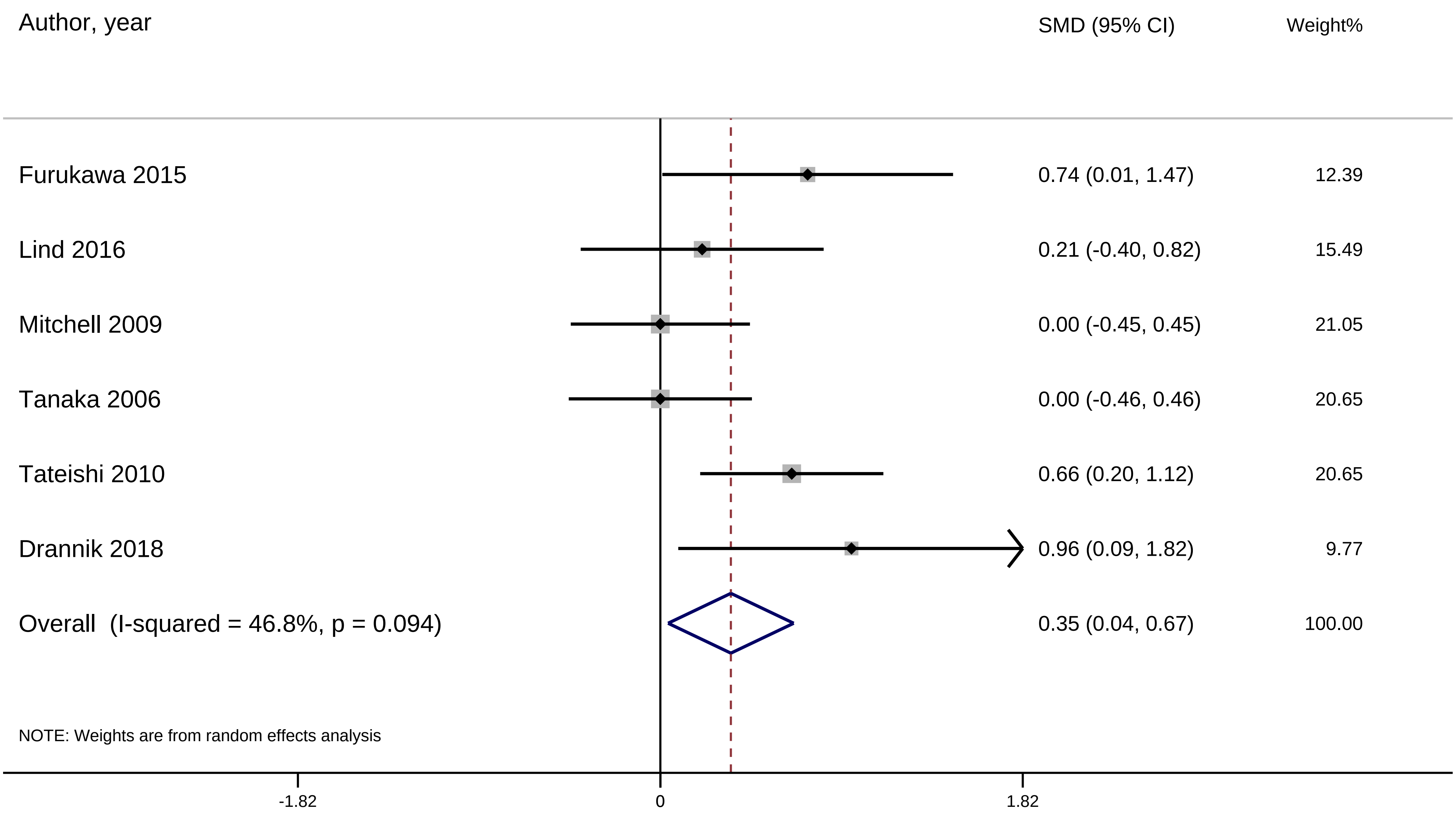

Figure S49. Random-effects meta-analysis of the association between CSF TNF- α level and ALS

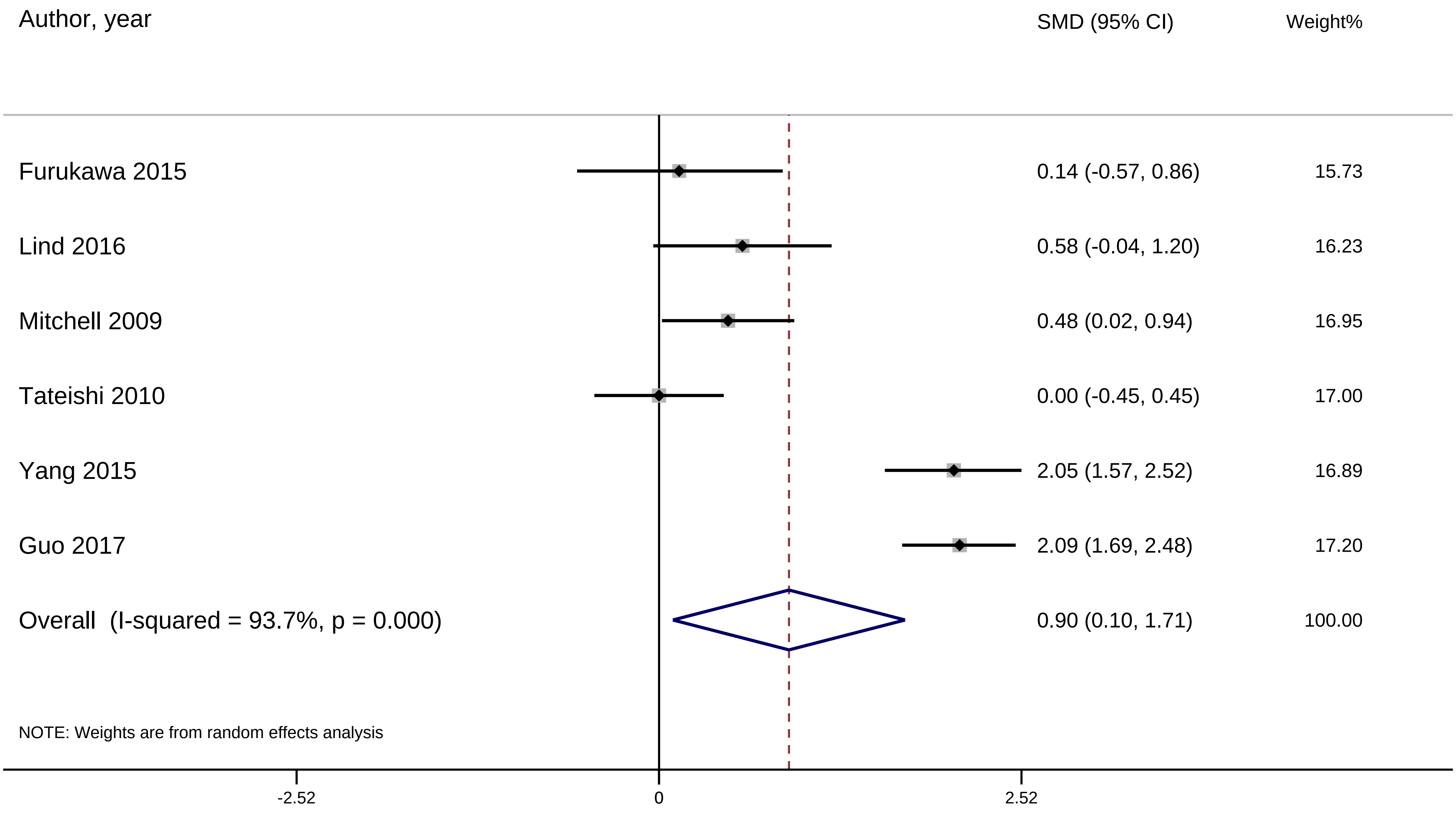

Figure S50. Random-effects meta-analysis of the association between CSF MIP-1 α level and ALS

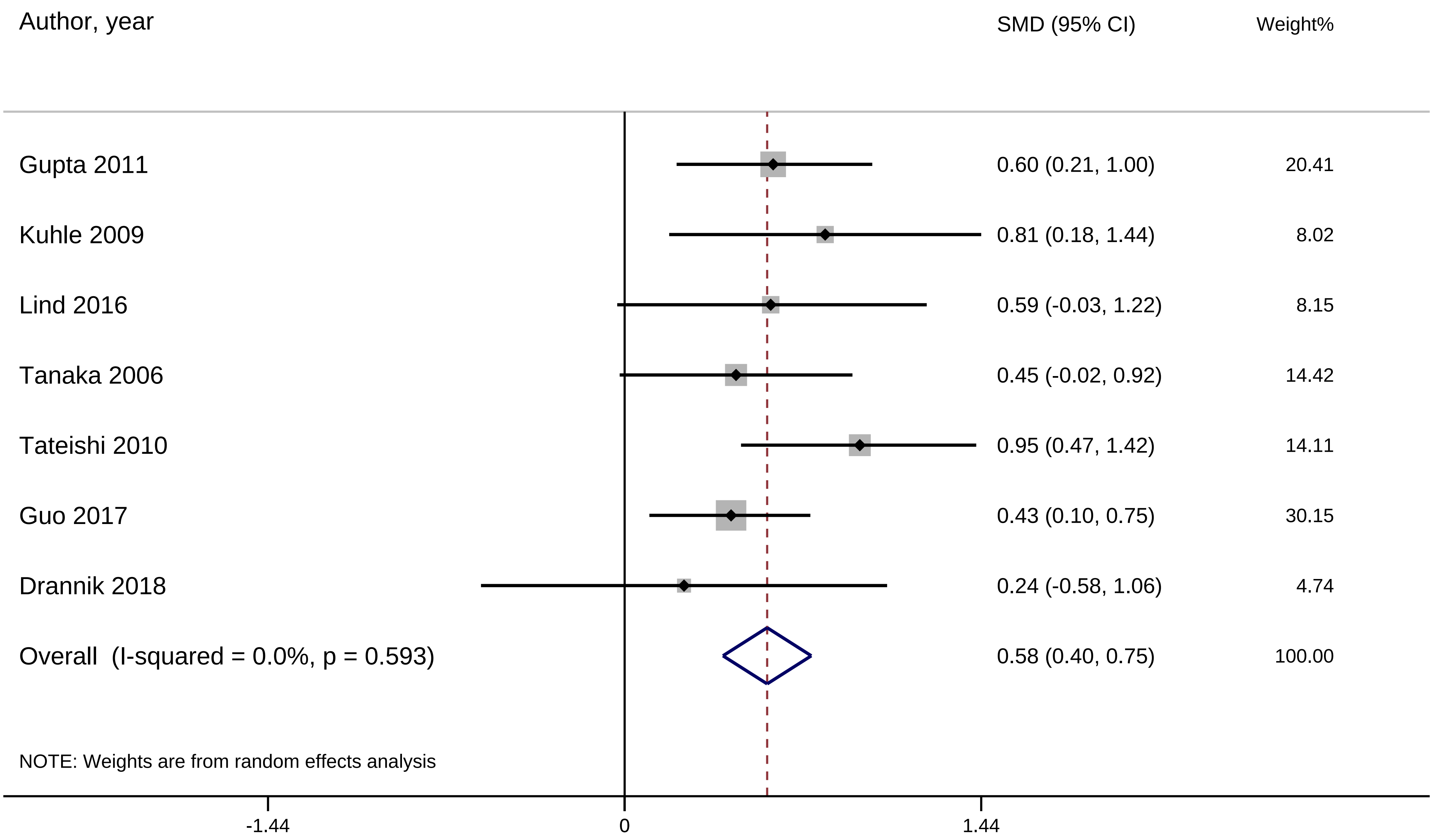

Figure S51. Random-effects meta-analysis of the association between CSF MCP-1 level and ALS

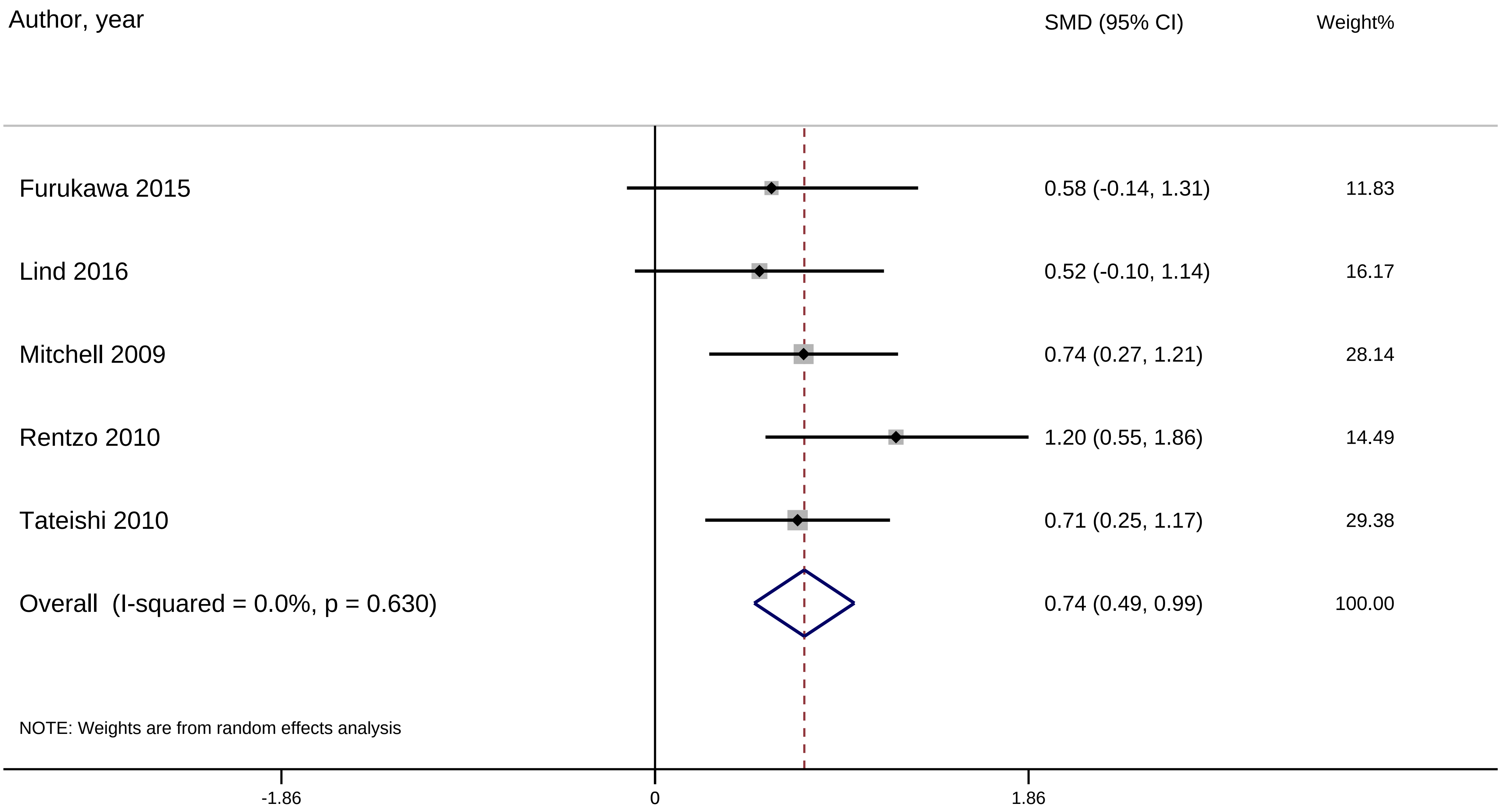

Figure S52. Random-effects meta-analysis of the association between CSF IL-17 level and ALS

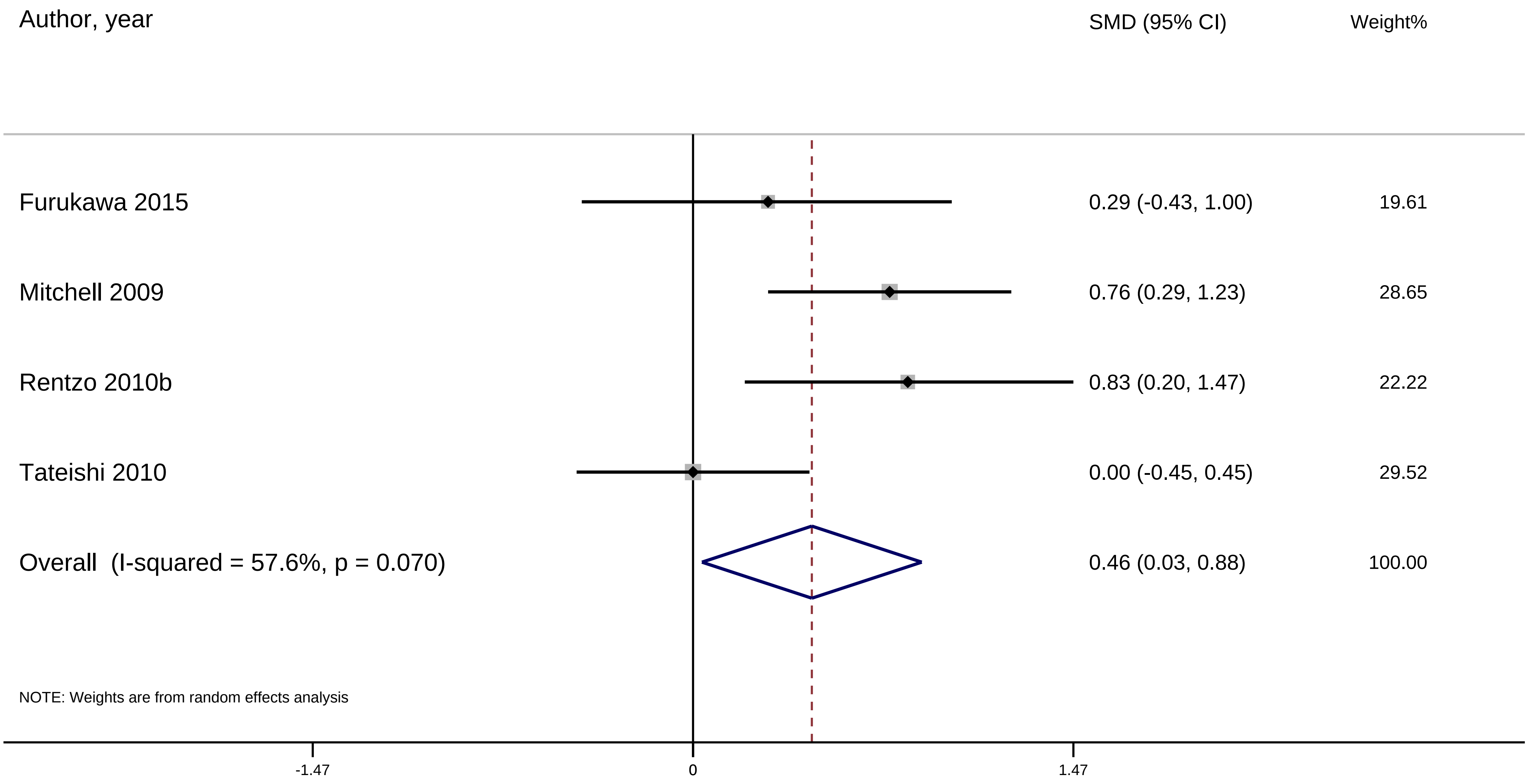

Figure S53. Random-effects meta-analysis of the association between CSF IL-15 level and ALS

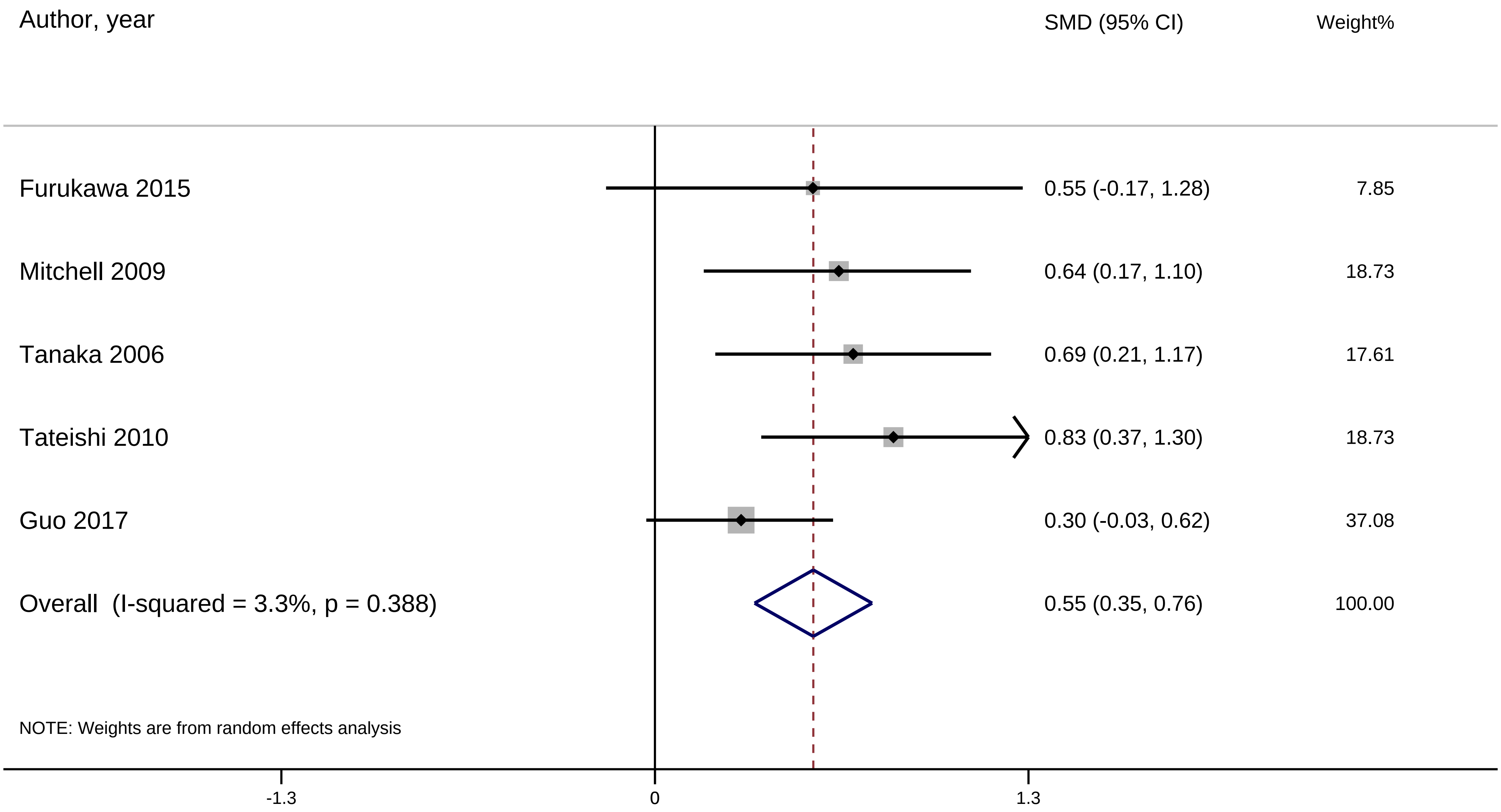

Figure S54. Random-effects meta-analysis of the association between CSF G-CSF level and ALS

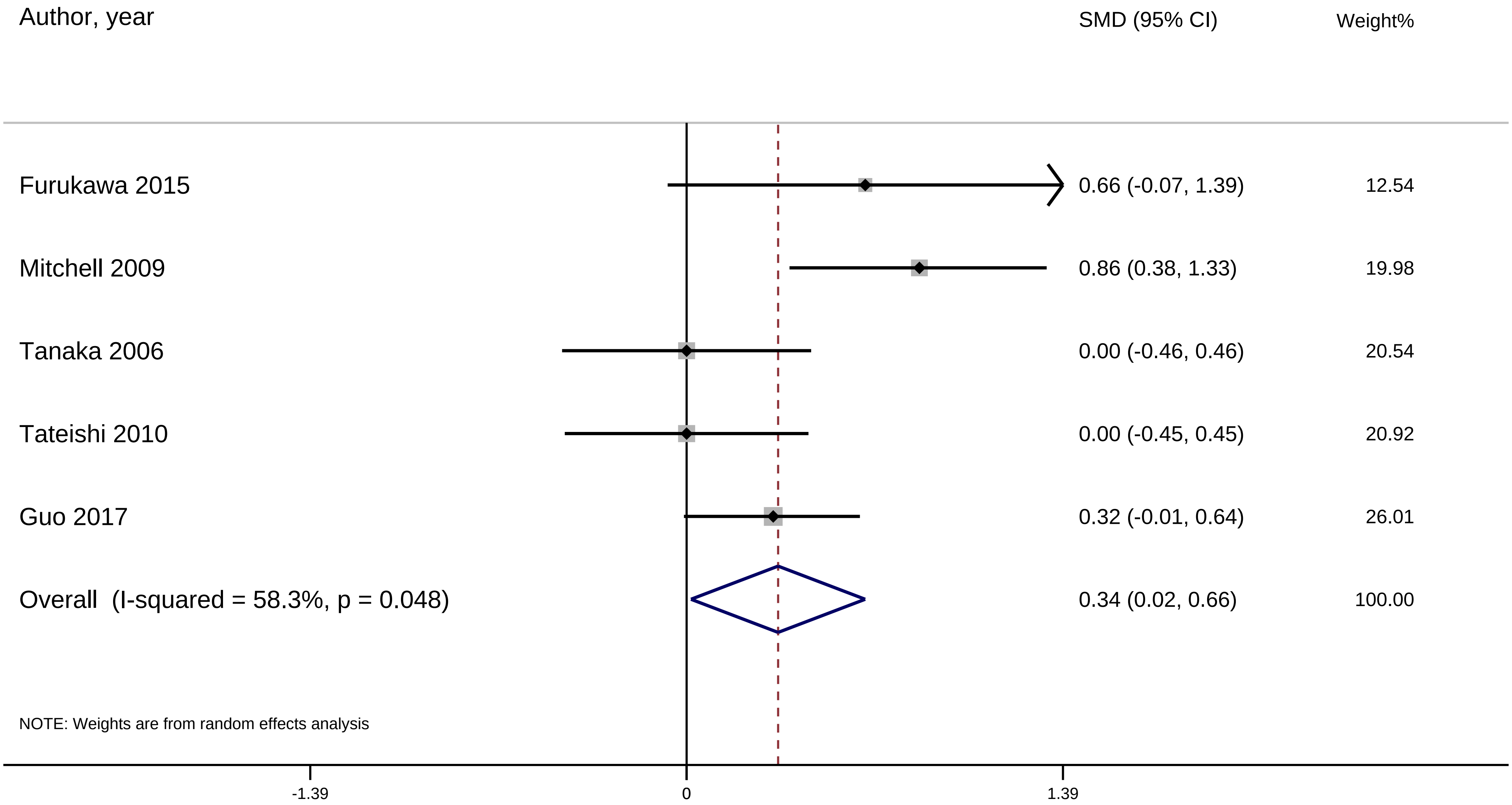

Figure S55. Random-effects meta-analysis of the association between CSF IL-2 level and ALS

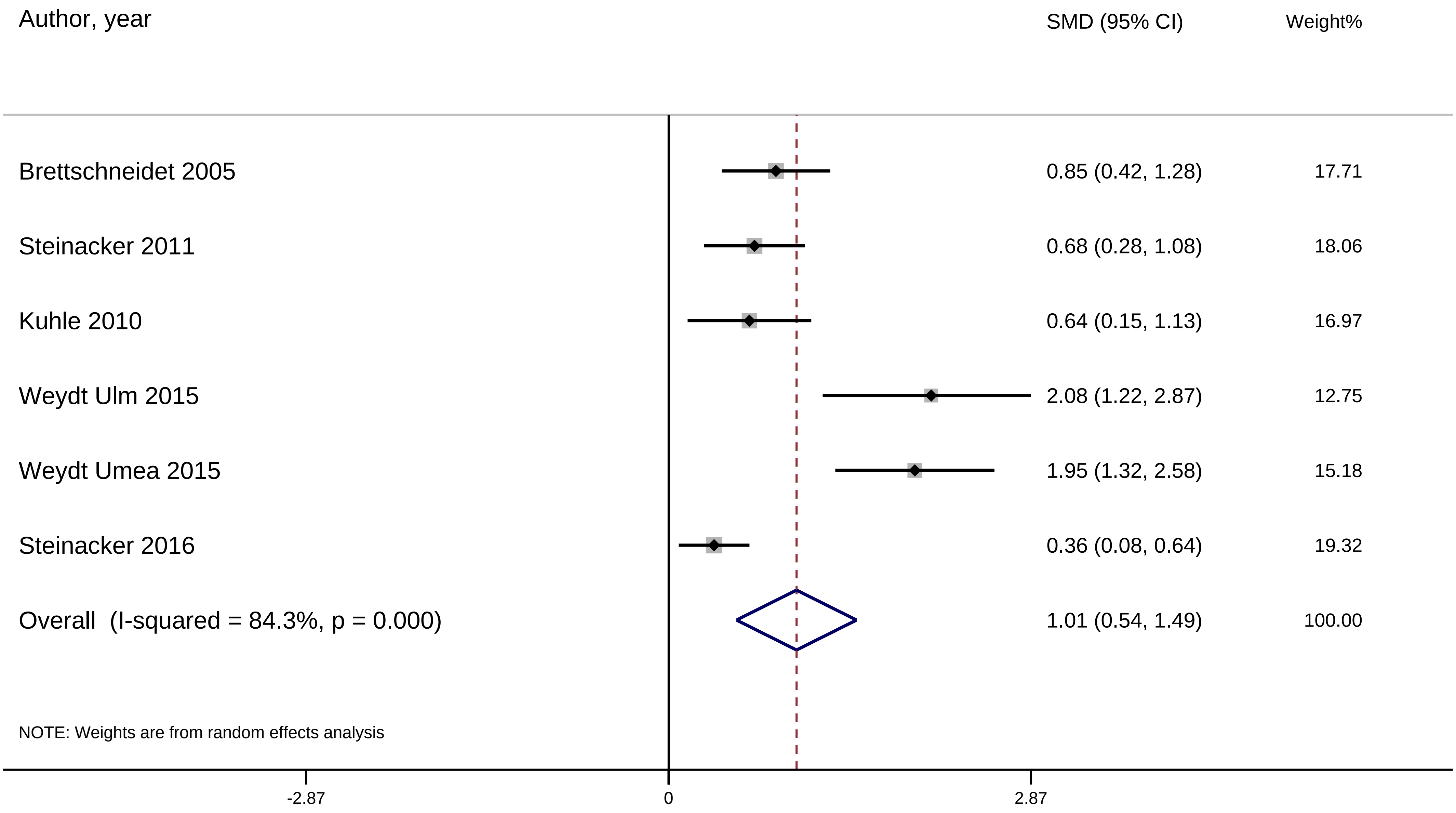

Figure S56. Random-effects meta-analysis of the association between CSF NFH level and ALS

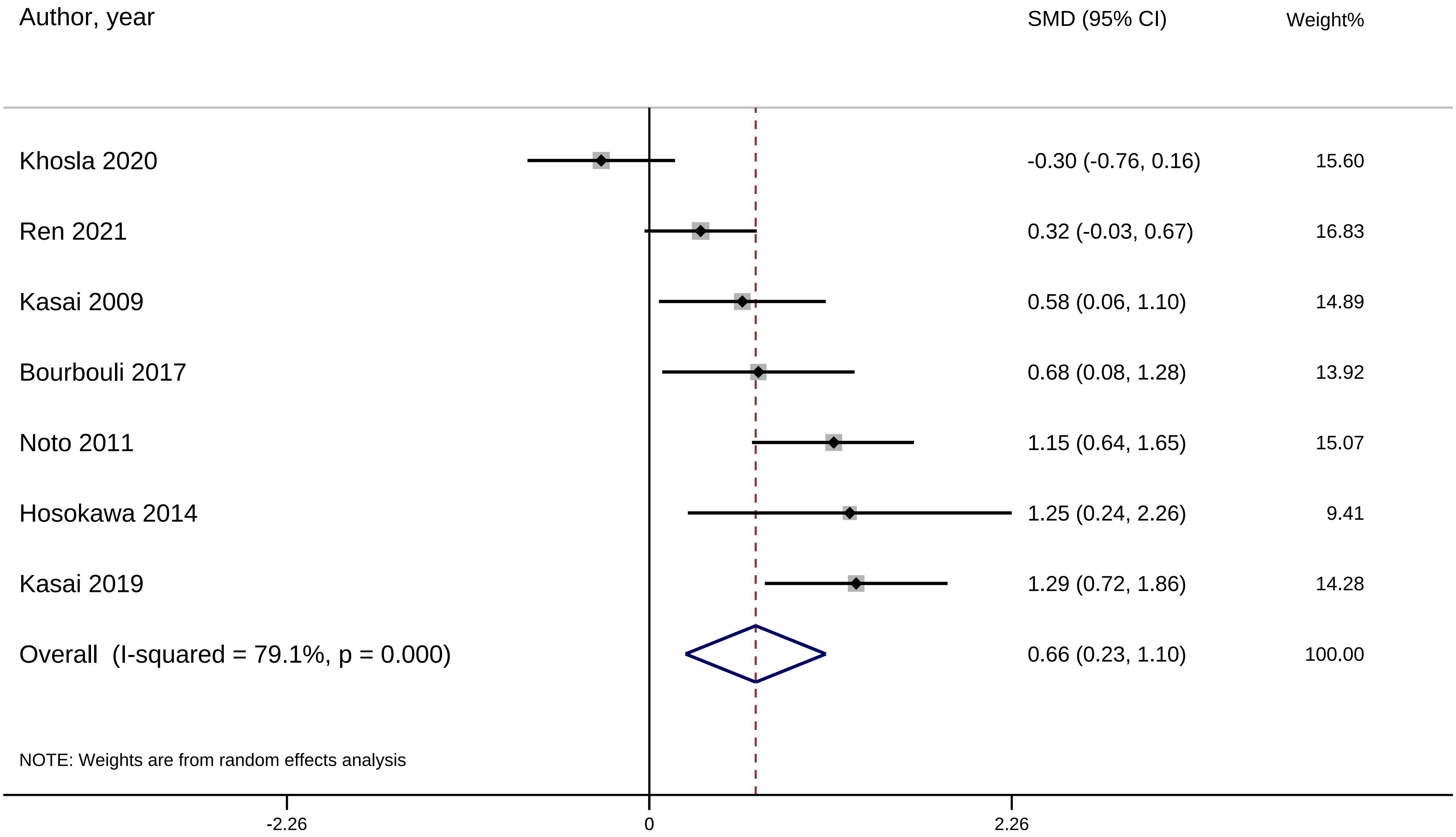

Figure S57. Random-effects meta-analysis of the association between CSF TDP-43 level and ALS

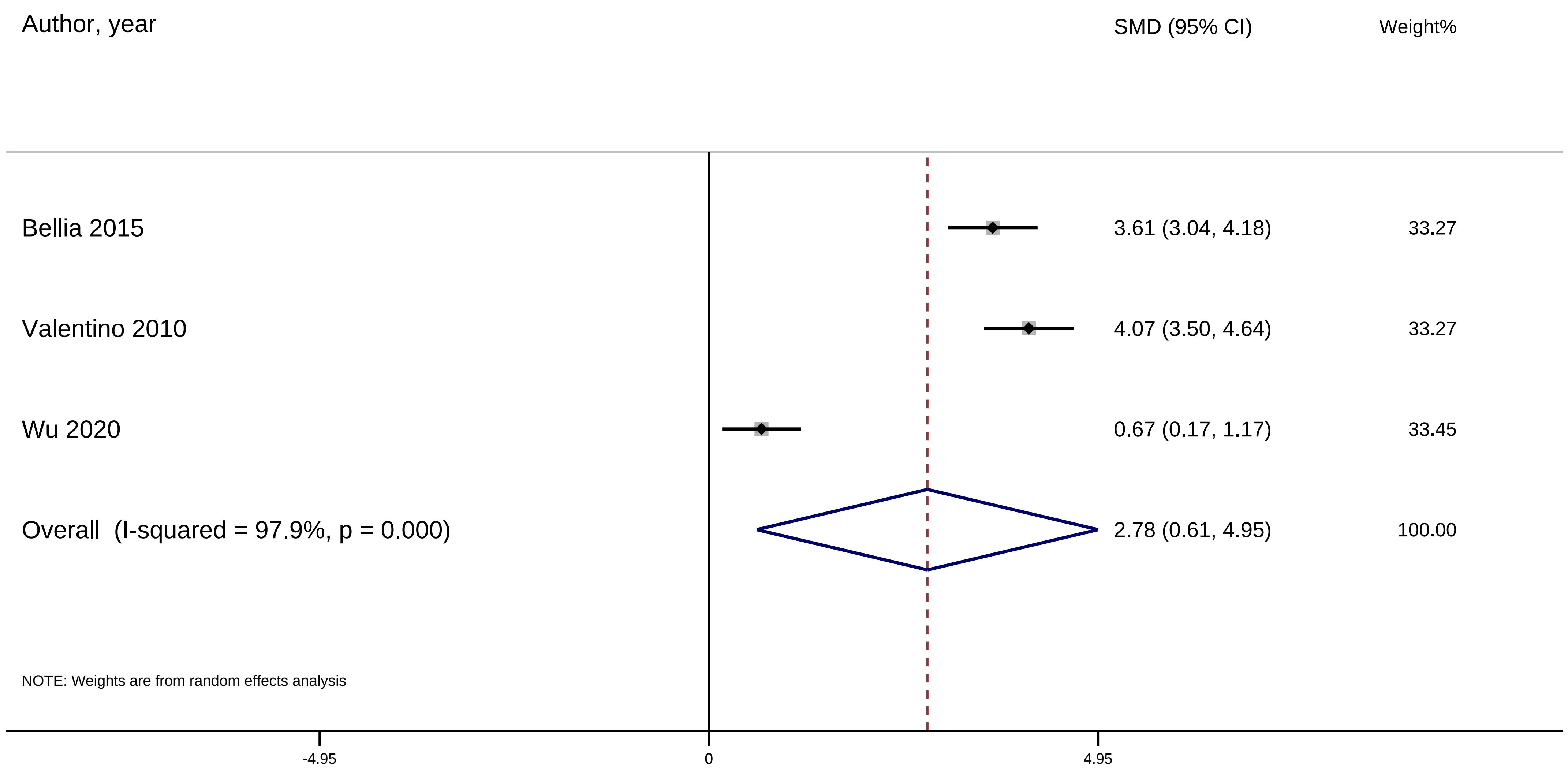

Figure S58. Random-effects meta-analysis of the association between CSF homocysteine level and ALS

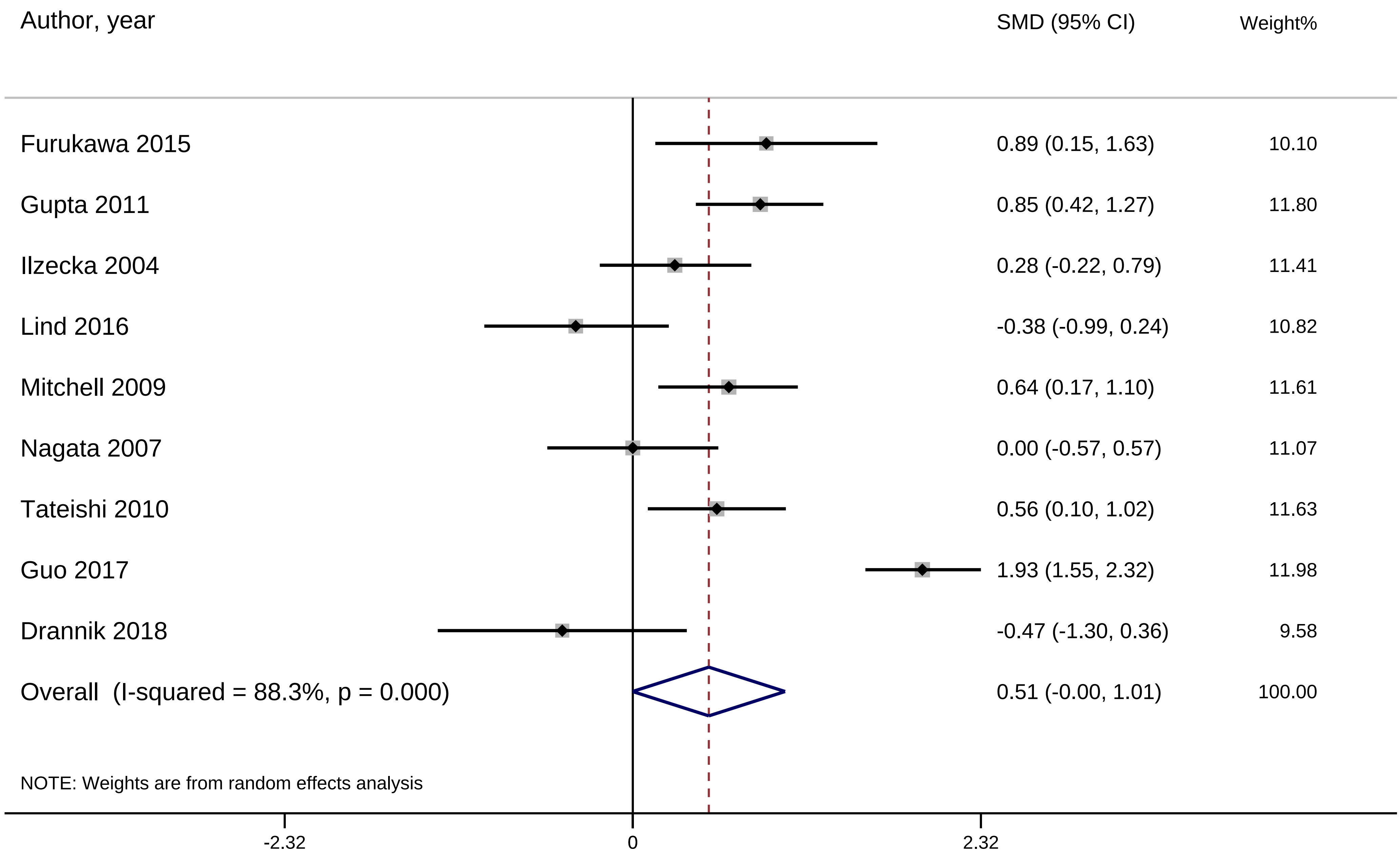

Figure S59. Random-effects meta-analysis of the association between CSF VEGF level and ALS

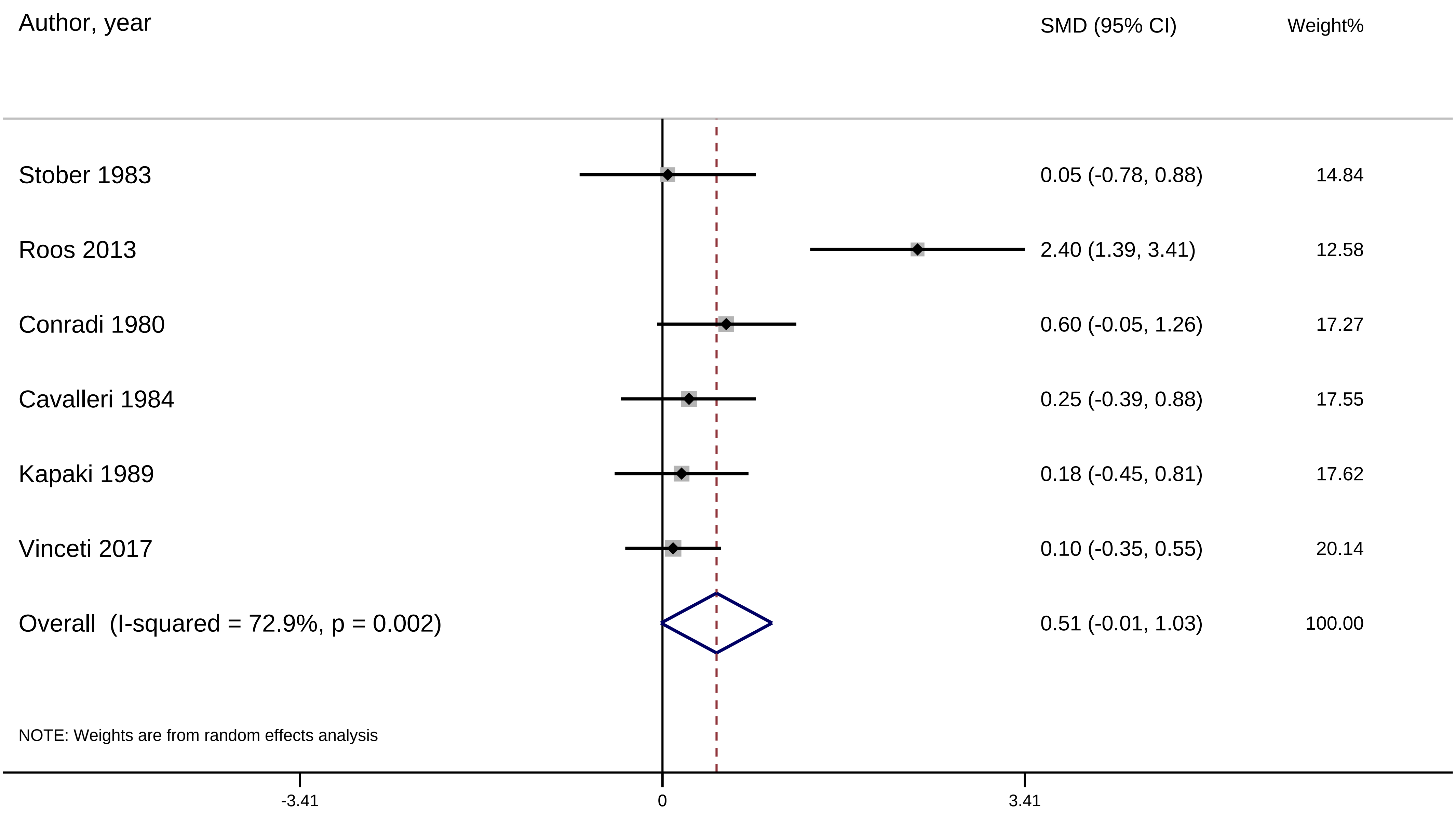

Figure S60. Random-effects meta-analysis of the association between CSF lead level and

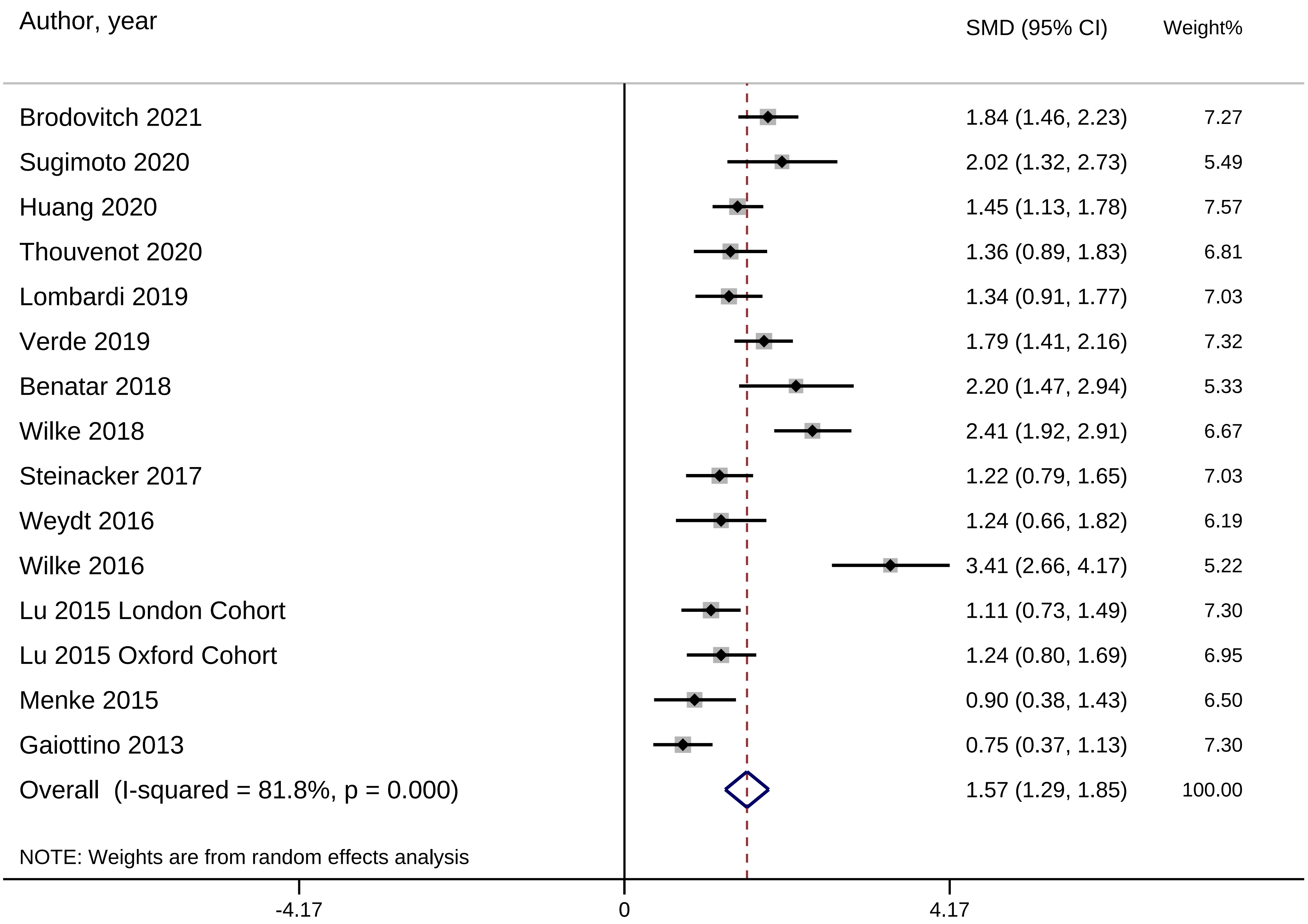

Figure S61. Random-effects meta-analysis of the association between serum NFL level and ALS

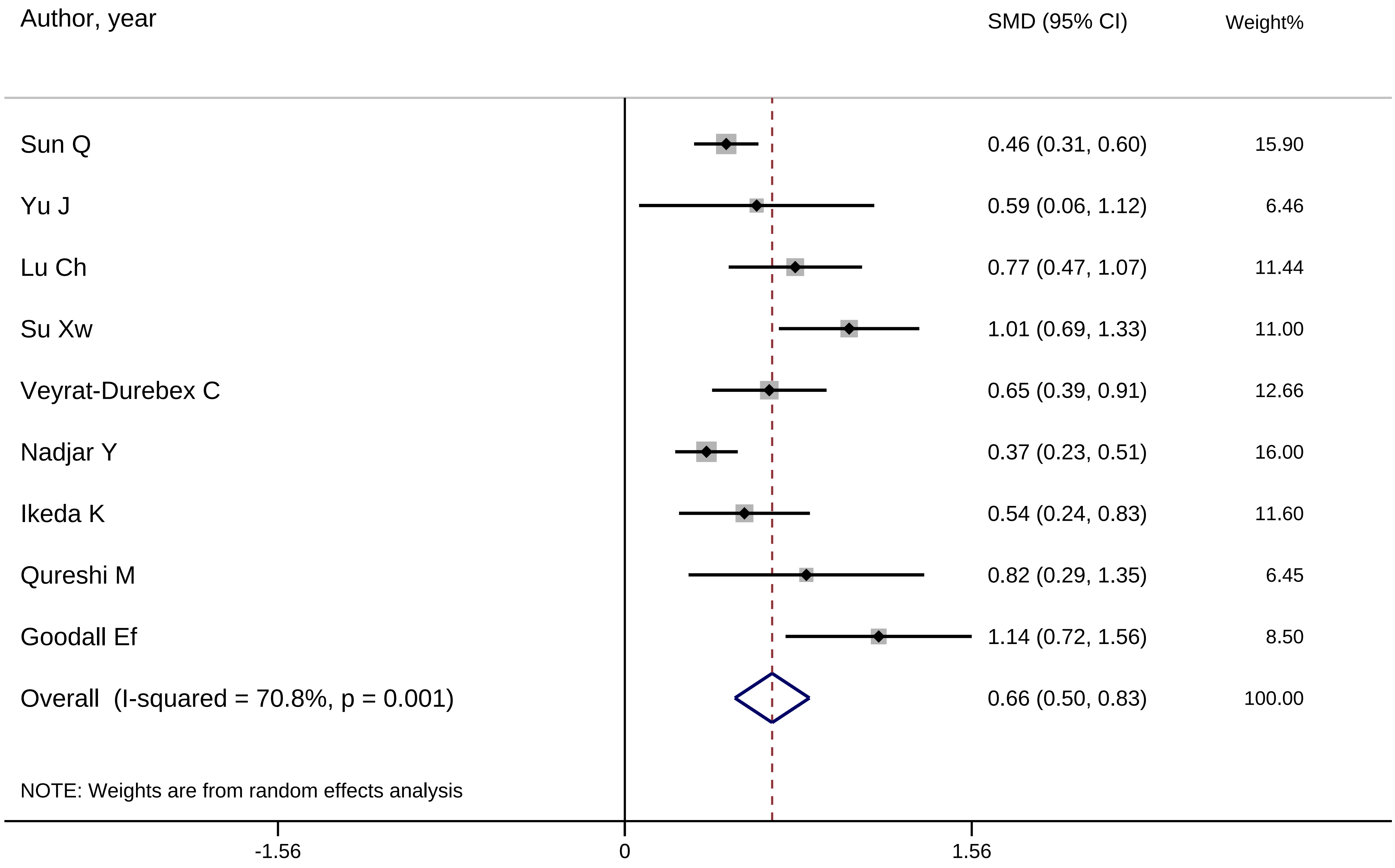

Figure S62. Random-effects meta-analysis of the association between serum feritin level and ALS

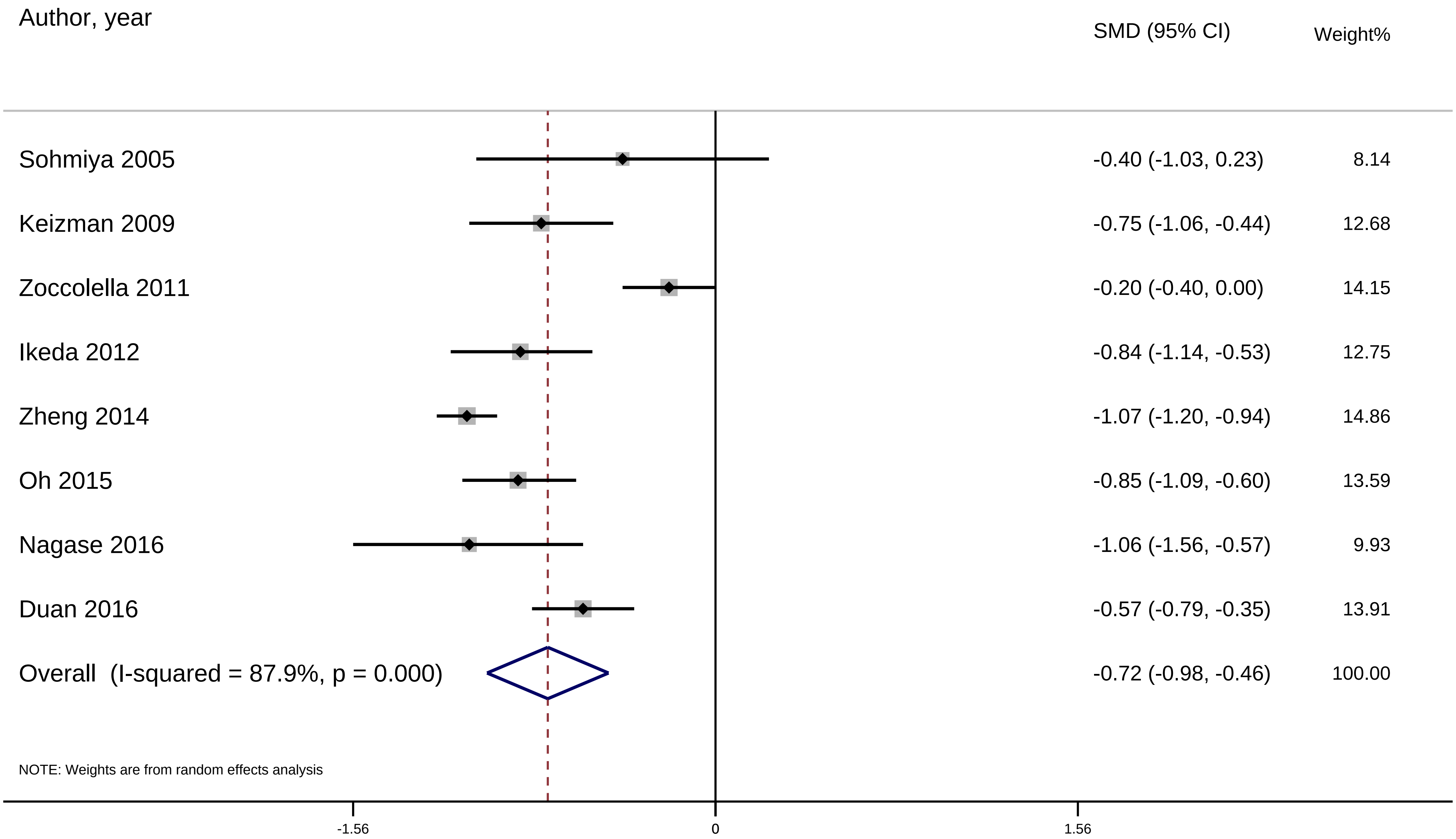

Figure S63. Random-effects meta-analysis of the association between serum uric acid level and ALS

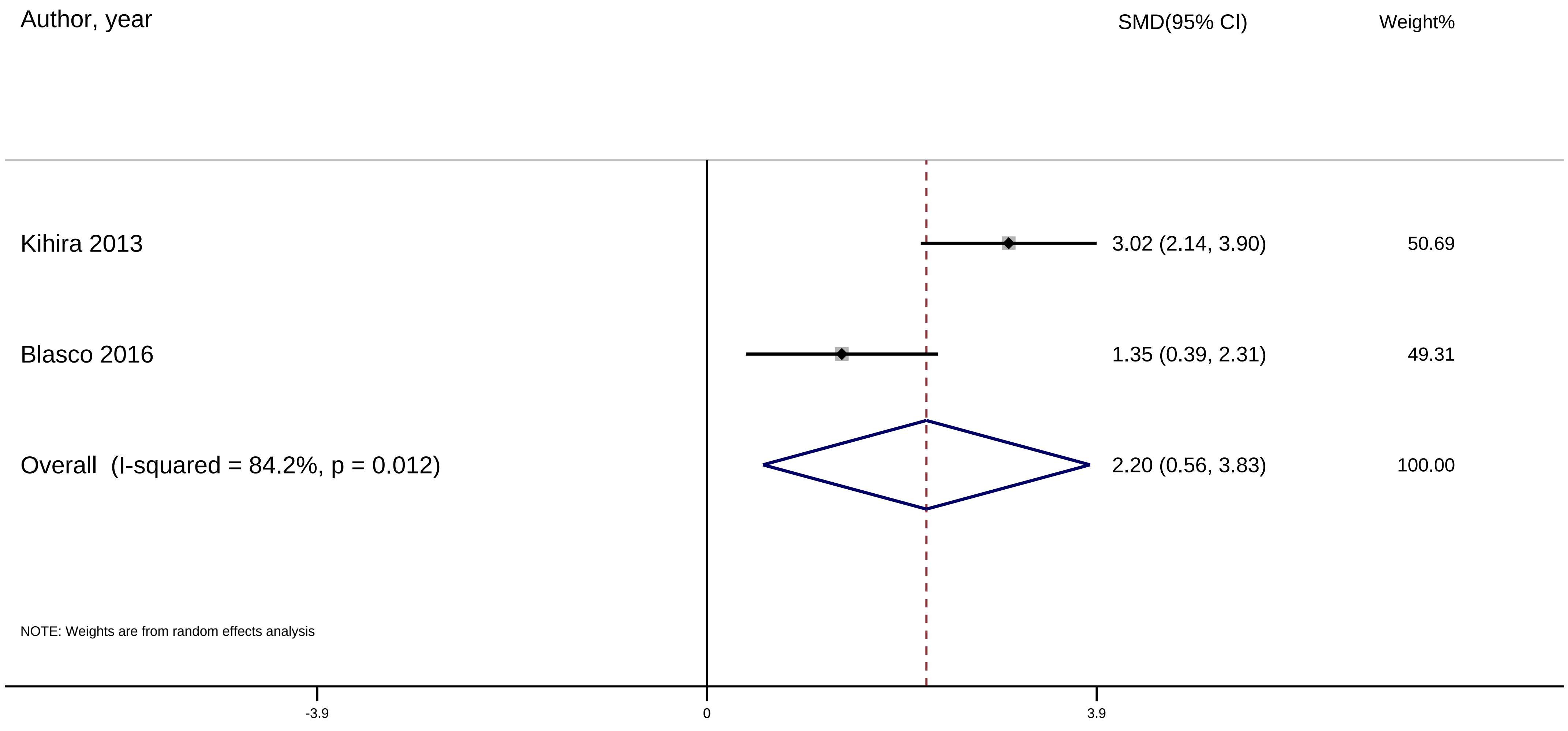

Figure S64. Random-effects meta-analysis of the association between serum 8-OHdG level and ALS

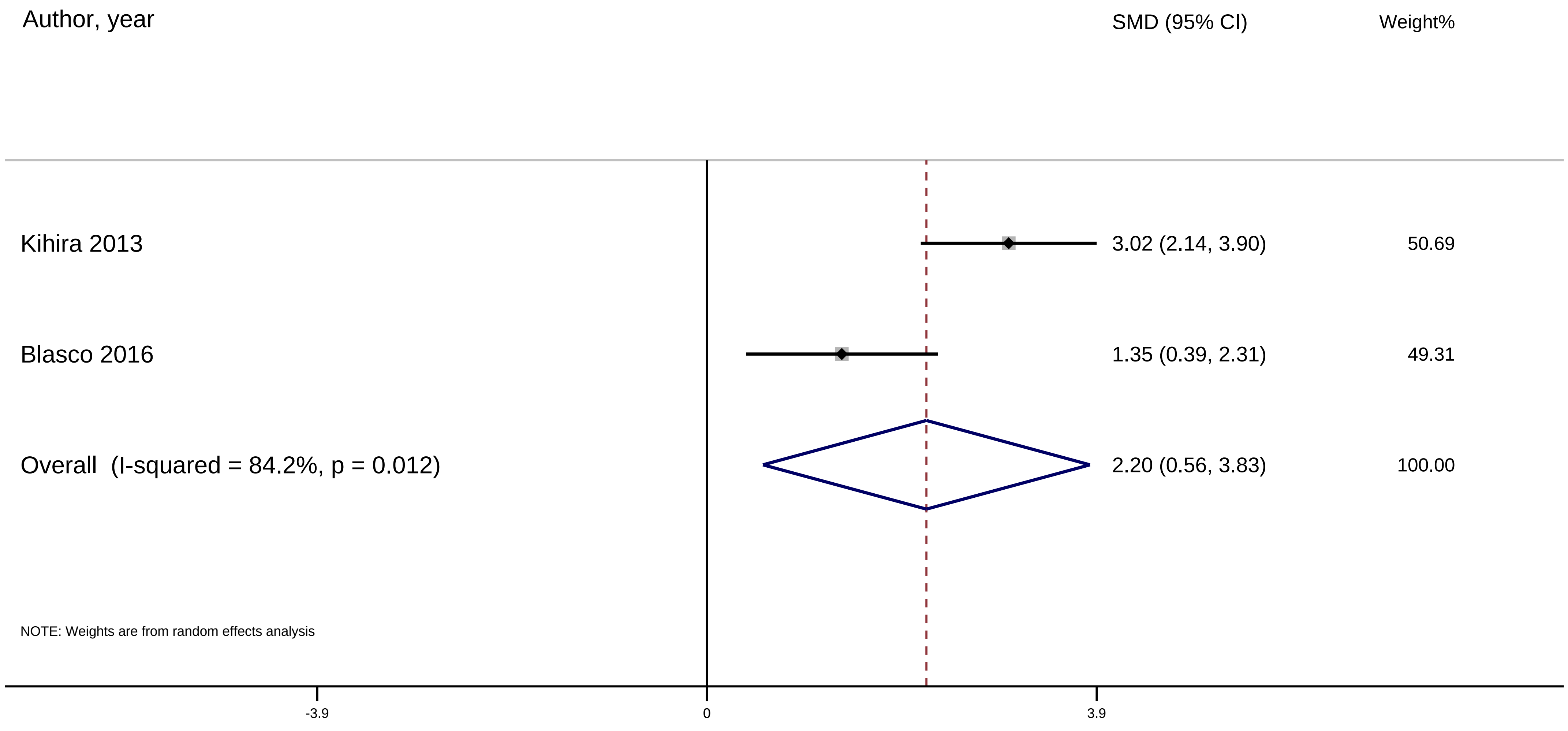

Figure S65. Random-effects meta-analysis of the association between serum GSH level and ALS

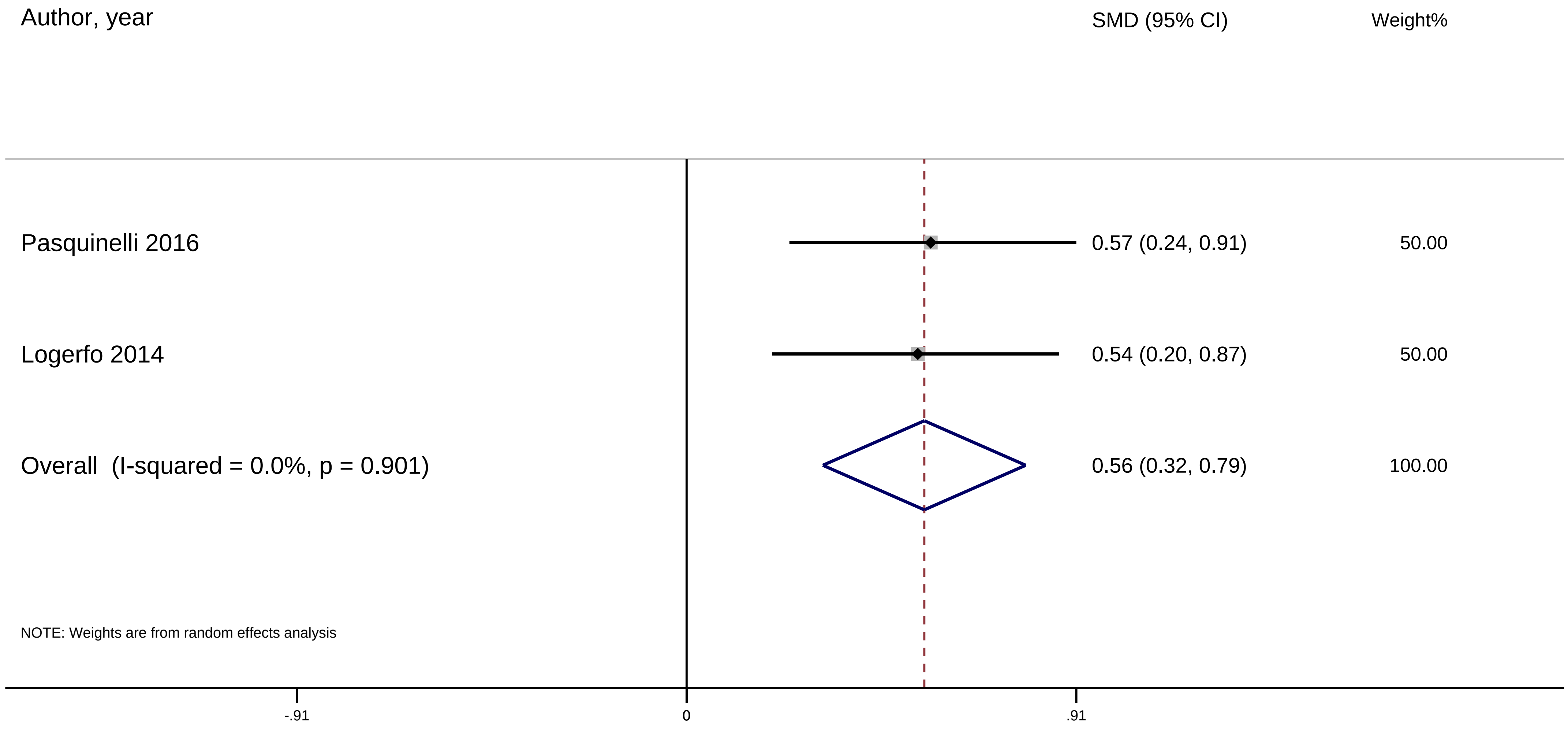

Figure S66. Random-effects meta-analysis of the association between serum AOPP level and ALS

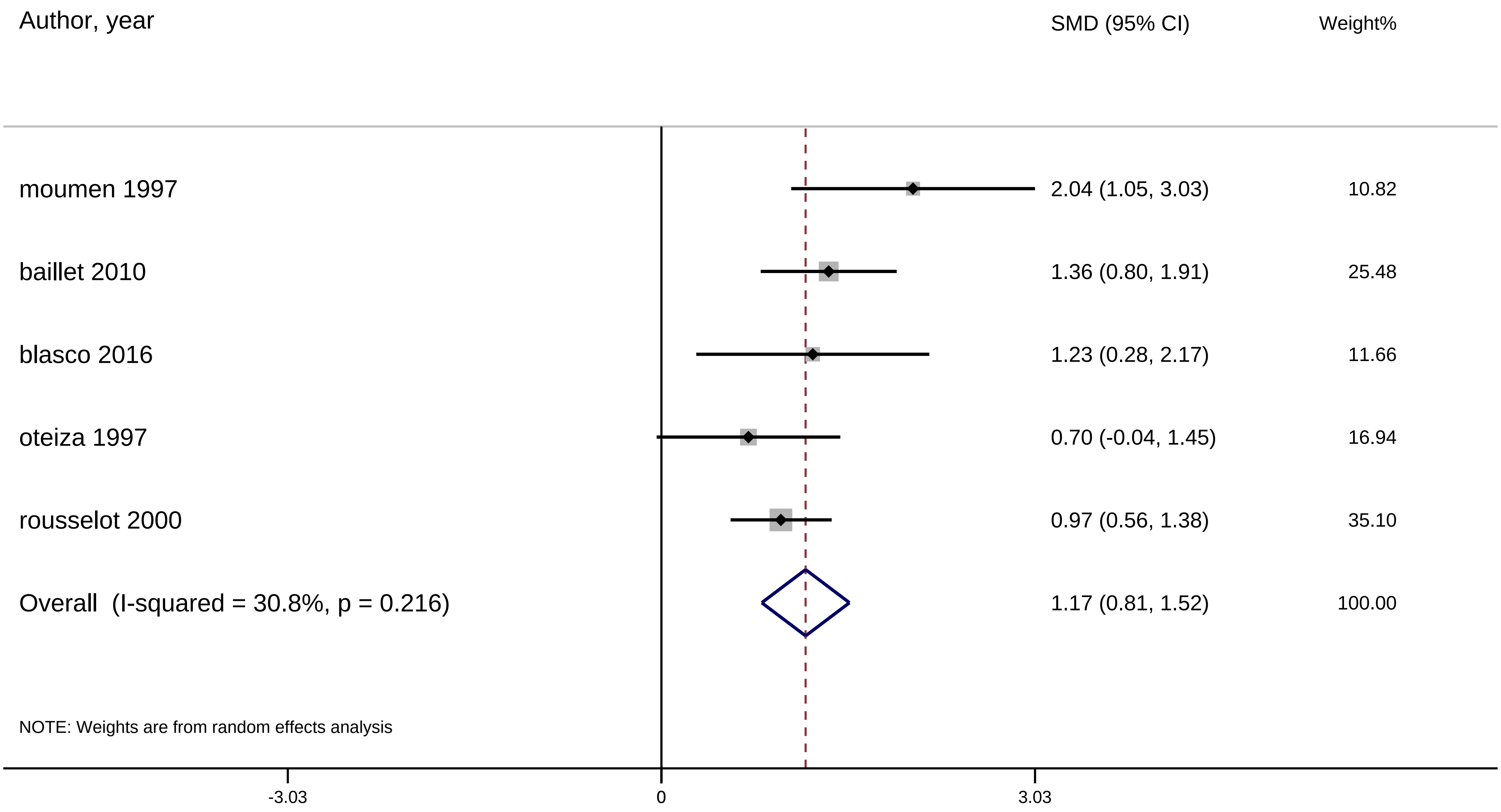

Figure S67. Random-effects meta-analysis of the association between serum MDA level and ALS

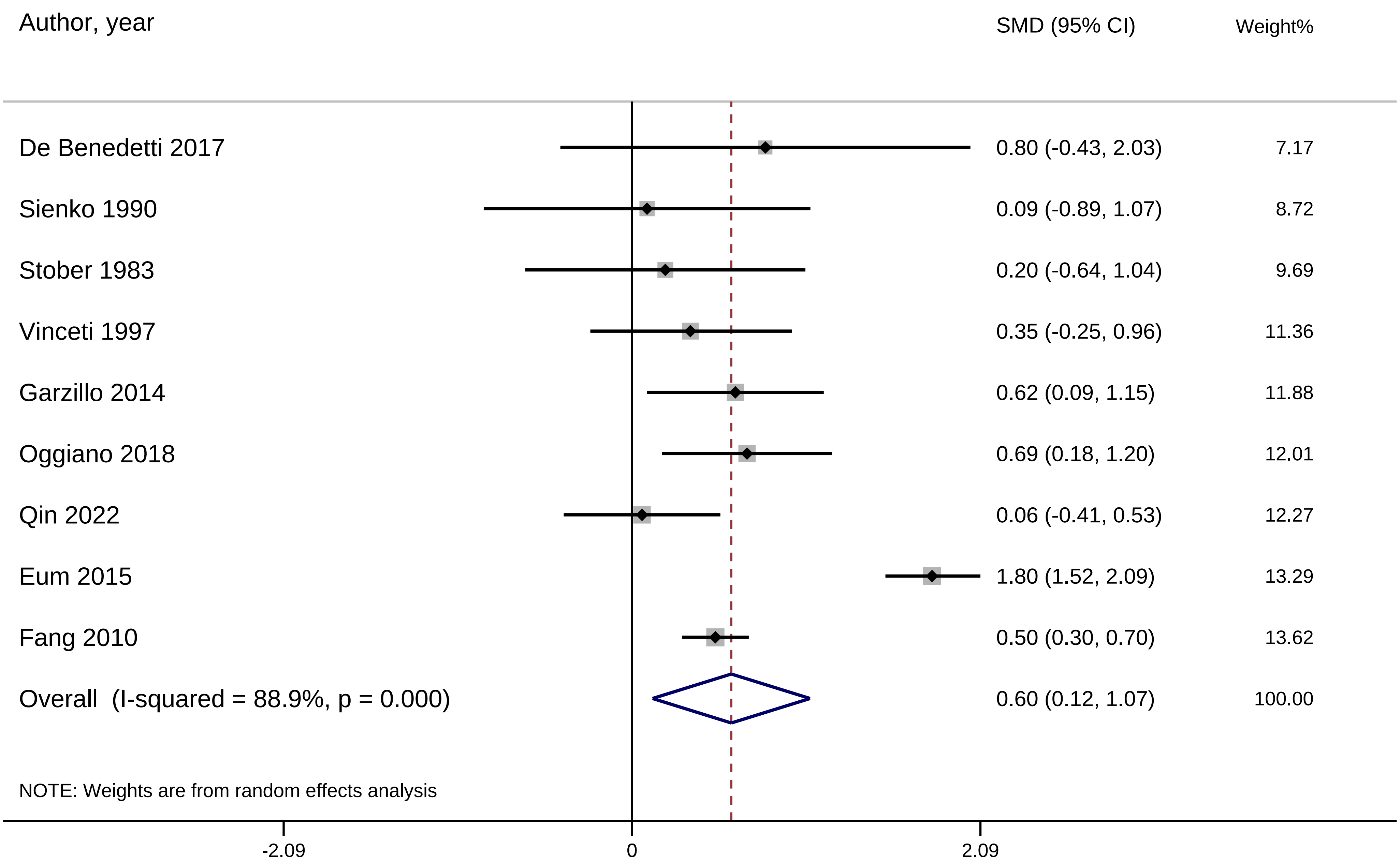

Figure S68. Random-effects meta-analysis of the association between serum lead level and ALS

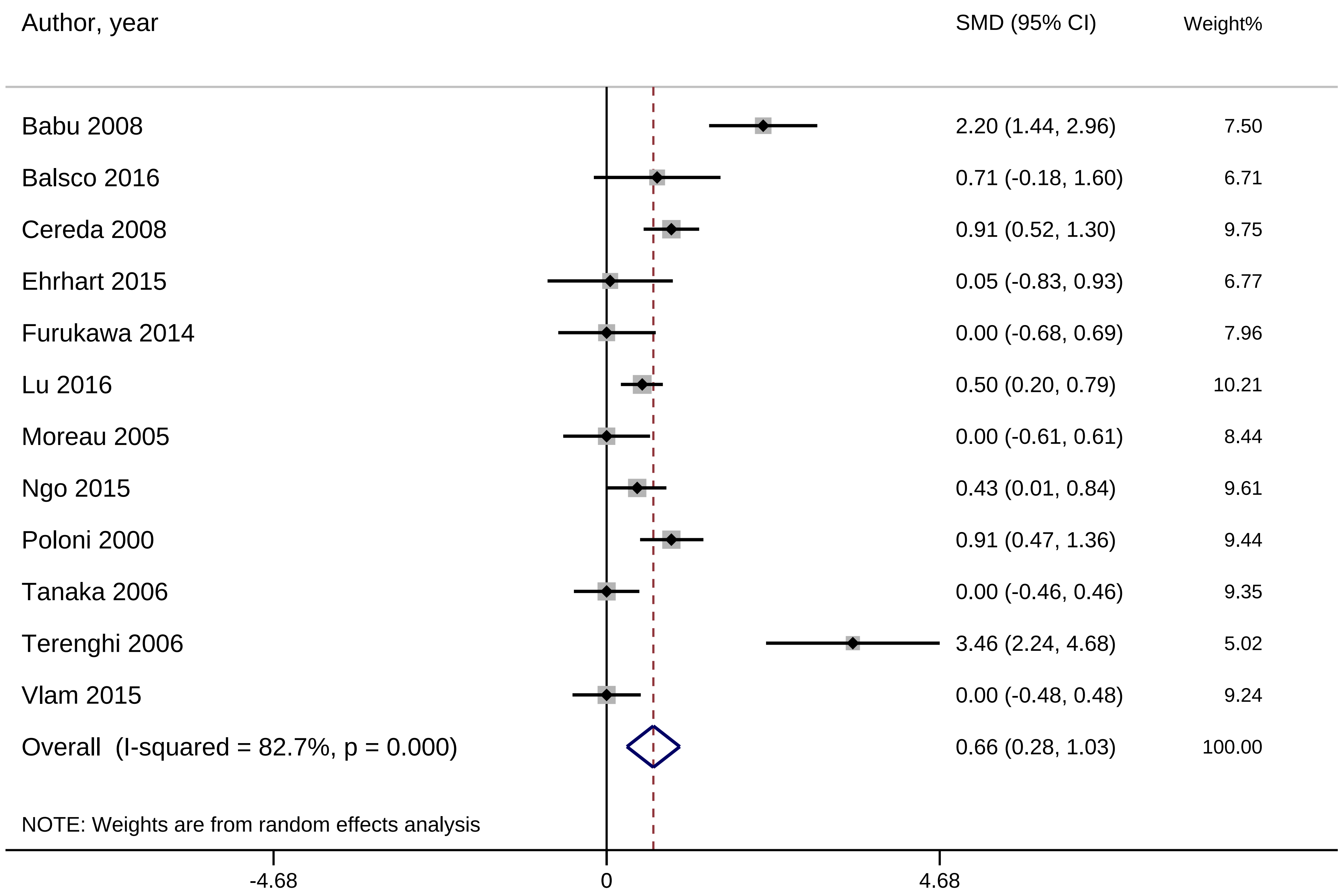

Figure S69. Random-effects meta-analysis of the association between serum TNF- α level and ALS

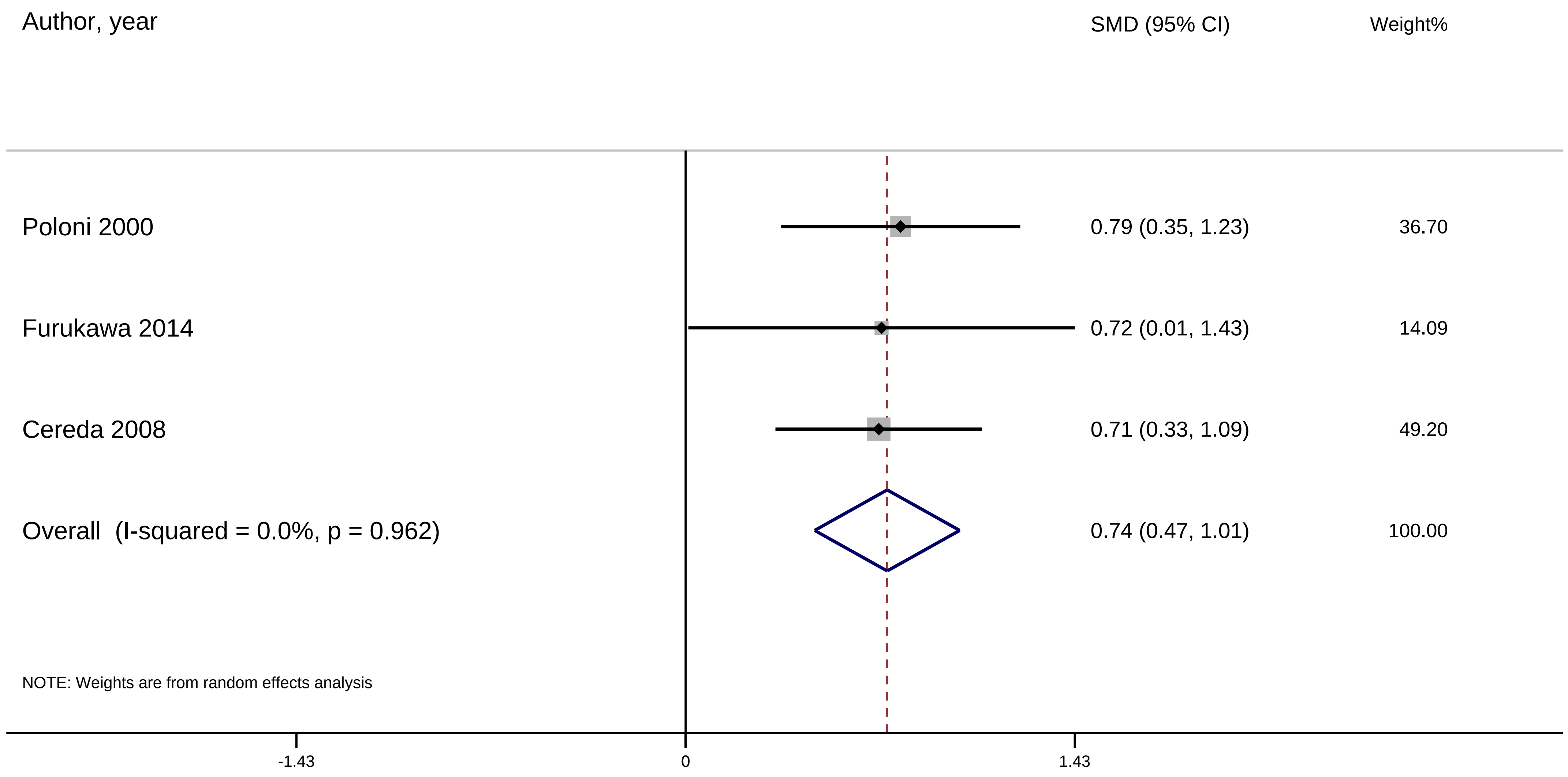

Figure S70. Random-effects meta-analysis of the association between serum TNFR1 level and ALS

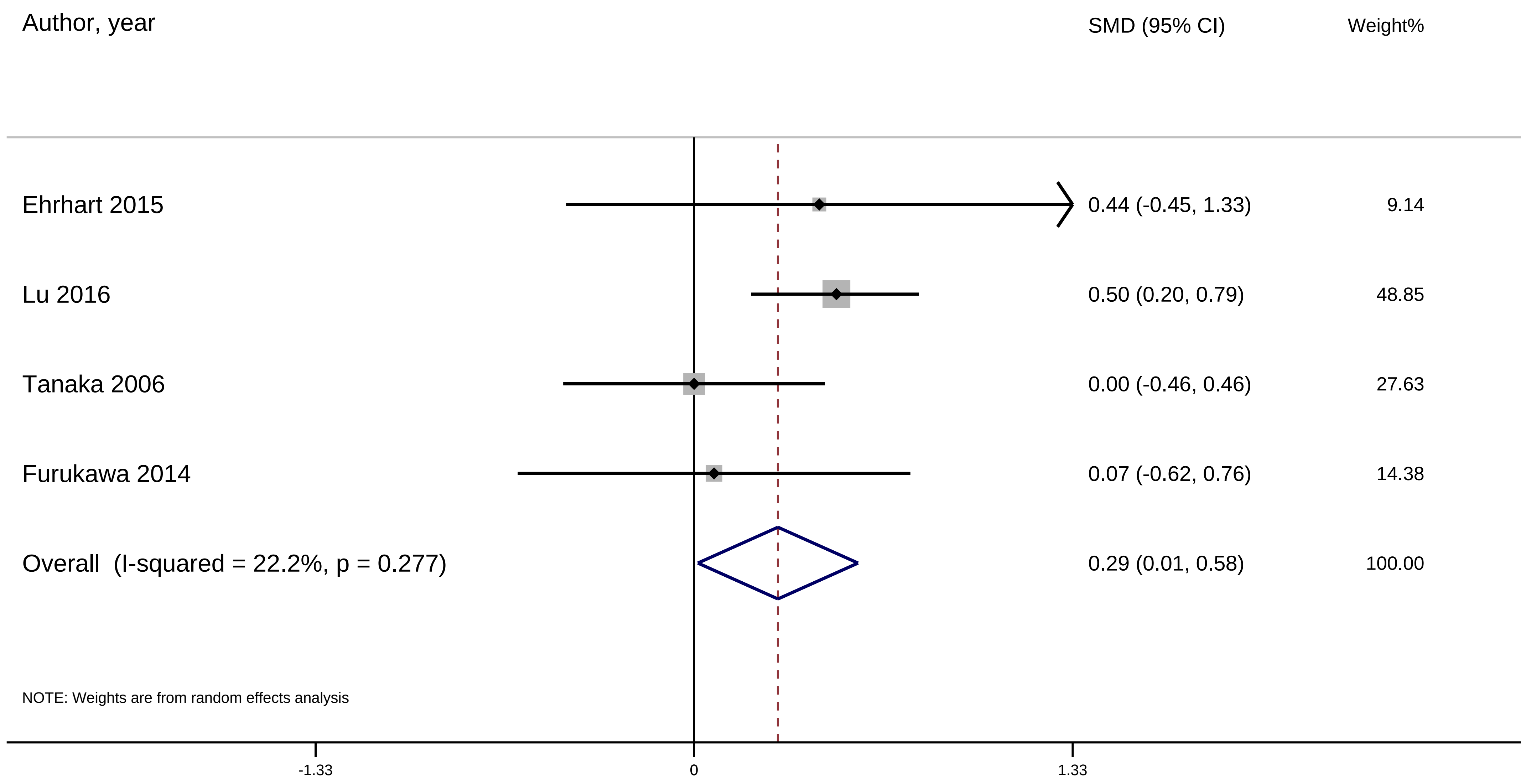

Figure S71. Random-effects meta-analysis of the association between serum IL-1 β level and ALS

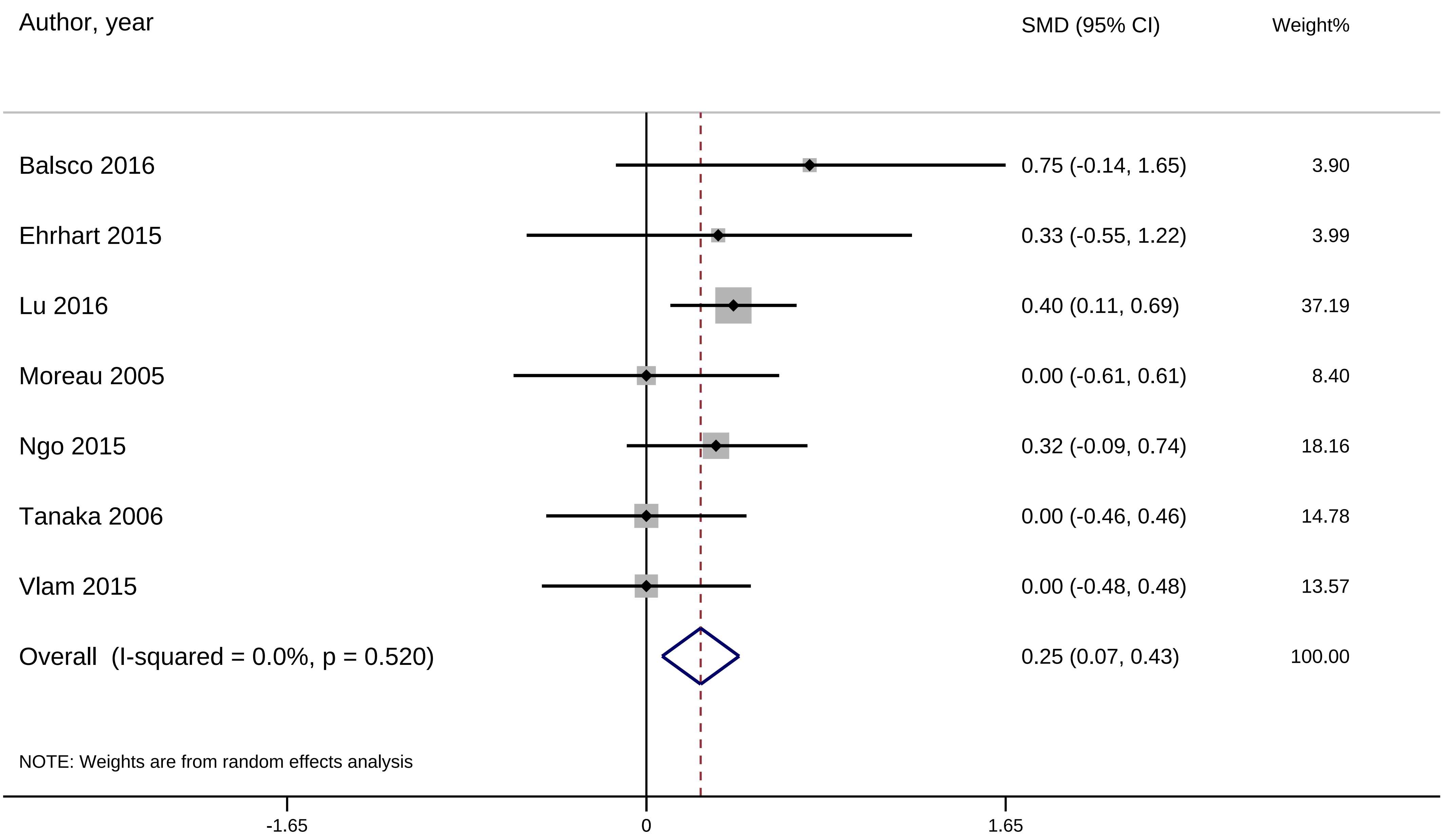

Figure S72. Random-effects meta-analysis of the association between serum IL-6 level and ALS

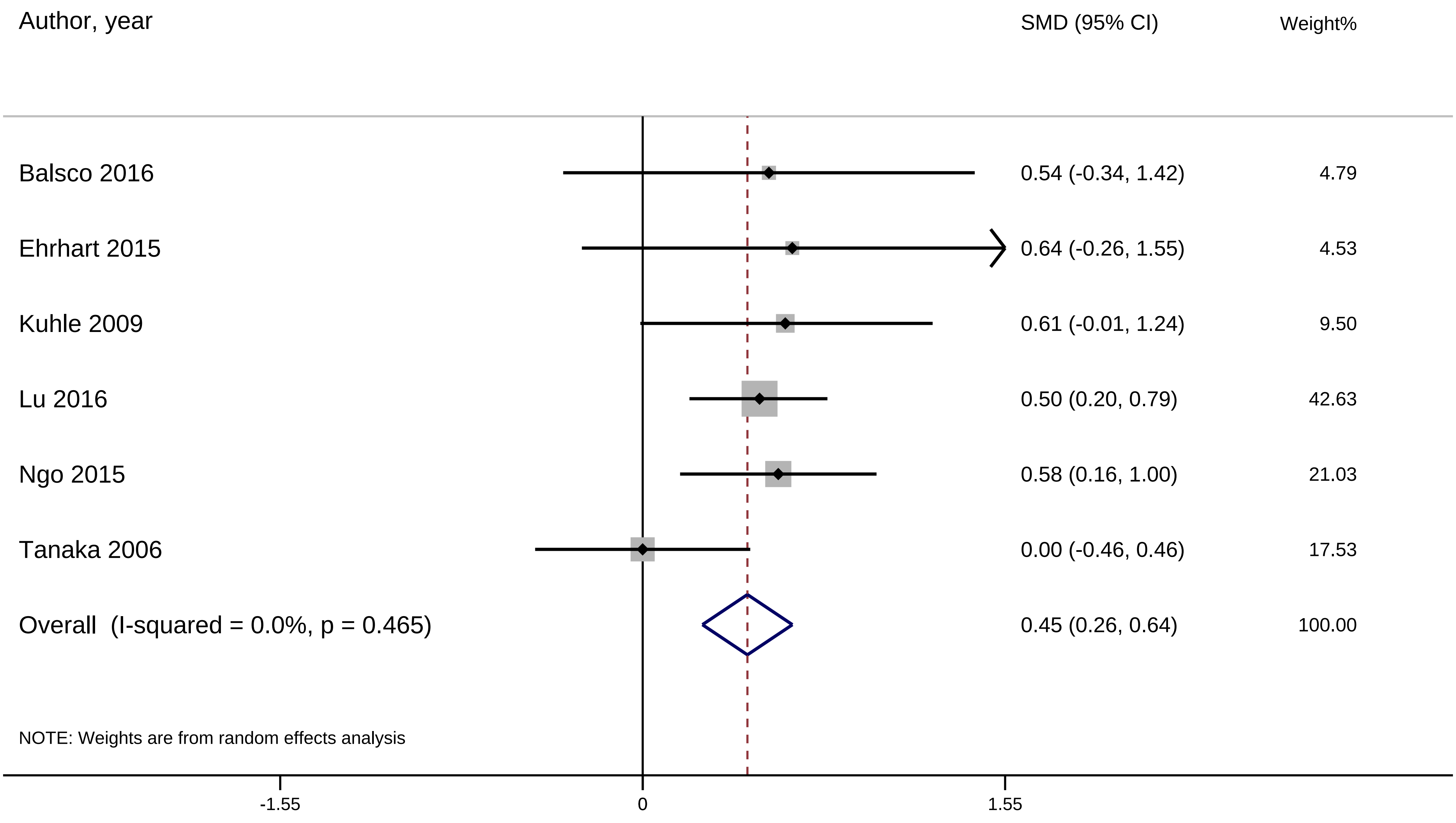

Figure S73. Random-effects meta-analysis of the association between serum IL-8 level and ALS

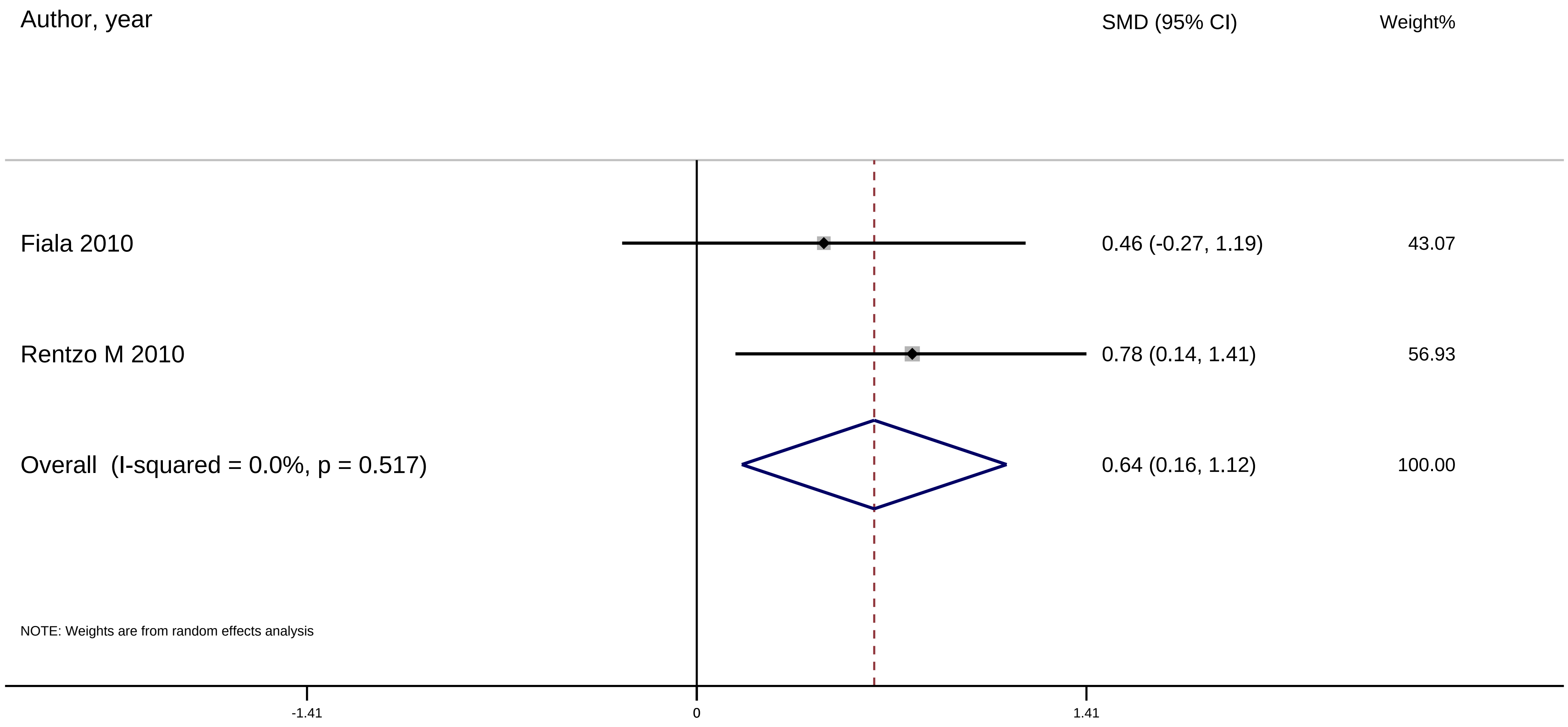

Figure S74. Random-effects meta-analysis of the association between serum IL-17 level and ALS

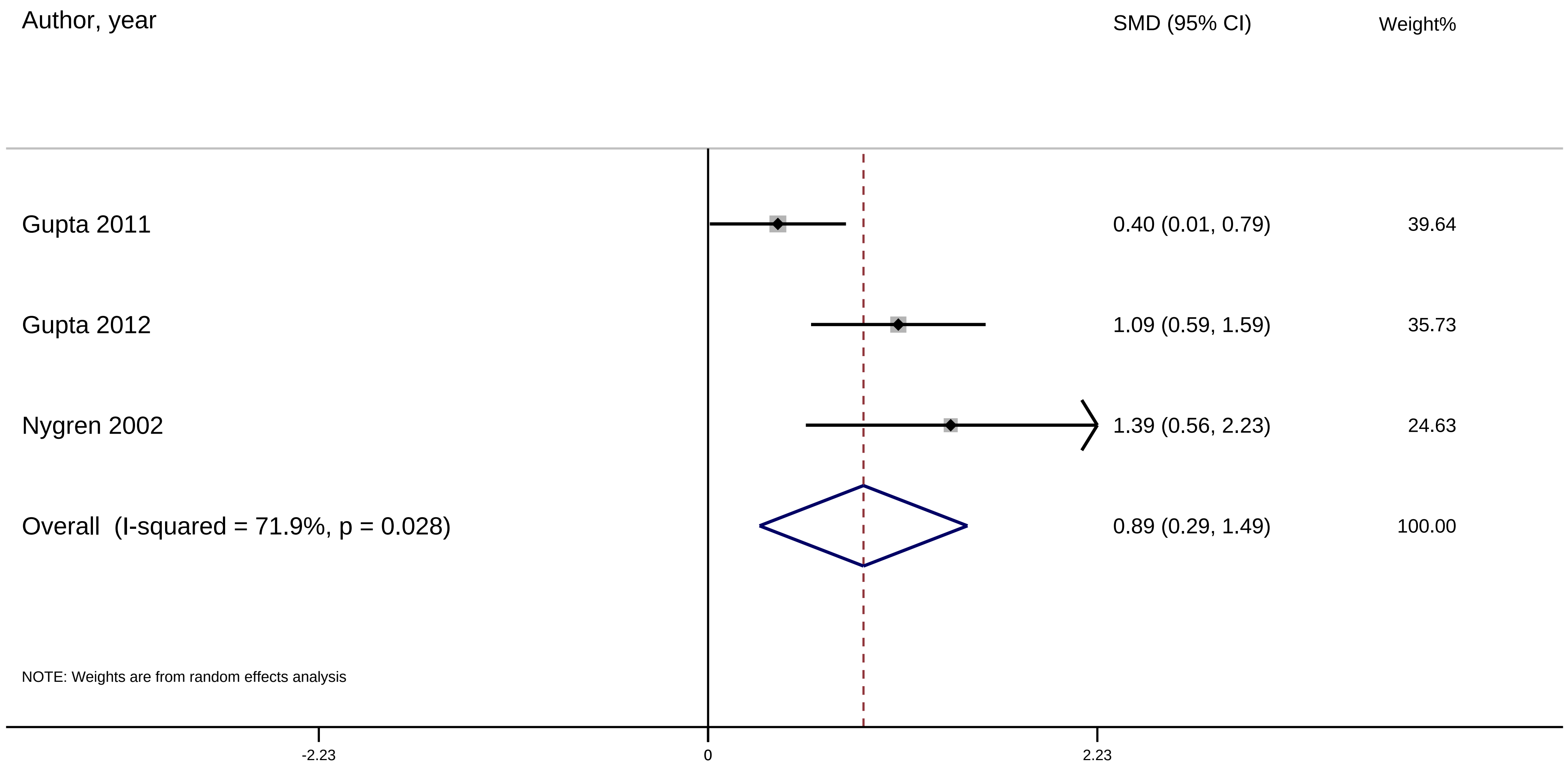

Figure S75. Random-effects meta-analysis of the association between serum VEGF level and ALS

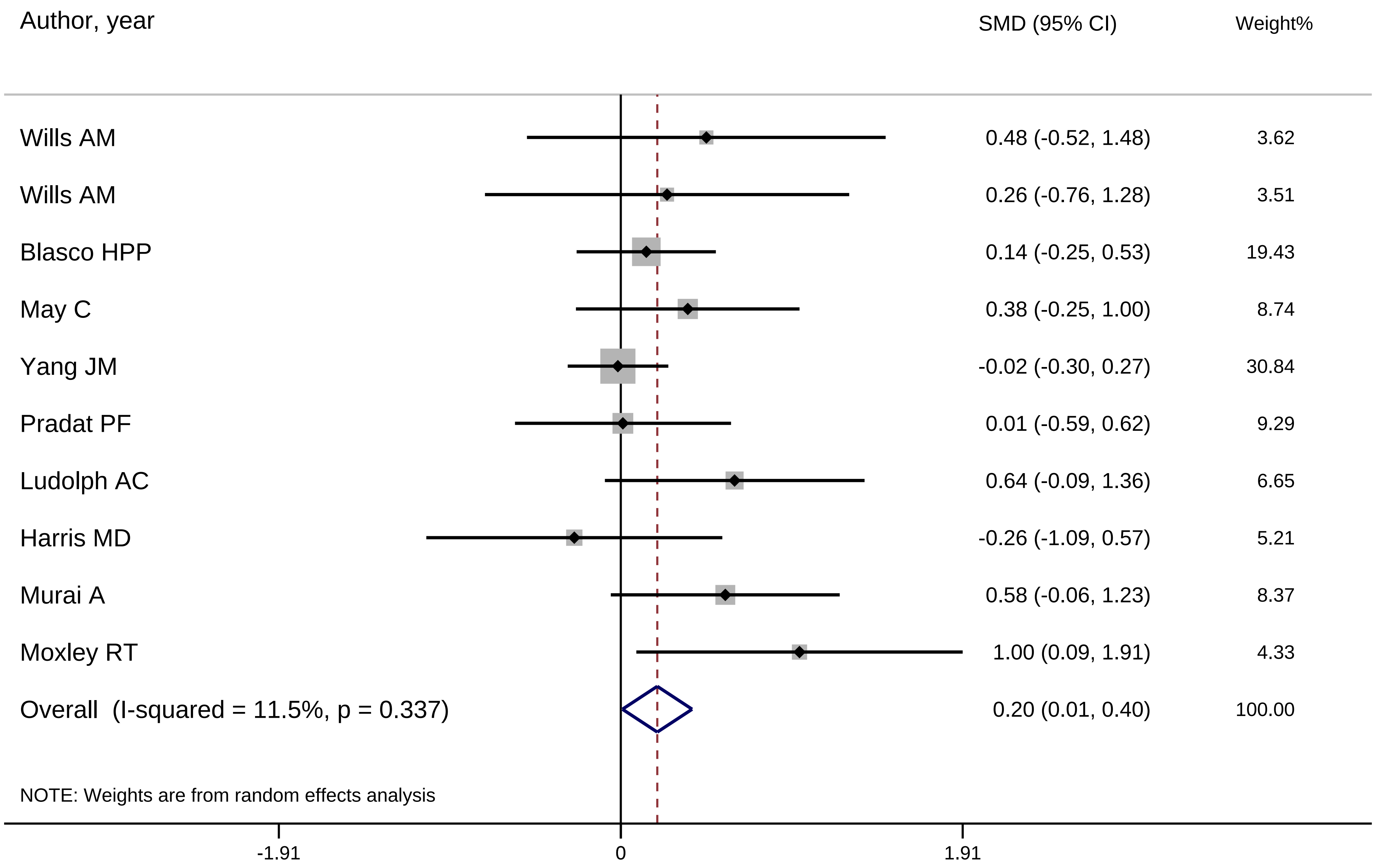

Figure S76. Random-effects meta-analysis of the association between serum FBG level and ALS

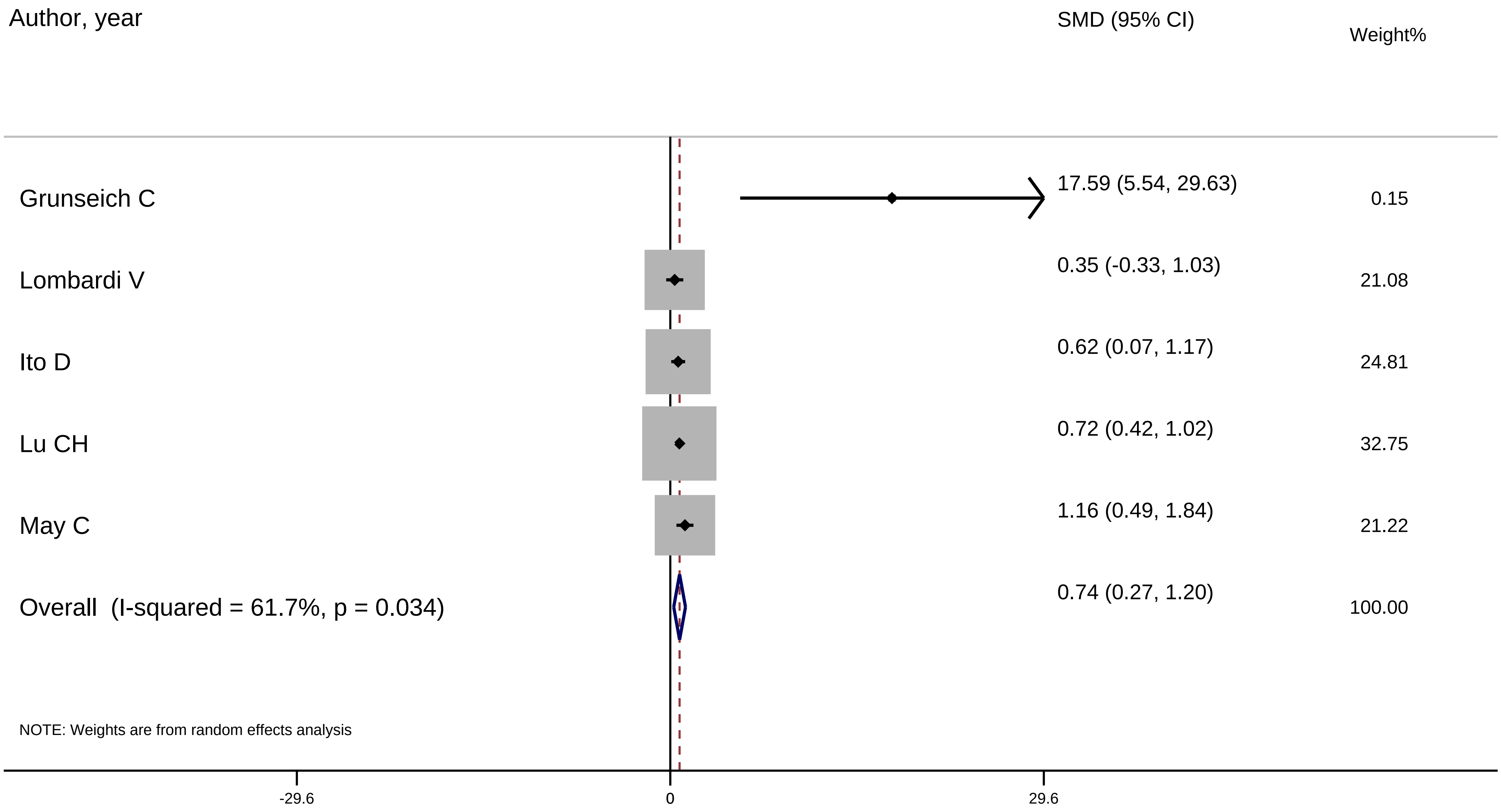

Figure S77. Random-effects meta-analysis of the association between serum CK level and ALS

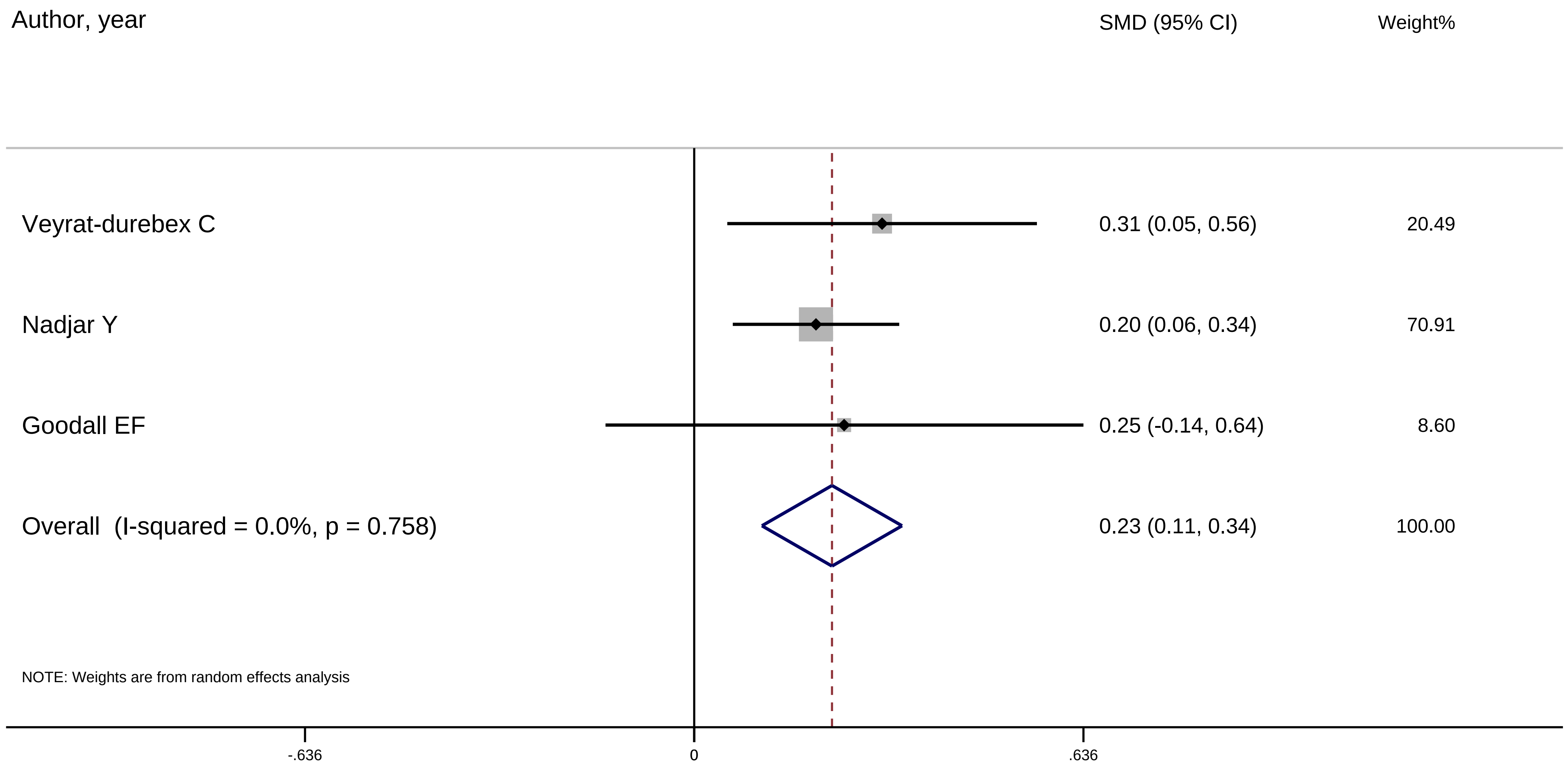

Figure S78. Random-effects meta-analysis of the association between serum TSC level and ALS

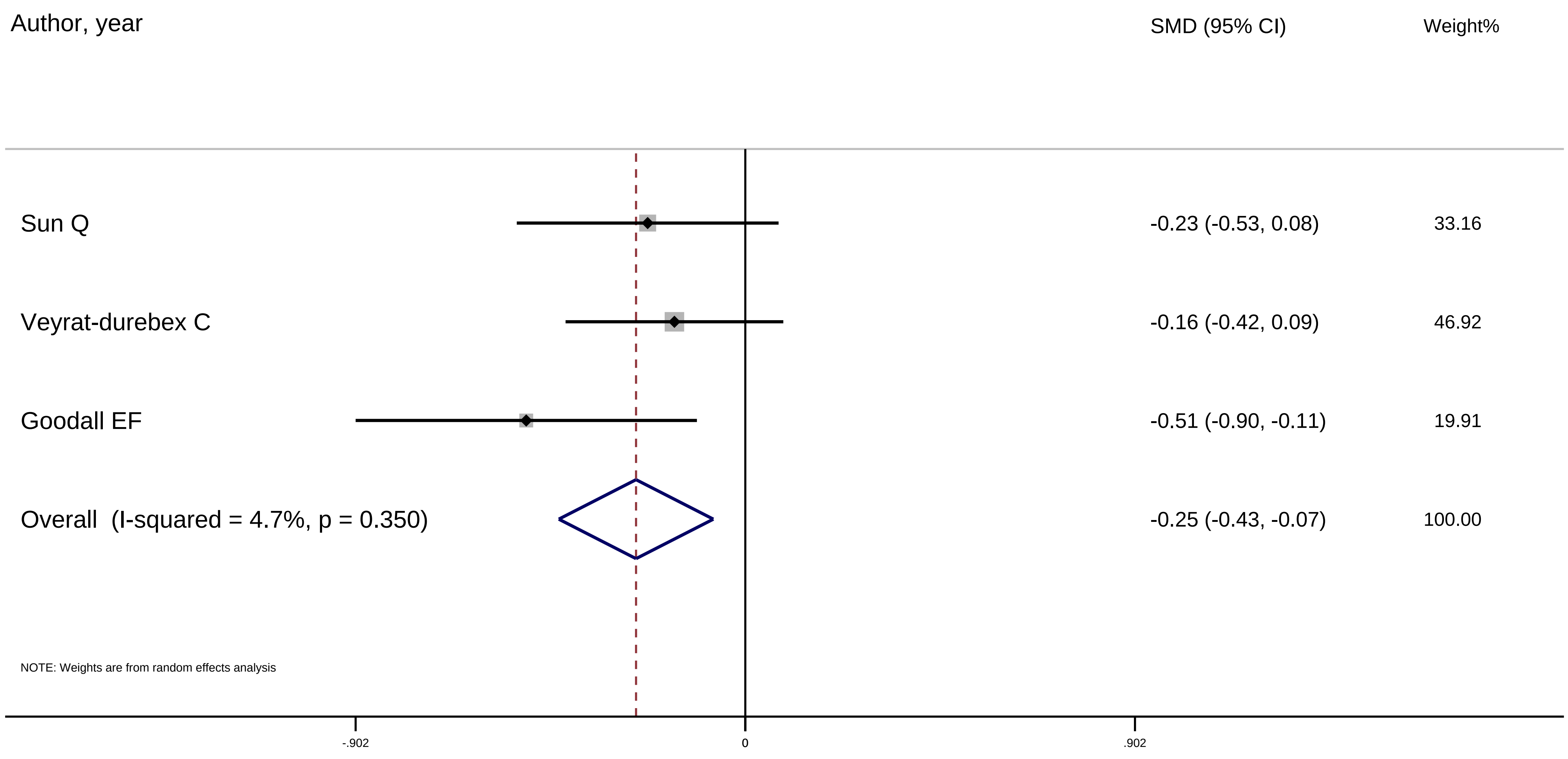

Figure S79. Random-effects meta-analysis of the association between serum TIBC level and ALS

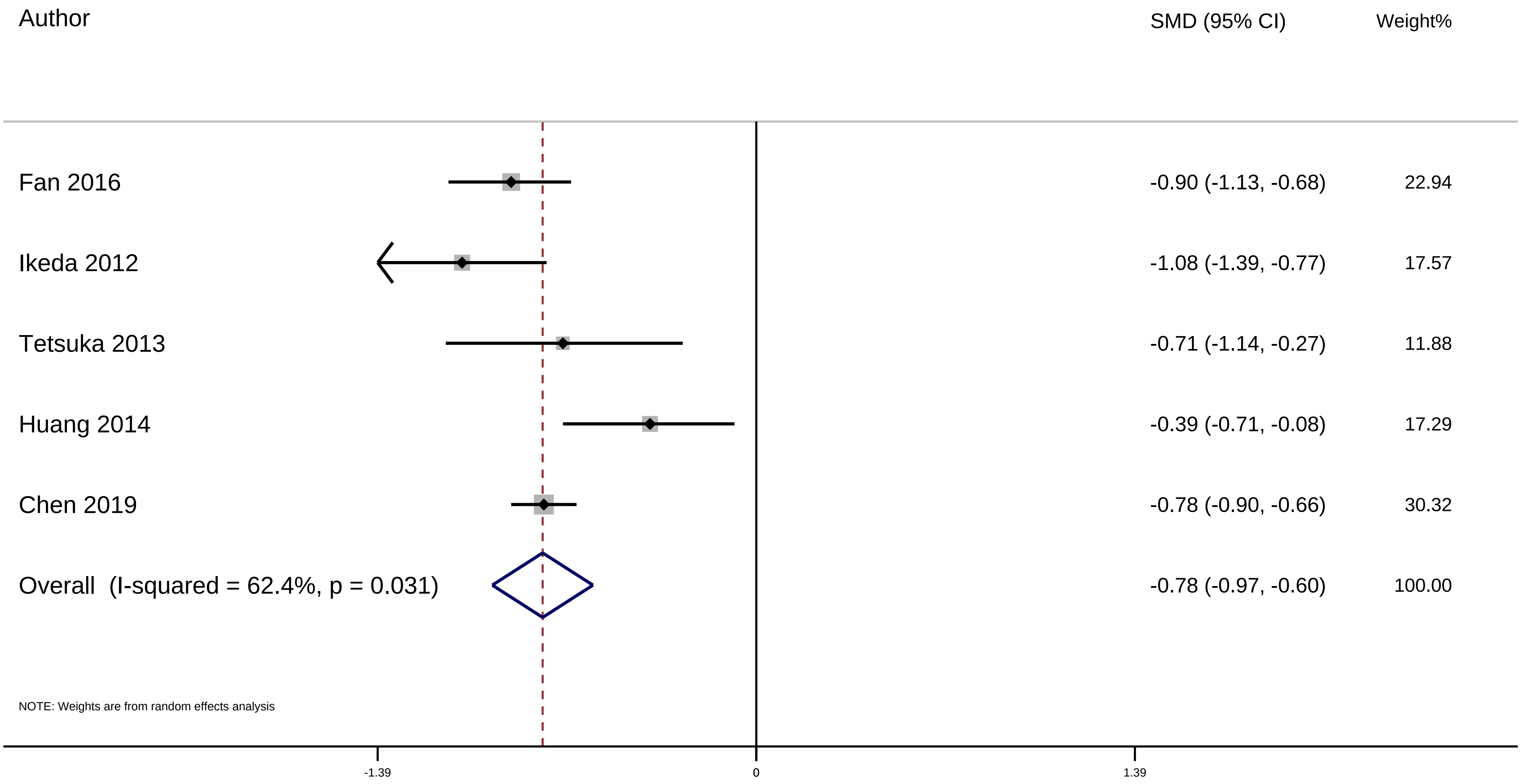

Figure S80. Random-effects meta-analysis of the association between serum creatinine level and ALS

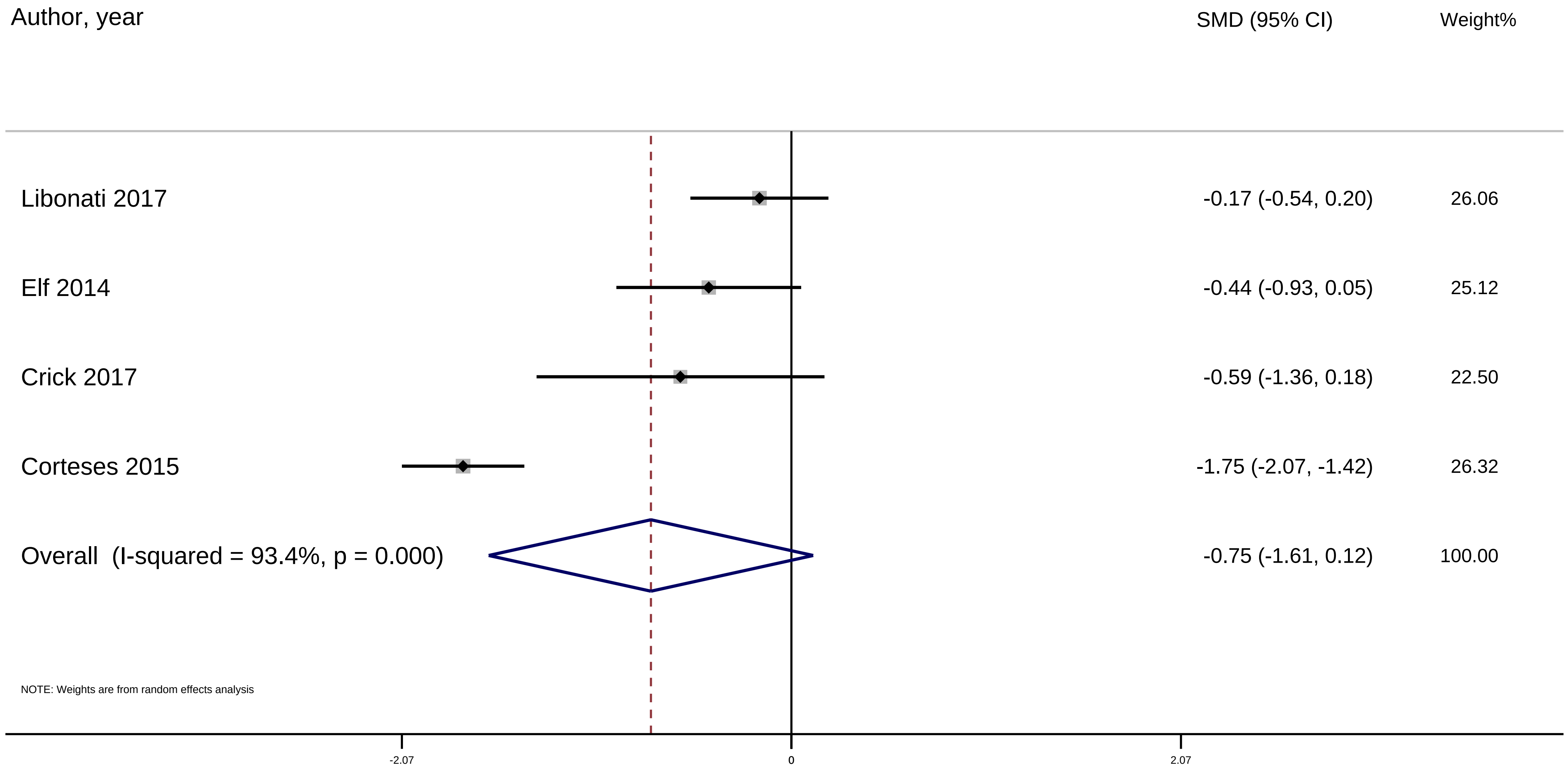

Figure S81. Random-effects meta-analysis of the association between serum vitamin D level and ALS

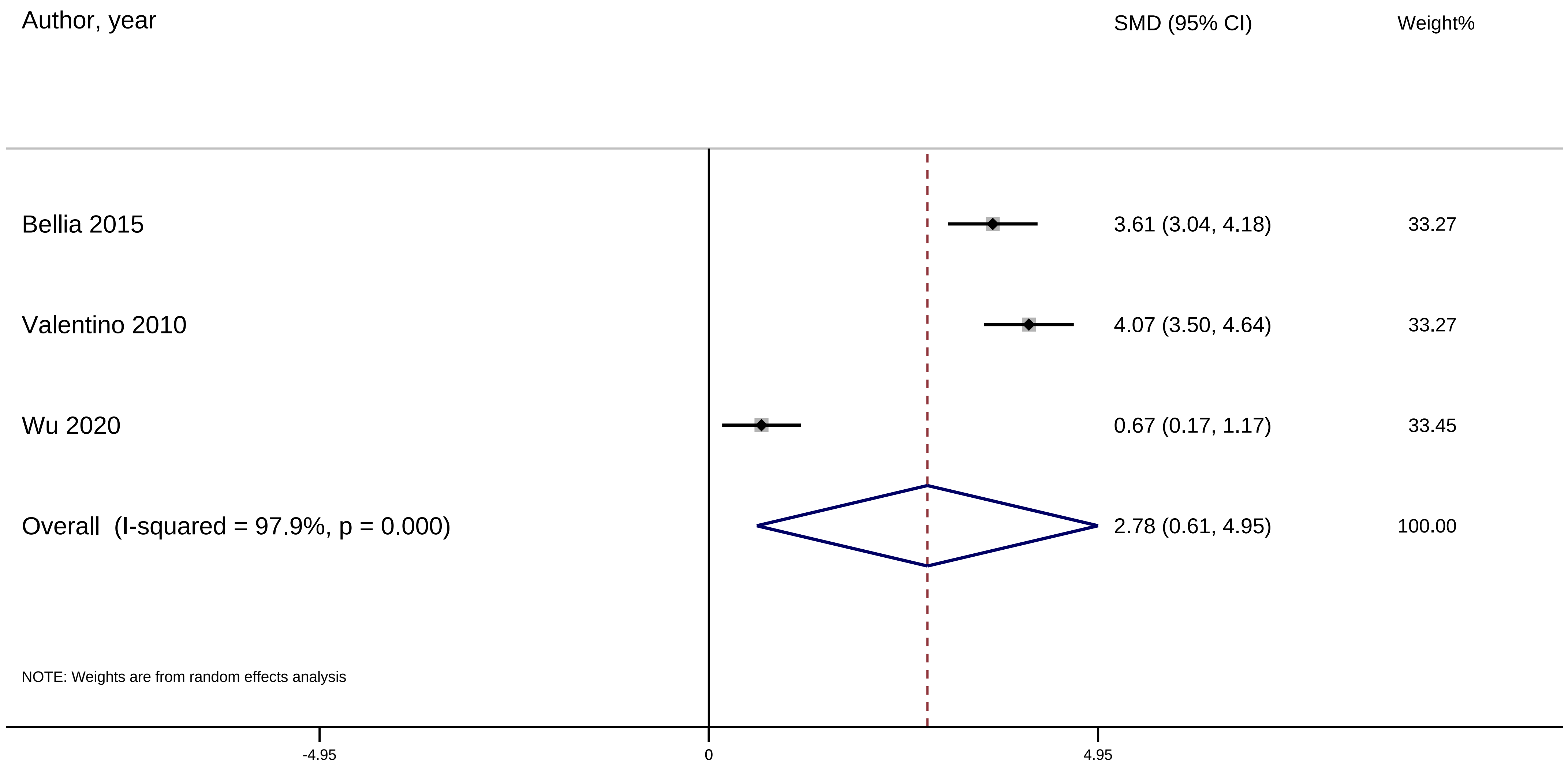

Figure S82. Random-effects meta-analysis of the association between serum folic level and ALS

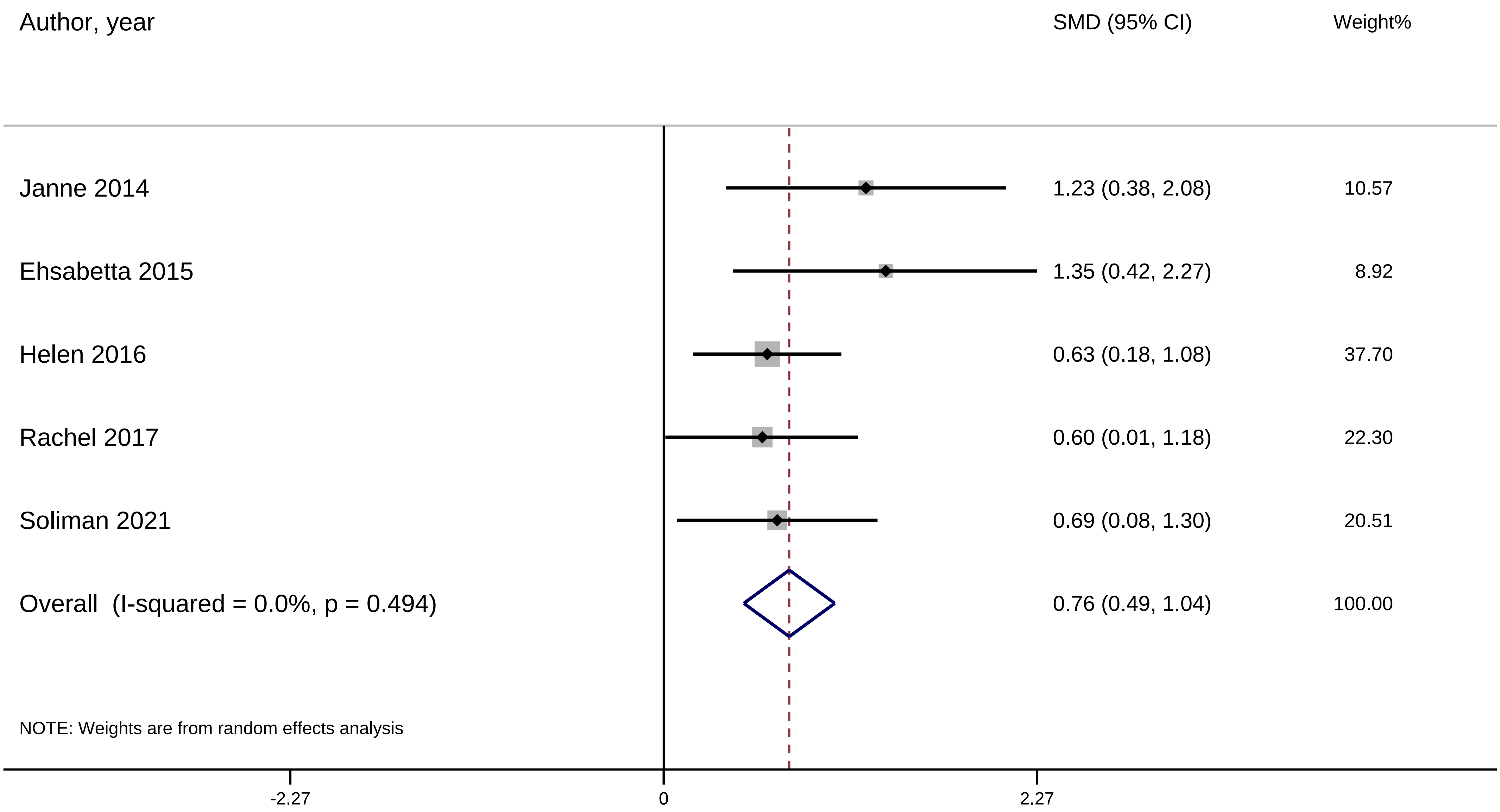

Figure S83. Random-effects meta-analysis of the association between serum miR-206 level and ALS

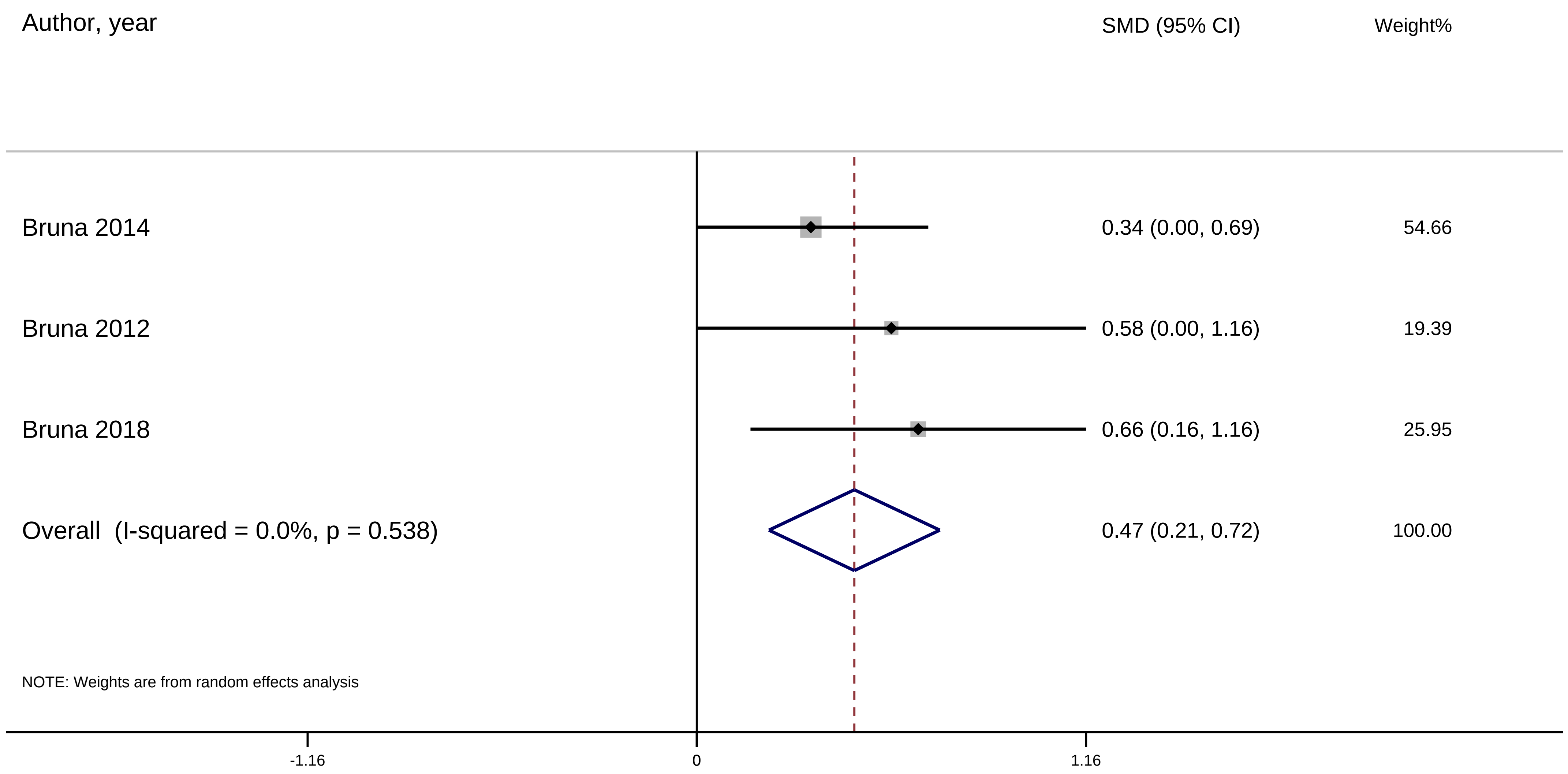

Figure S84. Random-effects meta-analysis of the association between serum miR-338-3p level and ALS

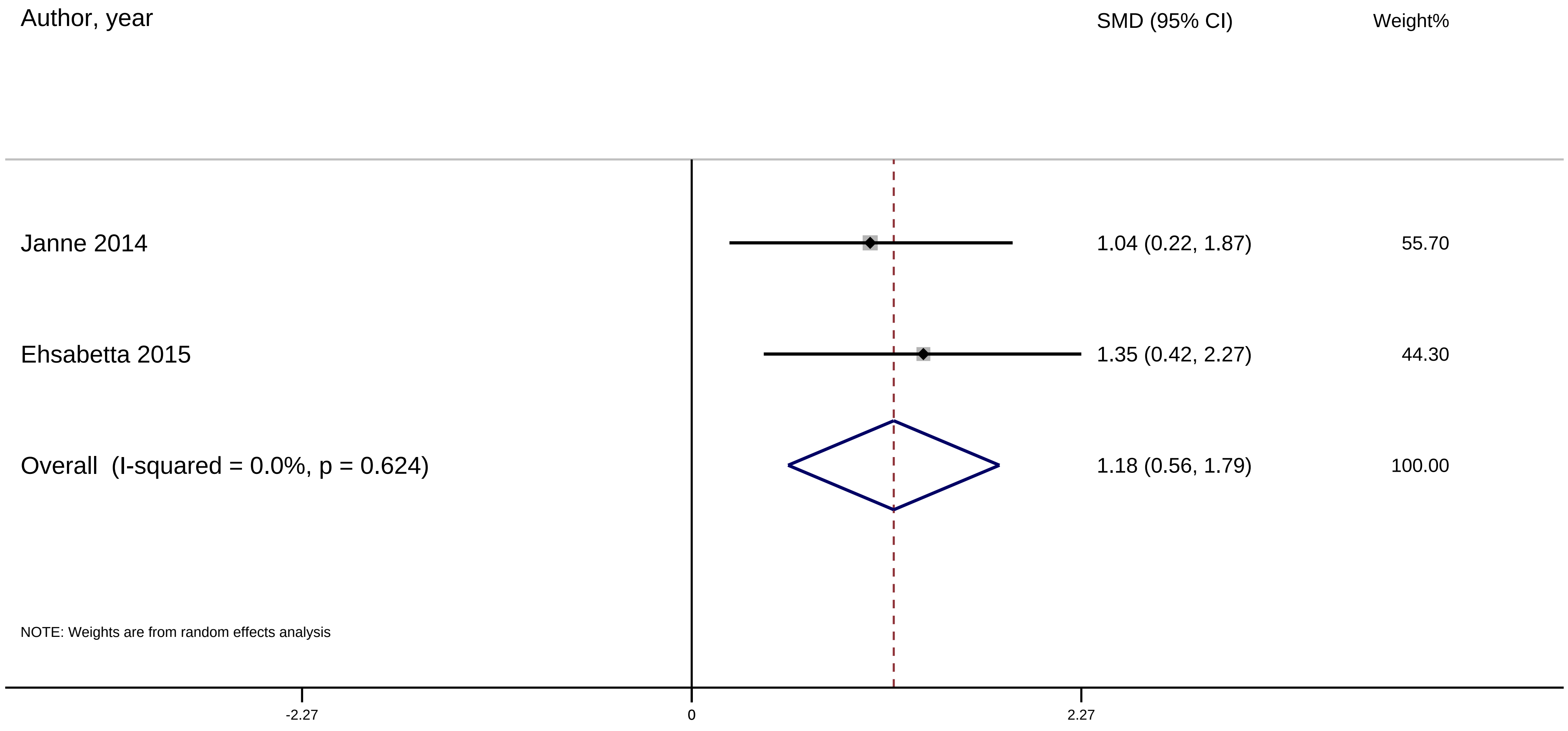

Figure S85. Random-effects meta-analysis of the association between serum miR-133b level and ALS

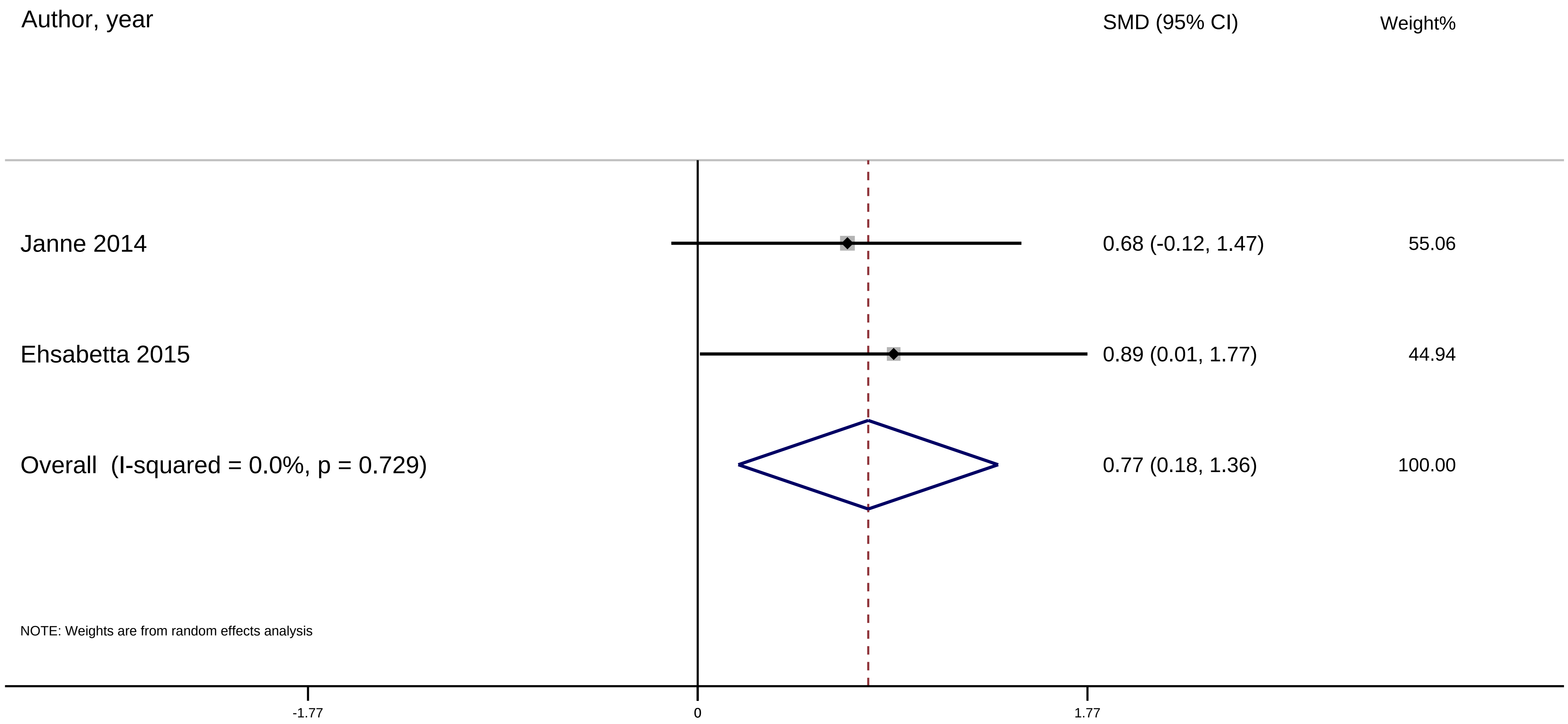

Figure S86. Random-effects meta-analysis of the association between serum miR-133a level and ALS

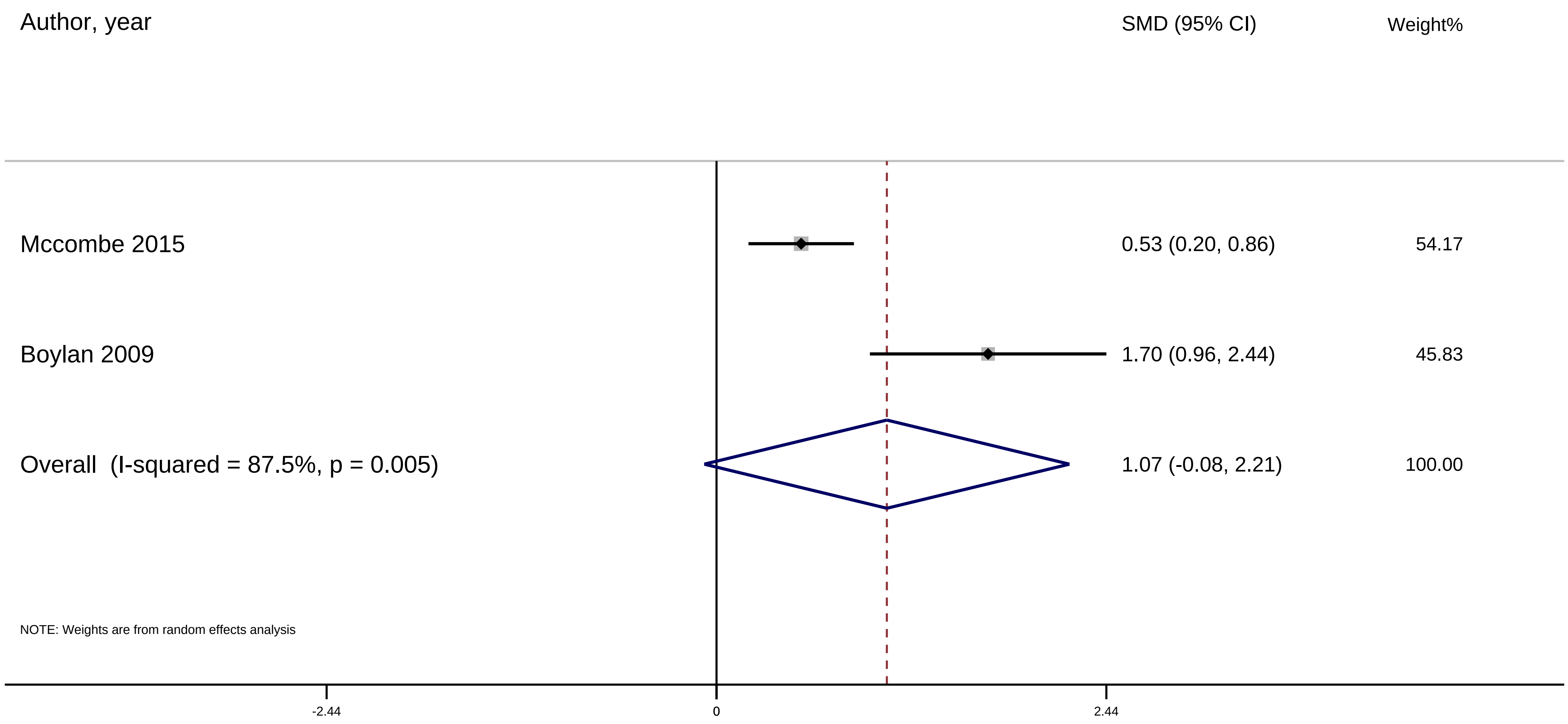

Figure S87. Random-effects meta-analysis of the association between serum NFH level and ALS

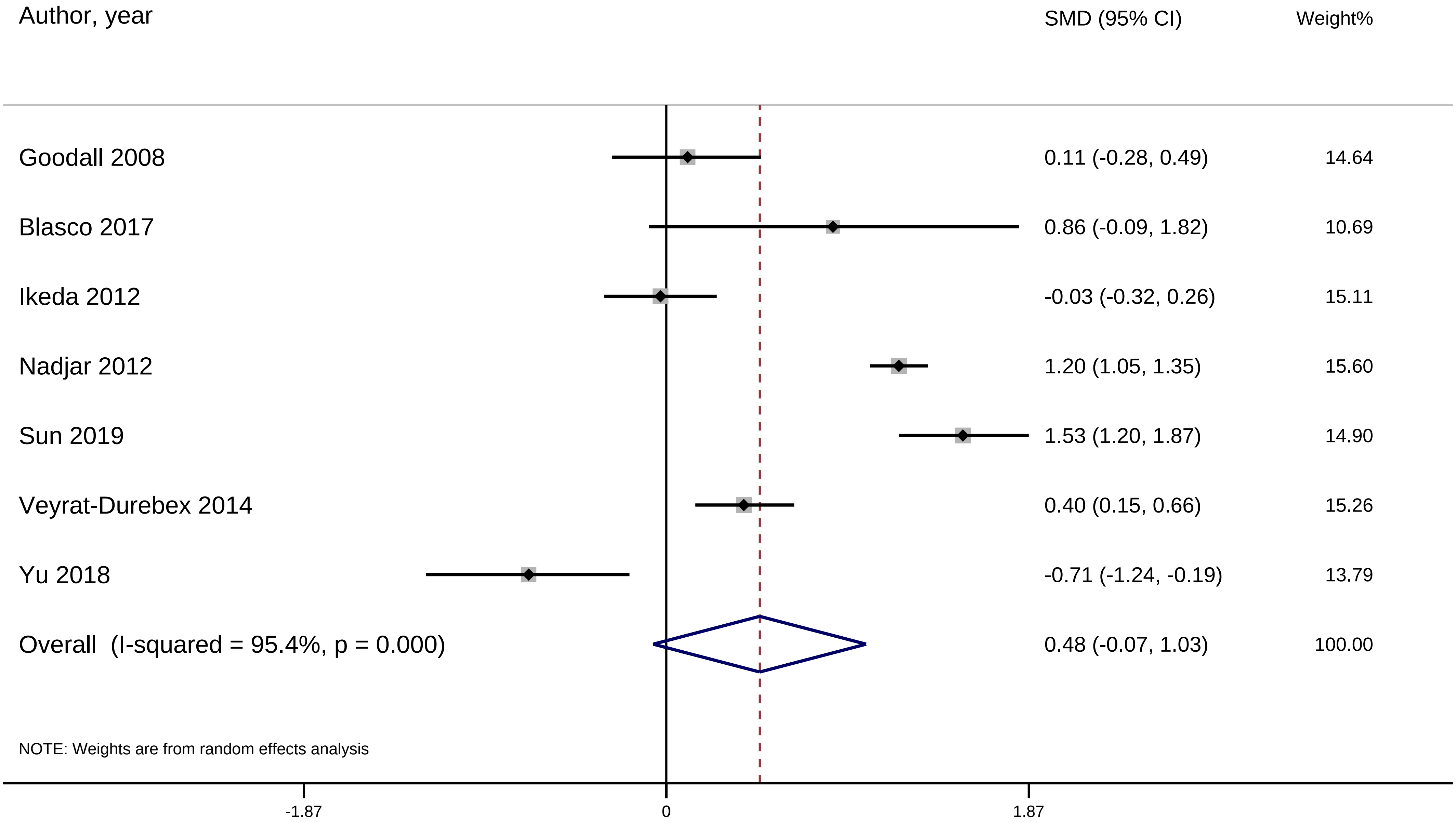

Figure S88. Random-effects meta-analysis of the association between serum iron level and ALS

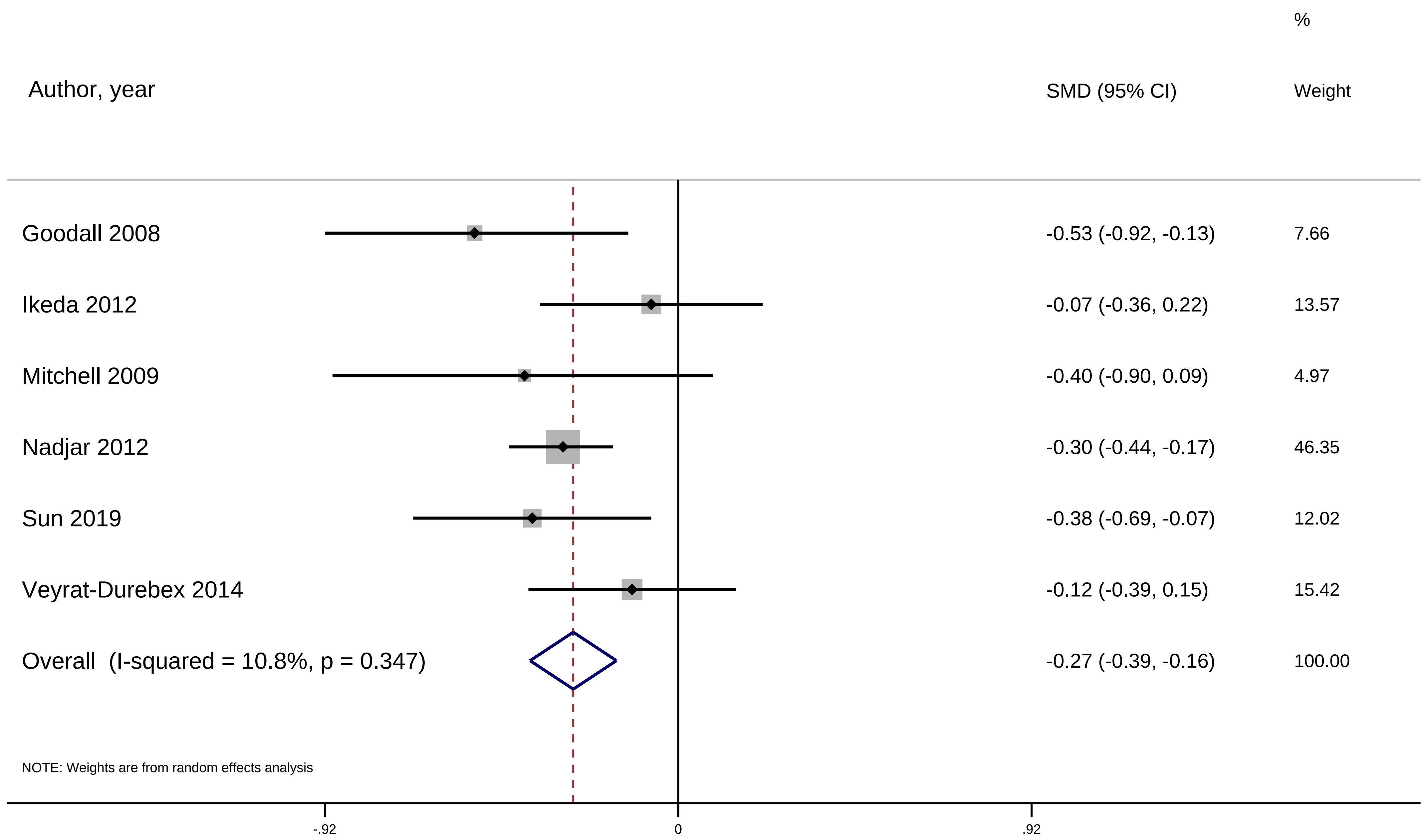

Figure S89. Random-effects meta-analysis of the association between serum transferrin level and ALS

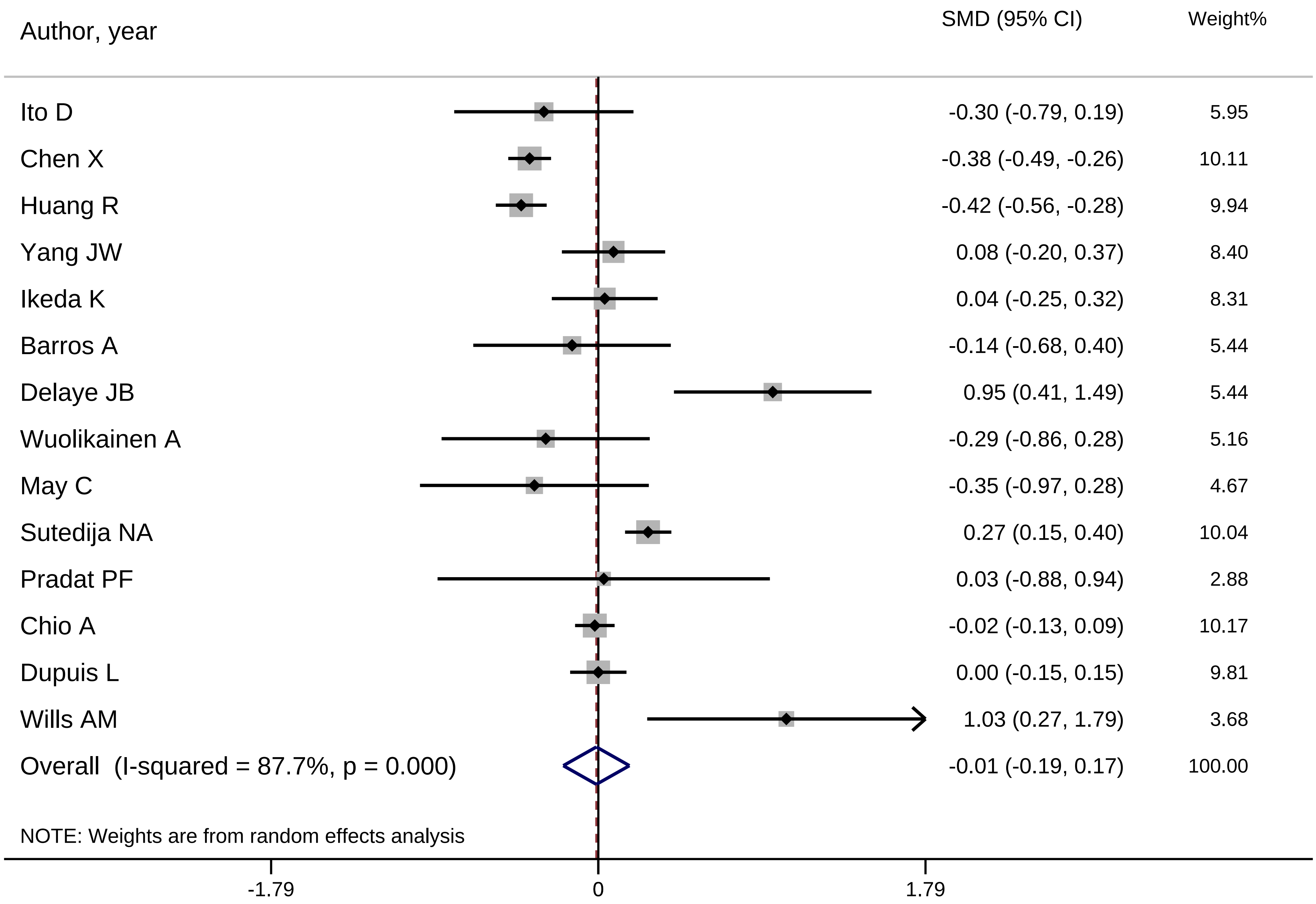

Figure S90. Random-effects meta-analysis of the association between serum HDL level and ALS

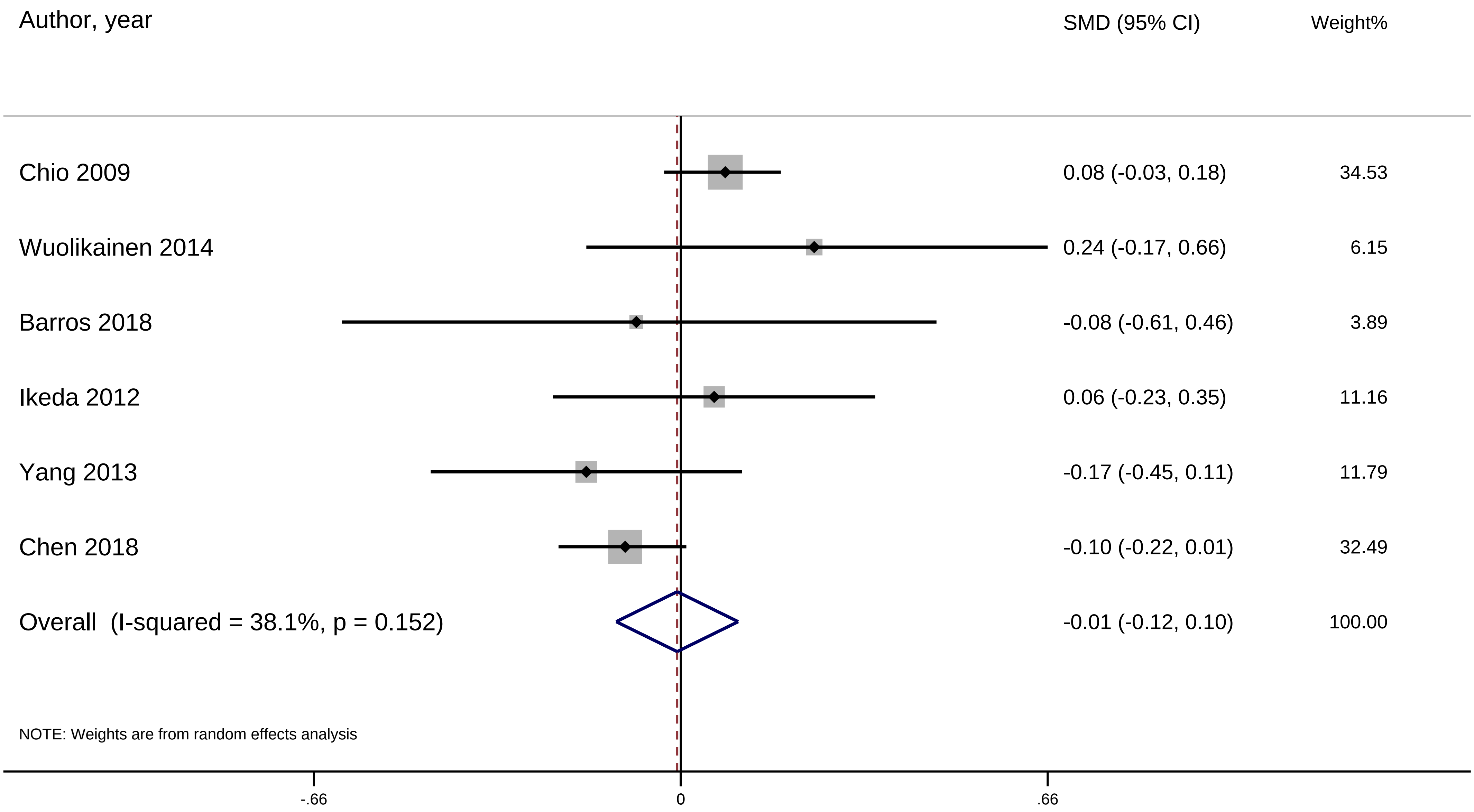

Figure S91. Random-effects meta-analysis of the association between serum LDL level and ALS

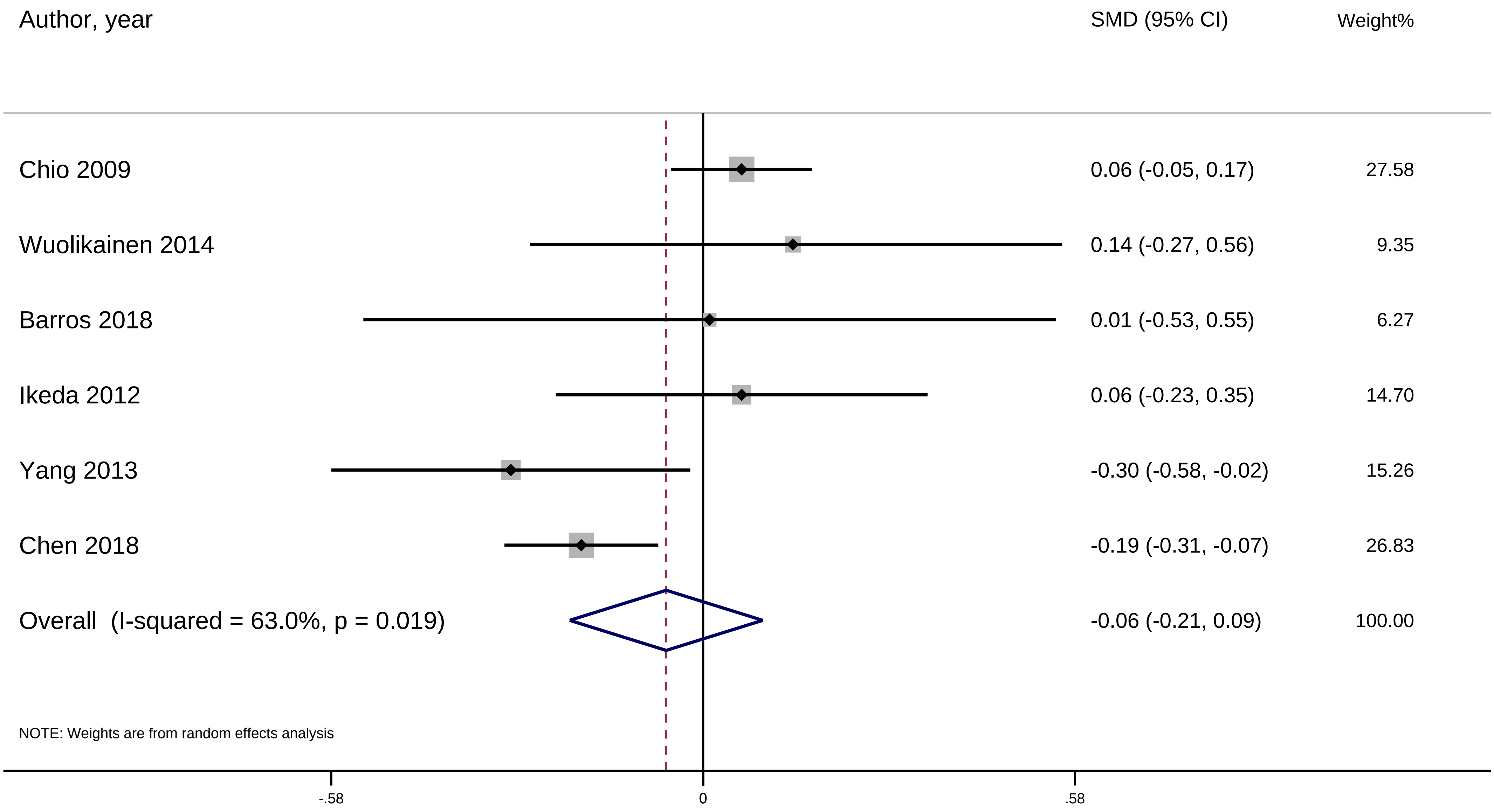

Figure S92. Random-effects meta-analysis of the association between serum TC level and ALS

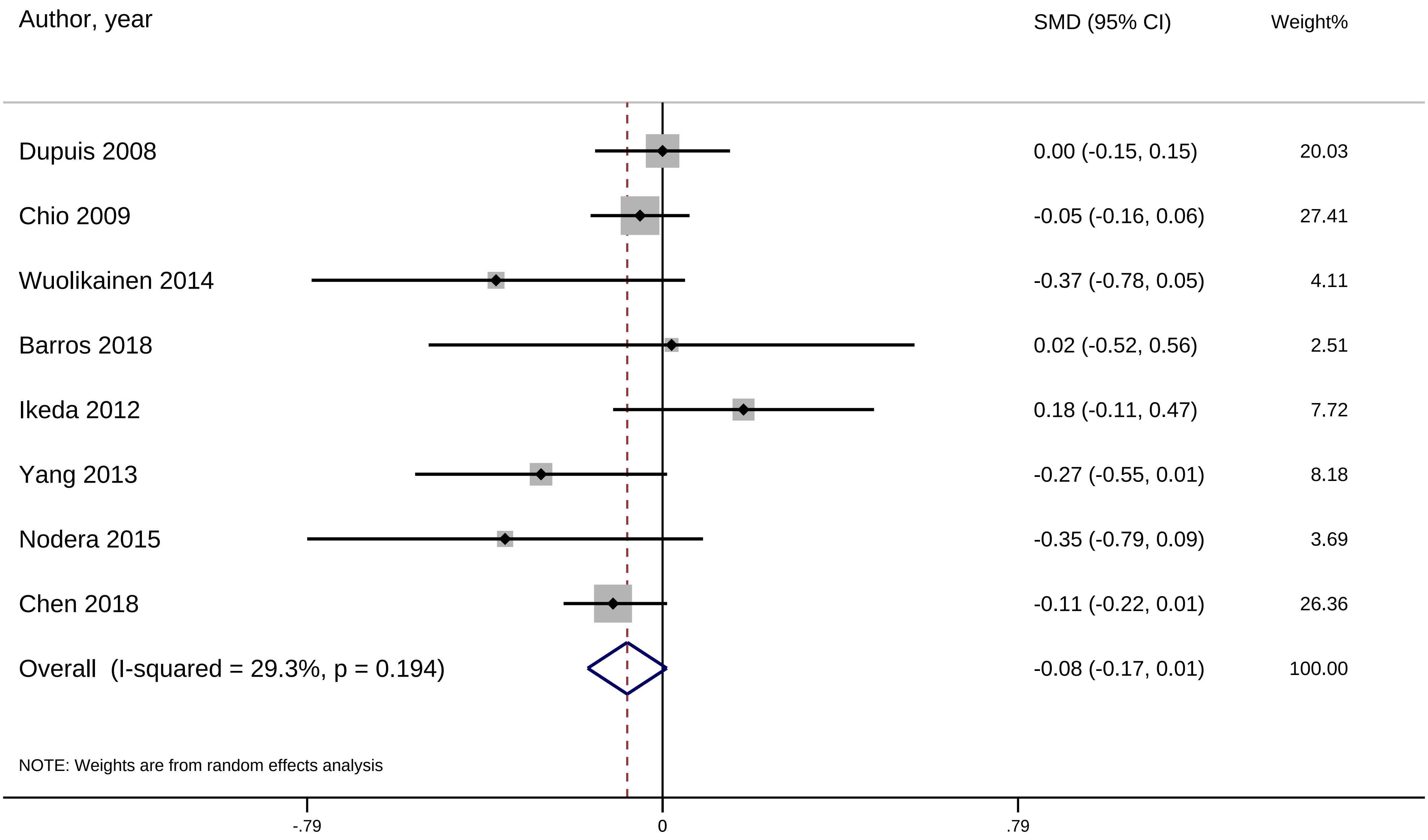

Figure S93. Random-effects meta-analysis of the association between Serum TG level and ALS

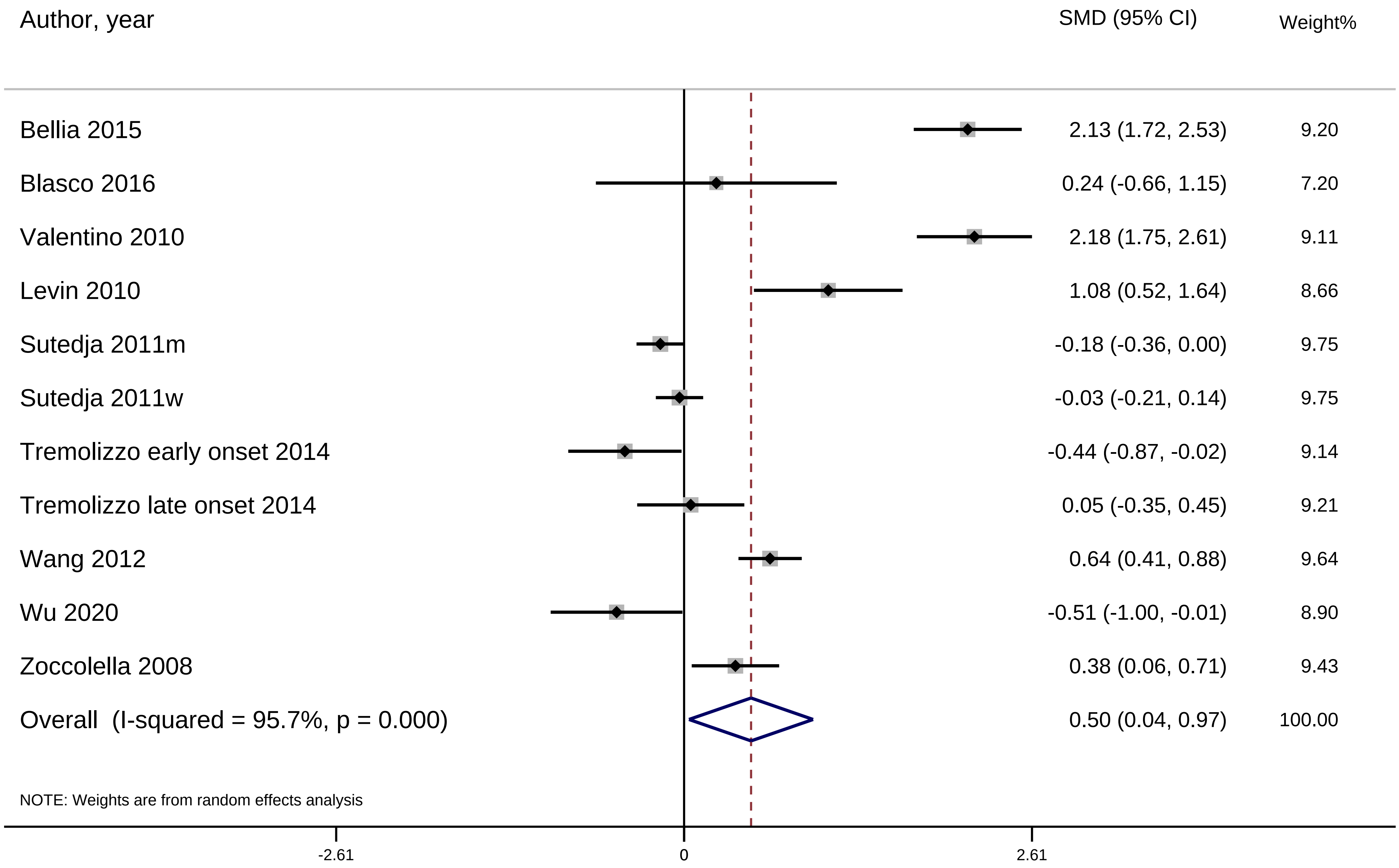

Figure S94. Random-effects meta-analysis of the association between serum homocysteine level and ALS

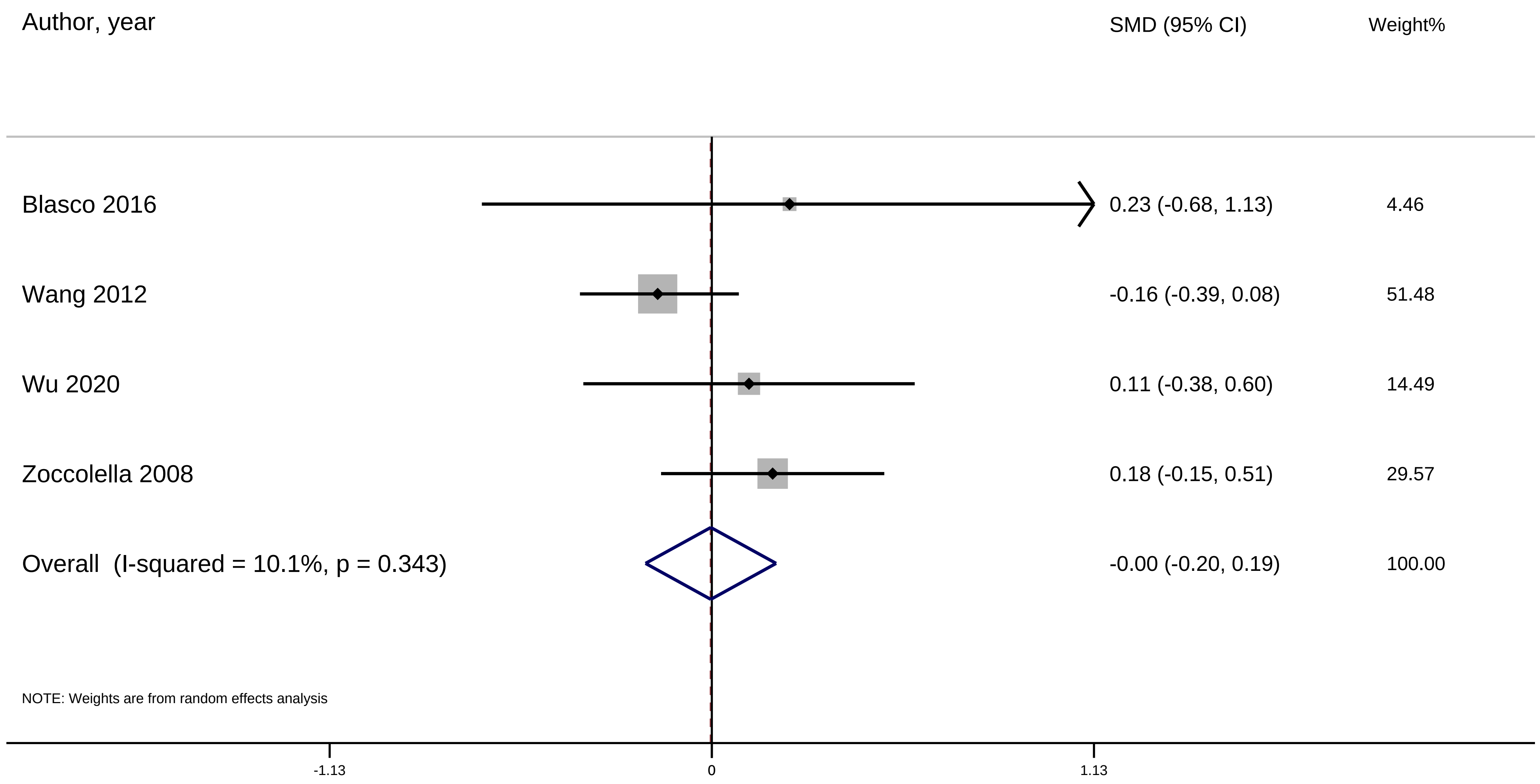

Figure S95. Random-effects meta-analysis of the association between serum vitamin B12 level and ALS

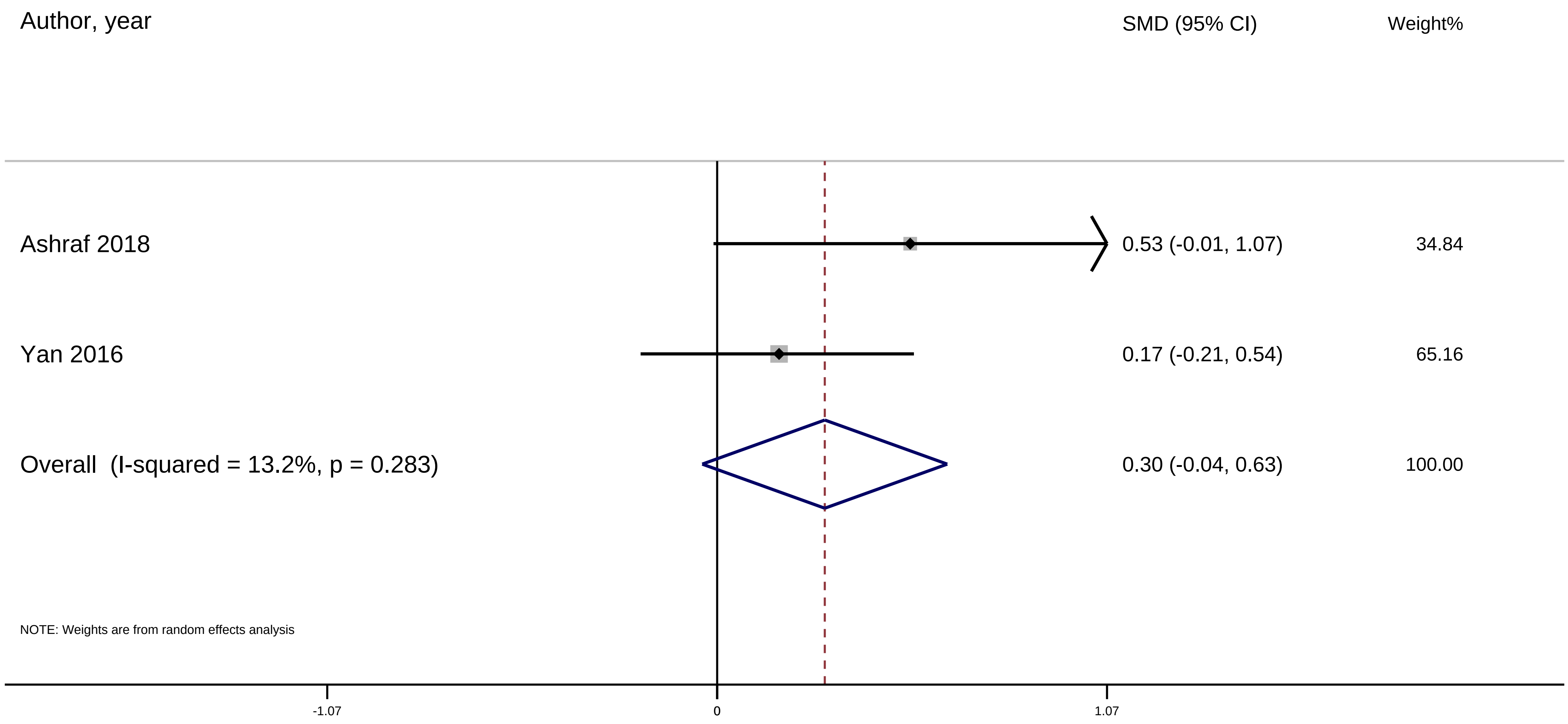

Figure S96. Random-effects meta-analysis of the association between serum galectin level and ALS

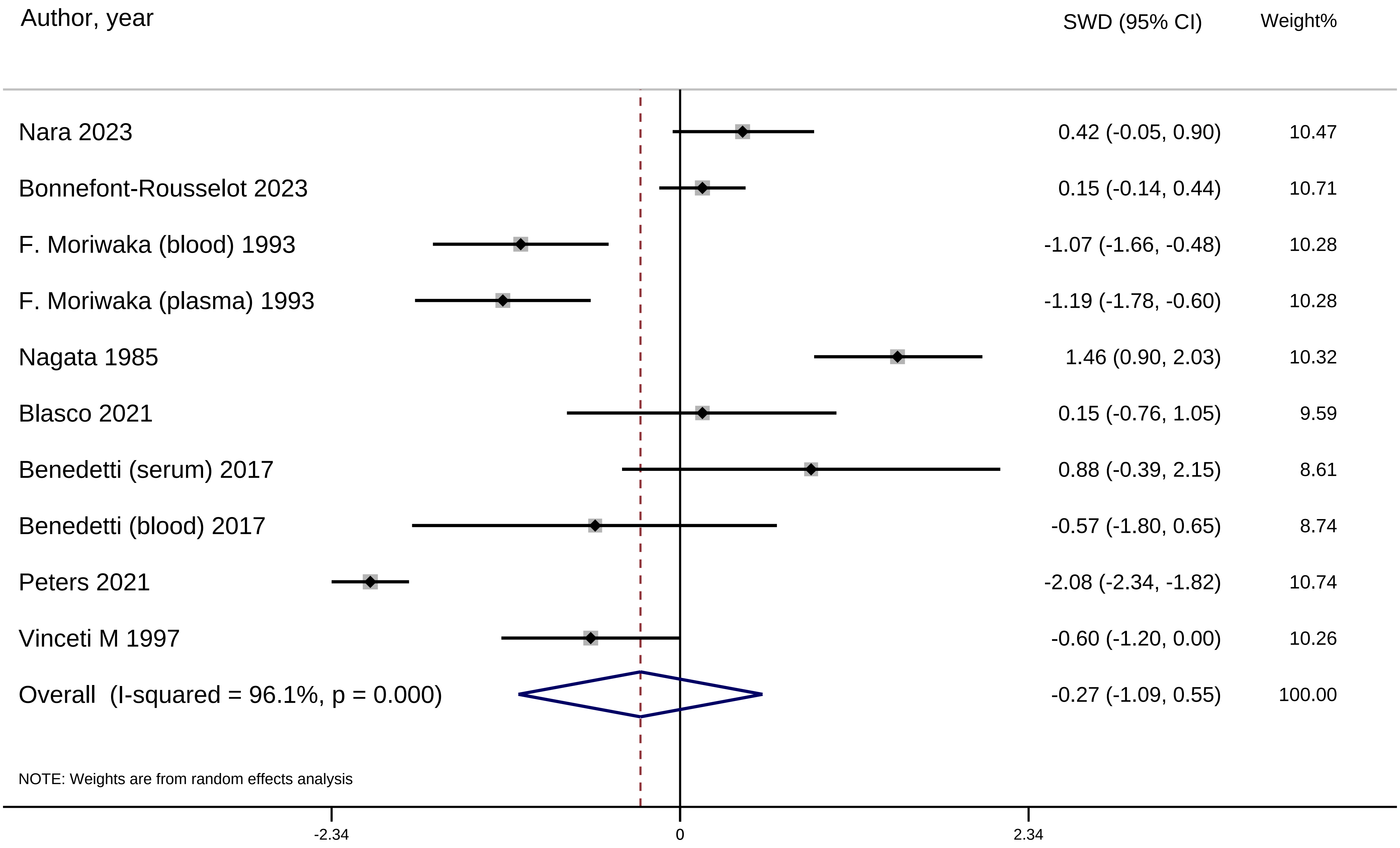

Figure S97. Random-effects meta-analysis of the association between serum selenium level and ALS

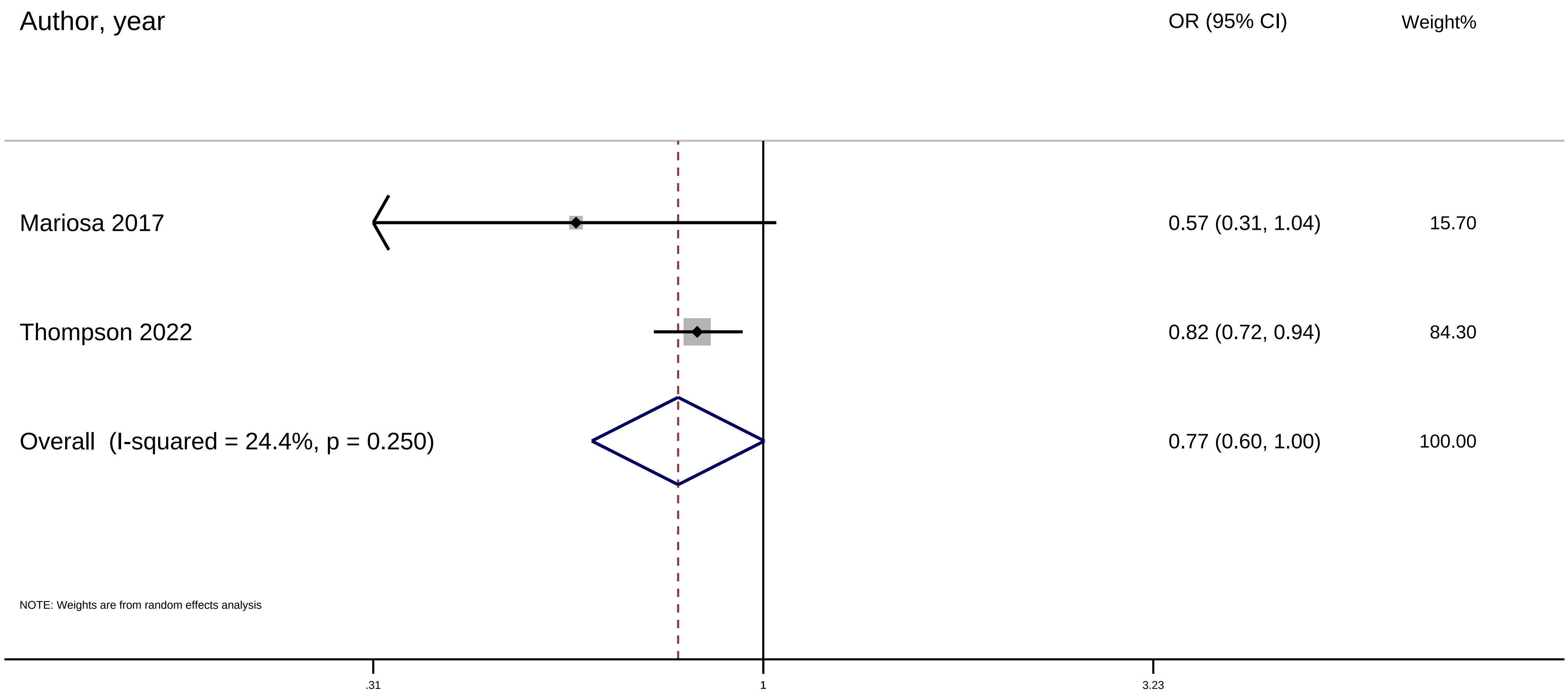

Figure S98. Random-effects meta-analysis of the association between serum ApoA1 level and ALS

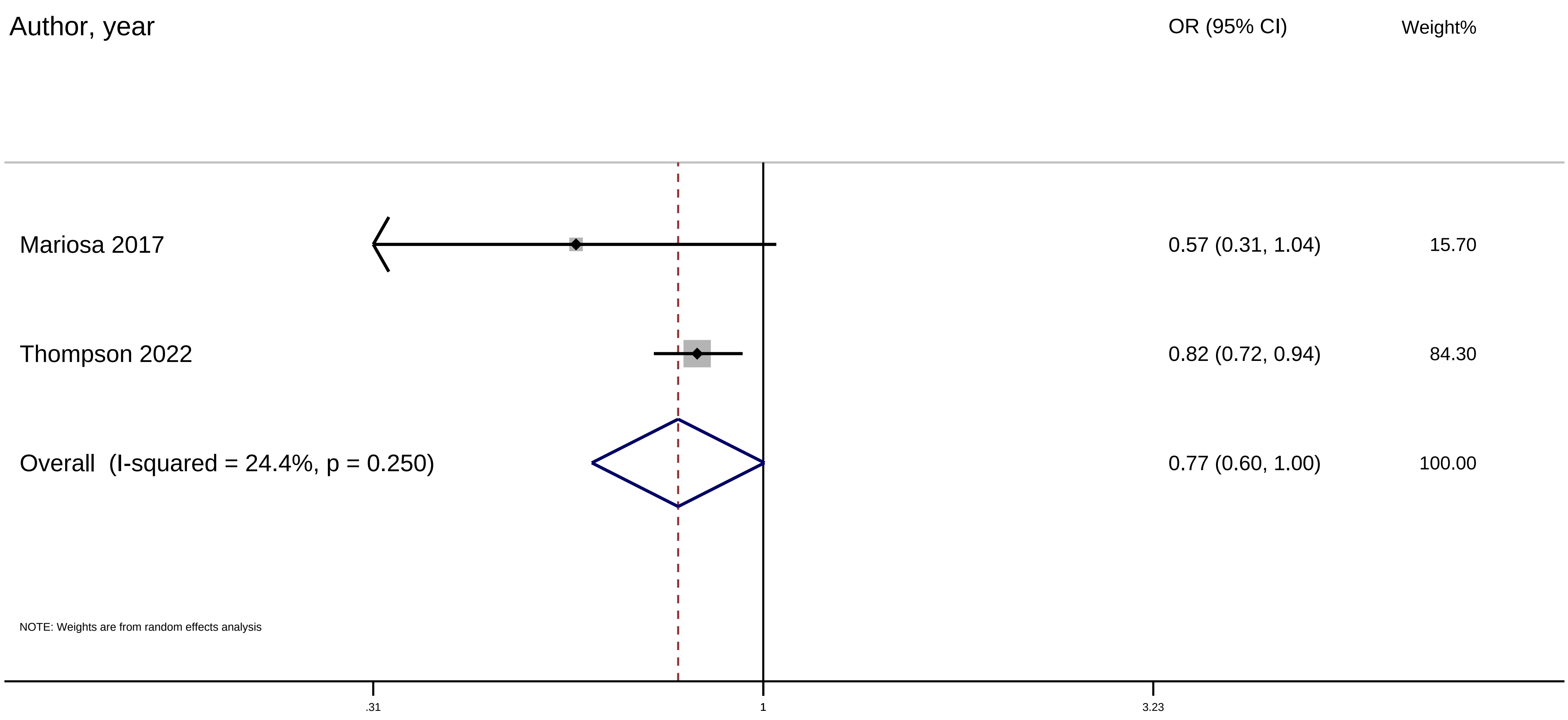

Figure S99. Random-effects meta-analysis of the association between serum ApoB level and ALS

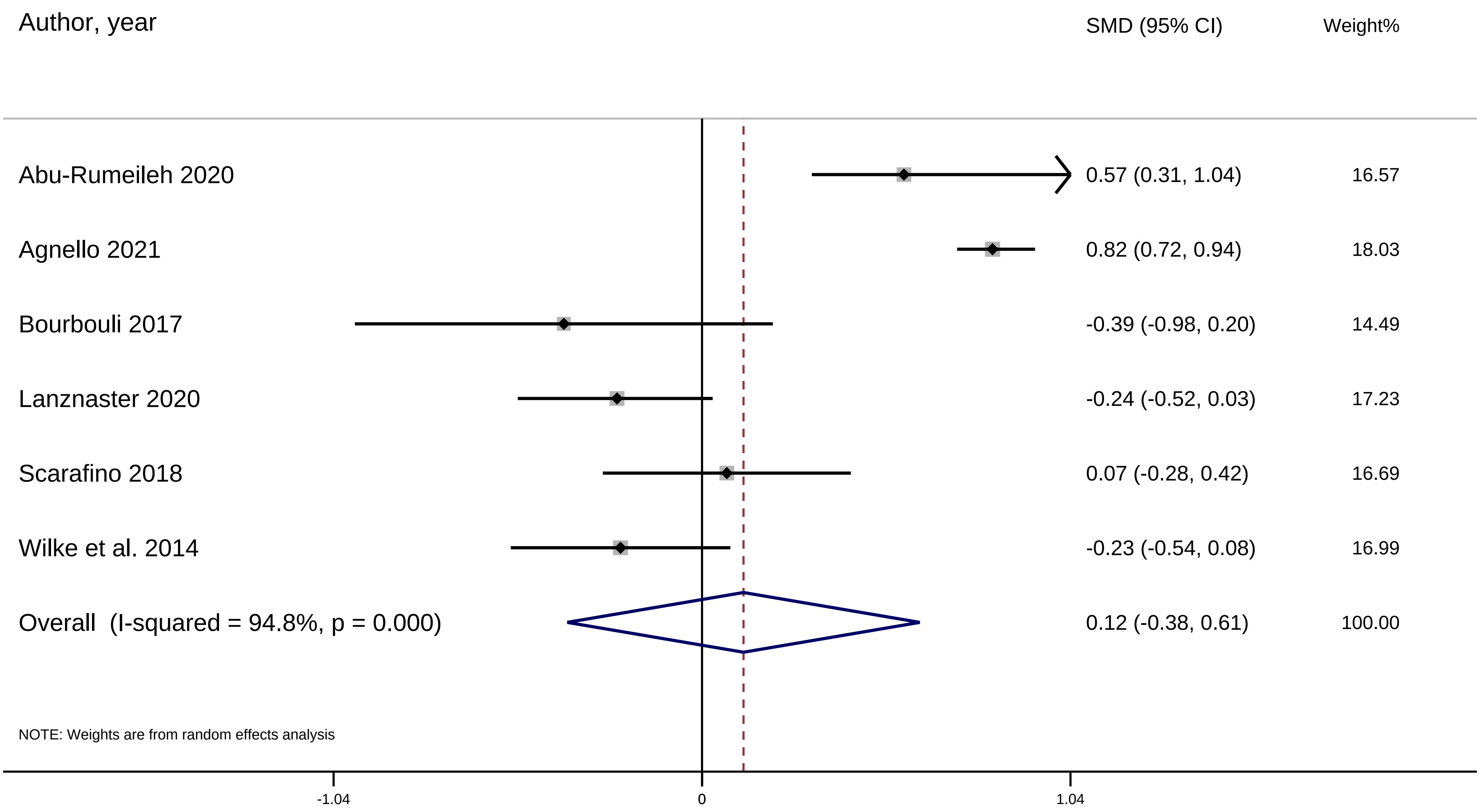

Figure S100. Random-effects meta-analysis of the association between CSF p-tau level and ALS

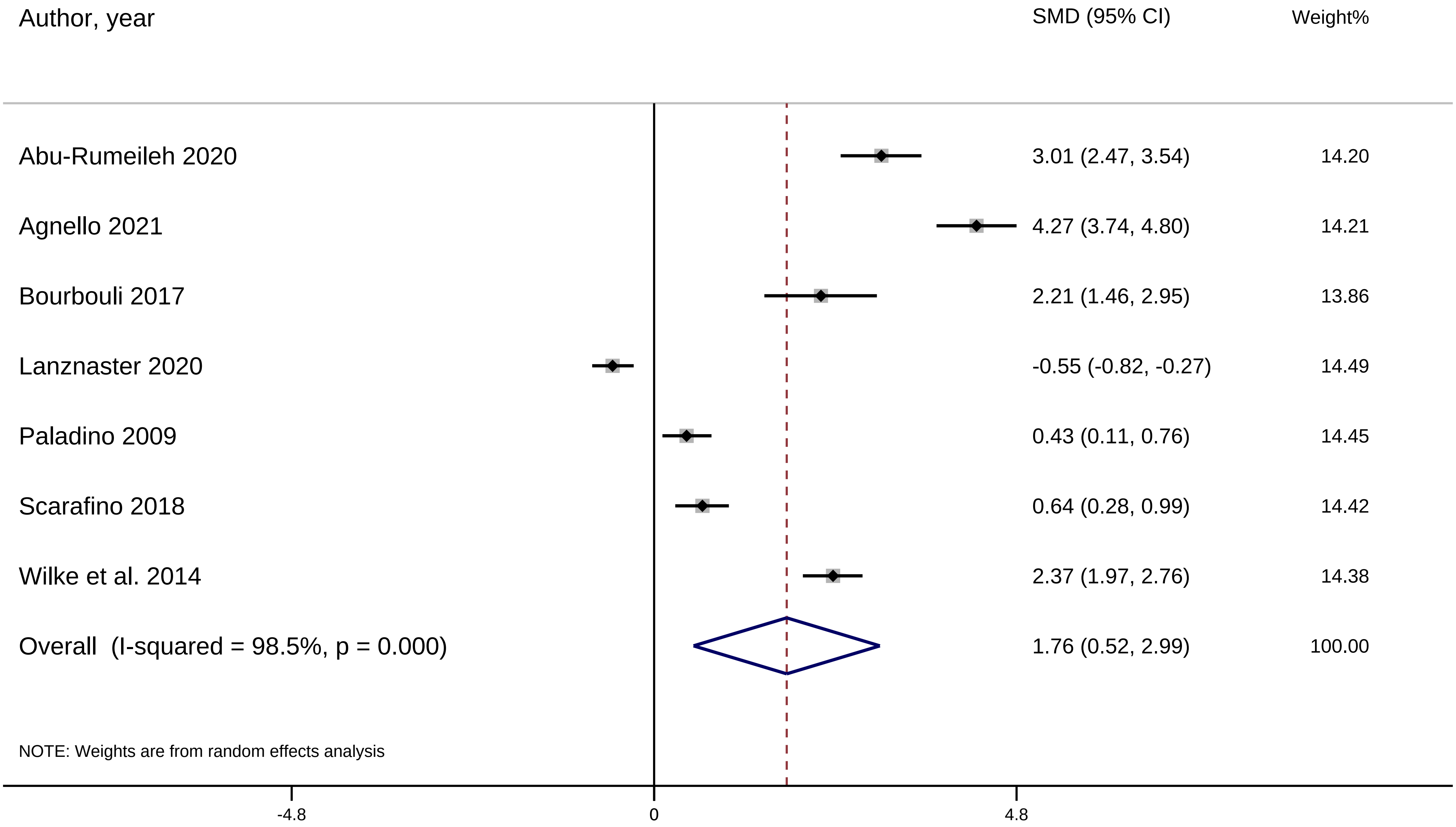

Figure S101. Random-effects meta-analysis of the association between CSF t-tau level and ALS

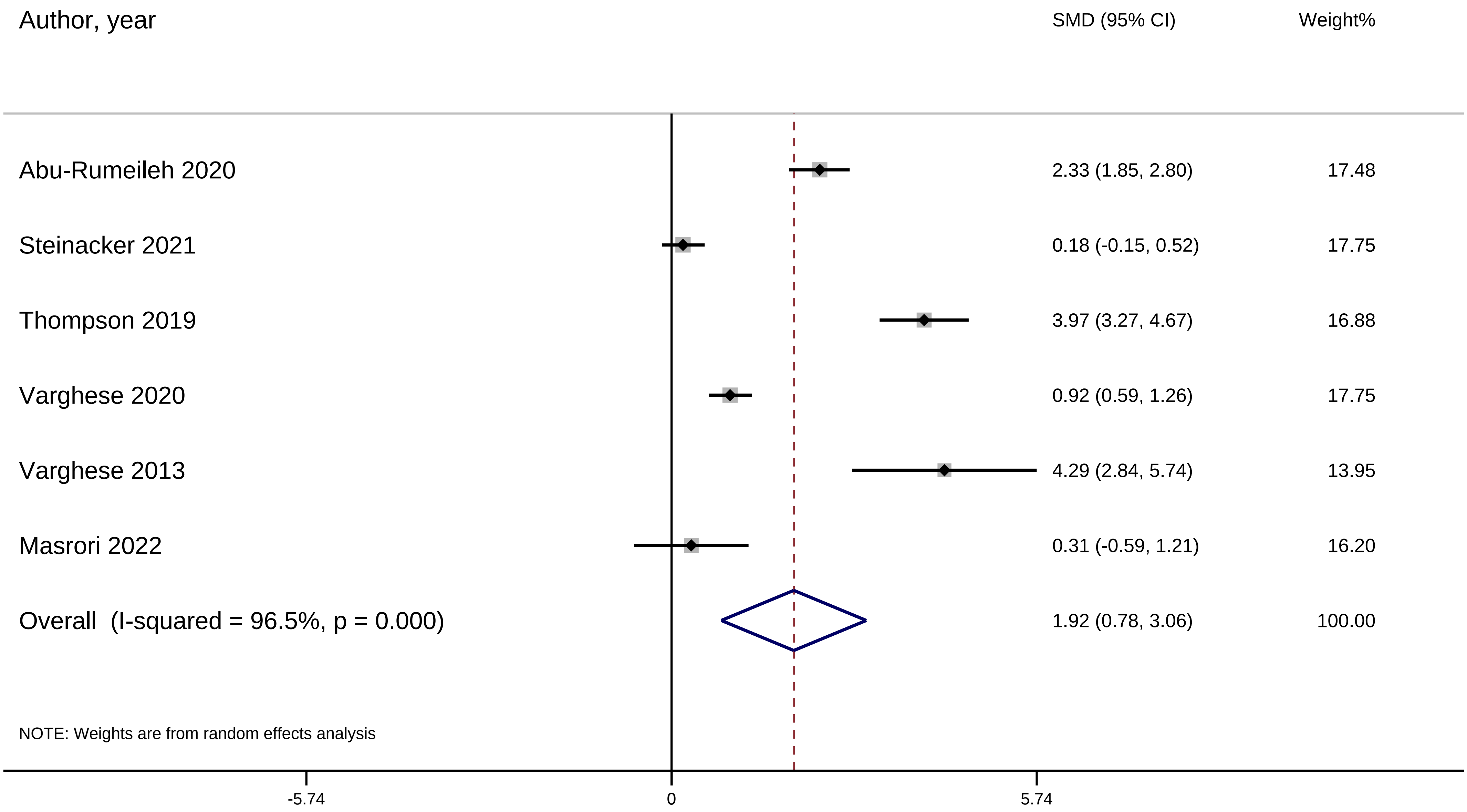

Figure S102. Random-effects meta-analysis of the association between CSF CHIT1 level and ALS

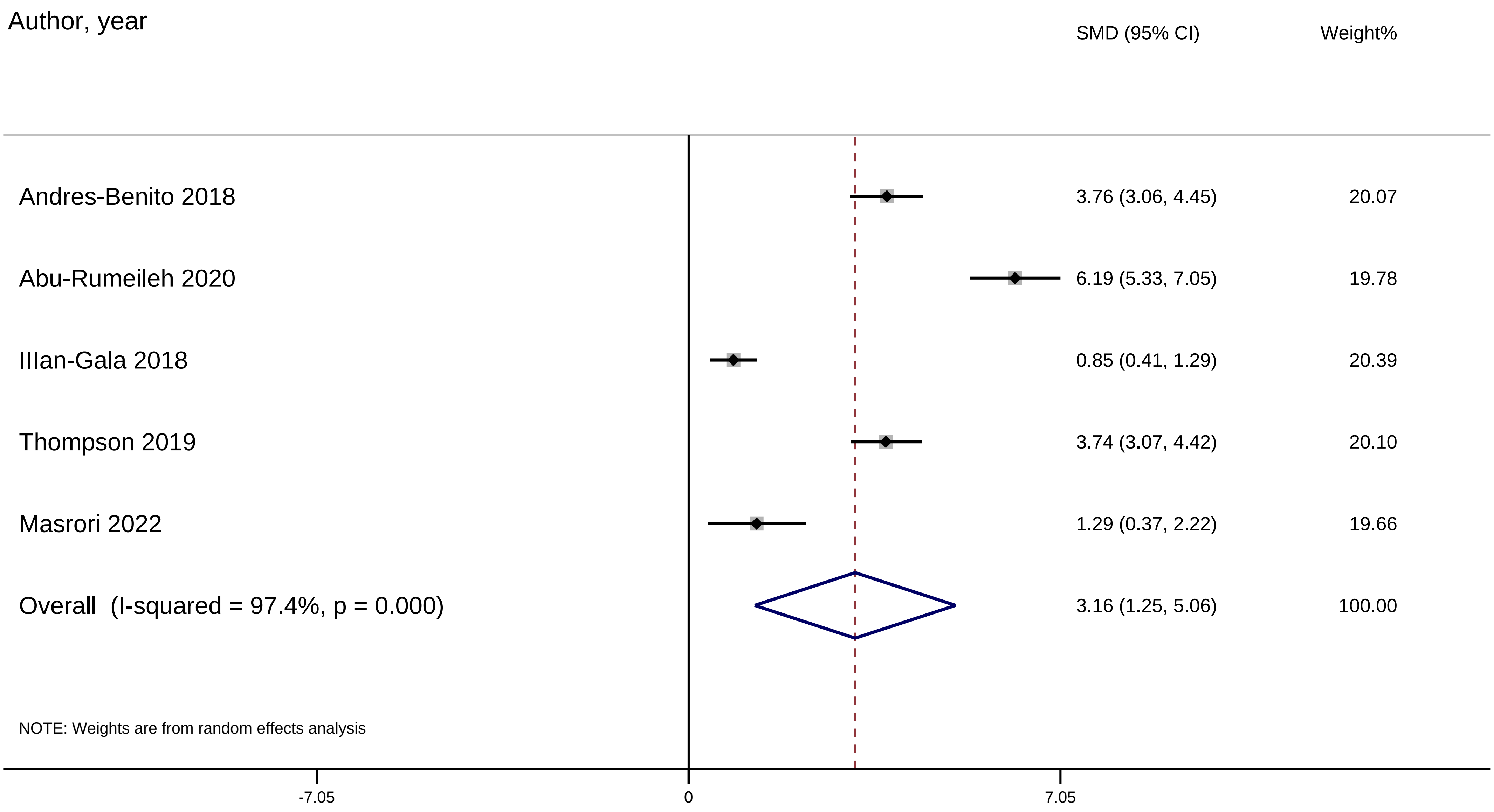

Figure S103. Random-effects meta-analysis of the association between CSF CHI3L1 level and ALS
