# Supplementary material for: Environmental risk factors, protective factors, and biomarkers for amyotrophic lateral sclerosis: an umbrella review
Source: Front Aging Neurosci. 2025 Jun 13;17:1541779. doi: 10.3389/fnagi.2025.1541779 (PMC12202415; doi:10.3389/fnagi.2025.1541779)
Supplement: Supplementary file 2 [file Data_Sheet_2.pdf]

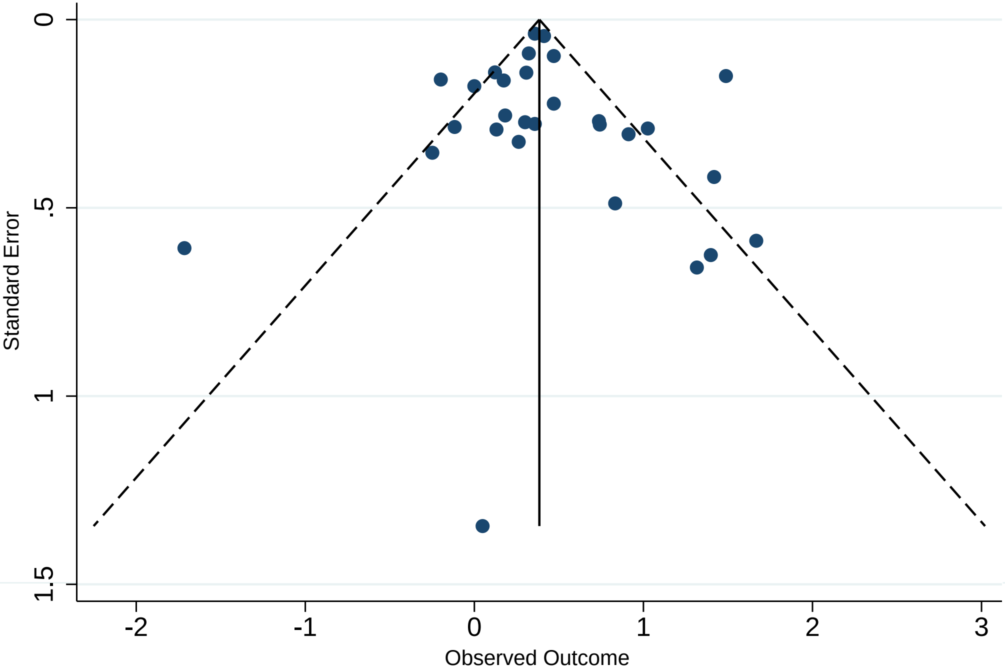

Figure S104. Funnel plot of meta-analysis of the association between trauma and ALS

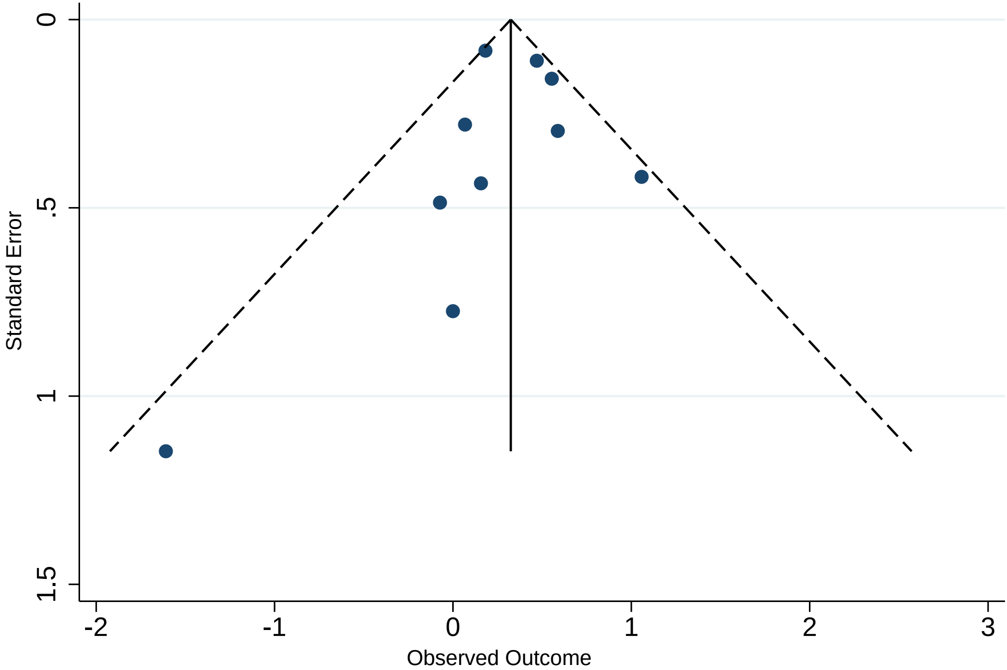

Figure S105. Funnel plot of meta-analysis of the association between farming occupation and ALS

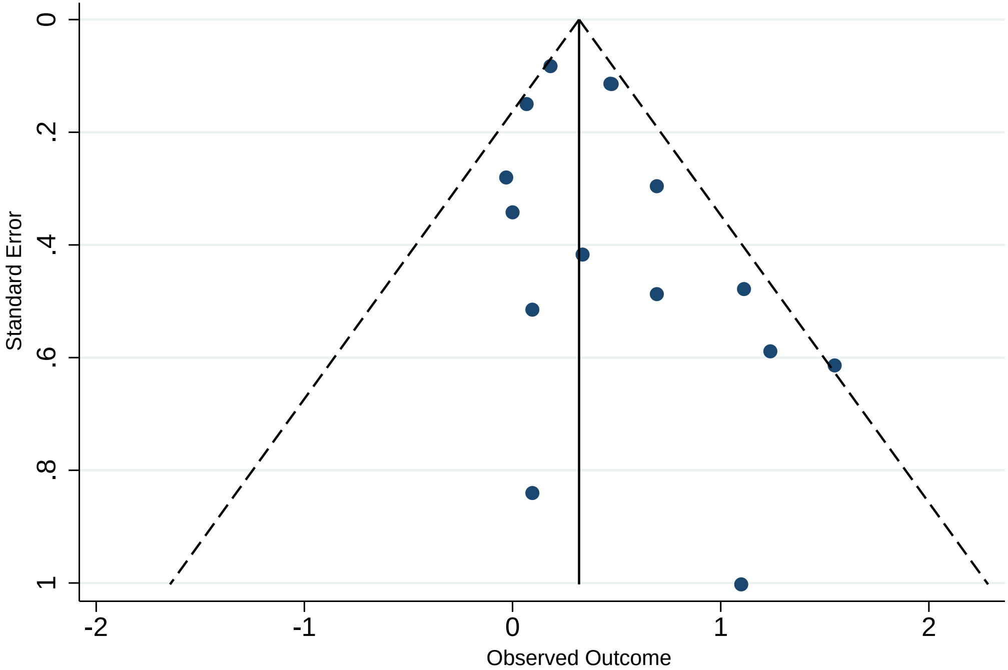

Figure S106. Funnel plot of meta-analysis of the association between pesticide and ALS

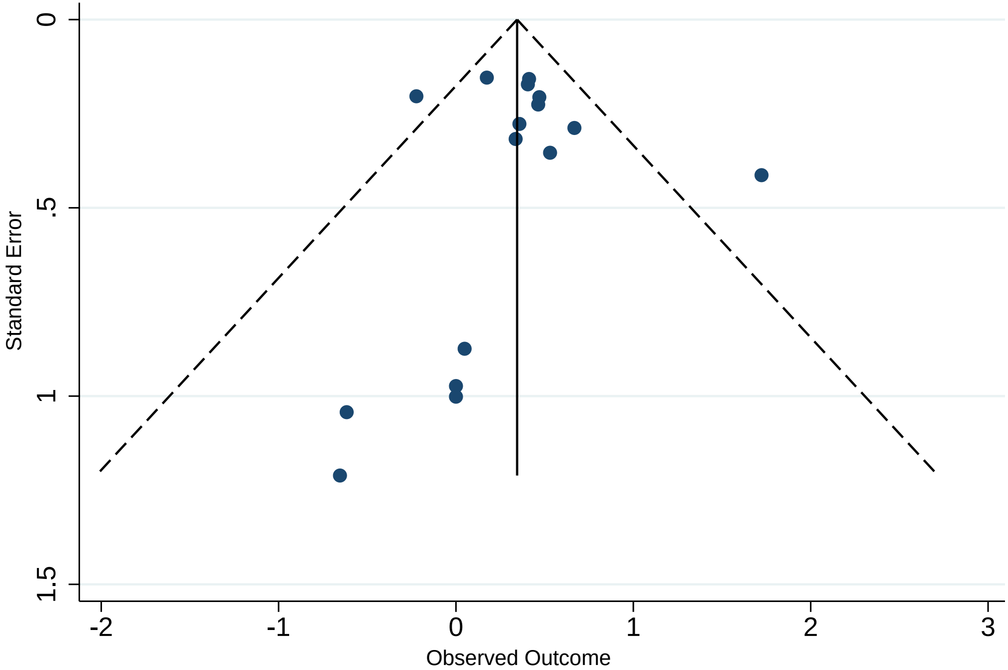

Figure S107. Funnel plot of meta-analysis of the association between head injury and ALS

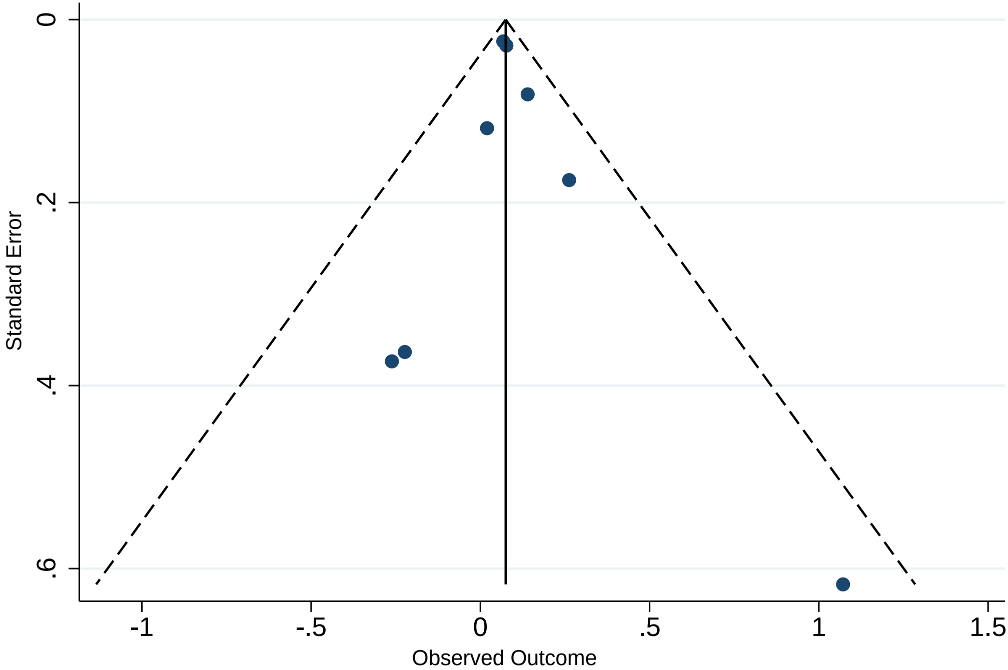

Figure S108. Funnel plot of meta-analysis of the association between leisure time activity and ALS

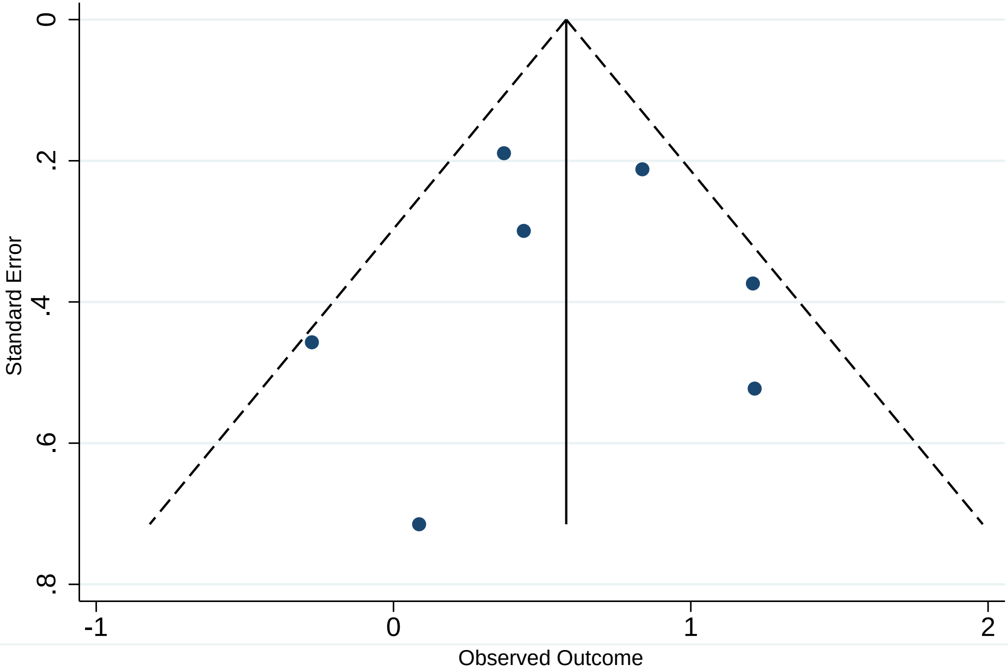

Figure S109. Funnel plot of meta-analysis of the association between heavy metals and ALS

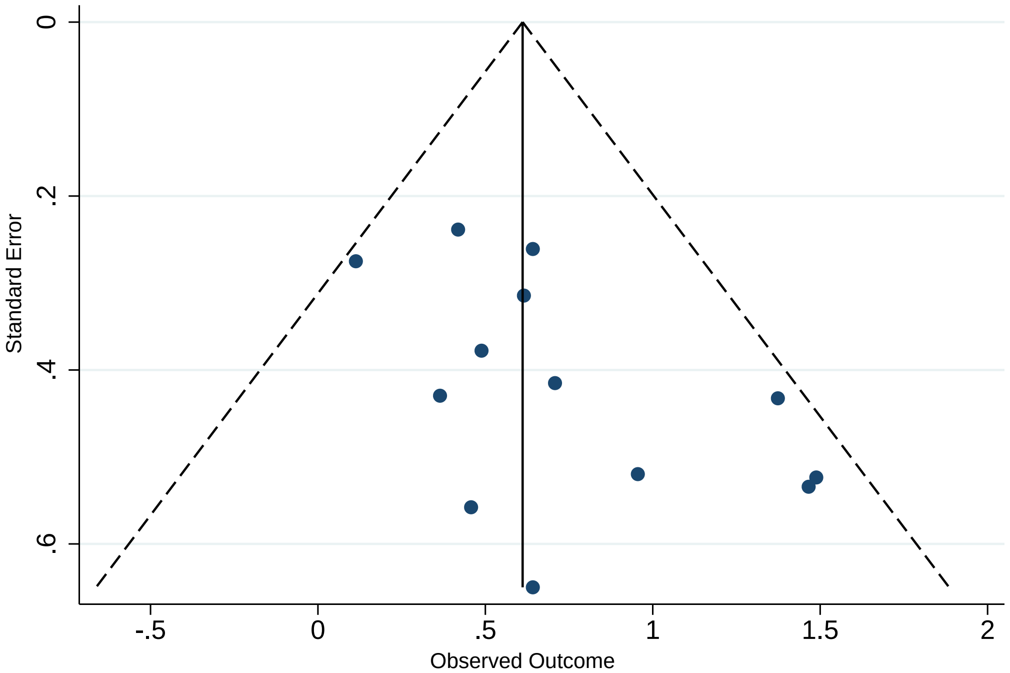

Figure S110. Funnel plot of meta-analysis of the association between metals and ALS

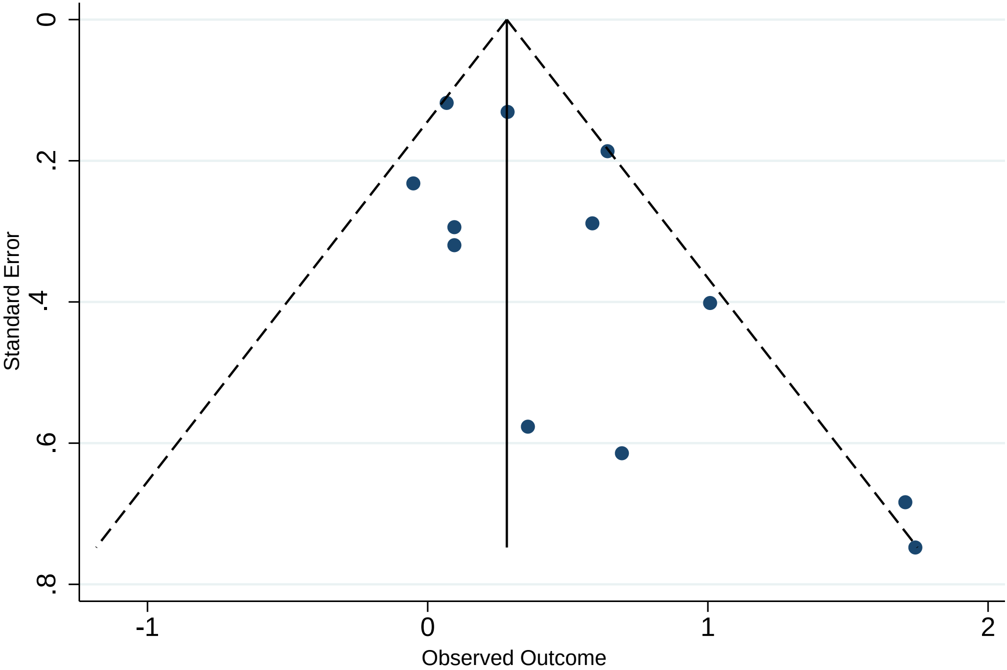

Figure S111. Funnel plot of meta-analysis of the association between lead and ALS

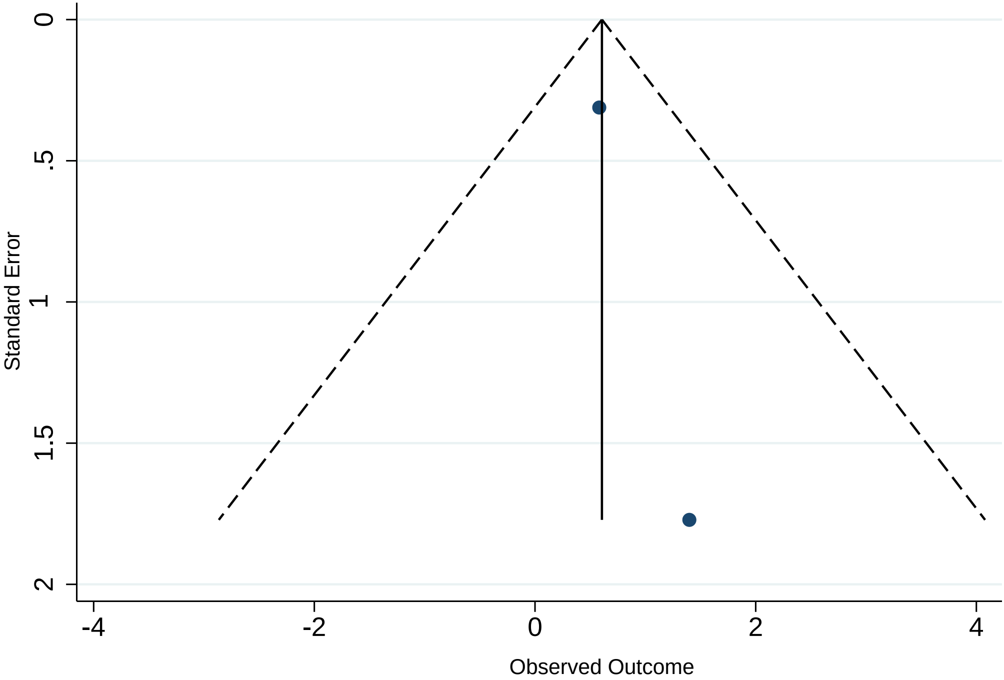

Figure S112. Random-effects meta-analysis of the association between annual PM2.5 exposure and ALS

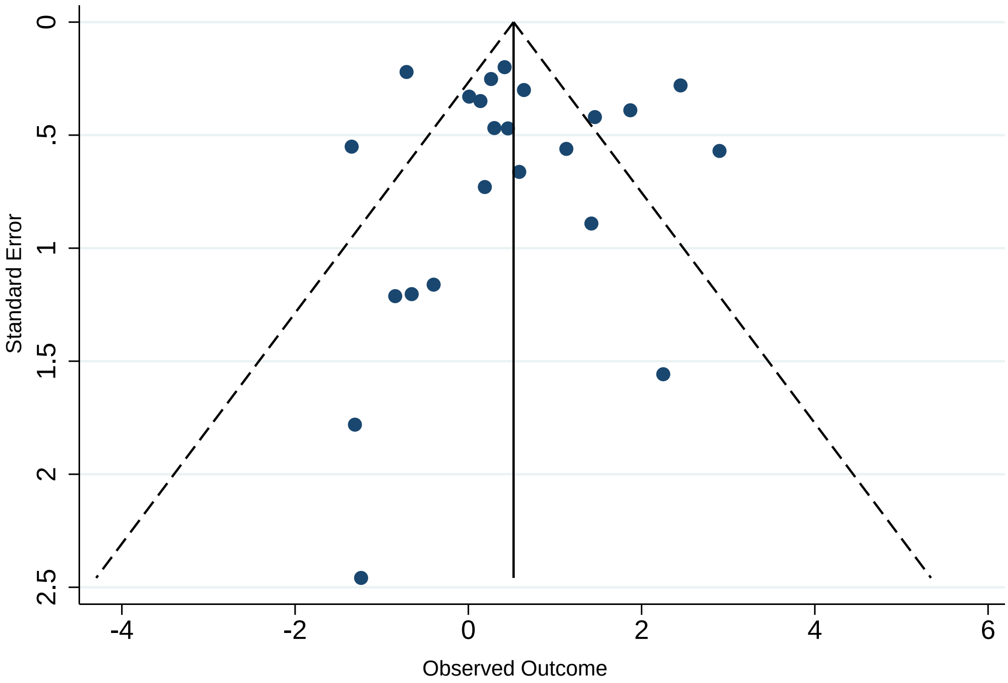

Figure S113. Funnel plot of meta-analysis of the association between competitive organized sports and ALS

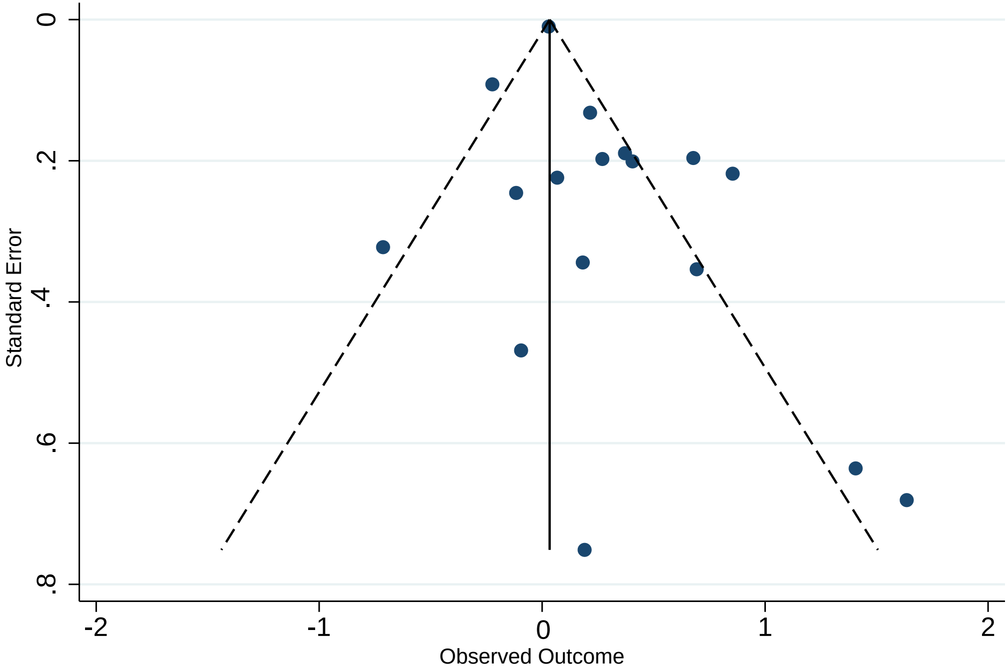

Figure S114. Funnel plot of meta-analysis of the association between vigorous physical activity and ALS

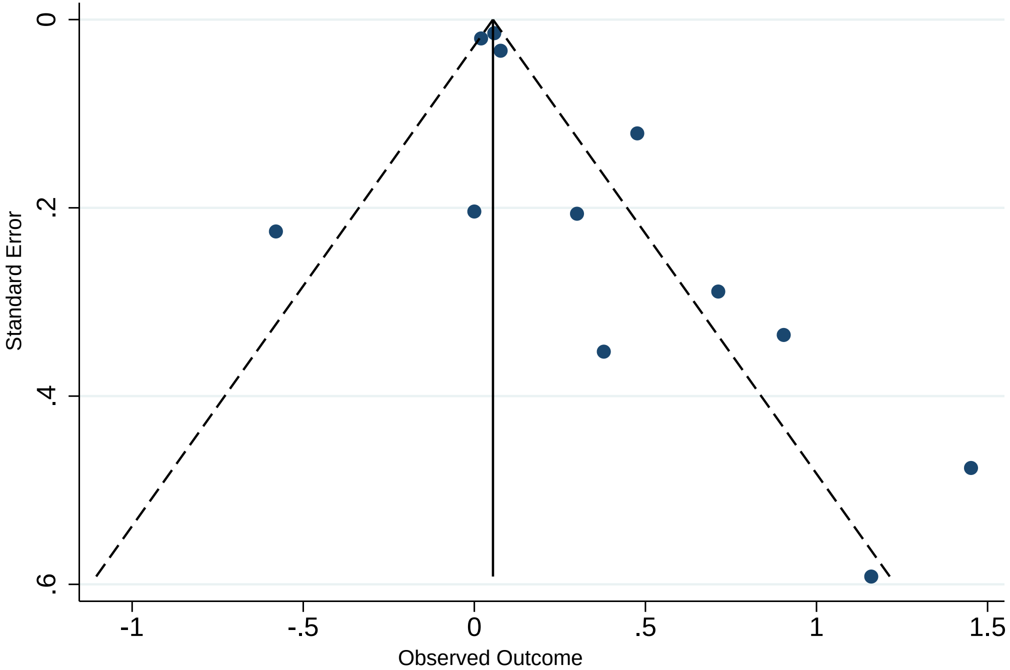

Figure S115. Funnel plot of meta-analysis of the association between occupational-related activity and ALS

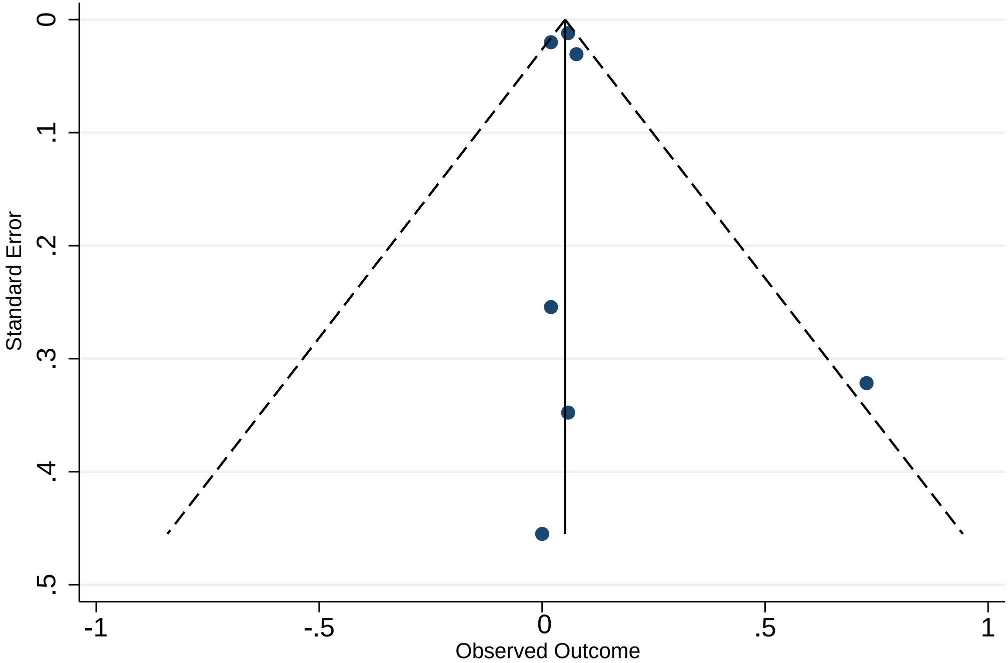

Figure S116. Funnel plot of meta-analysis of the association between unclassified physical activity and ALS

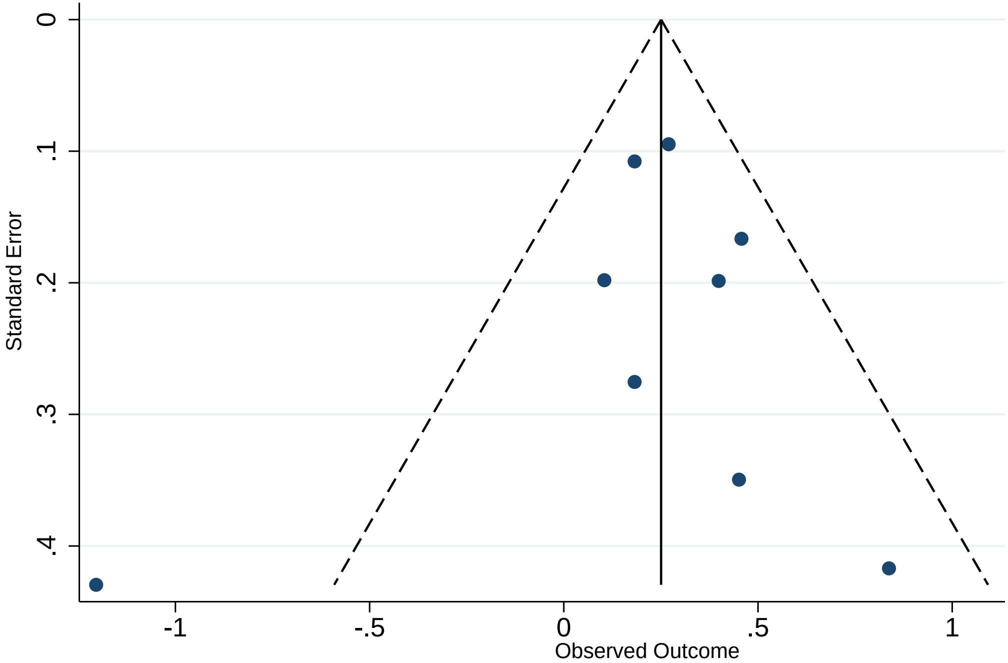

Figure S117. Funnel plot of meta-analysis of the association between military personnel and ALS

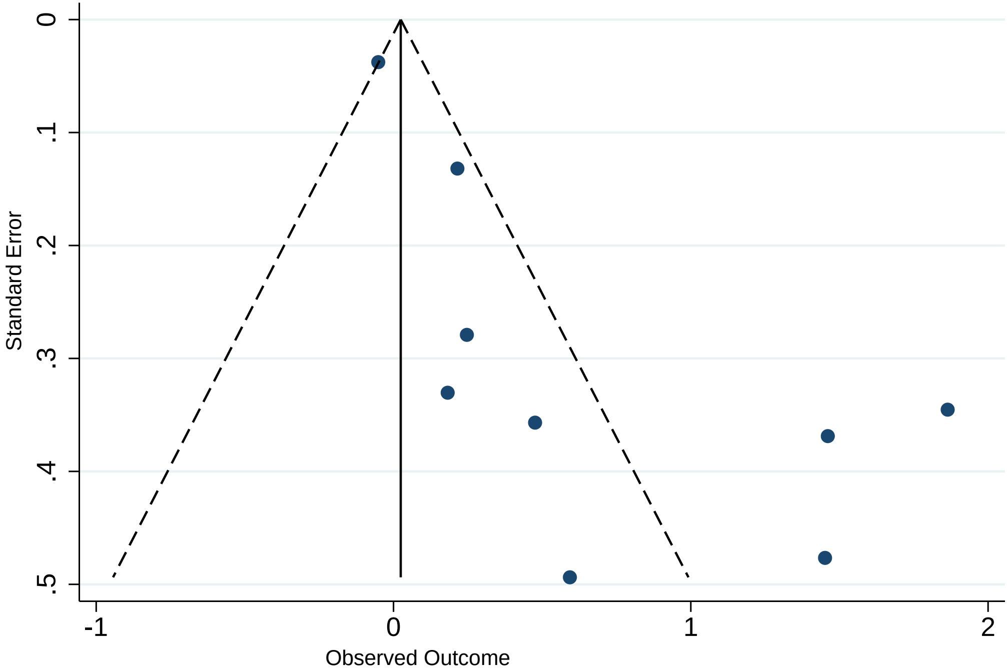

Figure S118. Funnel plot of meta-analysis of the association between heavy physical work and ALS

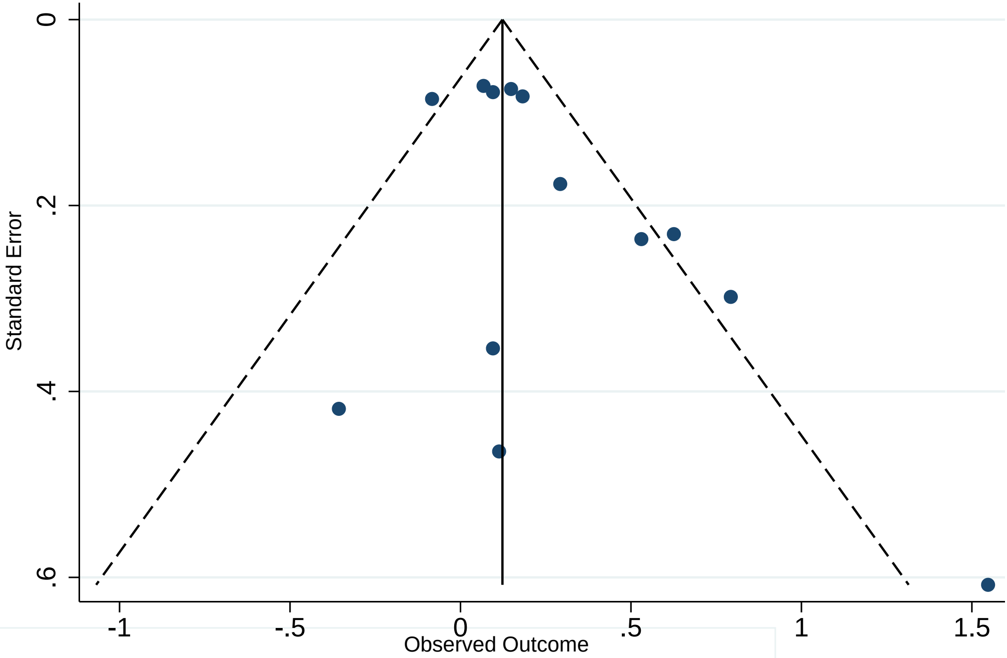

Figure S119. Funnel plot of meta-analysis of the association between chemicals and ALS

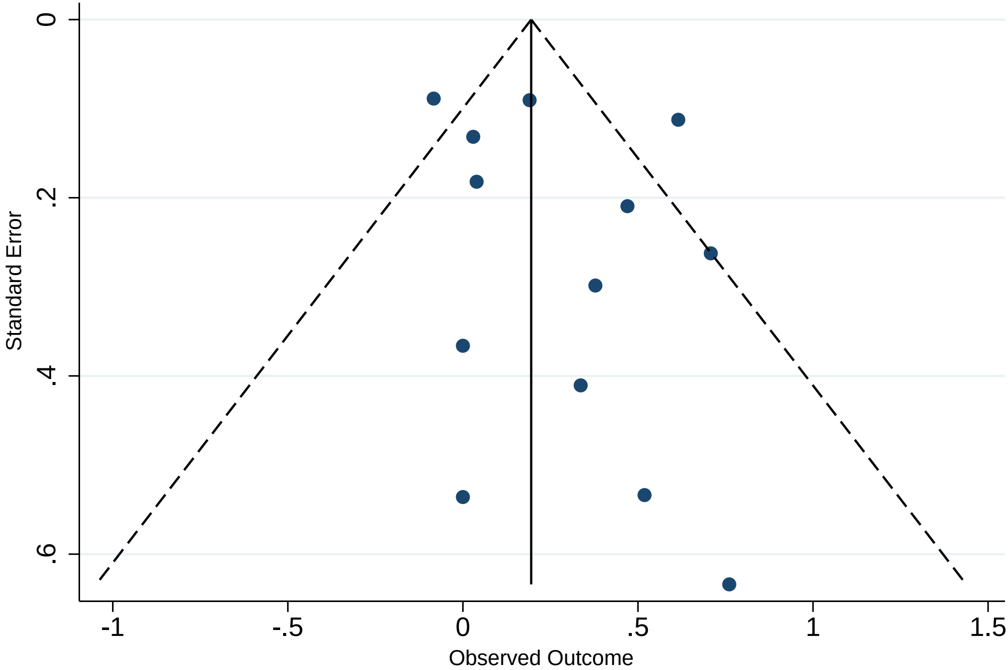

Figure S120. Funnel plot of meta-analysis of the association between environmental and occupational solvents and ALS

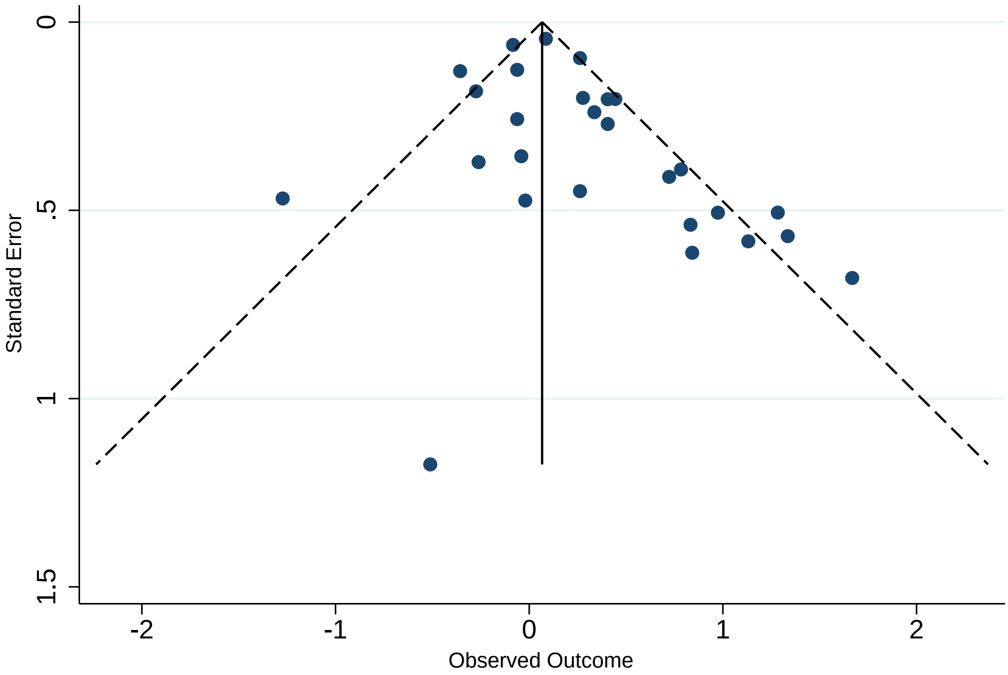

Figure S121. Funnel plot of meta-analysis of the association between extremely low frequency magnetic fields and ALS

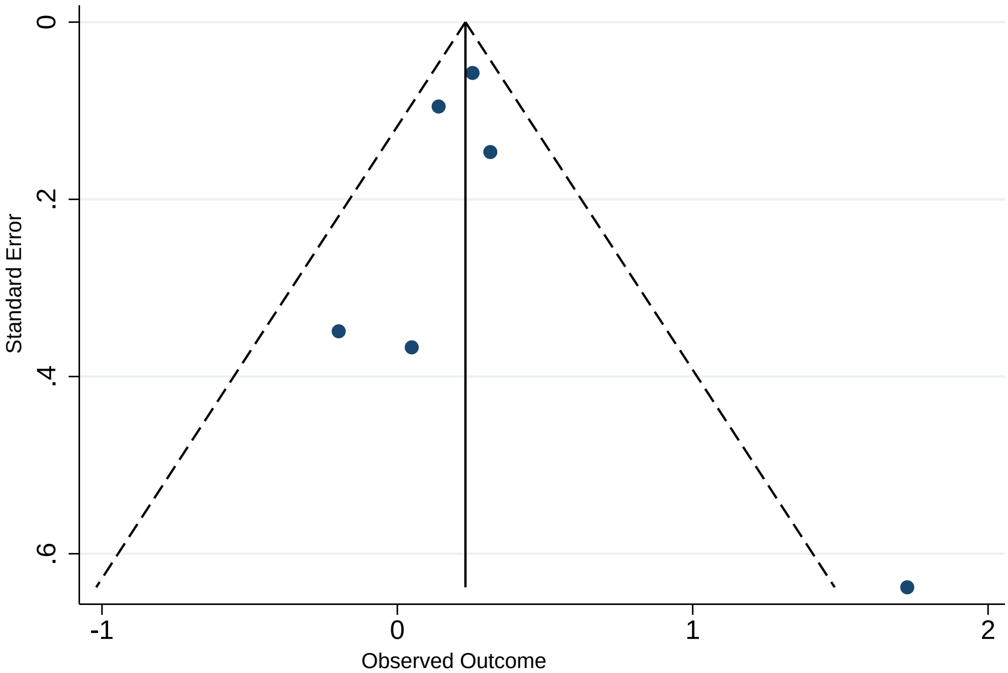

Figure S122. Funnel plot of meta-analysis of the association between stroke and ALS

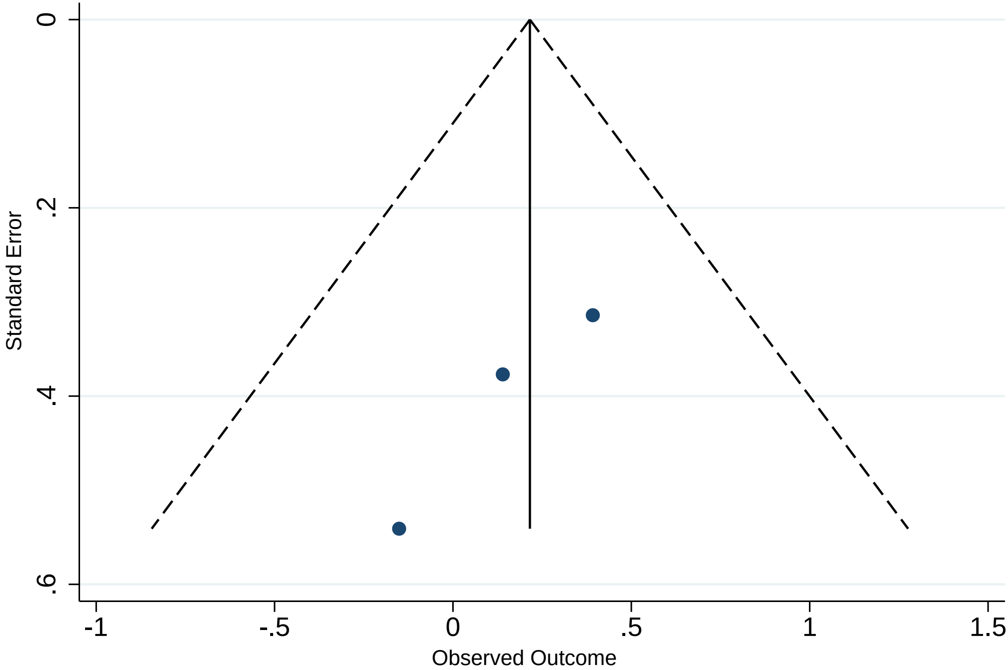

Figure S123. Funnel plot of meta-analysis of the association between occupation in industry and ALS

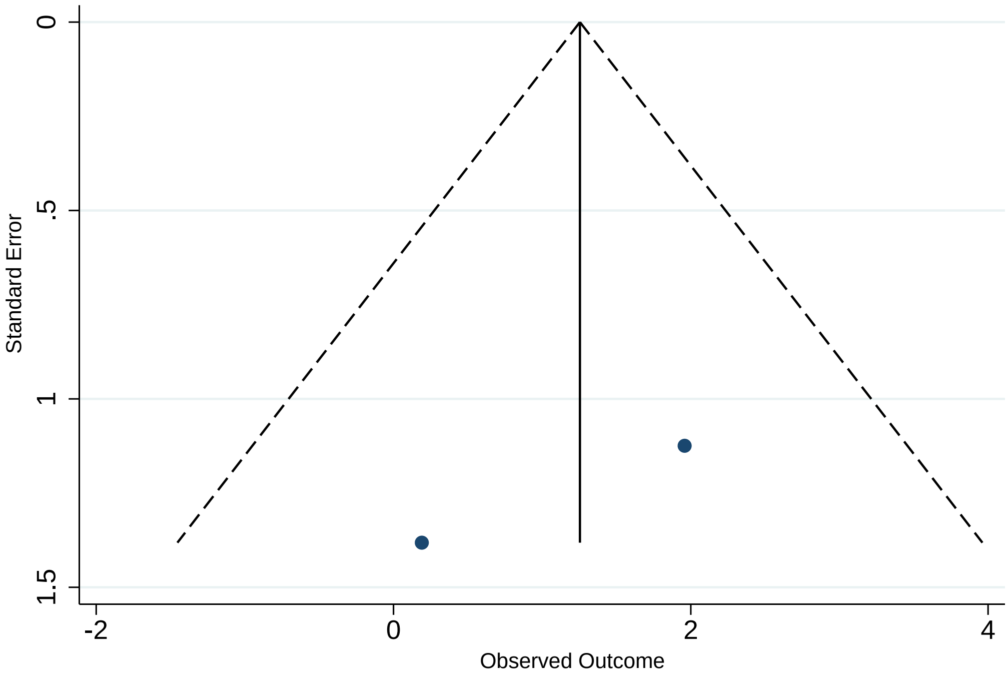

Figure S124. Funnel plot of meta-analysis of the association between annual PM10 exposure and ALS

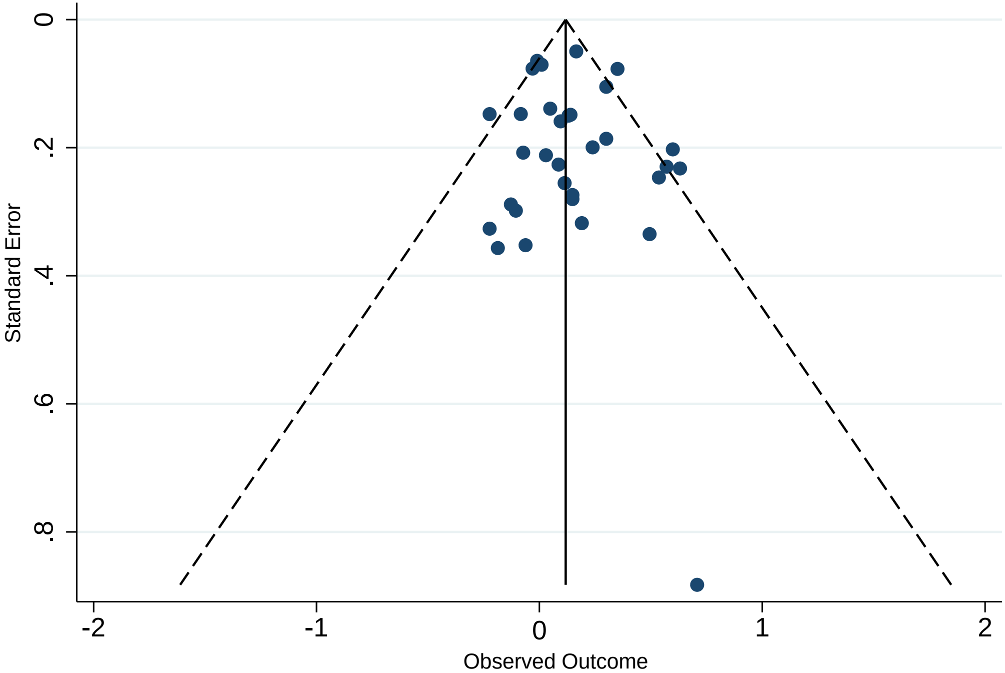

Figure S125. Funnel plot of meta-analysis of the association between smoking and ALS

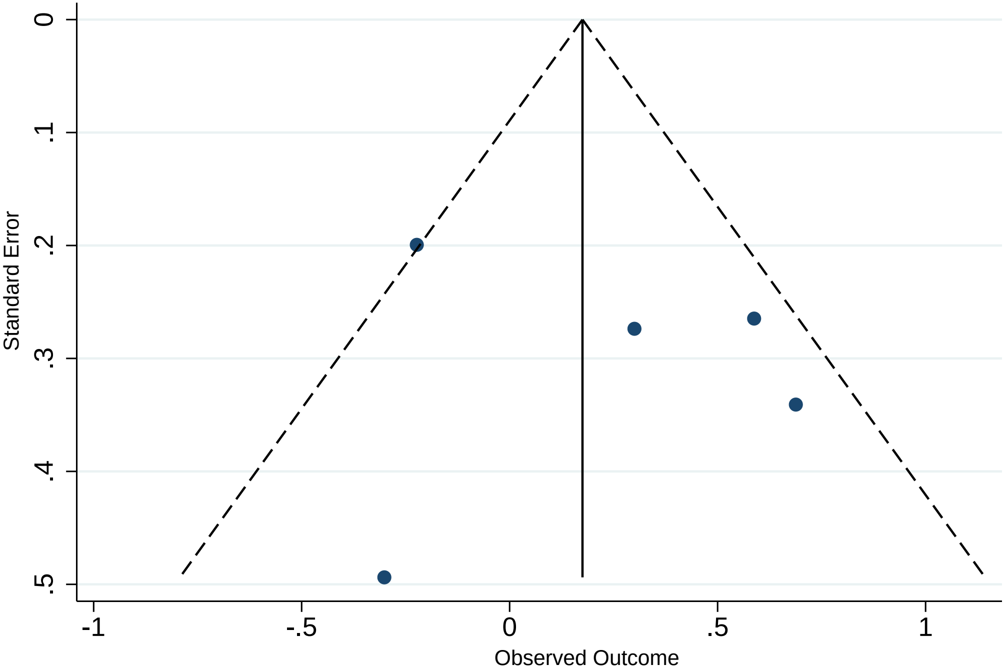

Figure S126. Funnel plot of meta-analysis of the association between rural residence and ALS

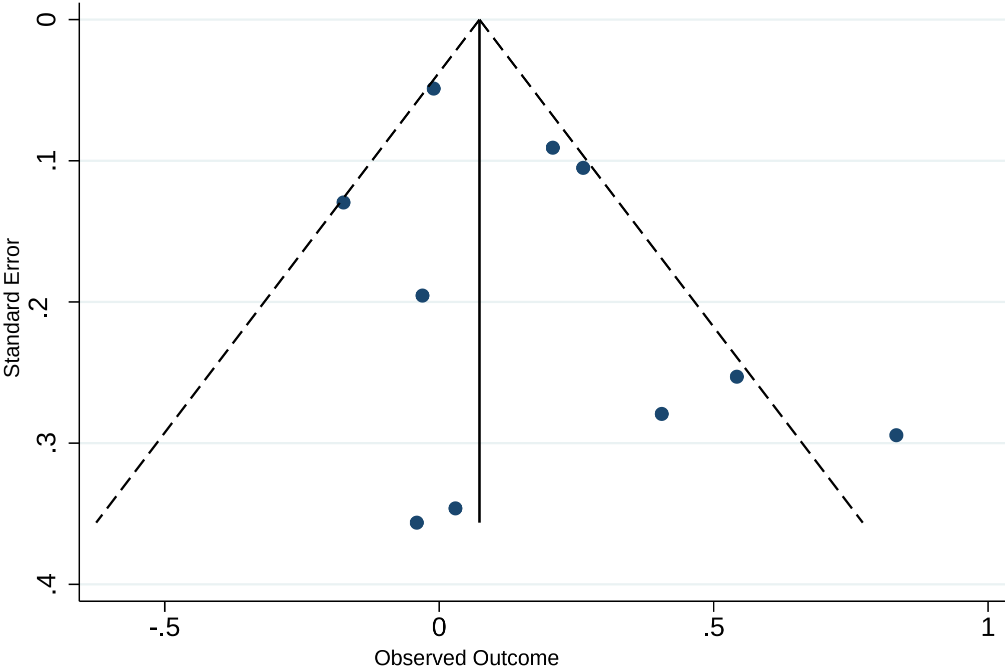

Figure S127. Funnel plot of meta-analysis of the association between work with electricity and ALS

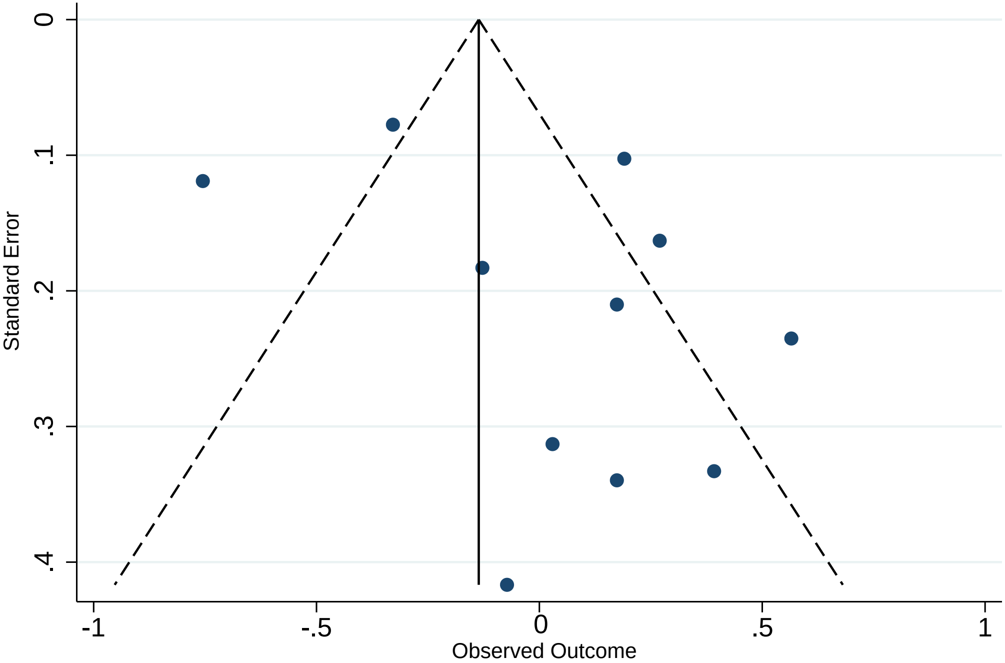

Figure S128. Funnel plot of meta-analysis of the association between alcohol consumption and ALS

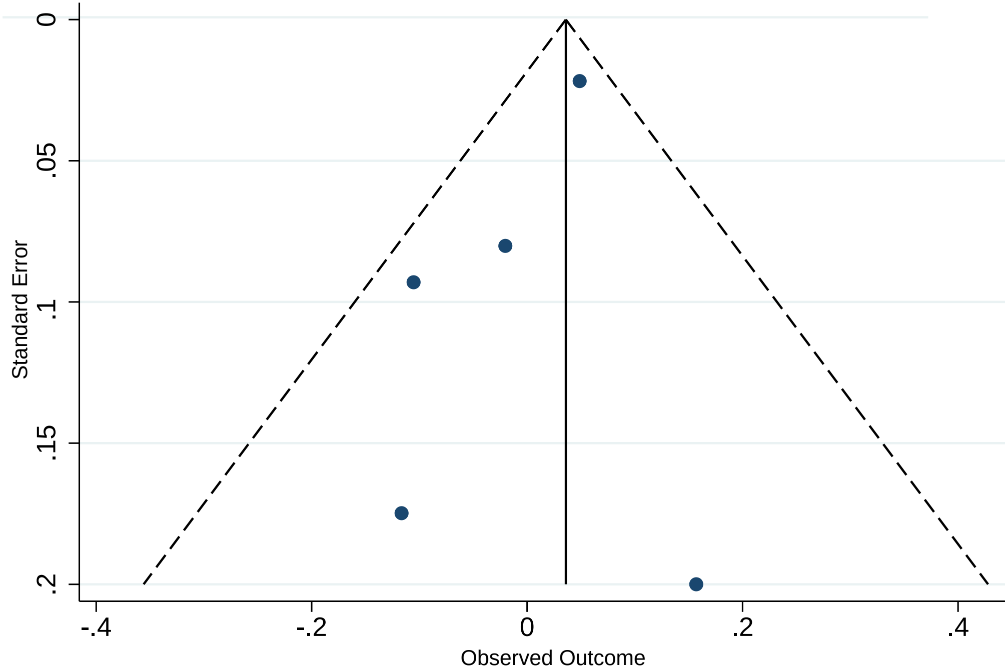

Figure S129. Funnel plot of meta-analysis of the association between hypertension and ALS

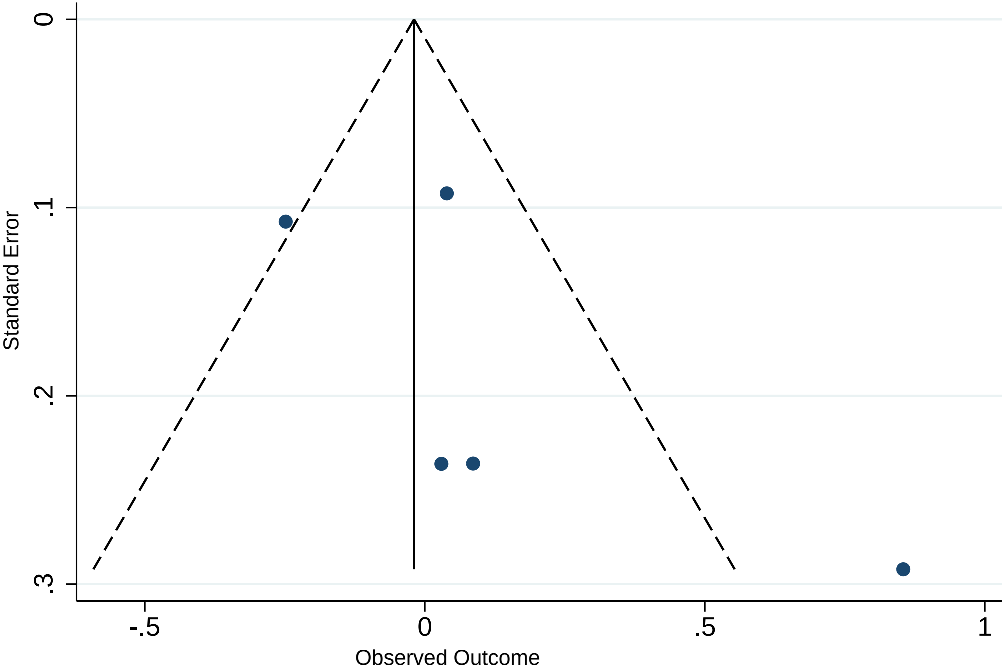

Figure S130. Funnel plot of meta-analysis of the association between NSAIDs and ALS

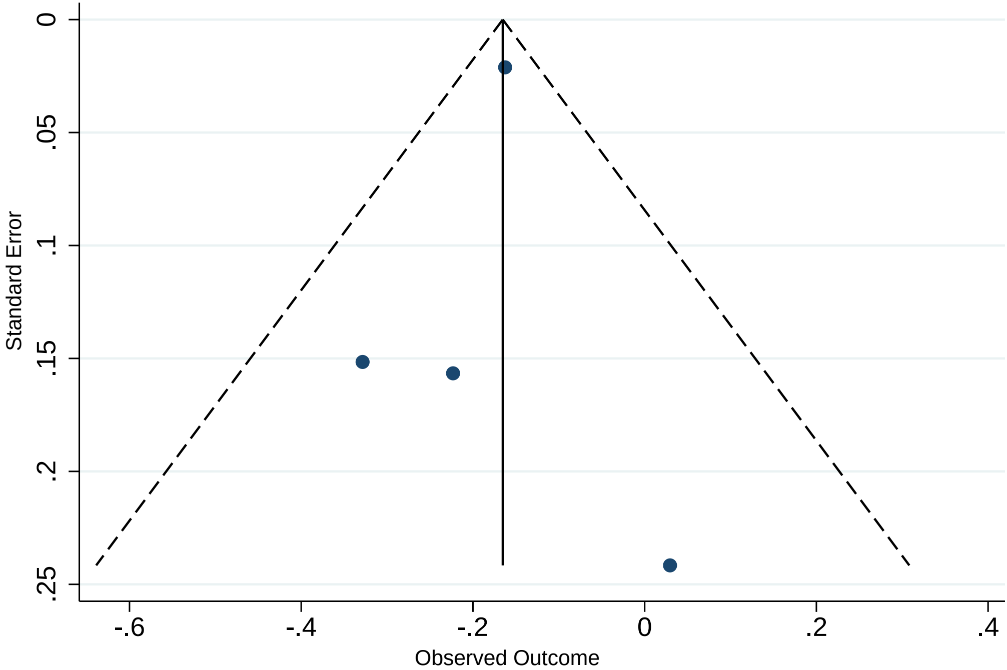

Figure S131. Funnel plot of meta-analysis of the association between anti-hypertensives and ALS

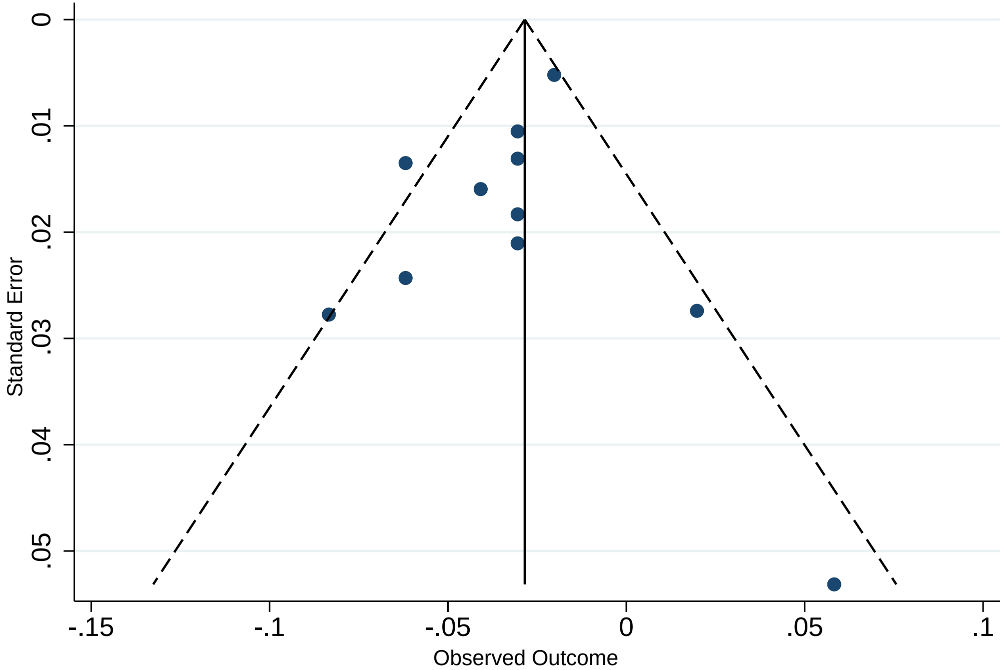

Figure S132. Funnel plot of meta-analysis of the association between premorbid body mass index and ALS

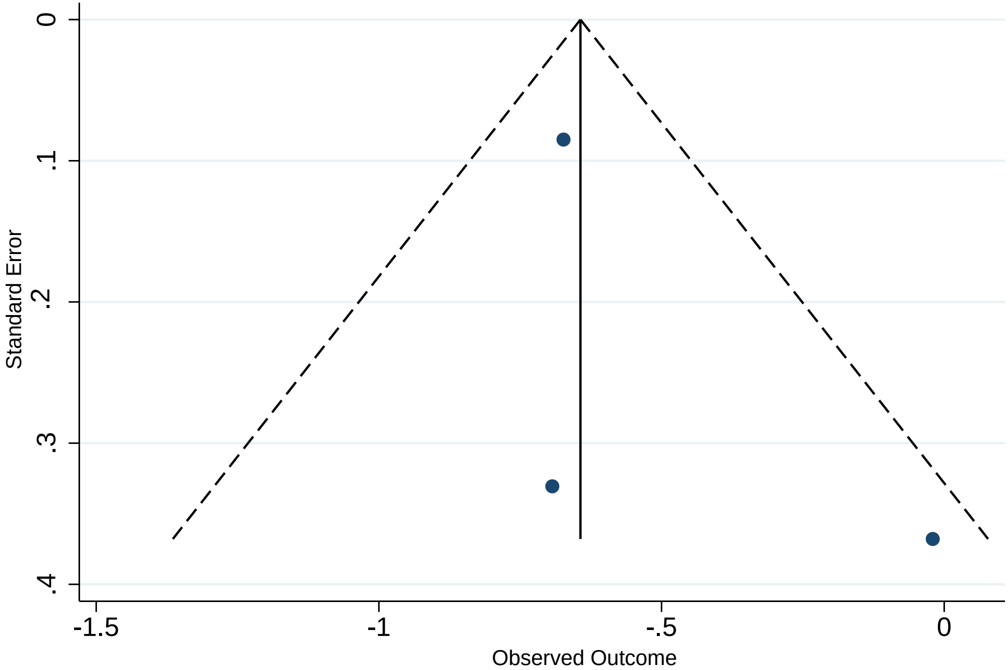

Figure S133. Funnel plot of meta-analysis of the association between anti-diabetes and ALS

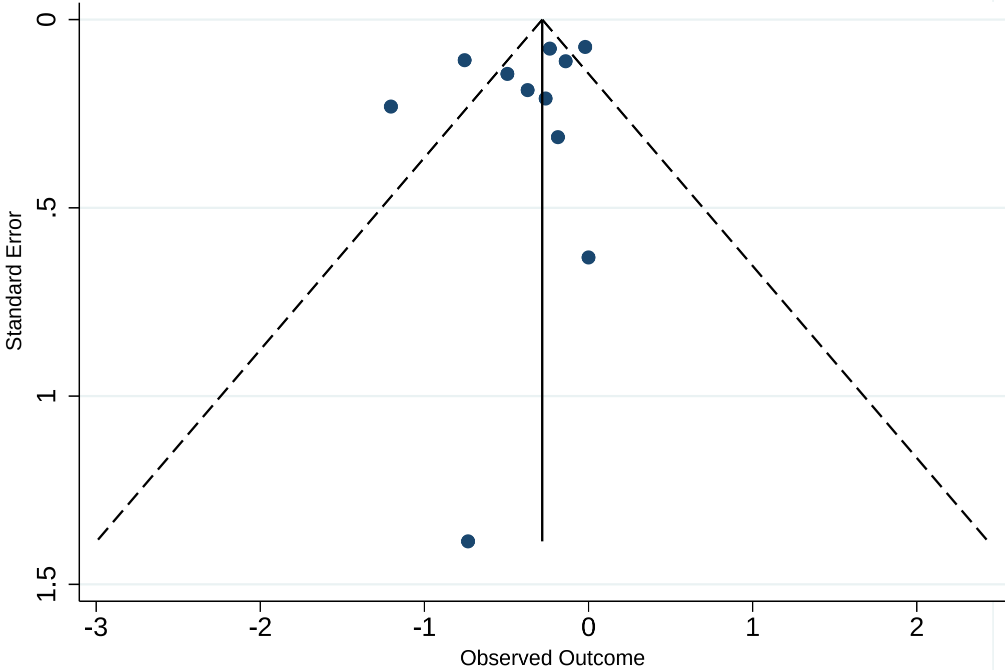

Figure S134. Funnel plot of meta-analysis of the association between diabetes mellitus and ALS

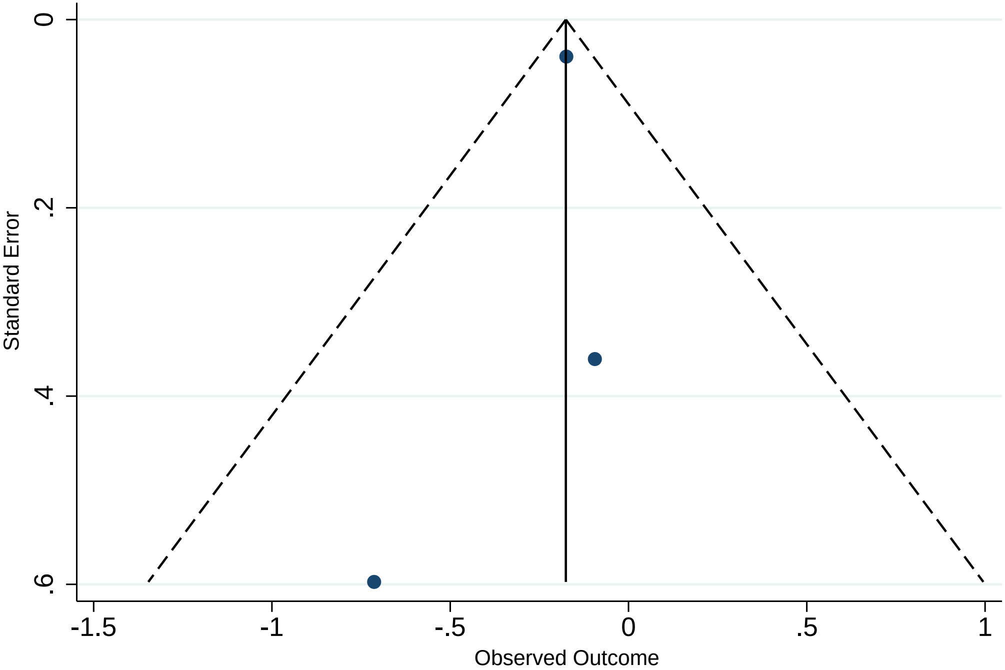

Figure S135. Funnel plot of meta-analysis of the association between kidney diseases and ALS

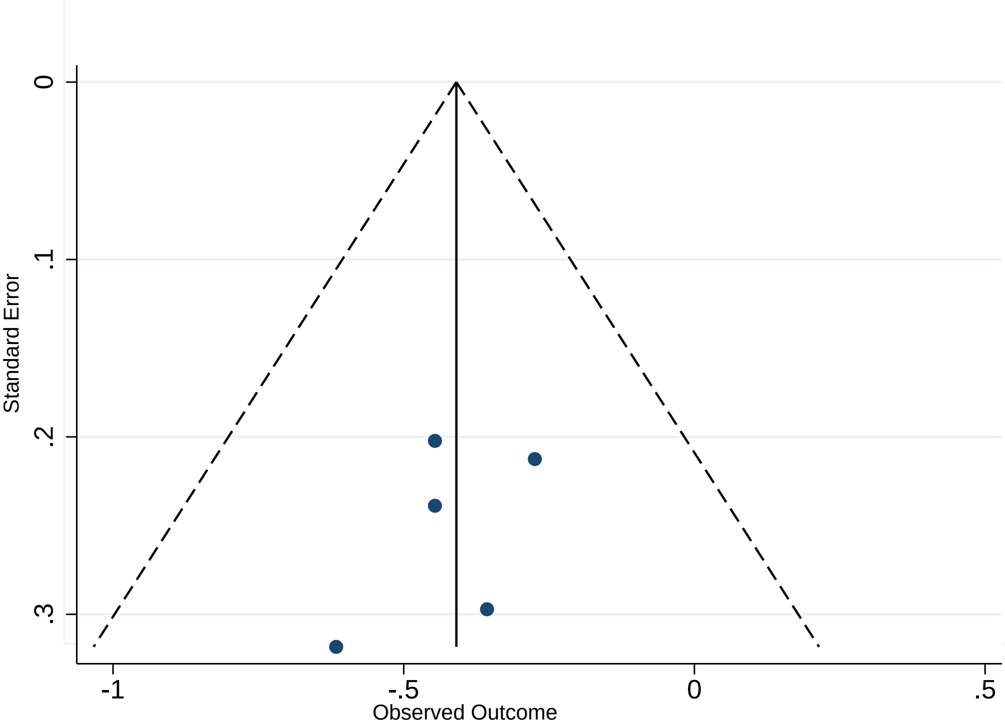

Figure S136. Funnel plot of meta-analysis of the association between ω-3 polyunsaturated fatty acid intake and ALS

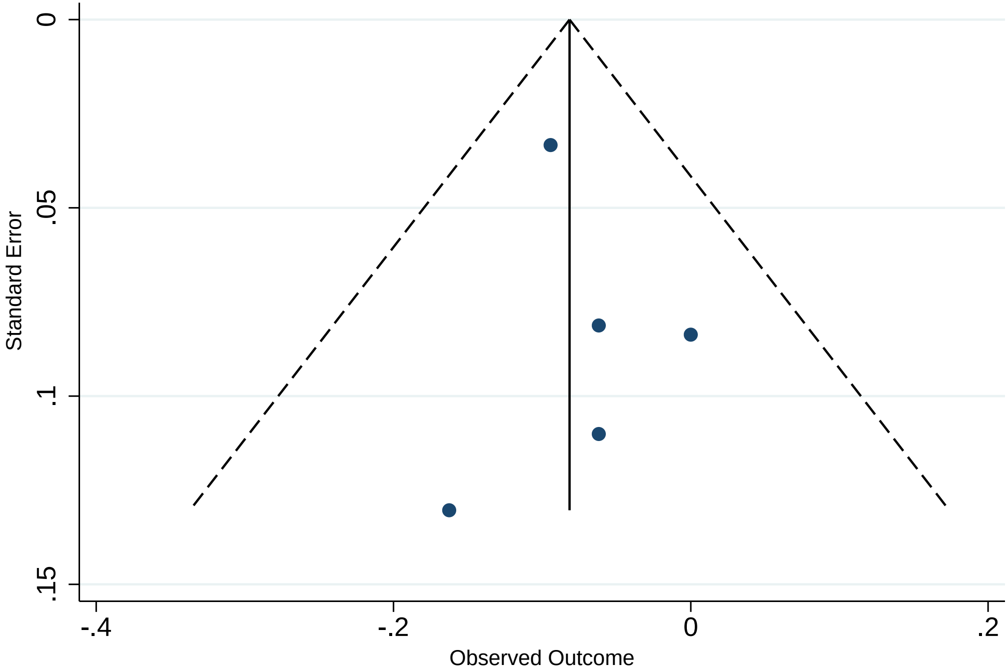

Figure S137. Funnel plot of meta-analysis of the association between carotenoids intake and ALS

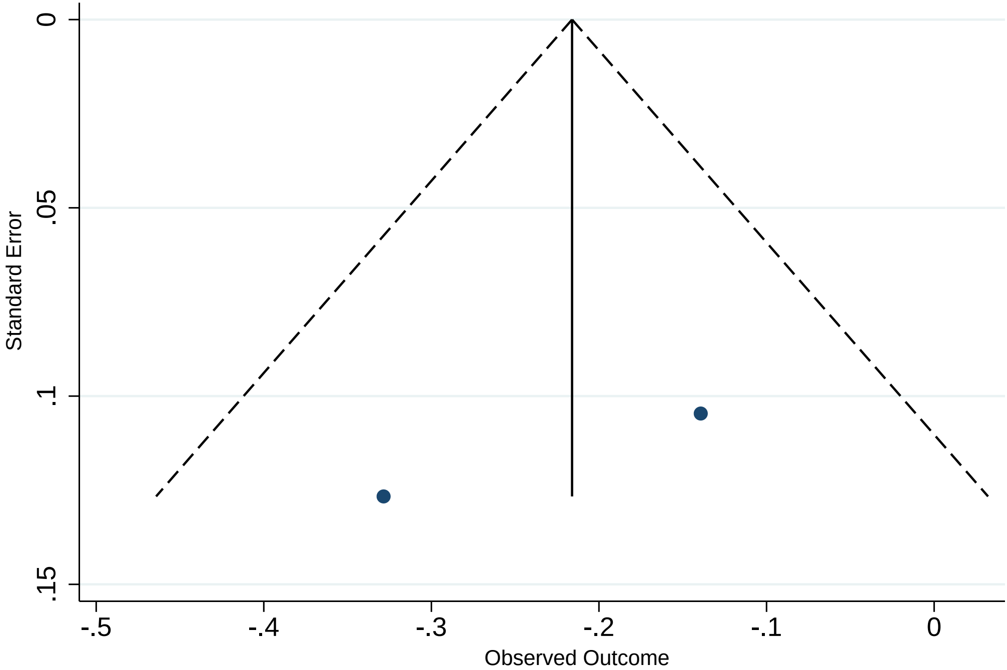

Figure S138. Funnel plot of meta-analysis of the association between acetaminophen and ALS

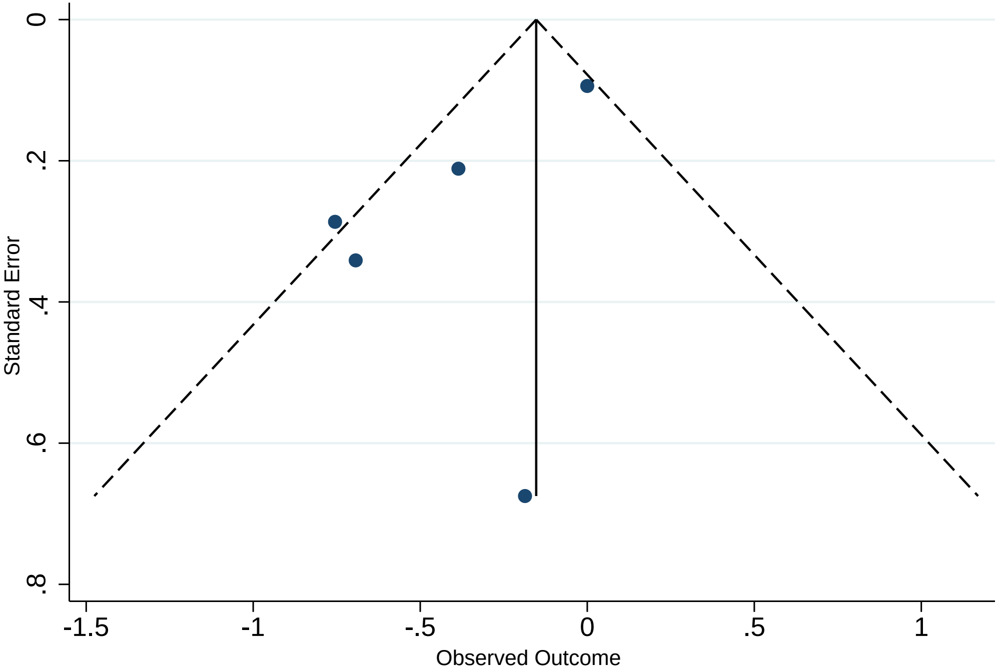

Figure S139. Funnel plot of meta-analysis of the association between living in urban and ALS

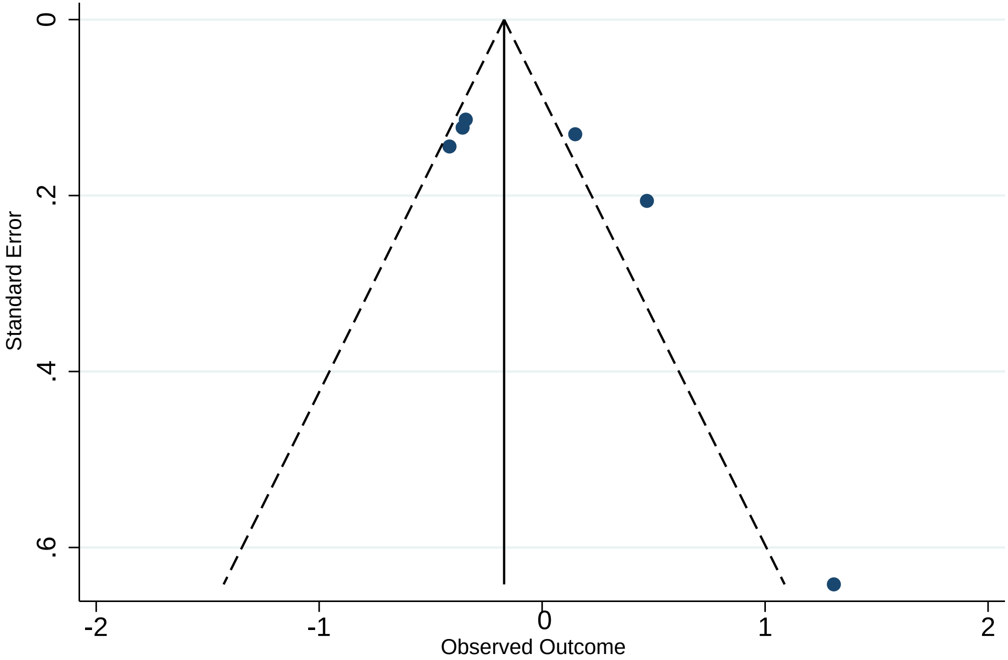

Figure S140. Funnel plot of meta-analysis of the association between welding and ALS

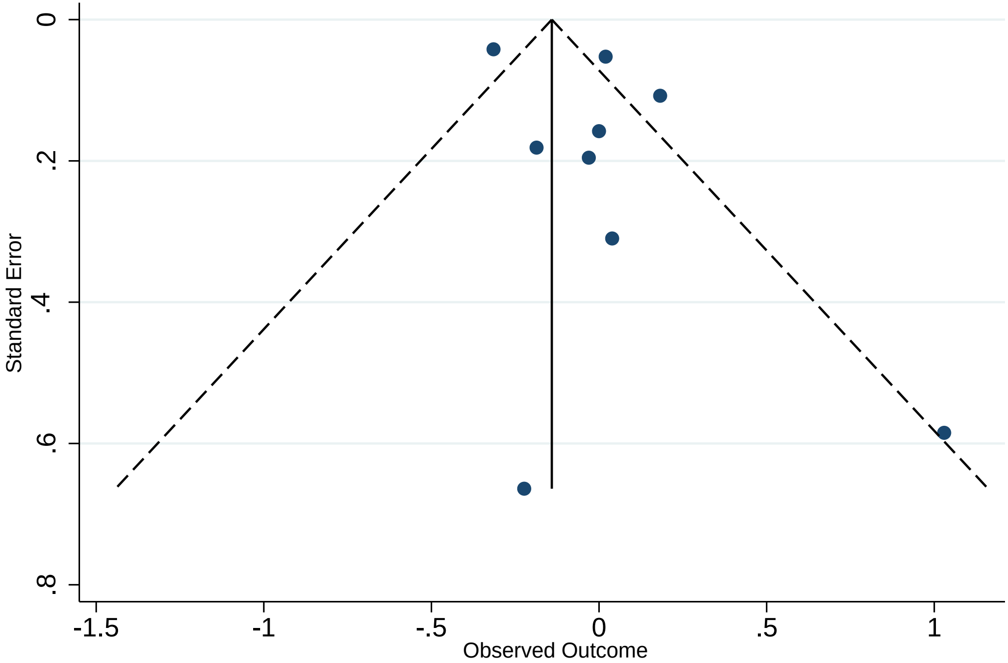

Figure S141. Funnel plot of meta-analysis of the association between electric shocks and ALS

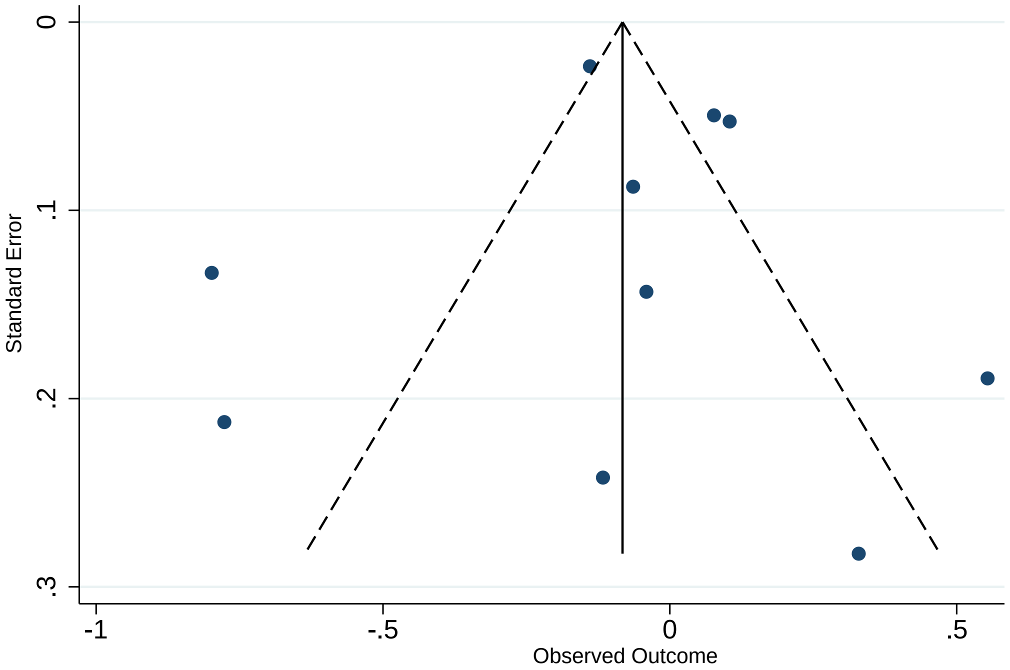

Figure S142. Funnel plot of meta-analysis of the association between statin and ALS

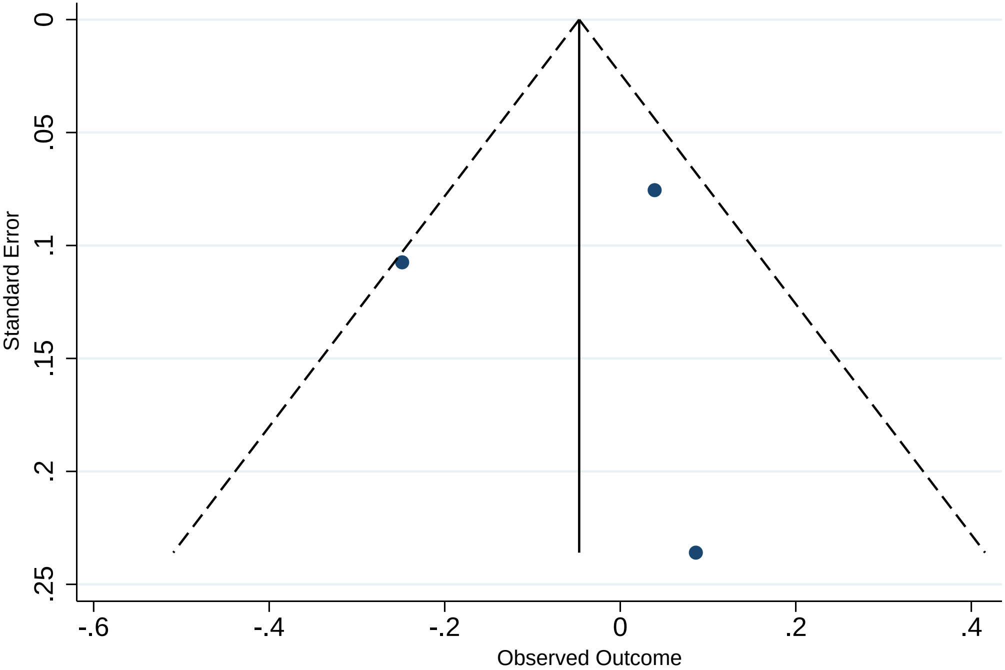

Figure S143. Funnel plot of meta-analysis of the association between aspirin and ALS

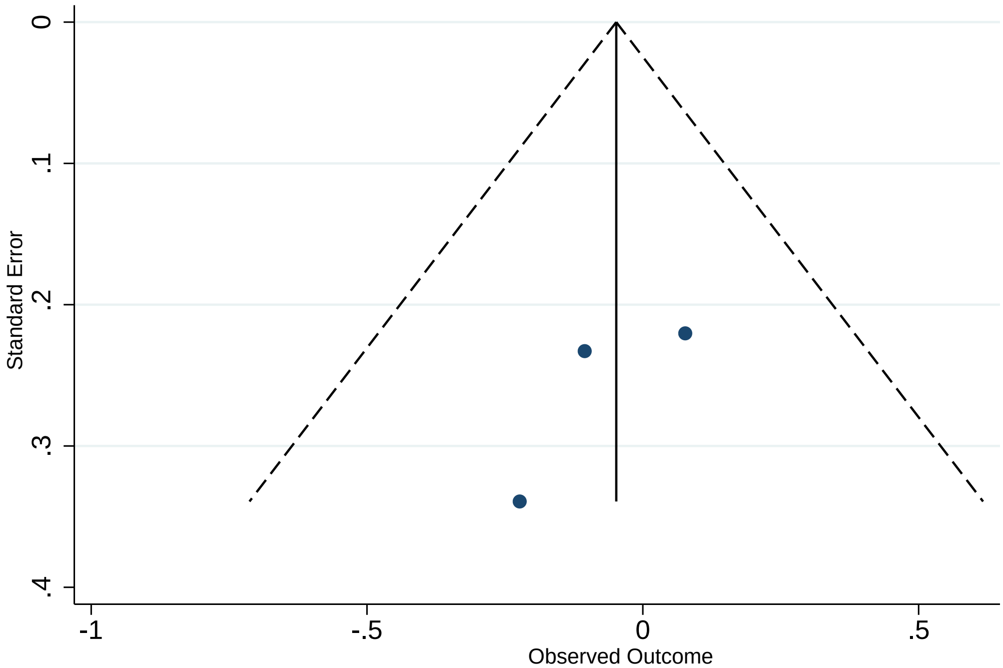

Figure S144. Funnel plot of meta-analysis of the association between high vitamin diet and ALS

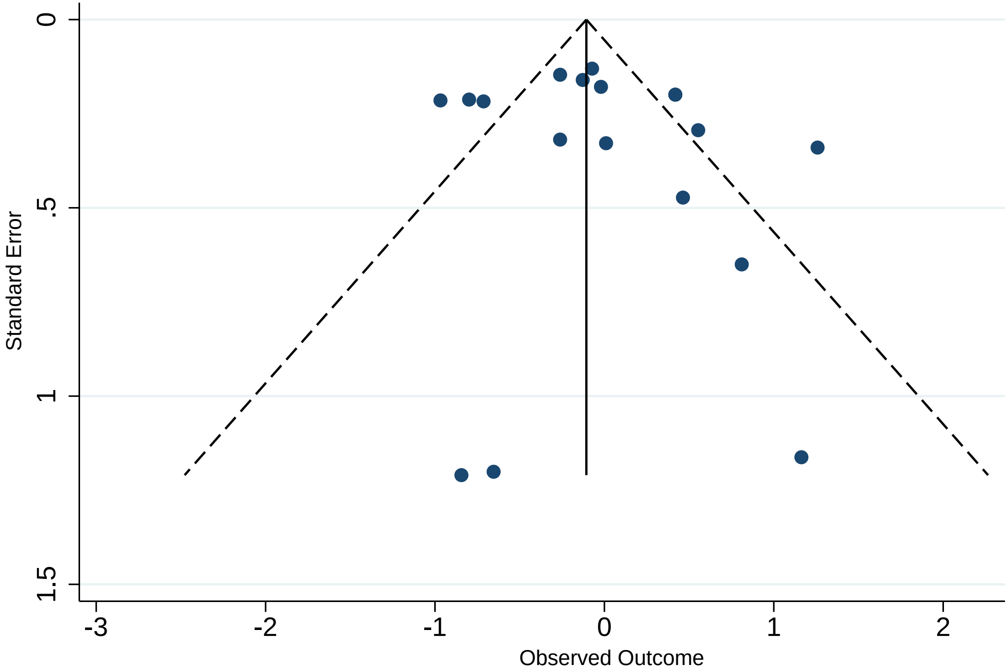

Figure S145. Funnel plot of meta-analysis of the association between sport-related activity and ALS

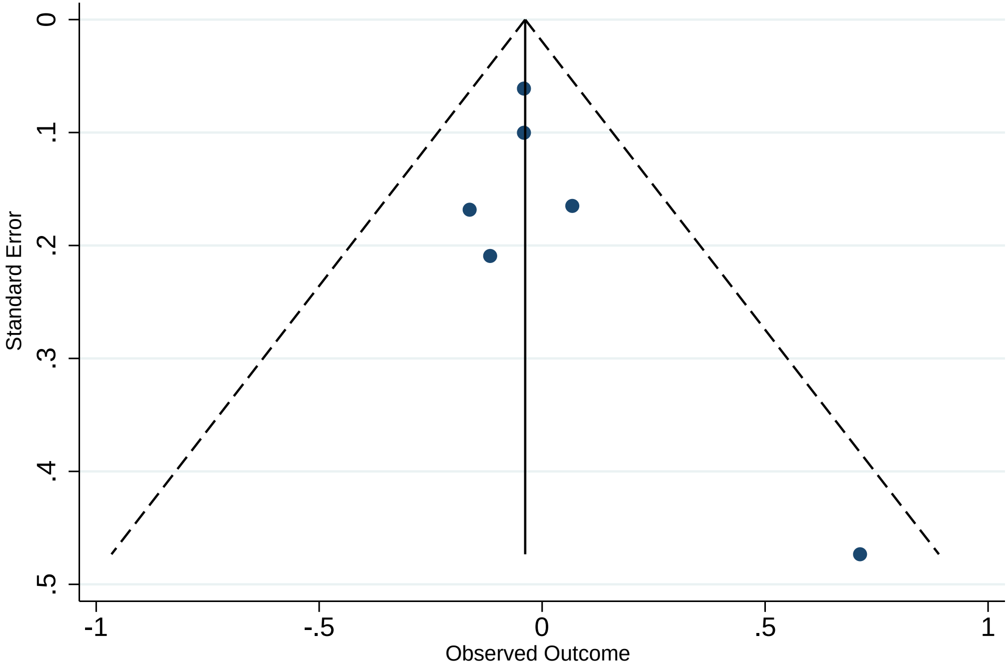

Figure S146. Funnel plot of meta-analysis of the association between AMI/IS and ALS

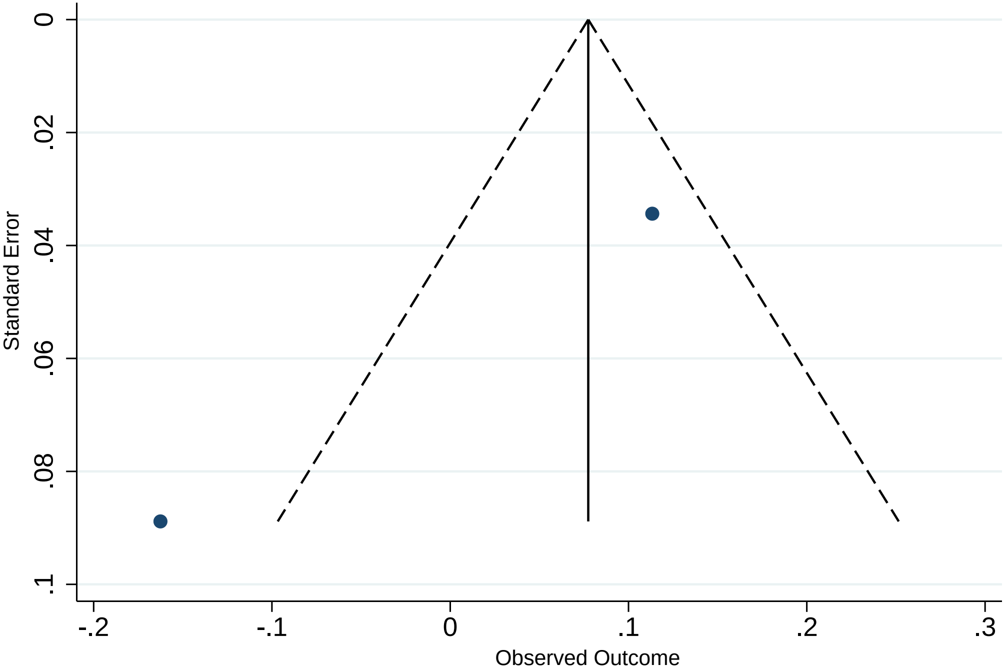

Figure S147. Funnel plot of meta-analysis of the association between cerebrovascular disease and ALS

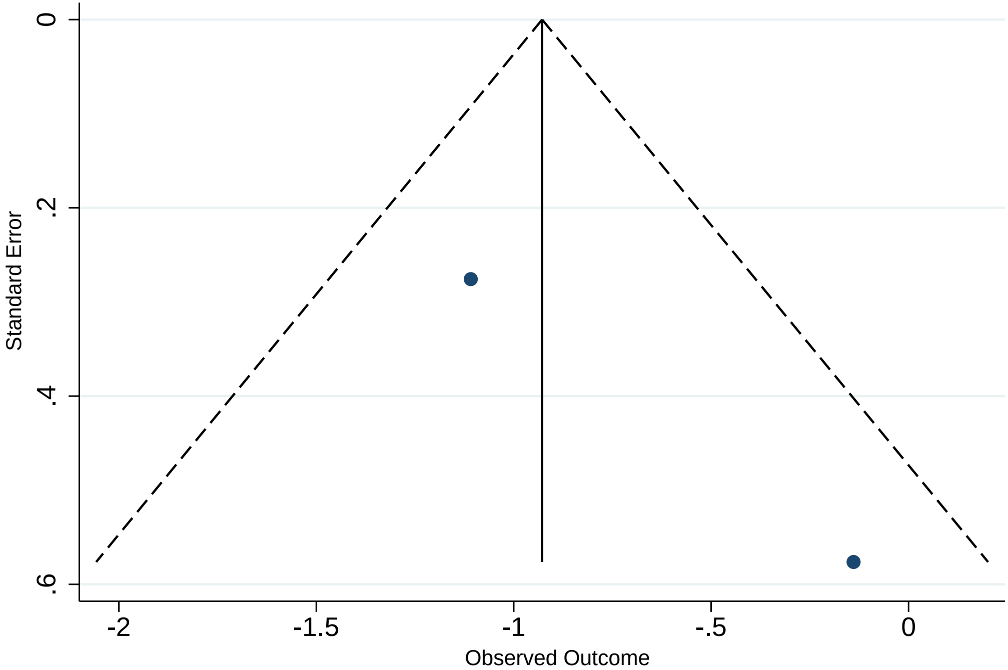

Figure S148. Funnel plot of meta-analysis of the association between occupation in service industry and ALS

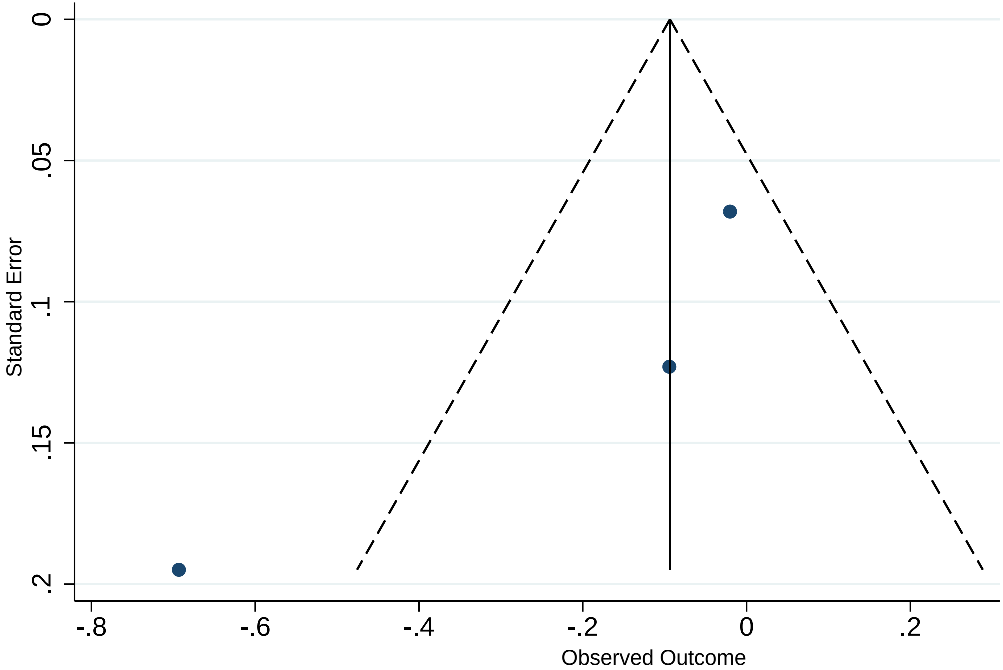

Figure S149. Funnel plot of meta-analysis of the association between coffee consumption and ALS

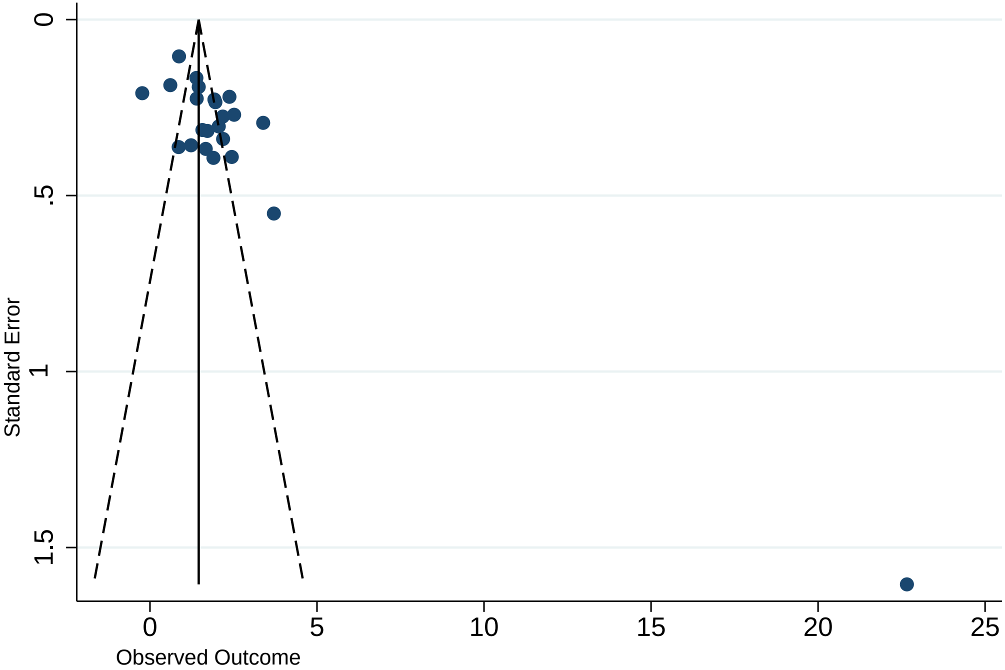

Figure S150. Funnel plot of meta-analysis of the association between CSF NFL level and ALS

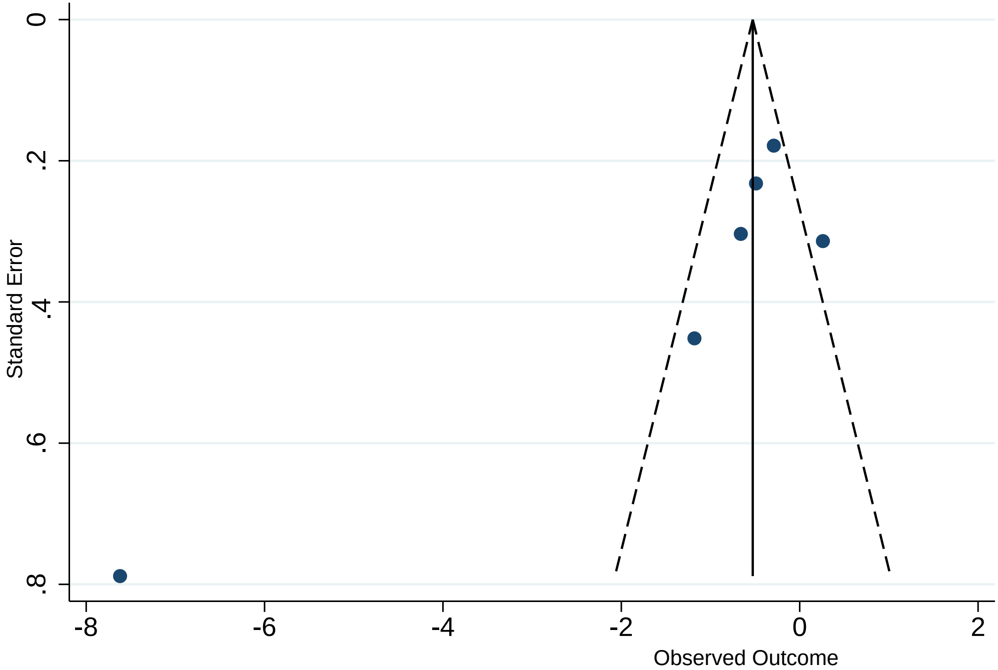

Figure S151. Funnel plot of meta-analysis of the association between CSF Cystatin C level and ALS

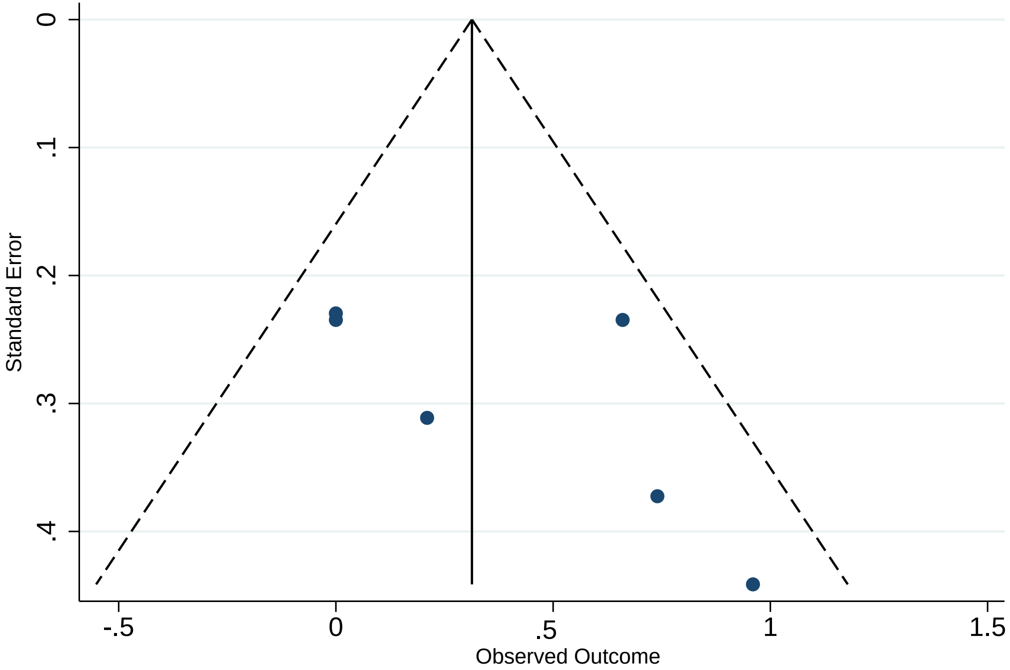

Figure S152. Funnel plot of meta-analysis of the association between CSF TNF- α level and ALS

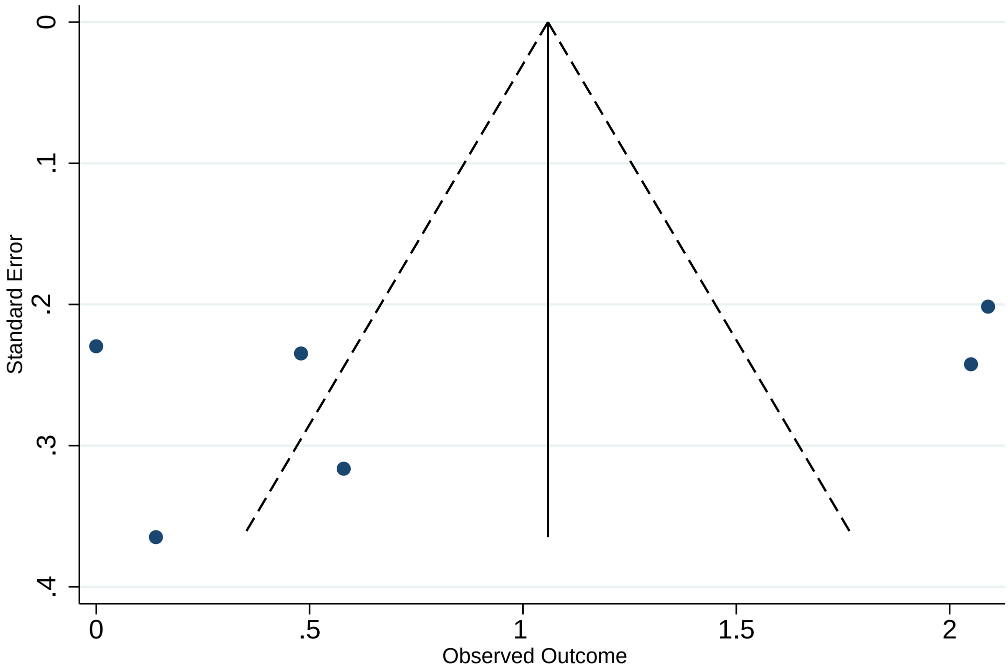

Figure S153. Funnel plot of meta-analysis of the association between CSF MIP-1 α level and ALS

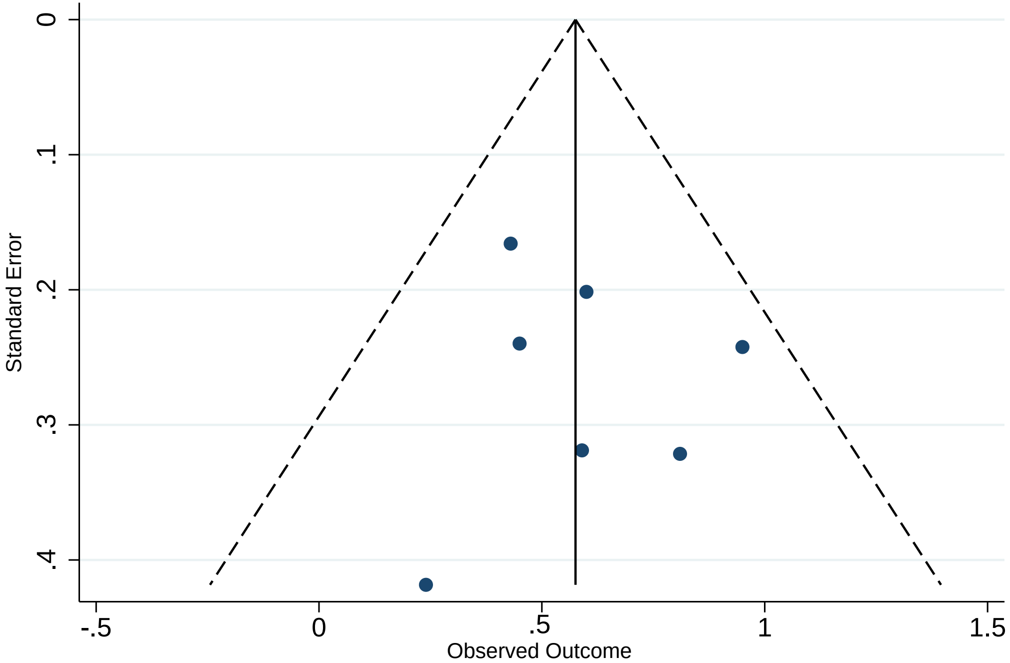

Figure S154. Funnel plot of meta-analysis of the association between CSF MCP-1 level and ALS

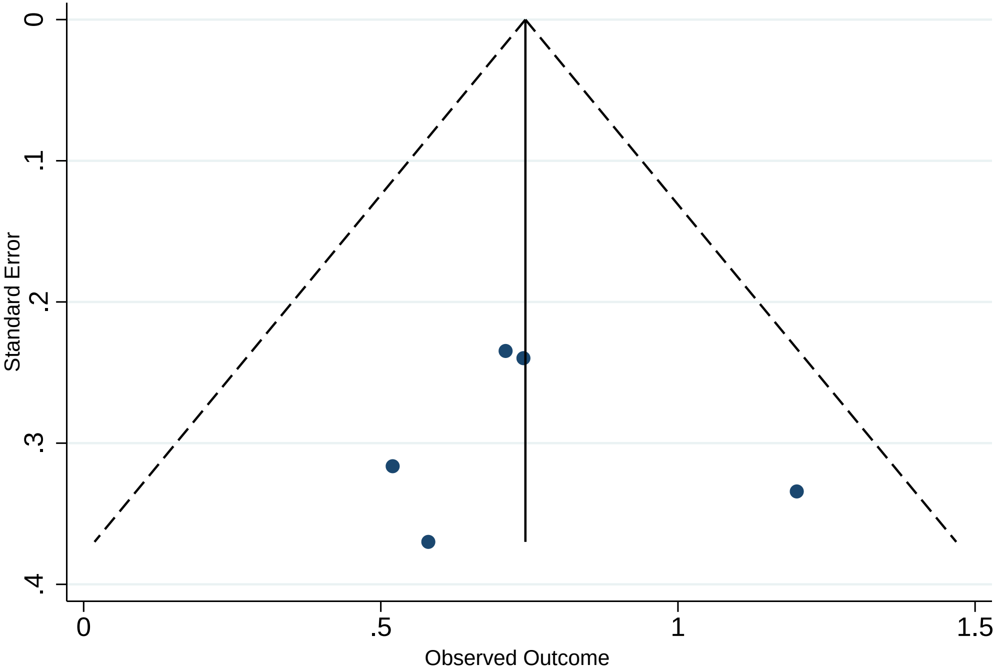

Figure S155. Funnel plot of meta-analysis of the association between CSF IL-17 level and ALS

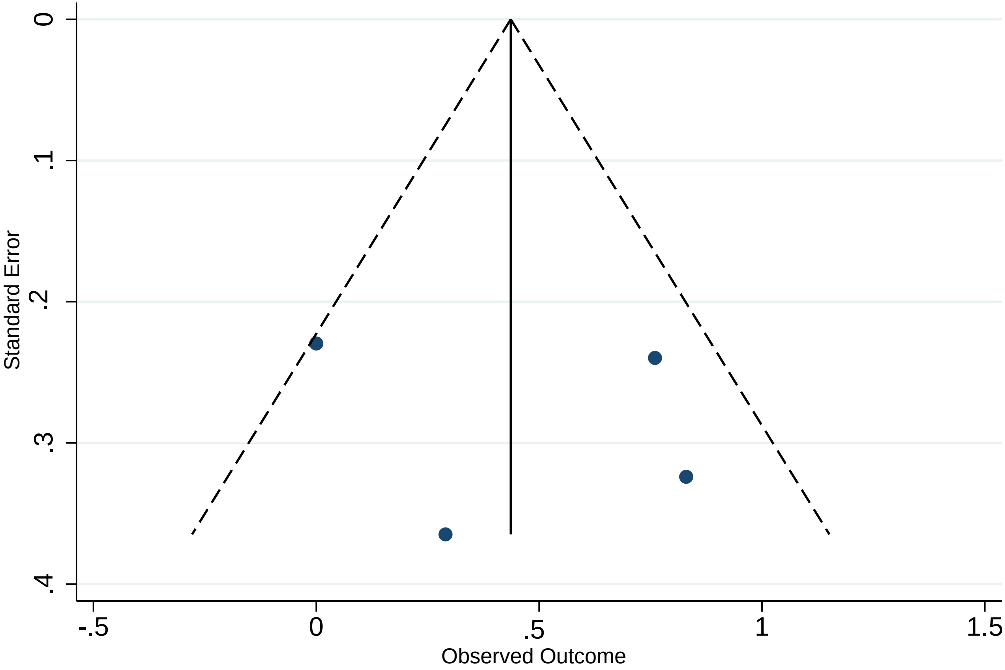

Figure S156. Funnel plot of meta-analysis of the association between CSF IL-15 level and ALS

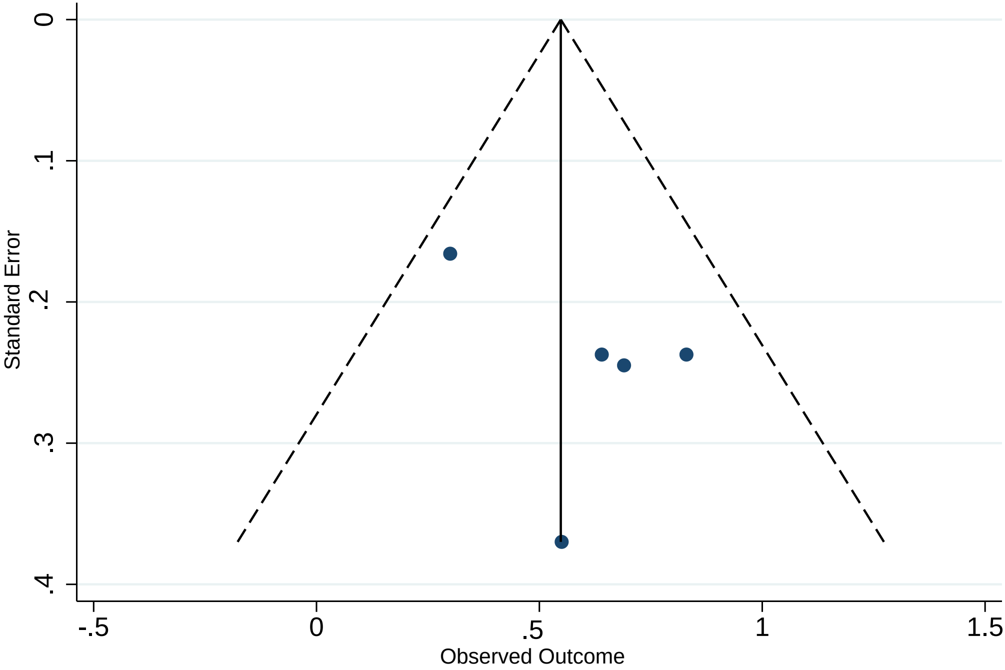

Figure S157. Funnel plot of meta-analysis of the association between CSF G-CSF level and ALS

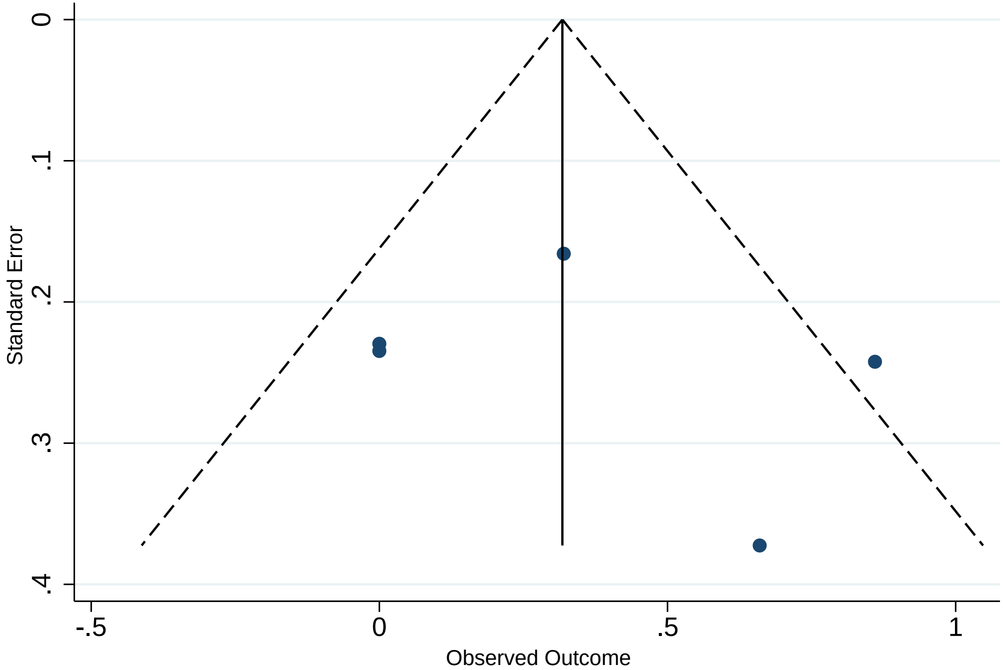

Figure S158. Funnel plot of meta-analysis of the association between IL-2 level and ALS

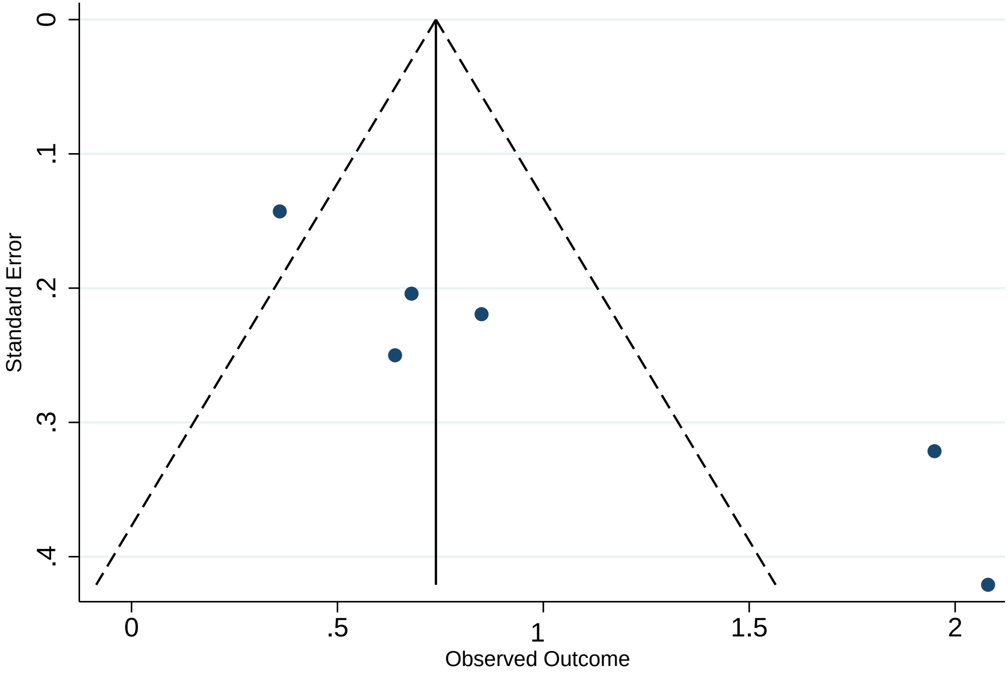

Figure S159. Funnel plot of meta-analysis of the association between CSF NFH level and ALS

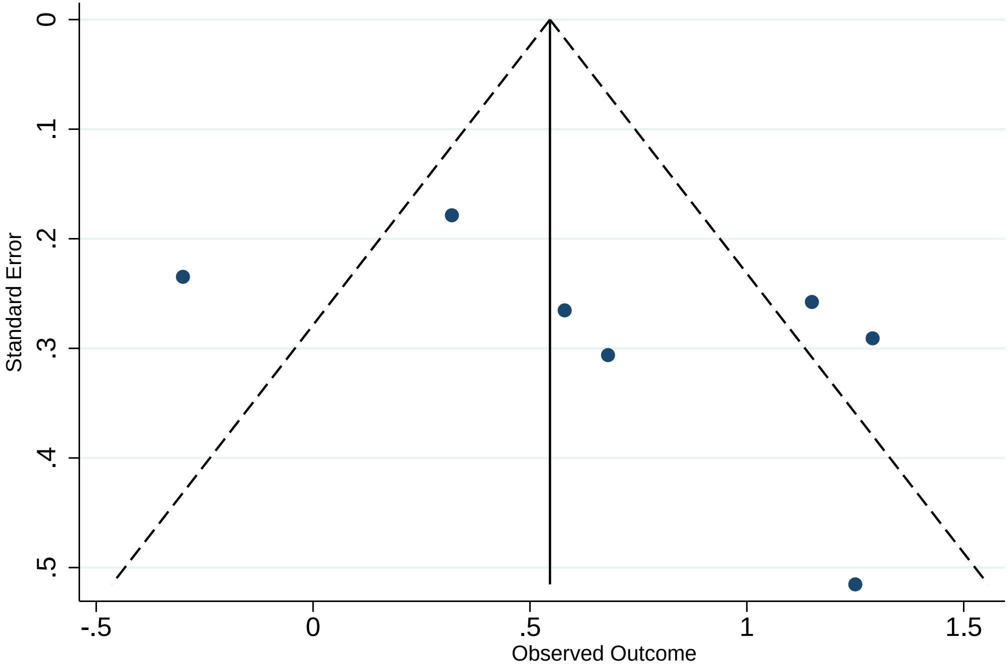

Figure S160. Funnel plot of meta-analysis of the association between CSF TDP-43 level and ALS

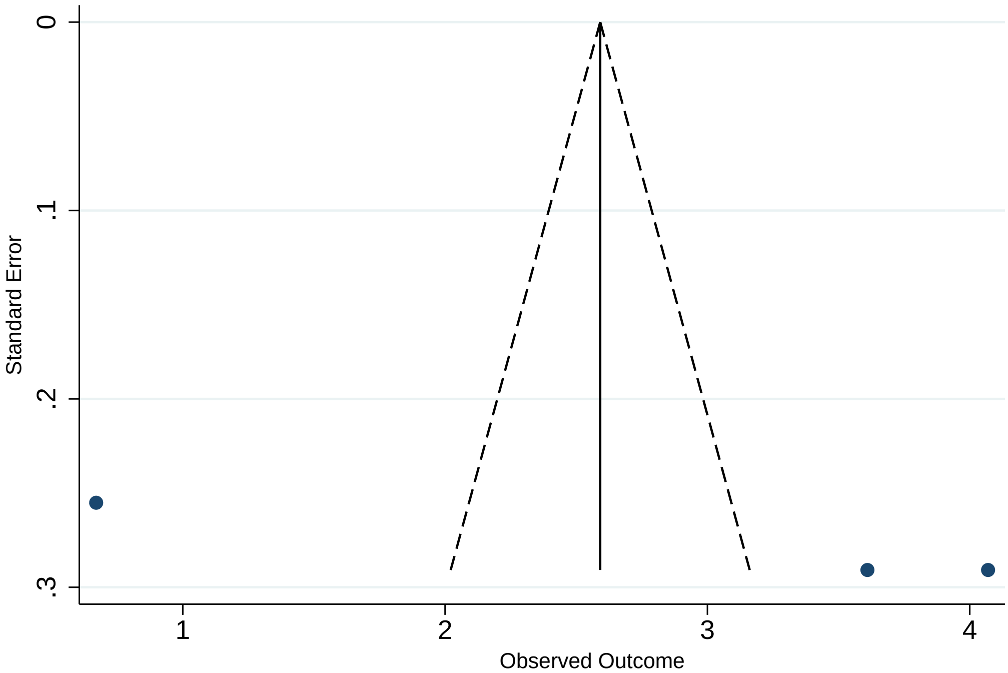

Figure S161. Funnel plot of meta-analysis of the association between CSF homocysteine level and ALS

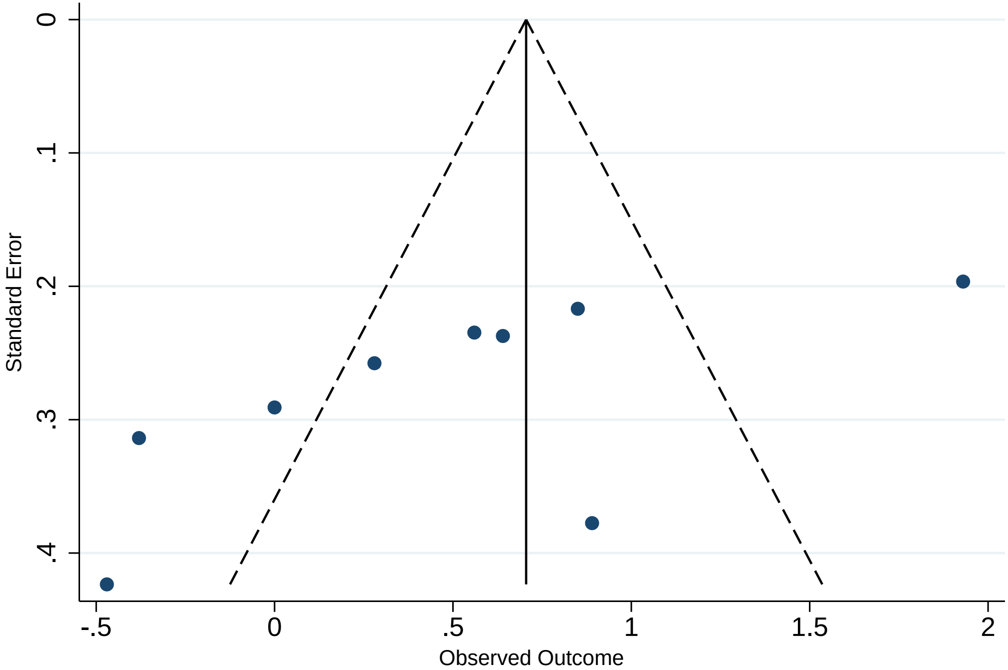

Figure S162. Funnel plot of meta-analysis of the association between CSF VEGF level and

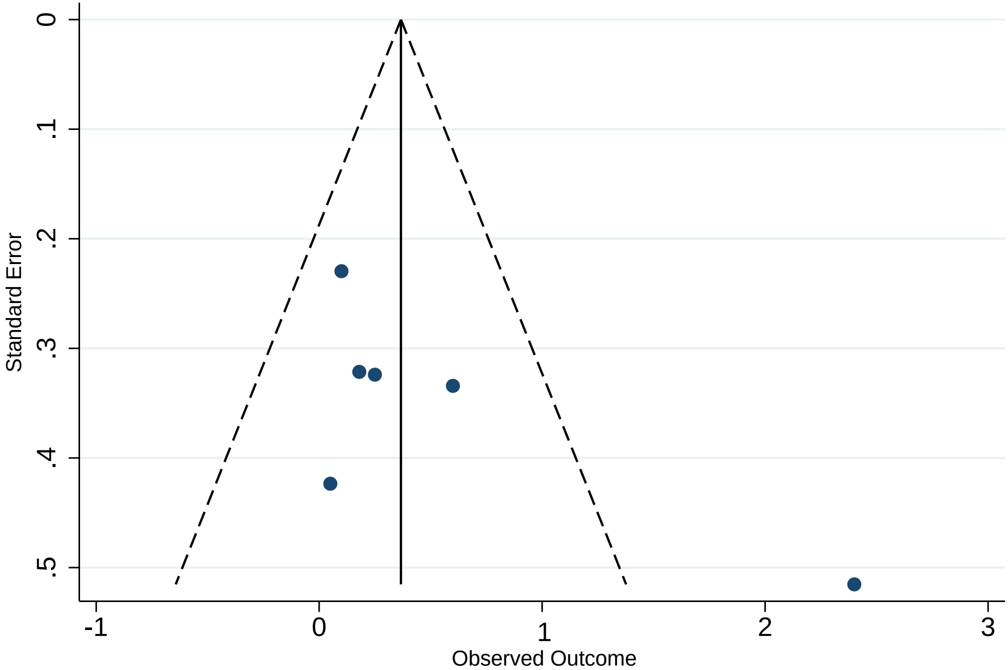

Figure S163. Funnel plot of meta-analysis of the association between CSF lead level and ALS

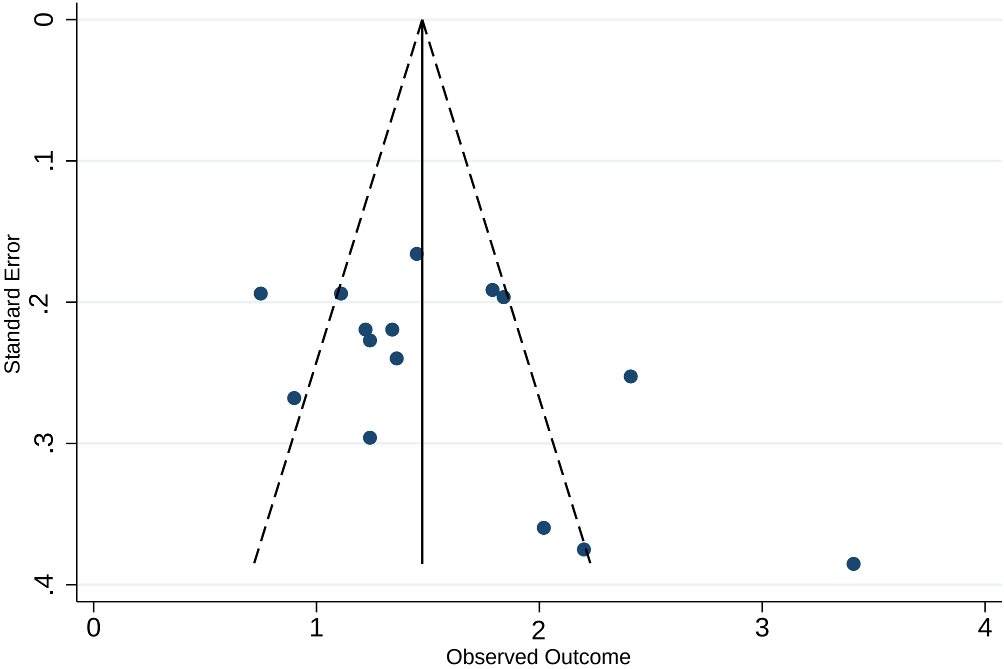

Figure S164. Funnel plot of meta-analysis of the association between serum NFL level and ALS

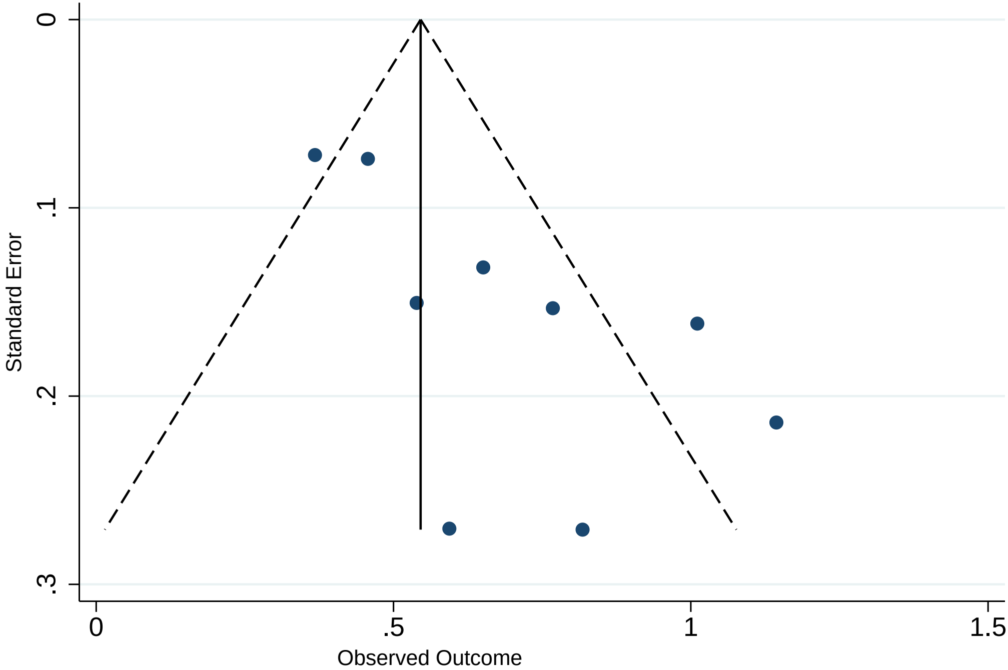

Figure S165. Funnel plot of meta-analysis of the association between serum feritin level and ALS

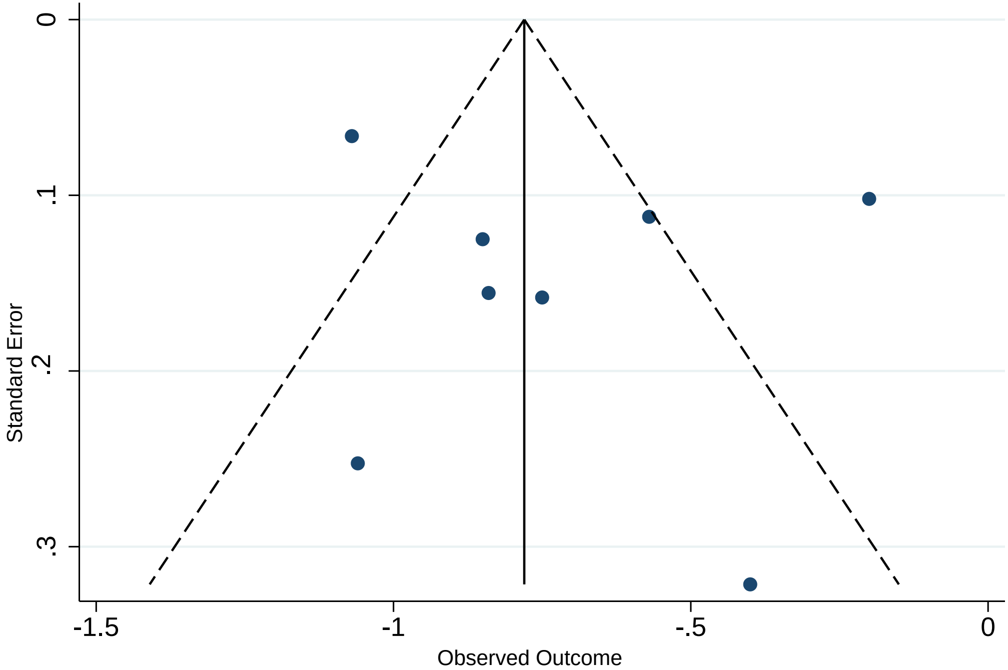

Figure S166. Funnel plot of meta-analysis of the association between serum uric acid level and ALS

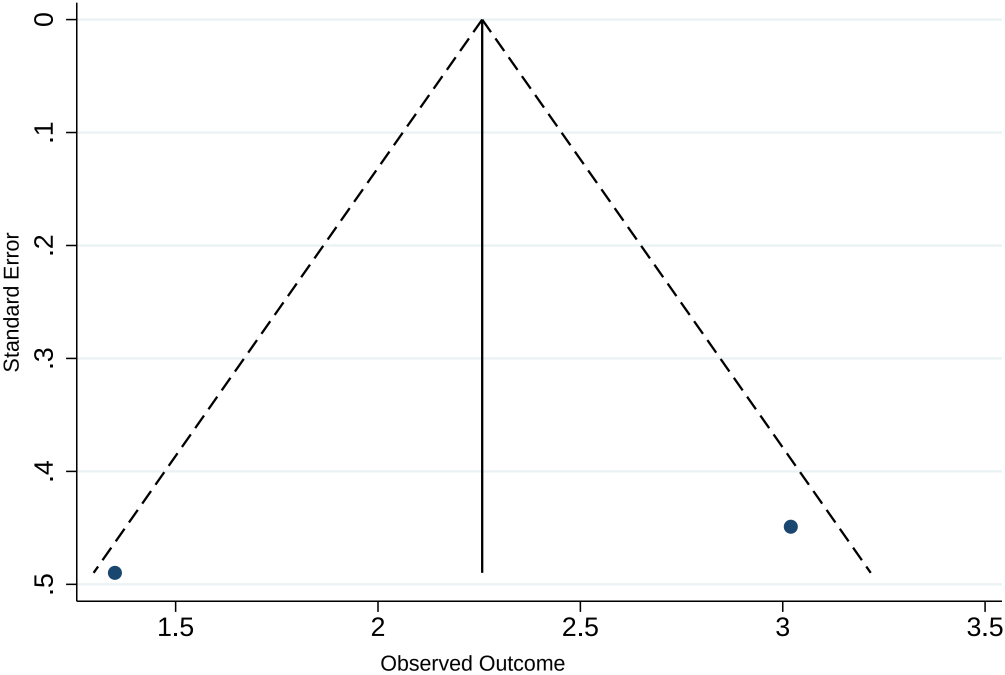

Figure S167. Funnel plot of meta-analysis of the association between serum 8-OHdG level and ALS

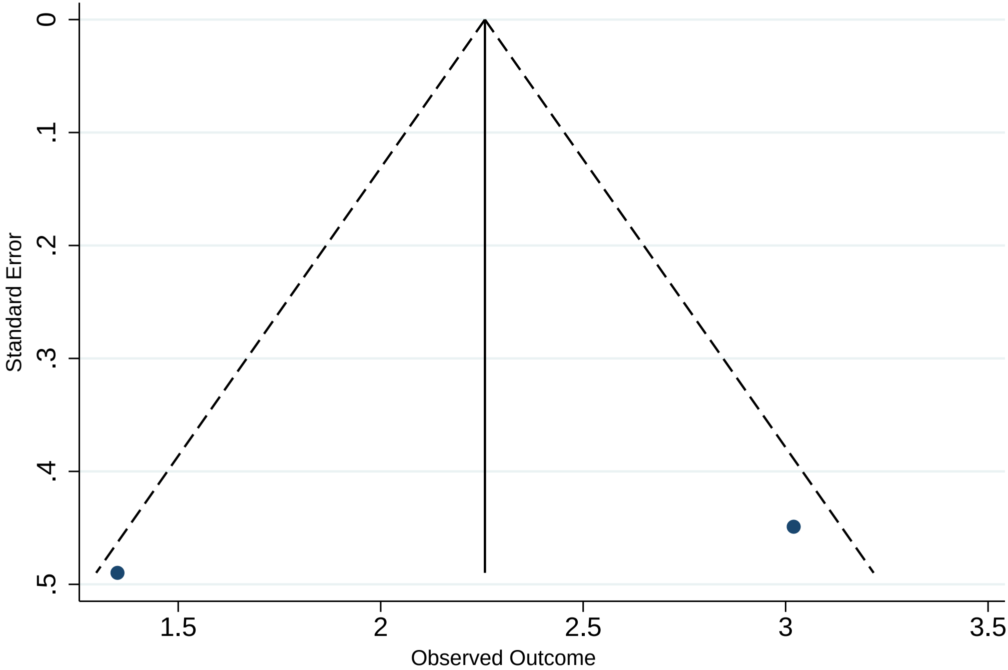

Figure S168. Funnel plot of meta-analysis of the association between serum GSH level and ALS

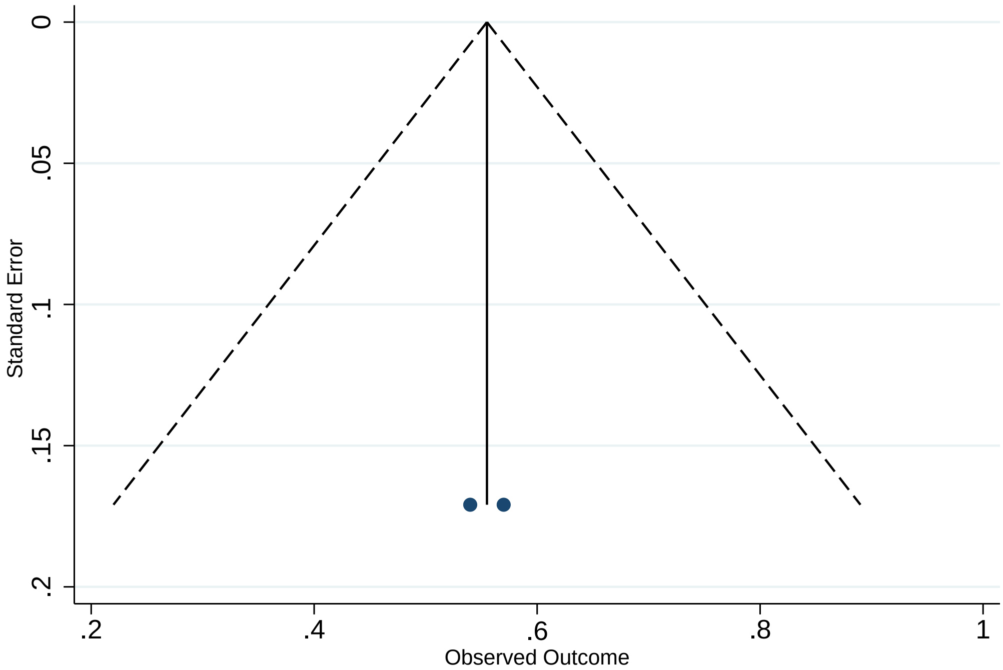

Figure S169. Funnel plot of meta-analysis of the association between serum AOPP level and ALS

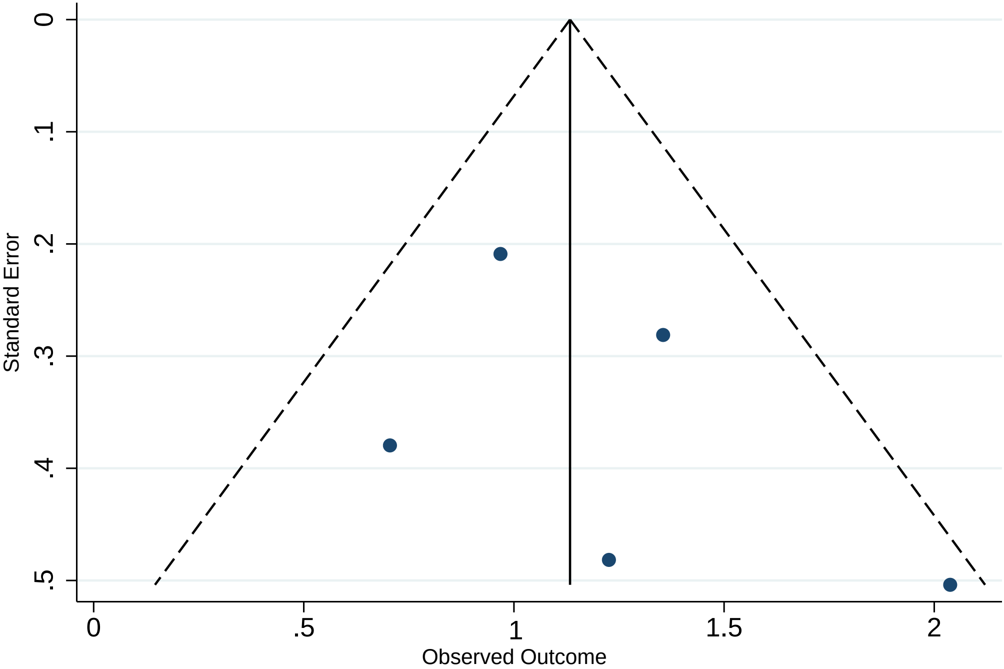

Figure S170. Funnel plot of meta-analysis of the association between serum MDA level and ALS

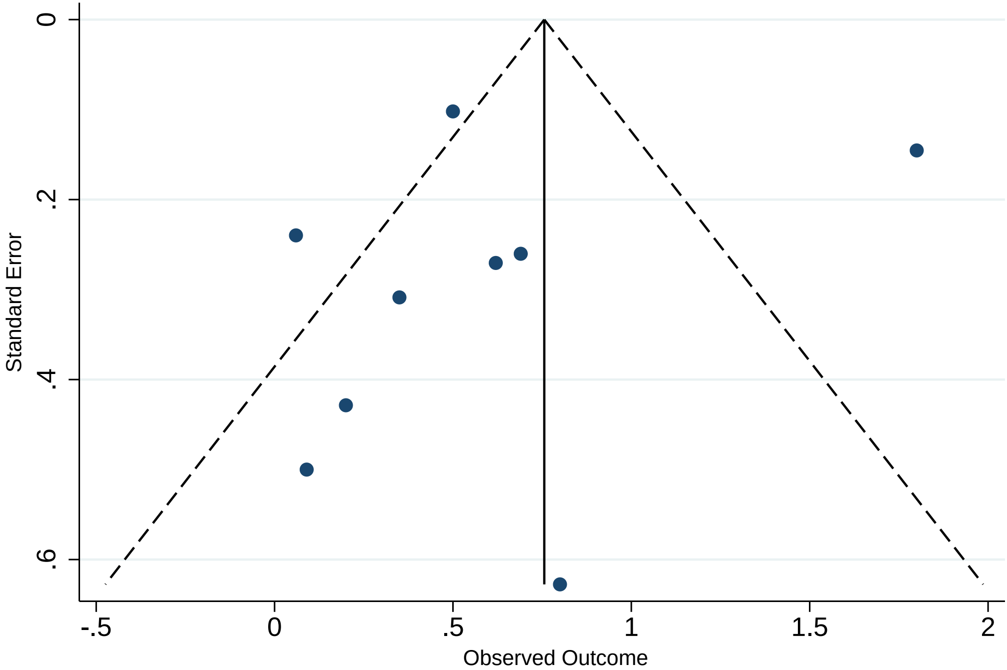

Figure S171. Funnel plot of meta-analysis of the association between serum lead level and ALS

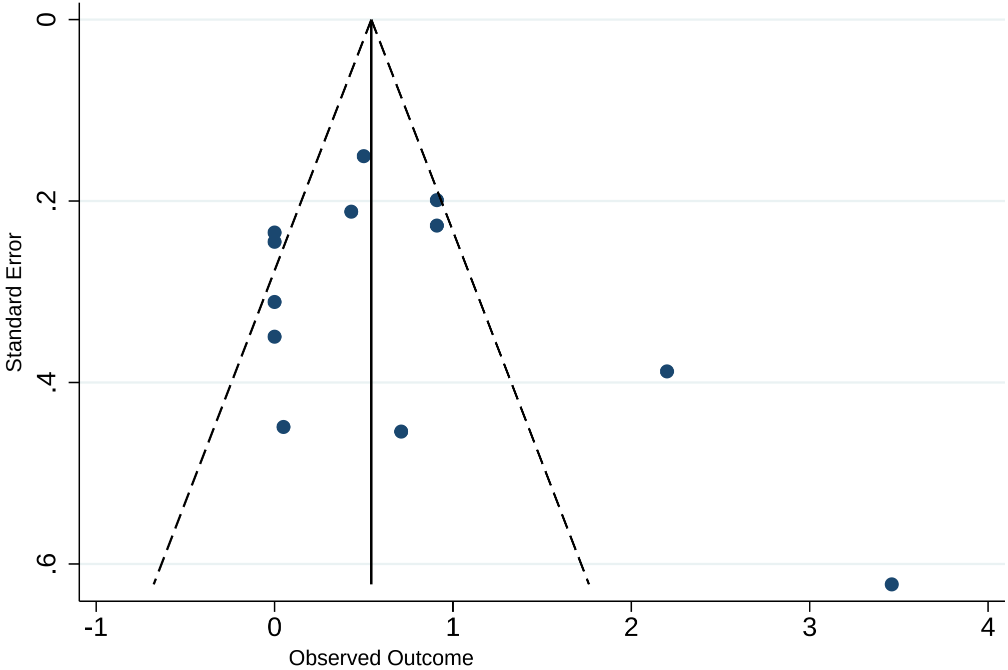

Figure S172. Funnel plot of meta-analysis of the association between serum TNF- α level and ALS

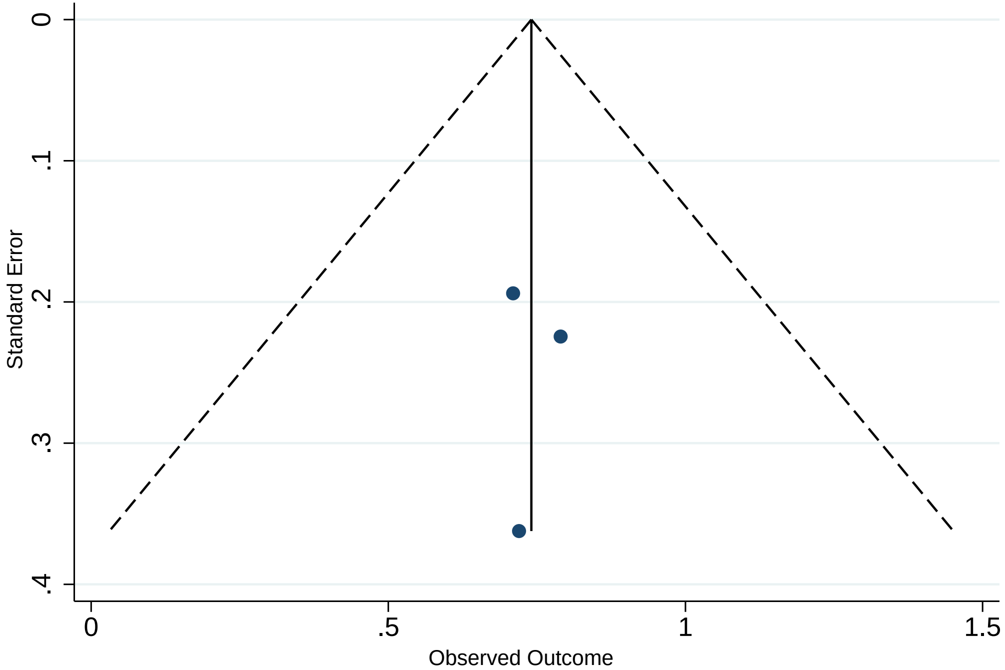

Figure S173. Funnel plot of meta-analysis of the association between serum TNFR1 level and ALS

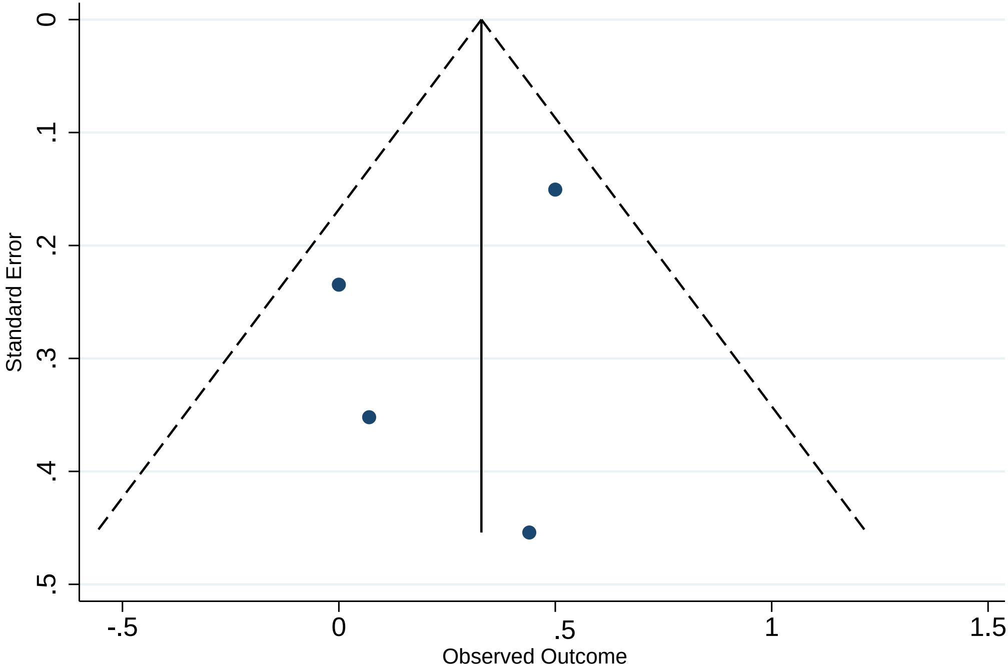

Figure S174. Funnel plot of meta-analysis of the association between serum IL-1β level and ALS

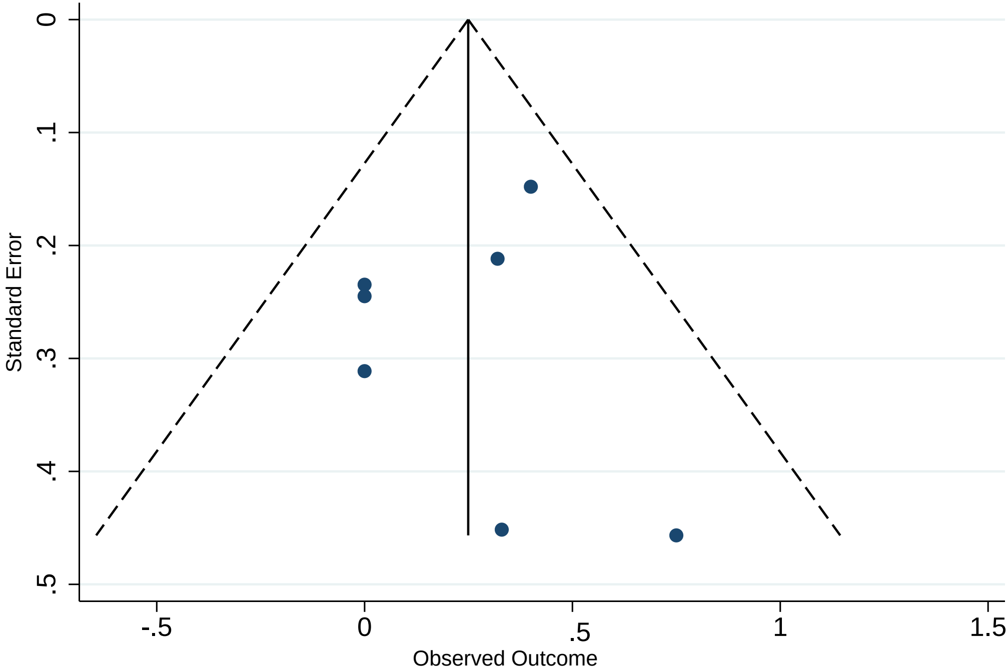

Figure S175. Funnel plot of meta-analysis of the association between serum IL-6 level and ALS

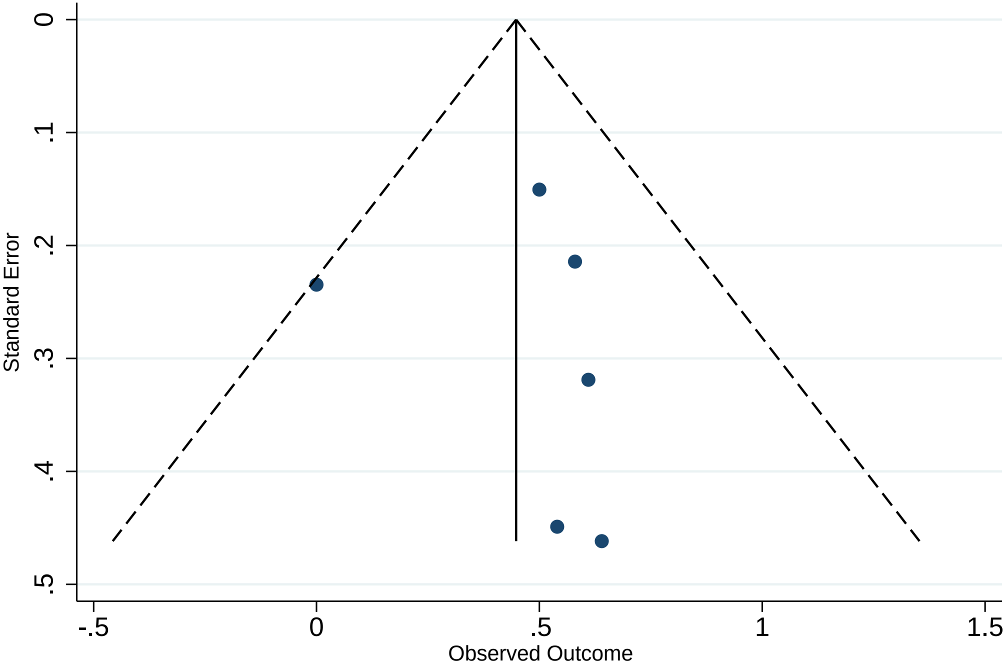

Figure S176. Funnel plot of meta-analysis of the association between serum IL-8 level and ALS

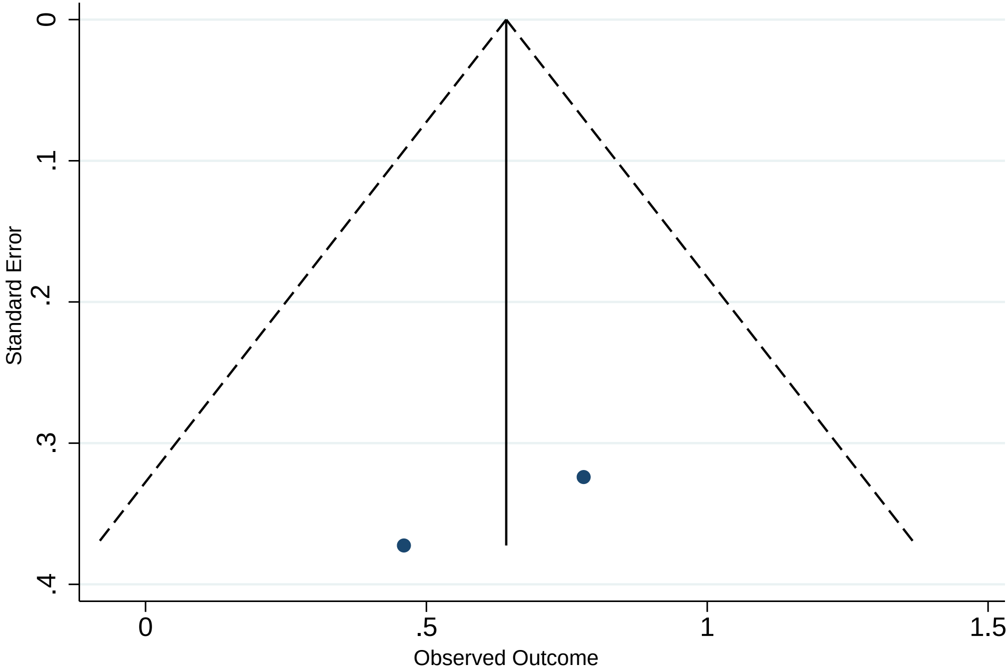

Figure S177. Funnel plot of meta-analysis of the association between serum IL-17 level and ALS

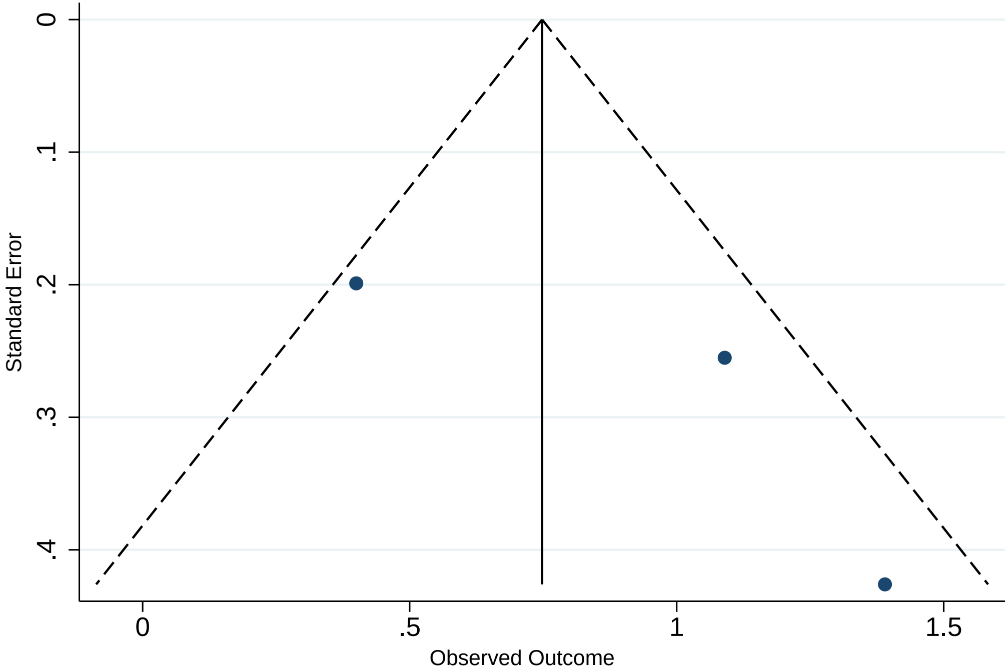

Figure S178. Funnel plot of meta-analysis of the association between serum VEGF level and ALS

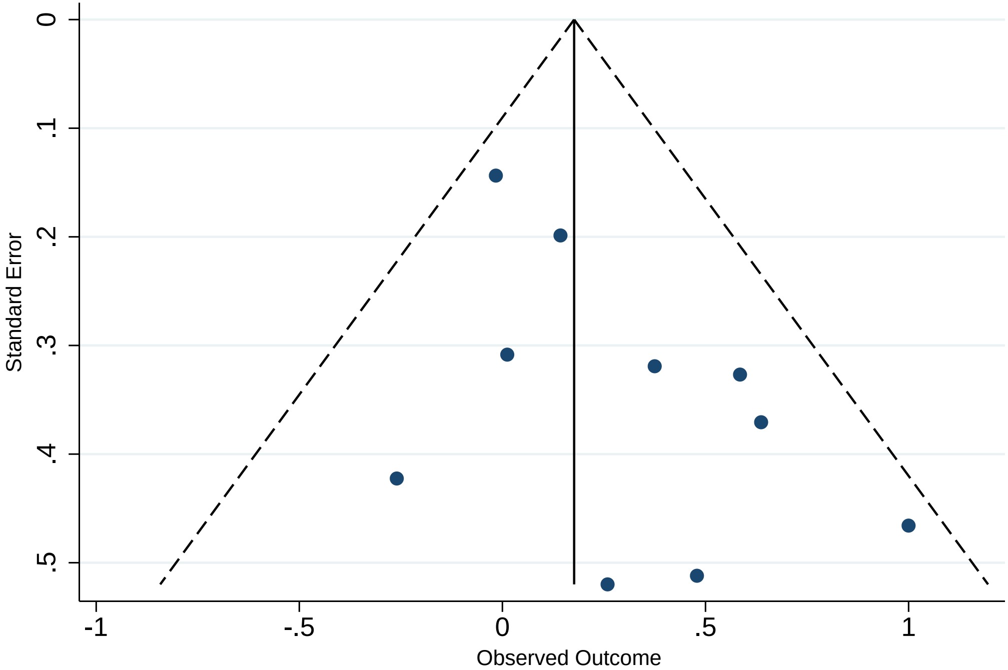

Figure S179. Funnel plot of meta-analysis of the association between serum FBG level and ALS

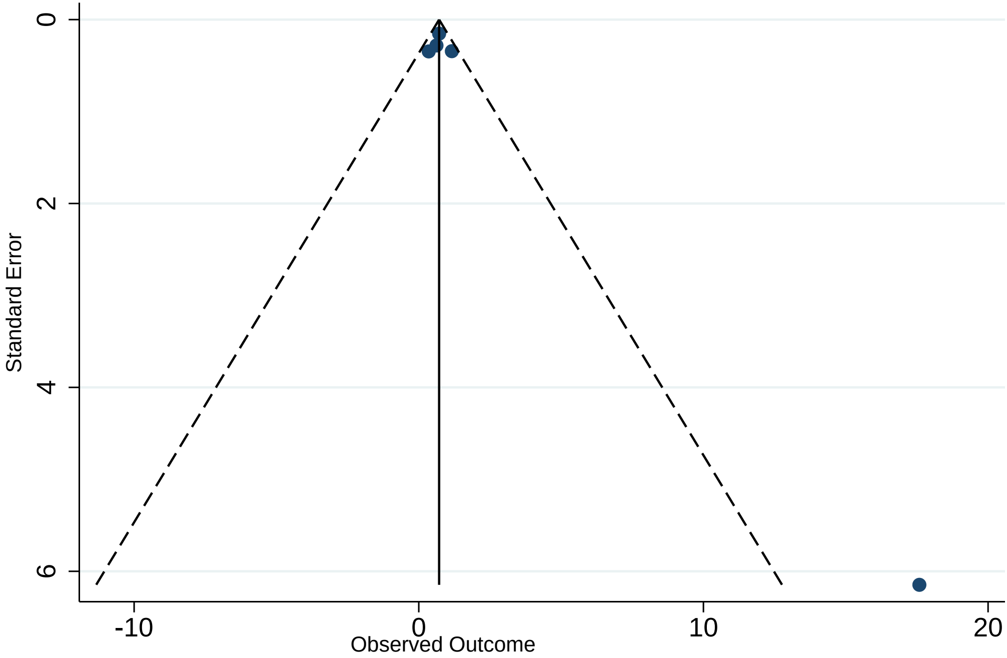

Figure S180. Funnel plot of meta-analysis of the association between serum CK level and ALS

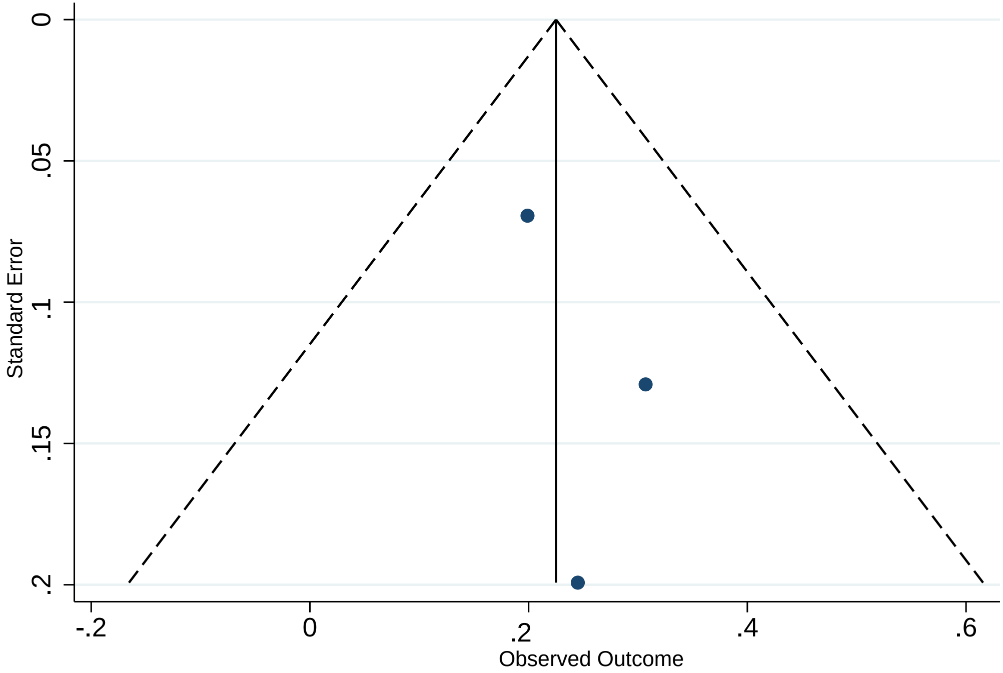

Figure S181. Funnel plot of meta-analysis of the association between serum TSC level and ALS

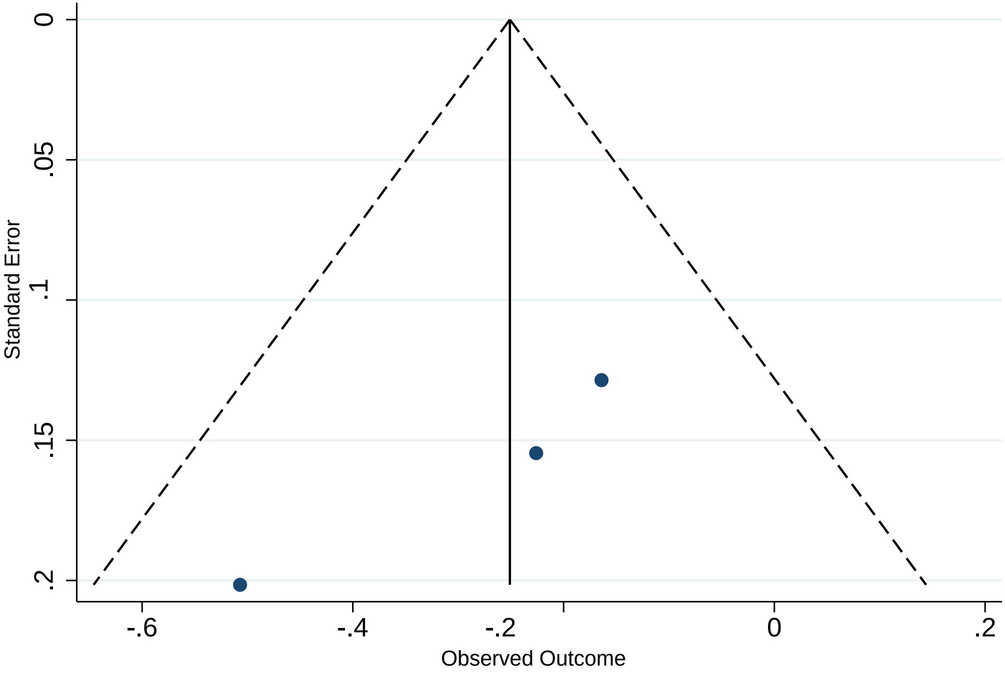

Figure S182. Funnel plot of meta-analysis of the association between serum TIBC level and ALS

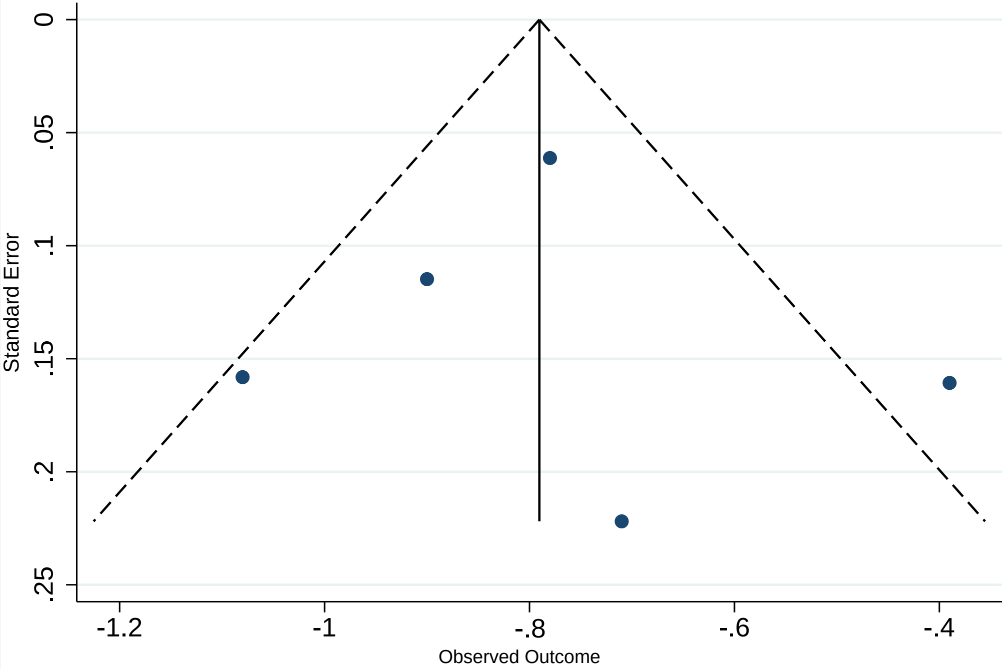

Figure S183. Funnel plot of meta-analysis of the association between serum creatinine level and ALS

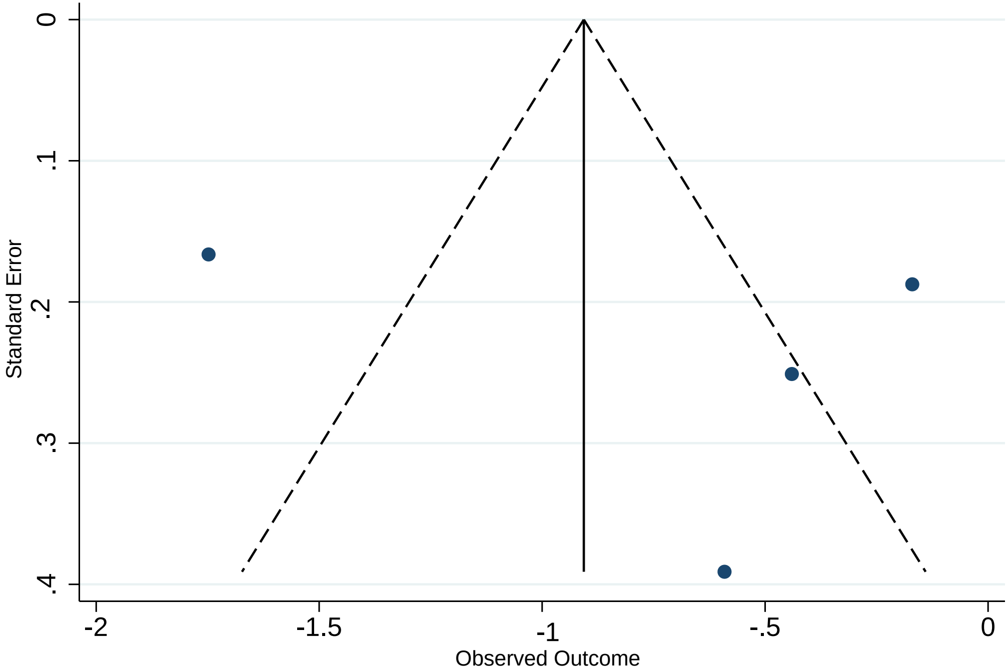

Figure S184. Funnel plot of meta-analysis of the association between serum vitamin D level and ALS

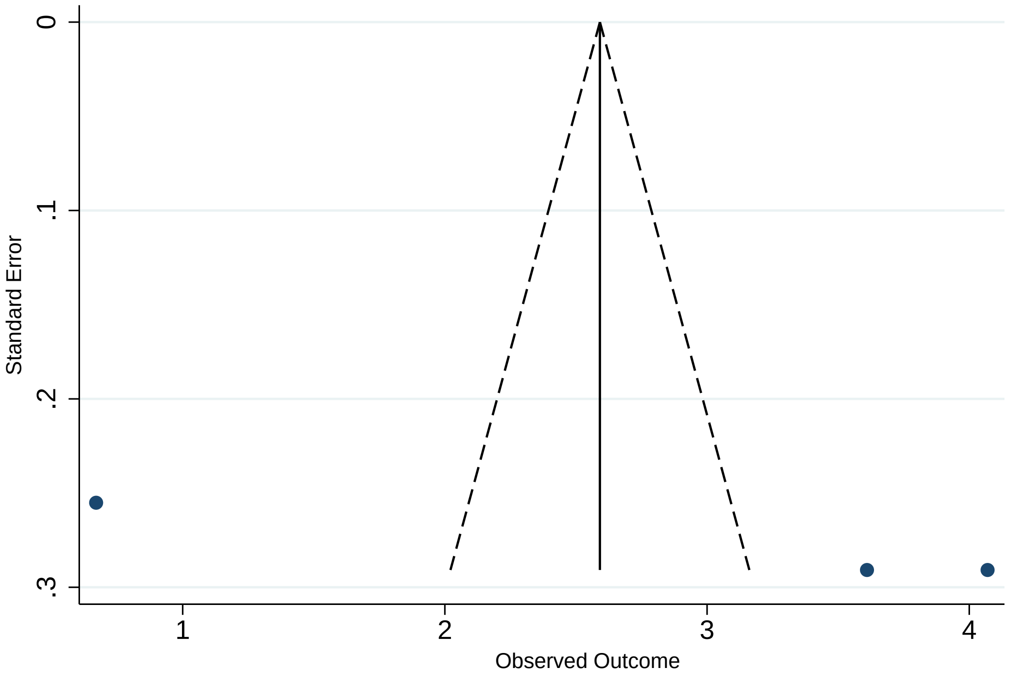

Figure S185. Funnel plot of meta-analysis of the association between serum folic level and ALS

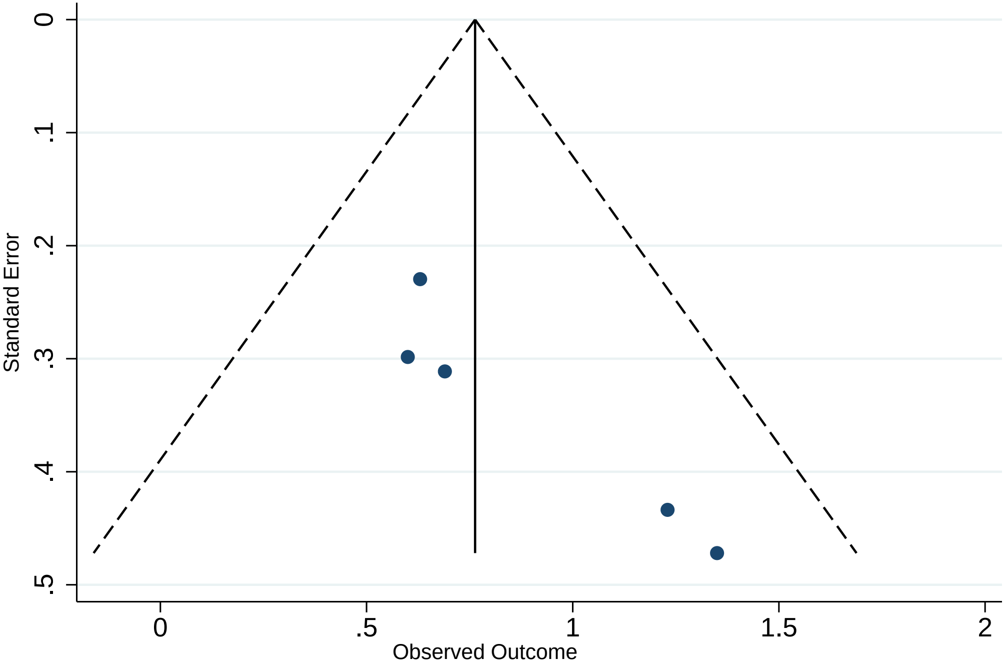

Figure S186. Funnel plot of meta-analysis of the association between serum miR-206 level and ALS

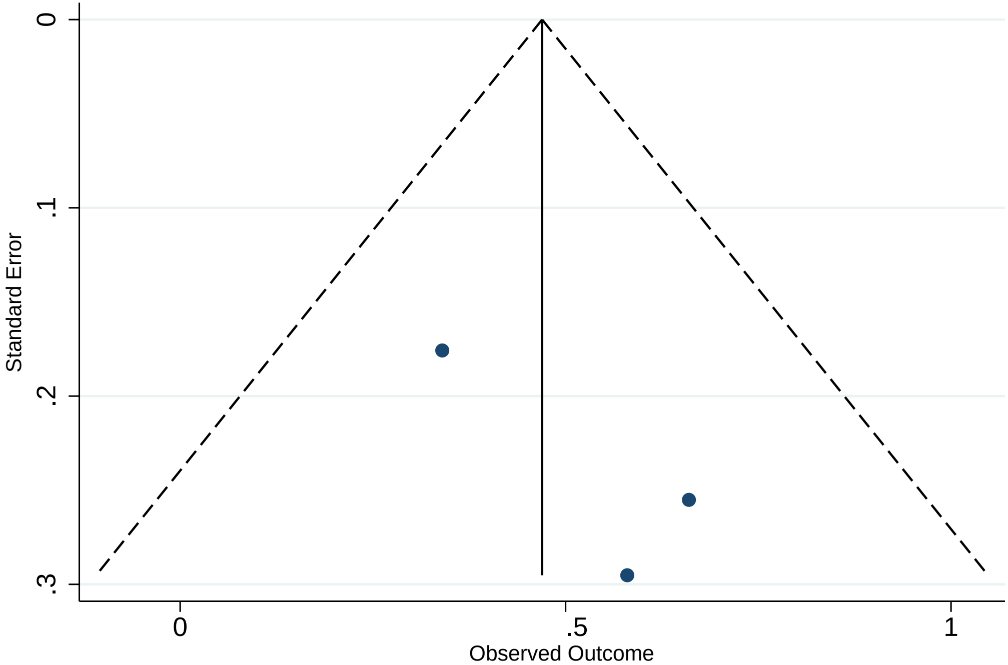

Figure S187. Funnel plot of meta-analysis of the association between serum miR-338-3p level and ALS

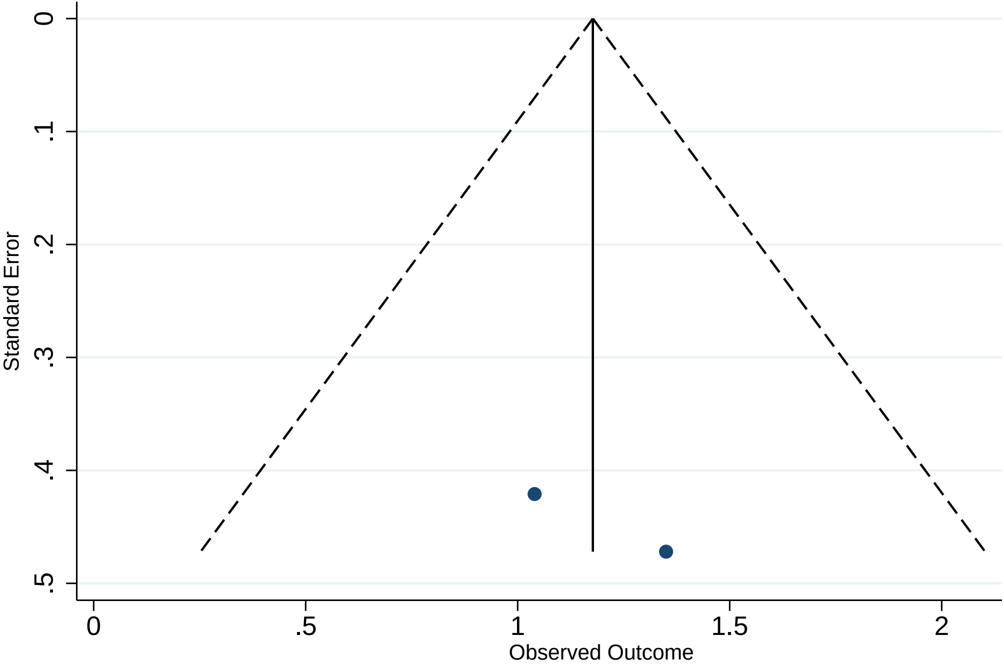

Figure S188. Funnel plot of meta-analysis of the association between serum miR-133b level and ALS

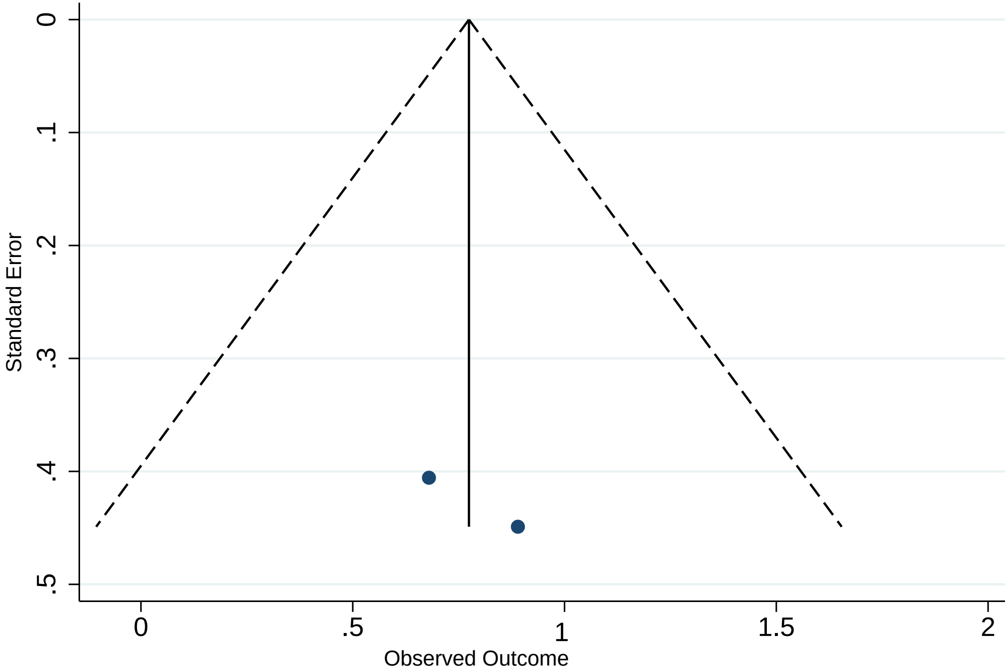

Figure S189. Funnel plot of meta-analysis of the association between serum miR-133a level and ALS

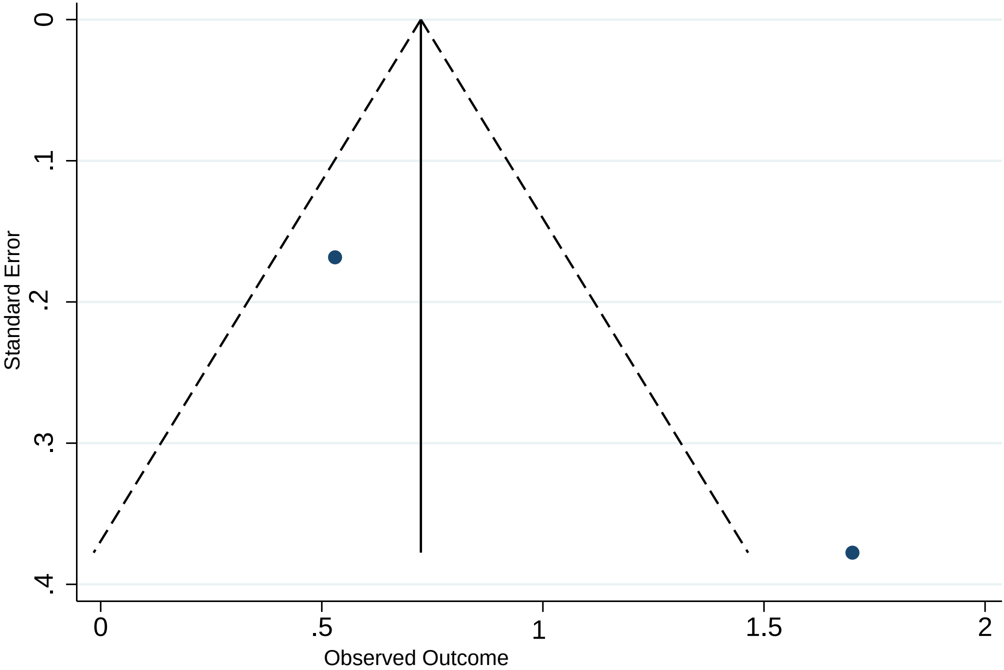

Figure S190. Funnel plot of meta-analysis of the association between serum NFH level and ALS

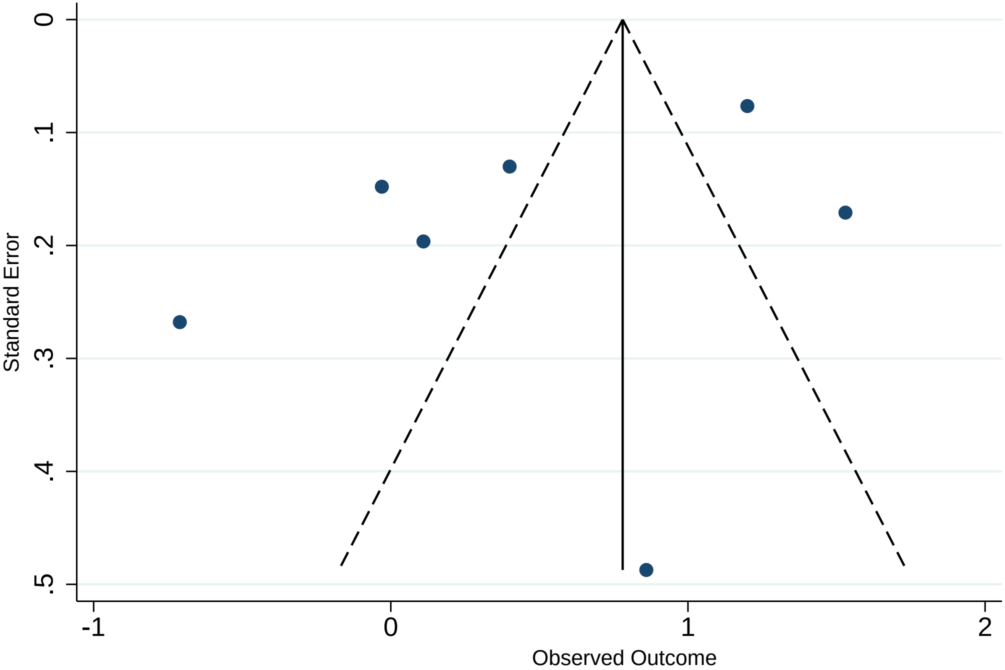

Figure S191. Funnel plot of meta-analysis of the association between serum iron level and ALS

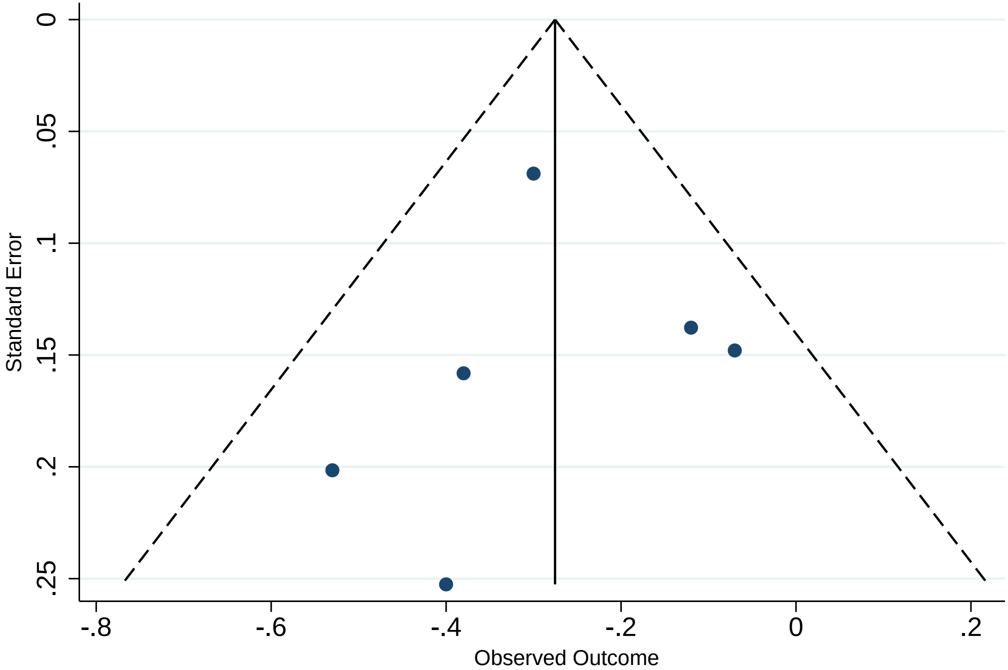

Figure S192. Funnel plot of meta-analysis of the association between serum transferrin level and ALS

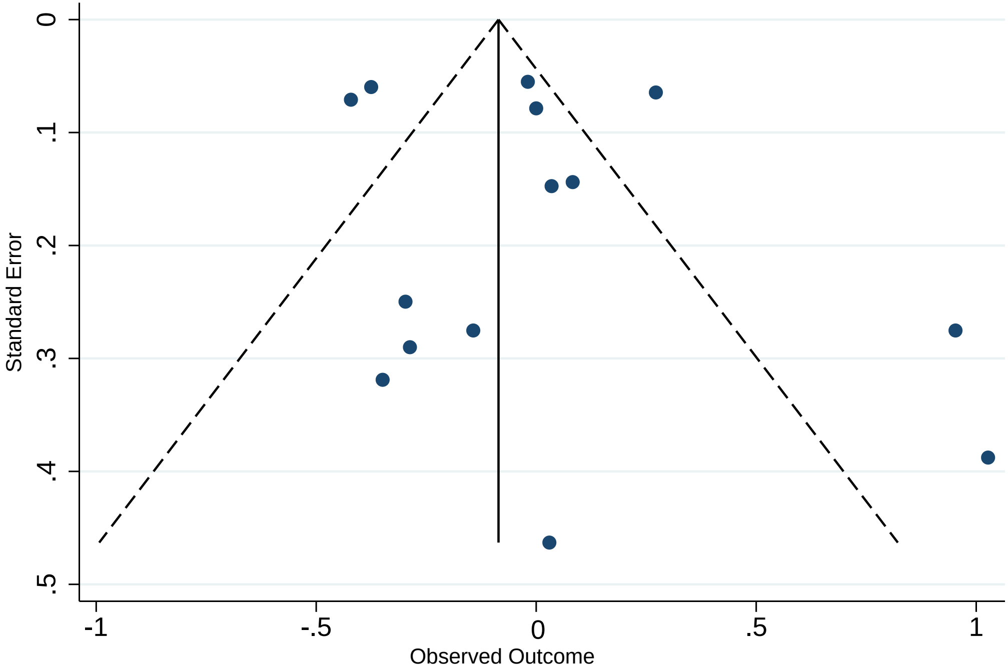

Figure S193. Funnel plot of meta-analysis of the association between serum HDL level and ALS

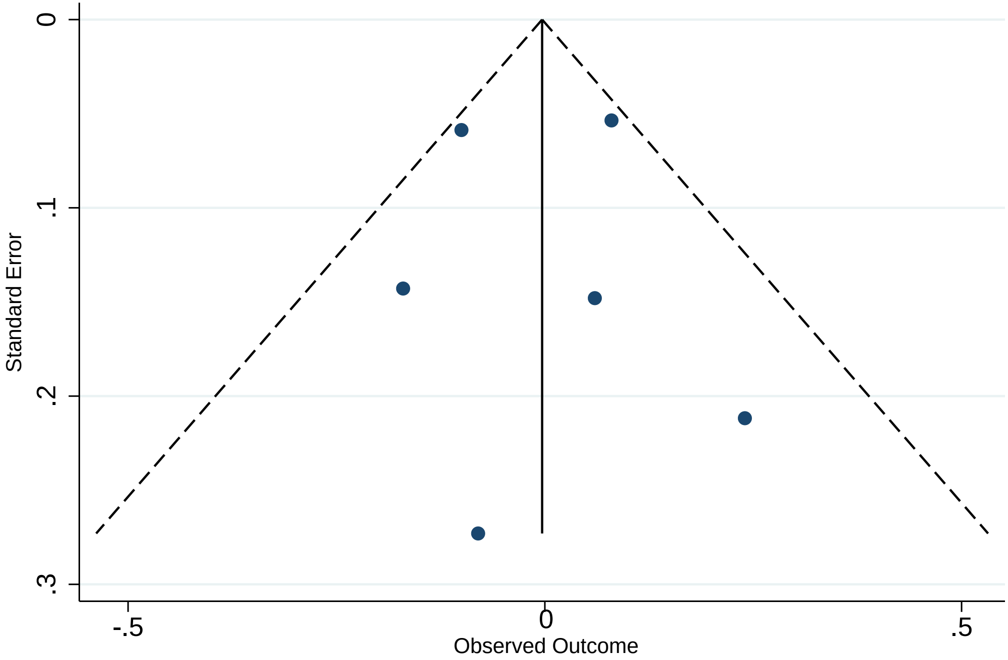

Figure S194. Funnel plot of meta-analysis of the association between serum LDL level and ALS

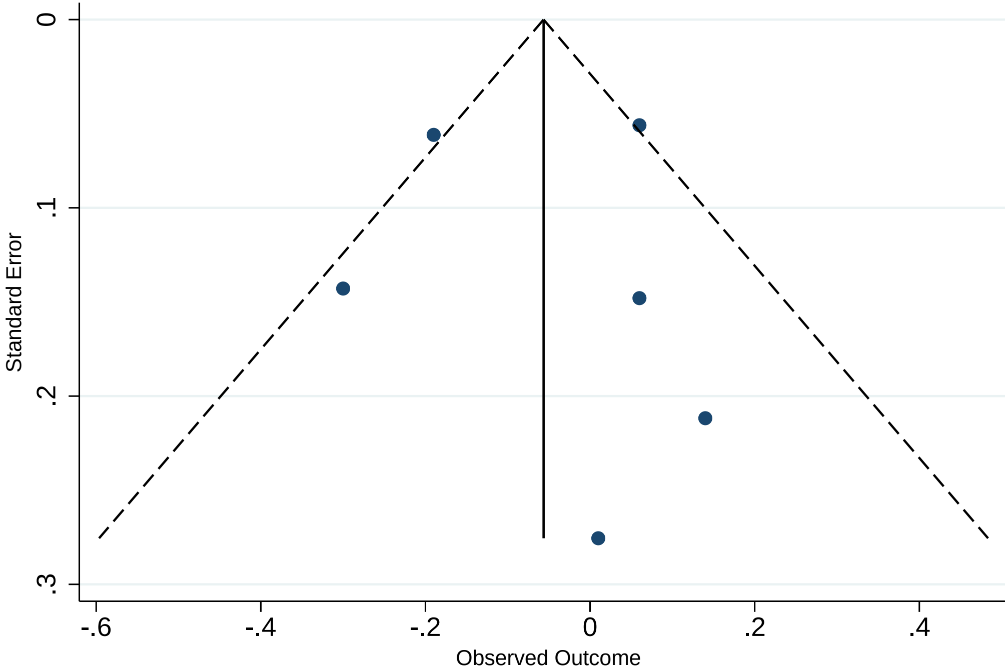

Figure S195. Funnel plot of meta-analysis of the association between serum TC level and ALS

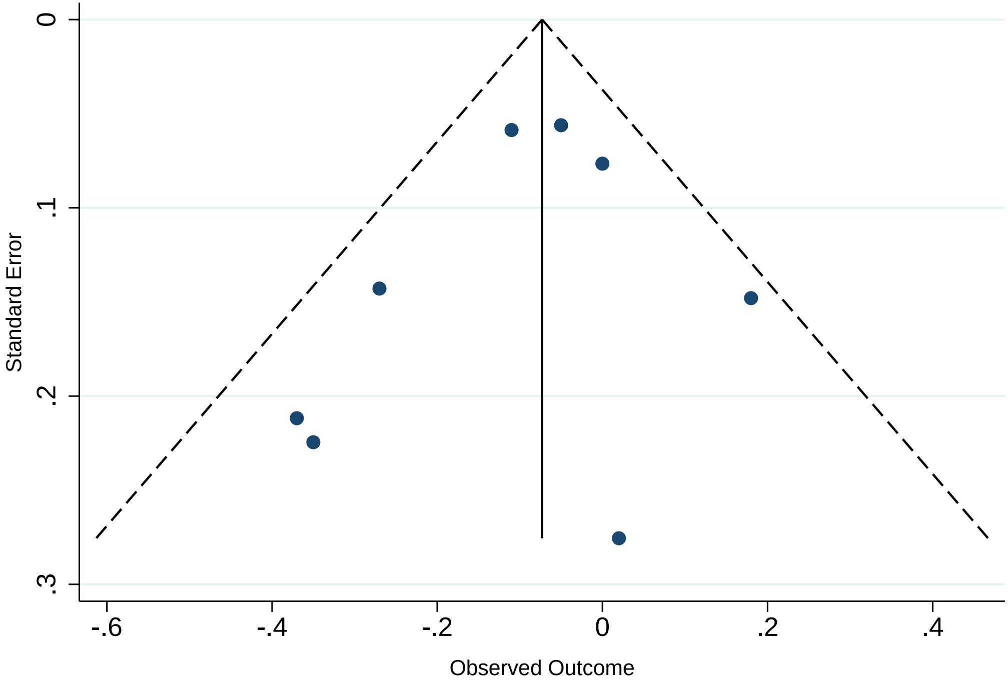

Figure S196. Funnel plot of meta-analysis of the association between serum TG level and ALS

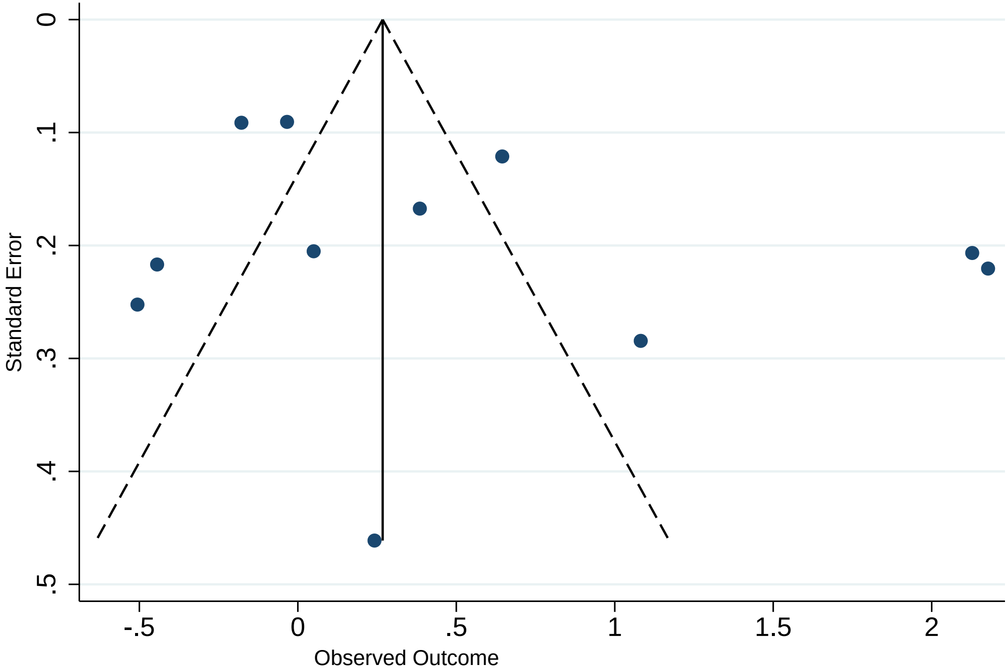

Figure S197. Funnel plot of meta-analysis of the association between serum homocysteine level and ALS

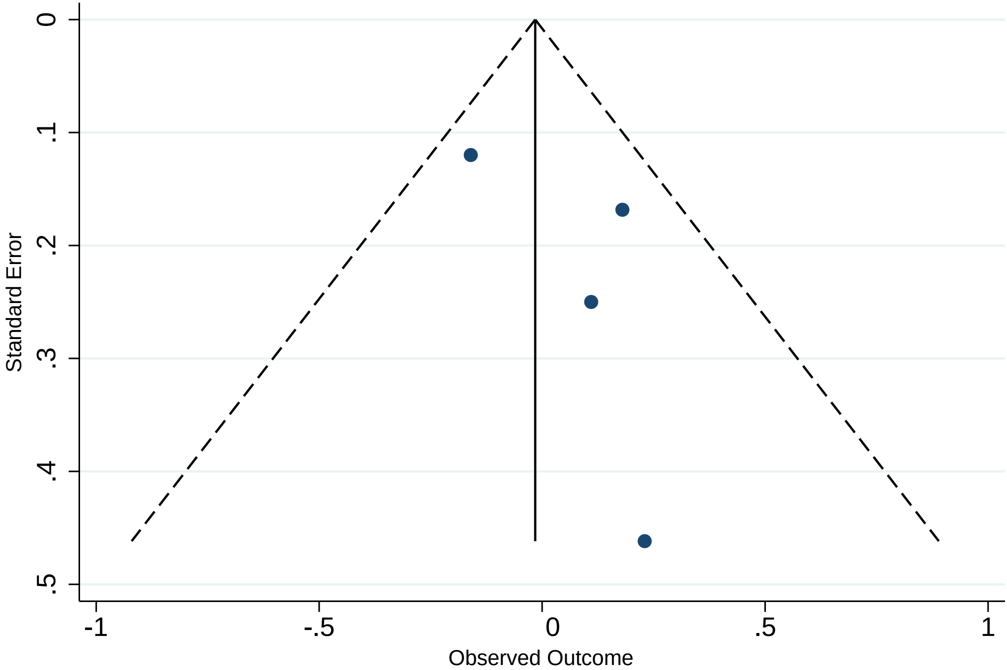

Figure S198. Funnel plot of meta-analysis of the association between serum vitamin B12 level and ALS

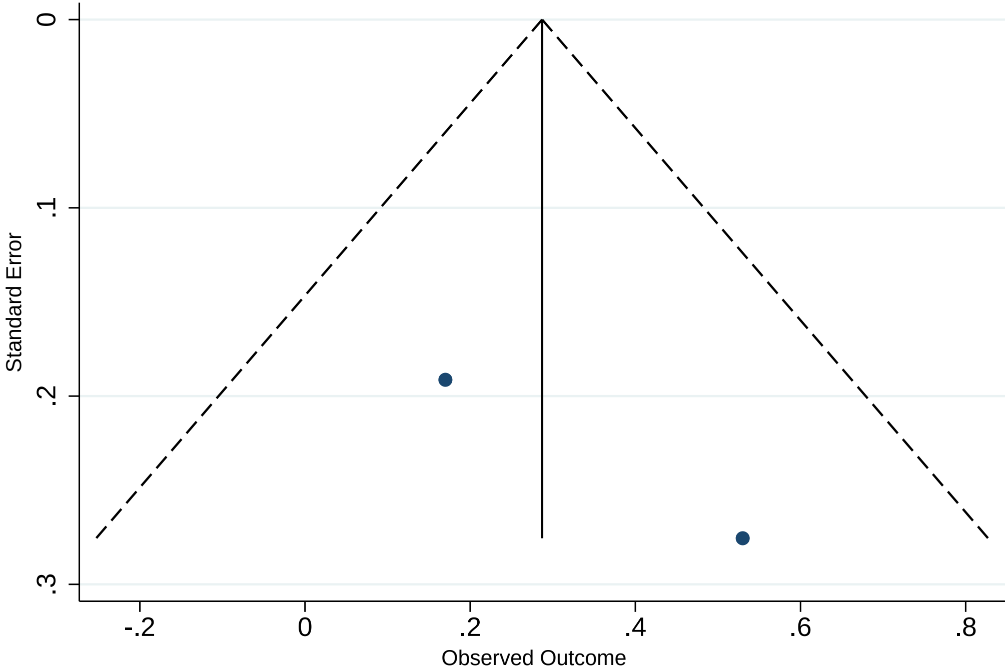

Figure S199. Funnel plot of meta-analysis of the association between serum galectin level and ALS

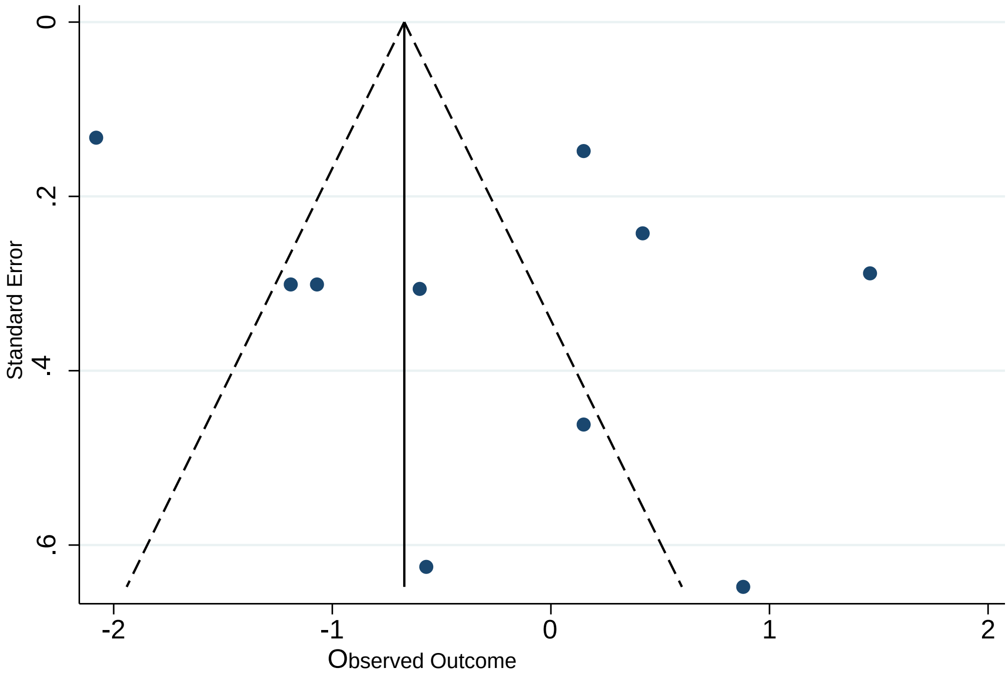

Figure S200. Funnel plot of meta-analysis of the association between serum selenium level and ALS

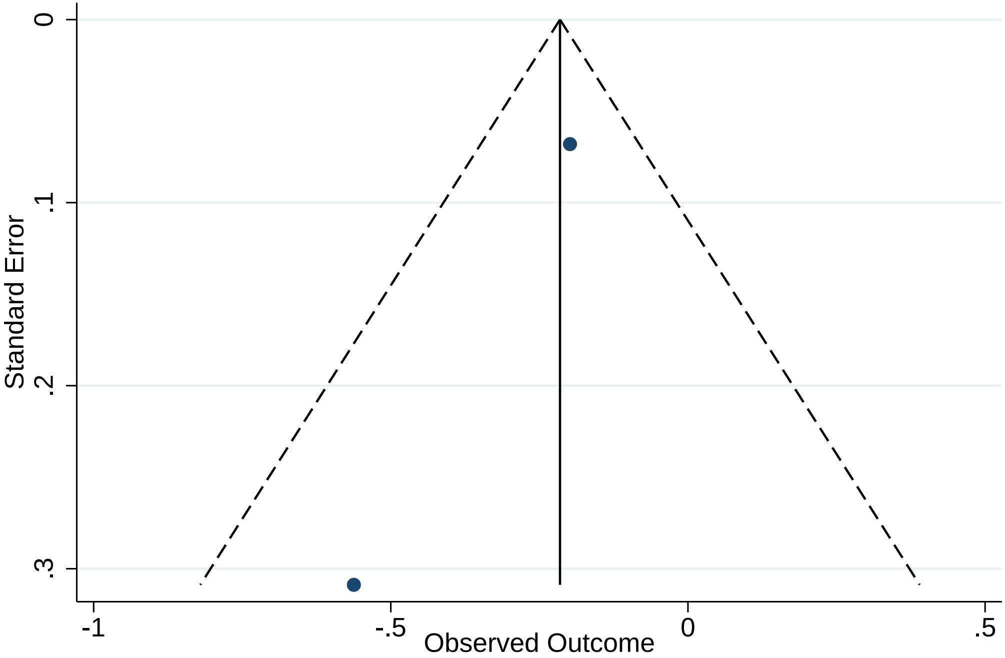

Figure S201. Funnel plot of meta-analysis of the association between serum ApoE1 level and ALS

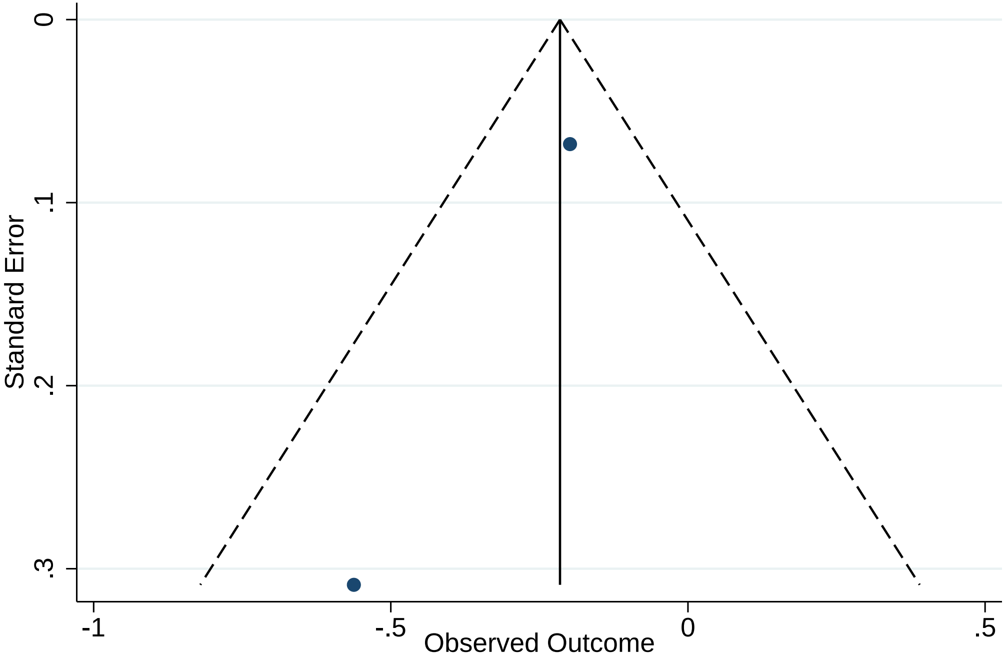

Figure S202. Funnel plot of meta-analysis of the association between serum ApoB level and ALS

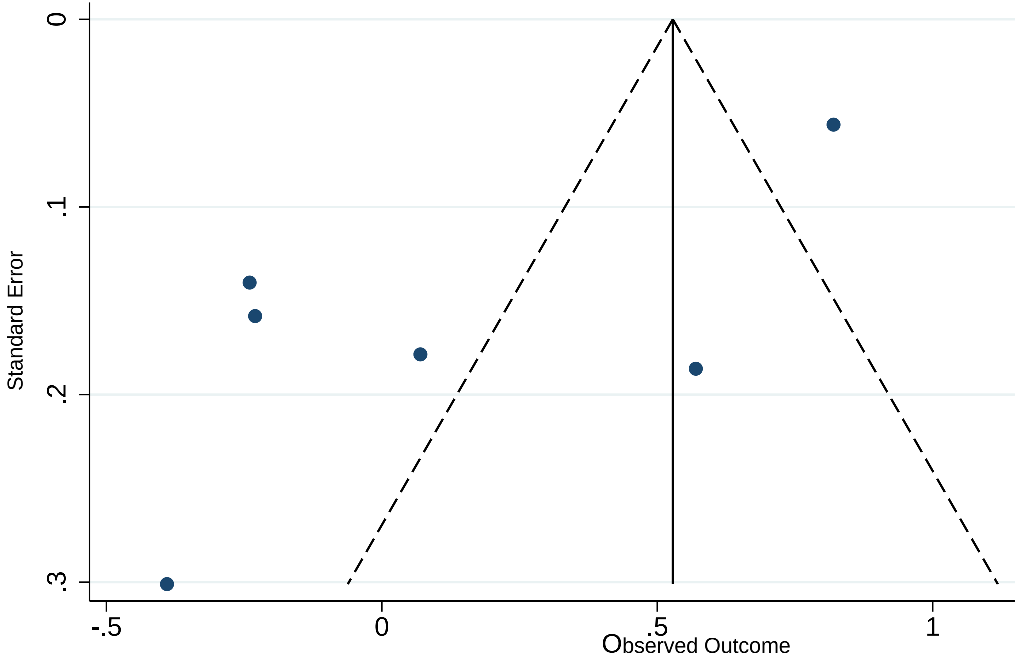

Figure S203. Funnel plot of meta-analysis of the association between CSF p-tau level and ALS

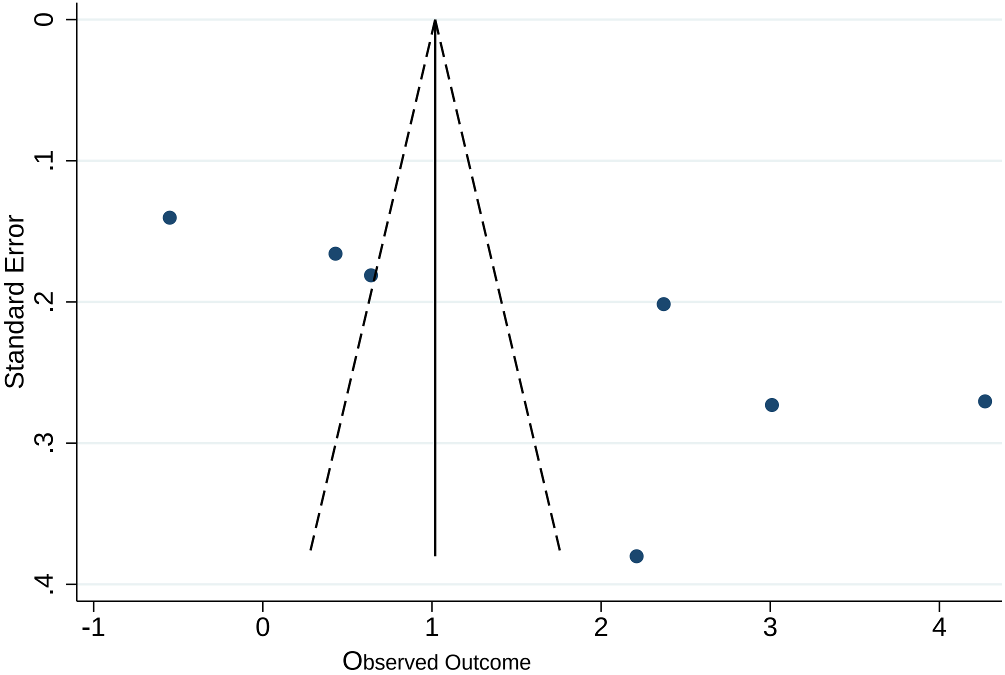

Figure S204. Funnel plot of meta-analysis of the association between CSF t-tau level and ALS

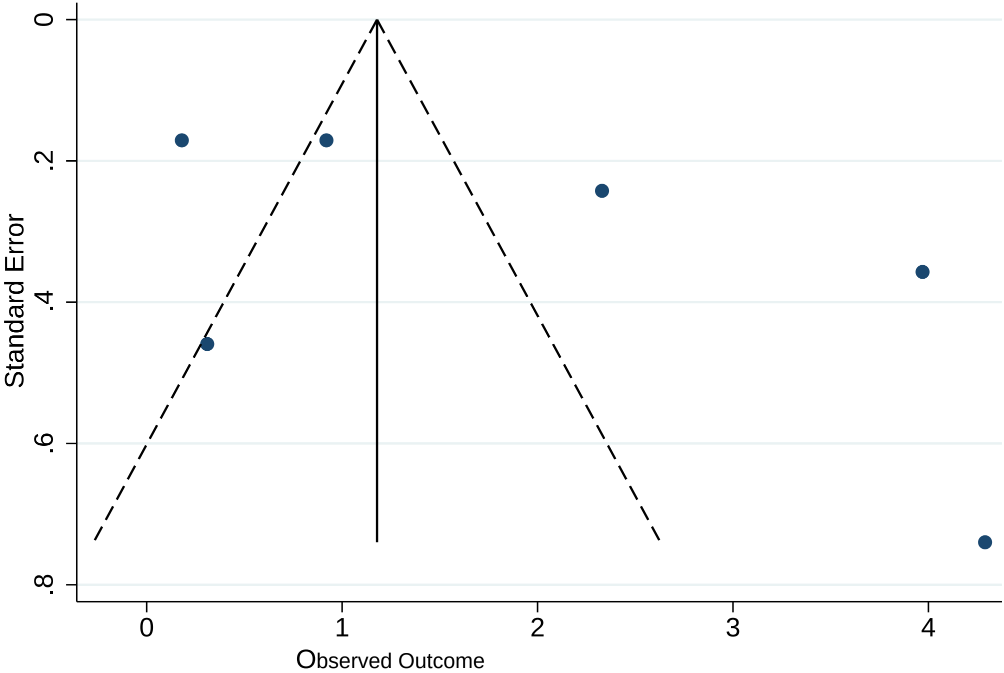

Figure S205. Funnel plot of meta-analysis of the association between CSF CHIT1 level and ALS

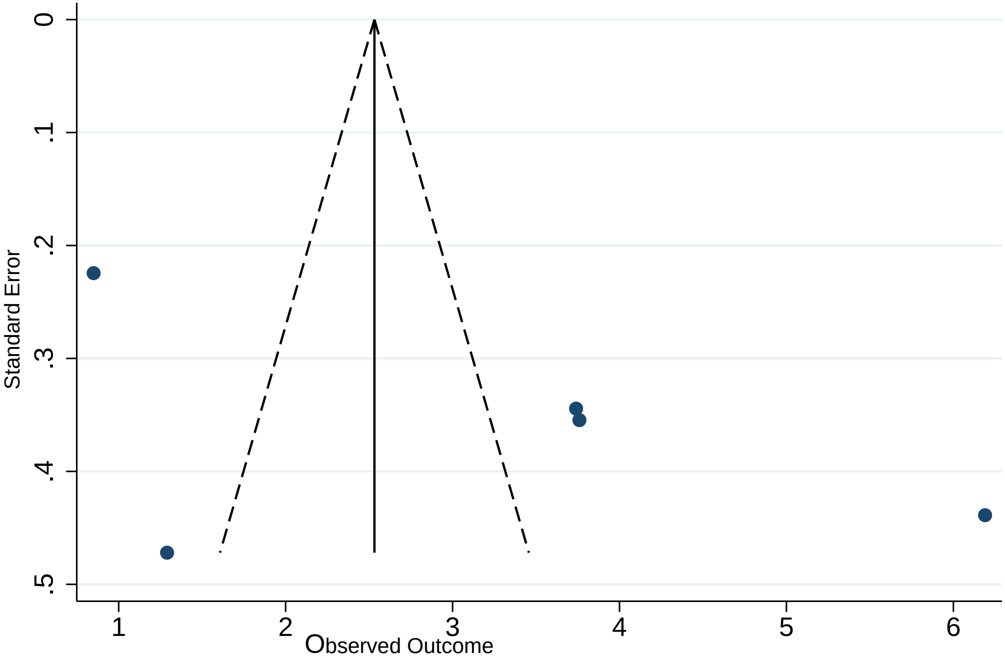

Figure S206. Funnel plot of meta-analysis of the association between CSF CHI3L1 level and ALS
