# Supplementary material for: Environmental risk factors, protective factors, and biomarkers for amyotrophic lateral sclerosis: an umbrella review
Source: Front Aging Neurosci. 2025 Jun 13;17:1541779. doi: 10.3389/fnagi.2025.1541779 (PMC12202415; doi:10.3389/fnagi.2025.1541779)
Supplement: Supplementary file 3 [file Table_1.docx]

| Evidence level | Convincing  (class I)* | Highly suggestive  (class II)* | Suggestive  (class III) | Weak  (class IV) | Not significant  (NS) |
| --- | --- | --- | --- | --- | --- |
| Random effects *p* value | <0·000001 | <0·000001 | <0·001 | <0·05 | >0·05 |
| Number of ALS cases | >1000 | >1000 | >1000 |  |  |
| *P* value of the largest study | <0.05 | <0·05 |  |  |  |
| Heterogeneity (*I²*) | <50% |  |  |  |  |
| Small study effects | Not detected |  |  |  |  |
| Excess significance bias | Not detected |  |  |  |  |
| 95% prediction interval | Excludes the null |  |  |  |  |

**Table S1. Level of evidence for grading levels.**
