# Supplementary material for: Environmental risk factors, protective factors, and biomarkers for amyotrophic lateral sclerosis: an umbrella review
Source: Front Aging Neurosci. 2025 Jun 13;17:1541779. doi: 10.3389/fnagi.2025.1541779 (PMC12202415; doi:10.3389/fnagi.2025.1541779)
Supplement: Supplementary file 4 [file Table_2.docx]

**Table S2. List of excluded full-text reference.**

| **Reasons for exclusion** | **Number** | **Reference** |
| --- | --- | --- |
| Not meta-analysis of observational studies | 38 | [1-38] |
| Not largest meta-analysis of same topic | 28 | [39-66] |
| Letters, conference abstracts, and reviews | 21 | [67-87] |
| Not focused on ALS risk | 12 | [88-99] |
| Not environmental factors or CSF/serum biomarkers | 8 | [100-107] |
| Non-English publication | 3 | [108-110] |

1. Abhinav, K., et al., *Electrical injury and amyotrophic lateral sclerosis: a systematic review of the literature.* J Neurol Neurosurg Psychiatry, 2007. **78**(5): p. 450-3.

2. Alqarni, S. and M. Alsebai, *Could VGF and/or its derived peptide act as biomarkers for the diagnosis of neurodegenerative diseases: A systematic review.* Front Endocrinol (Lausanne), 2022. **13**: p. 1032192.

3. Bellomo, G., et al., *A systematic review on the risk of neurodegenerative diseases and neurocognitive disorders in professional and varsity athletes.* Neurol Sci, 2022. **43**(12): p. 6667-6691.

4. Bianchi, V.E., P.F. Herrera, and R. Laura, *Effect of nutrition on neurodegenerative diseases. A systematic review.* Nutr Neurosci, 2021. **24**(10): p. 810-834.

5. Bonnechère, B., N. Amin, and C. van Duijn, *What Are the Key Gut Microbiota Involved in Neurological Diseases? A Systematic Review.* Int J Mol Sci, 2022. **23**(22).

6. Brito, M.D., et al., *Metabolic Alteration and Amyotrophic Lateral Sclerosis Outcome: A Systematic Review.* Front Neurol, 2019. **10**: p. 1205.

7. Carvalho, T.L., et al., *Depression and anxiety in individuals with amyotrophic lateral sclerosis a systematic review.* Trends Psychiatry Psychother, 2016. **38**(1): p. 1-5.

8. Cicero, C.E., et al., *Metals and neurodegenerative diseases. A systematic review.* Environ Res, 2017. **159**: p. 82-94.

9. Cui, C., et al., *Medication use and risk of amyotrophic lateral sclerosis-a systematic review.* BMC Med, 2022. **20**(1): p. 251.

10. Daneshafrooz, N., et al., *Identification of let-7f and miR-338 as plasma-based biomarkers for sporadic amyotrophic lateral sclerosis using meta-analysis and empirical validation.* Sci Rep, 2022. **12**(1): p. 1373.

11. Erber, A.C., et al., *The role of gut microbiota, butyrate and proton pump inhibitors in amyotrophic lateral sclerosis: a systematic review.* Int J Neurosci, 2020. **130**(7): p. 727-735.

12. Ferri, L., et al., *Diabetes Mellitus and Amyotrophic Lateral Sclerosis: A Systematic Review.* Biomolecules, 2021. **11**(6).

13. Gao, J., et al., *Creatine kinase and prognosis in amyotrophic lateral sclerosis: a literature review and multi-centre cohort analysis.* Journal of Neurology, 2022.

14. He, D. and L. Cui, *Assessing the Causal Role of Selenium in Amyotrophic Lateral Sclerosis: A Mendelian Randomization Study.* Front Genet, 2021. **12**: p. 724903.

15. Hersi, M., et al., *Systematic reviews of factors associated with the onset and progression of neurological conditions in humans: A methodological overview.* Neurotoxicology, 2017. **61**: p. 12-18.

16. Hui, B.S.M., et al., *The Role of Interferon-α in Neurodegenerative Diseases: A Systematic Review.* J Alzheimers Dis, 2023.

17. Kharel, S., et al., *C-reactive protein levels in patients with amyotrophic lateral sclerosis: A systematic review.* Brain Behav, 2022. **12**(3): p. e2532.

18. Lacorte, E., et al., *Physical activity, and physical activity related to sports, leisure and occupational activity as risk factors for ALS: A systematic review.* Neurosci Biobehav Rev, 2016. **66**: p. 61-79.

19. Lekoubou, A., et al., *Effects of diabetes mellitus on amyotrophic lateral sclerosis: a systematic review.* BMC Res Notes, 2014. **7**: p. 171.

20. Nakane, S., et al., *A Potential Link between Amyotrophic Lateral Sclerosis and Bullous Pemphigoid: Six New Cases and a Systematic Review of the Literature.* Intern Med, 2016. **55**(15): p. 1985-90.

21. Ou, Y.N., et al., *Causal effects of serum sex hormone binding protein levels on the risk of amyotrophic lateral sclerosis: a mendelian randomization study.* Ann Transl Med, 2022. **10**(19): p. 1054.

22. Pardo-Moreno, T., et al., *Amyotrophic Lateral Sclerosis and Serum Lipid Level Association: A Systematic Review and Meta-Analytic Study.* Int J Mol Sci, 2023. **24**(10).

23. Parton, M., H. Mitsumoto, and P.N. Leigh, *Amino acids for amyotrophic lateral sclerosis / motor neuron disease.* Cochrane Database Syst Rev, 2003(4): p. Cd003457.

24. Rawlings, L., et al., *Drug S-oxidation and phenylalanine hydroxylase: a biomarker for neurodegenerative susceptibility in Parkinson's disease and amyotrophic lateral sclerosis.* Drug Metab Pers Ther, 2019. **34**(2).

25. Saucier, D., et al., *Urbanization, air pollution, and water pollution: Identification of potential environmental risk factors associated with amyotrophic lateral sclerosis using systematic reviews.* Front Neurol, 2023. **14**: p. 1108383.

26. Su, C.L., et al., *Effects of pulmonary rehabilitation program on amyotrophic lateral sclerosis: A meta-analysis of randomized controlled trials.* NeuroRehabilitation, 2021. **48**(3): p. 255-265.

27. Sun, J., et al., *Gut microbiome and amyotrophic lateral sclerosis: A systematic review of current evidence.* J Intern Med, 2021. **290**(4): p. 758-788.

28. Sutedja, N.A., et al., *What we truly know about occupation as a risk factor for ALS: a critical and systematic review.* Amyotroph Lateral Scler, 2009. **10**(5-6): p. 295-301.

29. Sutedja, N.A., et al., *Exposure to chemicals and metals and risk of amyotrophic lateral sclerosis: a systematic review.* Amyotroph Lateral Scler, 2009. **10**(5-6): p. 302-9.

30. Torbick, N., et al., *Assessing Cyanobacterial Harmful Algal Blooms as Risk Factors for Amyotrophic Lateral Sclerosis.* Neurotox Res, 2018. **33**(1): p. 199-212.

31. van Schaik, I.N., et al., *Diagnostic value of GM1 antibodies in motor neuron disorders and neuropathies: a meta-analysis.* Neurology, 1995. **45**(8): p. 1570-7.

32. Vijayakumar, U.G., et al., *A Systematic Review of Suggested Molecular Strata, Biomarkers and Their Tissue Sources in ALS.* Front Neurol, 2019. **10**: p. 400.

33. Wang, H., et al., *Smoking and risk of amyotrophic lateral sclerosis: a pooled analysis of 5 prospective cohorts.* Arch Neurol, 2011. **68**(2): p. 207-13.

34. Wang, H., et al., *Vitamin e intake and risk of amyotrophic lateral sclerosis: A pooled analysis of data from 5 prospective cohort studies.* American Journal of Epidemiology, 2011. **173**(6): p. 595-602.

35. Wang, H., et al., *Systematic reviews and meta- and pooled analyses: Vitamin E intake and risk of amyotrophic lateral sclerosis: A pooled analysis of data from 5 prospective cohort studies.* American Journal of Epidemiology, 2011. **173**(6): p. 595-602.

36. Wu, P.F., et al., *Assessment of causal effects of physical activity on neurodegenerative diseases: A Mendelian randomization study.* J Sport Health Sci, 2021. **10**(4): p. 454-461.

37. Yap, K.H., et al., *Profiling neuroprotective potential of trehalose in animal models of neurodegenerative diseases: a systematic review.* Neural Regen Res, 2023. **18**(6): p. 1179-1185.

38. Zhang, H. and Z. Zhou, *COVID-19 and the risk of Alzheimer's disease, amyotrophic lateral sclerosis, and multiple sclerosis.* Ann Clin Transl Neurol, 2022. **9**(12): p. 1953-1961.

39. Abraham, A. and V.E. Drory, *Influence of serum uric acid levels on prognosis and survival in amyotrophic lateral sclerosis: a meta-analysis.* J Neurol, 2014. **261**(6): p. 1133-8.

40. Alonso, A., G. Logroscino, and M.A. Hernán, *Smoking and the risk of amyotrophic lateral sclerosis: a systematic review and meta-analysis.* J Neurol Neurosurg Psychiatry, 2010. **81**(11): p. 1249-52.

41. Bridel, C., et al., *Diagnostic Value of Cerebrospinal Fluid Neurofilament Light Protein in Neurology: A Systematic Review and Meta-analysis.* JAMA Neurol, 2019. **76**(9): p. 1035-1048.

42. Chang, M.C., et al., *Relationship between statins and the risk of amyotrophic lateral sclerosis: A PRISMA-compliant meta-analysis.* Medicine (Baltimore), 2021. **100**(30): p. e26751.

43. Chen, H., et al., *Head injury and amyotrophic lateral sclerosis.* Am J Epidemiol, 2007. **166**(7): p. 810-6.

44. E, M., et al., *Association between alcohol consumption and amyotrophic lateral sclerosis: a meta-analysis of five observational studies.* Neurol Sci, 2016. **37**(8): p. 1203-8.

45. Filippini, T., E.E. Hatch, and M. Vinceti, *Residential exposure to electromagnetic fields and risk of amyotrophic lateral sclerosis: a dose-response meta-analysis.* Sci Rep, 2021. **11**(1): p. 11939.

46. Forgrave, L.M., et al., *The diagnostic performance of neurofilament light chain in CSF and blood for Alzheimer's disease, frontotemporal dementia, and amyotrophic lateral sclerosis: A systematic review and meta-analysis.* Alzheimers Dement (Amst), 2019. **11**: p. 730-743.

47. Gunnarsson, L.G. and L. Bodin, *Occupational Exposures and Neurodegenerative Diseases-A Systematic Literature Review and Meta-Analyses.* Int J Environ Res Public Health, 2019. **16**(3).

48. Hu, X., et al., *Meta-analysis of the relationship between amyotrophic lateral sclerosis and susceptibility to serum ferritin level elevation.* Neurosciences (Riyadh), 2016. **21**(2): p. 120-5.

49. Huang, R., et al., *The serum lipid profiles of amyotrophic lateral sclerosis patients: A study from south-west China and a meta-analysis.* Amyotroph Lateral Scler Frontotemporal Degener, 2015. **16**(5-6): p. 359-65.

50. Huss, A., S. Peters, and R. Vermeulen, *Occupational exposure to extremely low-frequency magnetic fields and the risk of ALS: A systematic review and meta-analysis.* Bioelectromagnetics, 2018. **39**(2): p. 156-163.

51. Kamalian, A., et al., *Metal concentrations in cerebrospinal fluid, blood, serum, plasma, hair, and nails in amyotrophic lateral sclerosis: A systematic review and meta-analysis.* J Trace Elem Med Biol, 2023. **78**: p. 127165.

52. Kamel, F., et al., *Pesticide exposure and amyotrophic lateral sclerosis.* Neurotoxicology, 2012. **33**(3): p. 457-62.

53. Li, D., et al., *Neurofilaments in CSF As Diagnostic Biomarkers in Motor Neuron Disease: A Meta-Analysis.* Front Aging Neurosci, 2016. **8**: p. 290.

54. Liu, G., et al., *Head Injury and Amyotrophic Lateral Sclerosis: A Meta-Analysis.* Neuroepidemiology, 2021: p. 1-9.

55. Majumder, V., et al., *TDP-43 as a potential biomarker for amyotrophic lateral sclerosis: a systematic review and meta-analysis.* BMC Neurol, 2018. **18**(1): p. 90.

56. Malek, A.M., et al., *Pesticide exposure as a risk factor for amyotrophic lateral sclerosis: a meta-analysis of epidemiological studies: pesticide exposure as a risk factor for ALS.* Environ Res, 2012. **117**: p. 112-9.

57. Nabizadeh, F., et al., *Statins and risk of amyotrophic lateral sclerosis: a systematic review and meta-analysis.* Acta Neurol Belg, 2022. **122**(4): p. 979-986.

58. Perry, D.C., et al., *Association of traumatic brain injury with subsequent neurological and psychiatric disease: a meta-analysis.* J Neurosurg, 2016. **124**(2): p. 511-26.

59. Röösli, M. and H. Jalilian, *A meta-analysis on residential exposure to magnetic fields and the risk of amyotrophic lateral sclerosis.* Rev Environ Health, 2018. **33**(3): p. 309-313.

60. Sako, W. and S. Ishimoto, *Can cystatin C in cerebrospinal fluid be a biomarker for amyotrophic lateral sclerosis? A lesson from previous studies.* Neurology and Clinical Neuroscience, 2014. **2**(3): p. 72-75.

61. Vergara, X., et al., *Occupational exposure to extremely low-frequency magnetic fields and neurodegenerative disease: a meta-analysis.* J Occup Environ Med, 2013. **55**(2): p. 135-46.

62. Wang, M.D., et al., *Identification of risk factors associated with onset and progression of amyotrophic lateral sclerosis using systematic review and meta-analysis.* Neurotoxicology, 2017. **61**: p. 101-130.

63. Wang, S.Y., et al., *Neurofilament Light Chain in Cerebrospinal Fluid and Blood as a Biomarker for Neurodegenerative Diseases: A Systematic Review and Meta-Analysis.* J Alzheimers Dis, 2019. **72**(4): p. 1353-1361.

64. Zhang, L., et al., *Association between type 2 diabetes and amyotrophic lateral sclerosis.* Sci Rep, 2022. **12**(1): p. 2544.

65. Zheng, Z., L. Sheng, and H. Shang, *Statins and amyotrophic lateral sclerosis: a systematic review and meta-analysis.* Amyotroph Lateral Scler Frontotemporal Degener, 2013. **14**(4): p. 241-5.

66. Zhou, H., et al., *Association between extremely low-frequency electromagnetic fields occupations and amyotrophic lateral sclerosis: a meta-analysis.* PLoS One, 2012. **7**(11): p. e48354.

67. Agah, E., et al., *CSF and blood biomarkers in amyotrophic lateral sclerosis: protocol for a systematic review and meta-analysis.* Syst Rev, 2018. **7**(1): p. 237.

68. Bozzoni, V., et al., *Amyotrophic lateral sclerosis and environmental factors.* Functional Neurology, 2016. **31**(1): p. 7-19.

69. Catalá-López, F., et al., *Cancer and central nervous system disorders: protocol for an umbrella review of systematic reviews and updated meta-analyses of observational studies.* Syst Rev, 2017. **6**(1): p. 69.

70. Farace, C., et al., *Amyotrophic lateral sclerosis and lead: A systematic update.* Neurotoxicology, 2020. **81**: p. 80-88.

71. Fu, Y.W., H.S. Xu, and S. Liu, *COVID-19 and neurodegenerative diseases.* European Review for Medical and Pharmacological Sciences, 2022. **26**(12): p. 4535-4544.

72. Gagliardi, D., et al., *Diagnostic and Prognostic Role of Blood and Cerebrospinal Fluid and Blood Neurofilaments in Amyotrophic Lateral Sclerosis: A Review of the Literature.* Int J Mol Sci, 2019. **20**(17).

73. Gómez-Anca, S. and J.M. Barros-Dios, *Radon Exposure and Neurodegenerative Disease.* Int J Environ Res Public Health, 2020. **17**(20).

74. Goncharova, P.S., et al., *Nutrient Effects on Motor Neurons and the Risk of Amyotrophic Lateral Sclerosis.* Nutrients, 2021. **13**(11).

75. Hamidou, B., et al., *Epidemiological evidence that physical activity is not a risk factor for ALS.* Eur J Epidemiol, 2014. **29**(7): p. 459-75.

76. Heckler, I. and I. Venkataraman, *Phosphorylated neurofilament heavy chain: a potential diagnostic biomarker in amyotrophic lateral sclerosis.* J Neurophysiol, 2022. **127**(3): p. 737-745.

77. Lotz, S.K., et al., *Microbial Infections Are a Risk Factor for Neurodegenerative Diseases.* Frontiers in Cellular Neuroscience, 2021. **15**.

78. Luna, J., et al., *Current issues in ALS epidemiology: Variation of ALS occurrence between populations and physical activity as a risk factor.* Revue Neurologique, 2017. **173**(5): p. 244-253.

79. McKay, K.A., et al., *Military service and related risk factors for amyotrophic lateral sclerosis.* Acta Neurol Scand, 2021. **143**(1): p. 39-50.

80. Mitchell, J.D., *Amyotrophic lateral sclerosis: toxins and environment.* Amyotroph Lateral Scler Other Motor Neuron Disord, 2000. **1**(4): p. 235-50.

81. Ngo, S.T., F.J. Steyn, and P.A. McCombe, *Body mass index and dietary intervention: Implications for prognosis of amyotrophic lateral sclerosis.* Journal of the Neurological Sciences, 2014. **340**(1-2): p. 5-12.

82. Petzold, A., *The 2022 Lady Estelle Wolfson lectureship on neurofilaments.* J Neurochem, 2022. **163**(3): p. 179-219.

83. Roda, M., et al., *Biomarkers in Tears and Ocular Surface: A Window for Neurodegenerative Diseases.* Eye Contact Lens, 2020. **46 Suppl 2**: p. S129-s134.

84. Sataer, X., et al., *Exosomal microRNAs as diagnostic biomarkers and therapeutic applications in neurodegenerative diseases.* Neurological Research, 2023. **45**(3): p. 191-199.

85. Stefanis, L., J.B.C. de Andrade, and J.P. Mohr, *Brain Arteriovenous Malformation and Amyotrophic Lateral Sclerosis: a Review Based on Published Cases.* SN Comprehensive Clinical Medicine, 2020. **2**(4): p. 392-396.

86. Swash, M. and M. de Carvalho, *Risk factors for onset of amyotrophic lateral sclerosis.* European Journal of Neurology, 2017. **24**(1): p. 9-10.

87. Youn, B.Y., et al., *Digital biomarkers for neuromuscular disorders: A systematic scoping review.* Diagnostics, 2021. **11**(7).

88. Dardiotis, E., et al., *Body mass index and survival from amyotrophic lateral sclerosis: A meta-analysis.* Neurol Clin Pract, 2018. **8**(5): p. 437-444.

89. Haji, S., et al., *The value of serum uric acid as a prognostic biomarker in amyotrophic lateral sclerosis: Evidence from a meta-analysis.* Clin Neurol Neurosurg, 2021. **203**: p. 106566.

90. Janse van Mantgem, M.R., et al., *Association Between Serum Lipids and Survival in Patients With Amyotrophic Lateral Sclerosis: A Meta-analysis and Population-Based Study.* Neurology, 2023. **100**(10): p. e1062-e1071.

91. Kutlubaev, M.A., et al., *Apathy in amyotrophic lateral sclerosis: systematic review and meta-analysis of frequency, correlates, and outcomes.* Amyotroph Lateral Scler Frontotemporal Degener, 2022: p. 1-10.

92. Lanznaster, D., et al., *Plasma creatinine and amyotrophic lateral sclerosis prognosis: a systematic review and meta-analysis.* Amyotroph Lateral Scler Frontotemporal Degener, 2019. **20**(3-4): p. 199-206.

93. Larsson, S.C. and S. Burgess, *Causal role of high body mass index in multiple chronic diseases: a systematic review and meta-analysis of Mendelian randomization studies.* BMC Med, 2021. **19**(1): p. 320.

94. Li, N., et al., *Green leafy vegetable and lutein intake and multiple health outcomes.* Food Chem, 2021. **360**: p. 130145.

95. Lugg, A., et al., *Nerve excitability as a biomarker for amyotrophic lateral sclerosis: a systematic review and meta-analysis*. 2022.

96. Morales, J.S., et al., *Mortality Risk from Neurodegenerative Disease in Sports Associated with Repetitive Head Impacts: Preliminary Findings from a Systematic Review and Meta-Analysis.* Sports Med, 2022. **52**(4): p. 835-846.

97. Song, Y., et al., *Herbal medicine for amyotrophic lateral sclerosis: A systematic review and meta-analysis.* Front Pharmacol, 2022. **13**: p. 946548.

98. Zhou, B., et al., *Different observation period of exercise training in amyotrophic lateral sclerosis patients: A meta-analysis.* Front Neurol, 2022. **13**: p. 986882.

99. Zhou, Y.N., et al., *Role of Blood Neurofilaments in the Prognosis of Amyotrophic Lateral Sclerosis: A Meta-Analysis.* Front Neurol, 2021. **12**: p. 712245.

100. Cao, M.C. and E.L. Scotter, *Novel and known transcriptional targets of ALS/FTD protein TDP-43: Meta-analysis and interactive graphical databases*. 2021.

101. Danborg, P.B., et al., *The potential of microRNAs as biofluid markers of neurodegenerative diseases--a systematic review.* Biomarkers, 2014. **19**(4): p. 259-68.

102. Das, T., et al., *Intersection of network medicine and machine learning towards investigating the key biomarkers and pathways underlying amyotrophic lateral sclerosis: a systematic review.* Brief Bioinform, 2022. **23**(6).

103. Hu, N., J. Wang, and M. Liu, *Split hand in amyotrophic lateral sclerosis: A systematic review and meta-analysis.* J Clin Neurosci, 2021. **90**: p. 293-301.

104. Lu, W.Z., et al., *Split-hand index for amyotrophic lateral sclerosis diagnosis: A frequentist and Bayesian meta-analysis.* Clin Neurophysiol, 2022. **143**: p. 56-66.

105. Nona, R.J., et al., *HLA and amyotrophic lateral sclerosis: a systematic review and meta-analysis.* Amyotroph Lateral Scler Frontotemporal Degener, 2022: p. 1-9.

106. Rai, T., et al., *CSF TAR DNA Binding Protein -43 (TDP-43) As A Potential Biomarker in Diagnosing Amyotrophic Lateral Sclerosis (ALS): A Meta-Analysis.* Journal of Pharmaceutical Negative Results, 2022. **13**: p. 1226-1236.

107. Shi, G., et al., *Urinary p75(ECD) levels in patients with amyotrophic lateral sclerosis: a meta-analysis.* Amyotroph Lateral Scler Frontotemporal Degener, 2022. **23**(5-6): p. 438-445.

108. Capozzella, A., et al., *Work related etiology of amyotrophic lateral sclerosis (ALS): a meta-analysis.* Ann Ig, 2014. **26**(5): p. 456-72.

109. Nicolle-Mir, L., *Occupational exposure to extremely low-frequency magnetic fields and neurodegenerative diseases.* Environnement, Risques et Sante, 2014. **13**(1): p. 7-8.

110. Shen, D., et al., *Rural environment, pesticide exposure and the risk of amyotrophic lateral sclerosis: a meta analysis.* Chinese Journal of Neurology, 2016. **49**(1): p. 54-63.
