# Supplementary material for: Environmental risk factors, protective factors, and biomarkers for amyotrophic lateral sclerosis: an umbrella review
Source: Front Aging Neurosci. 2025 Jun 13;17:1541779. doi: 10.3389/fnagi.2025.1541779 (PMC12202415; doi:10.3389/fnagi.2025.1541779)
Supplement: Supplementary file 5 [file Table_3.docx]

**Table S3. Characteristics of included meta-analyses evaluating associations between environmental factors and ALS risk.**

| **Environmental factors** | **Author, year** | **Comparison** | **Article retrieval time** | **Assessment tool of original study** | **Study designs** |
| --- | --- | --- | --- | --- | --- |
|  |  |  |  |  |  |
| **Class I** |  |  |  |  |  |
| Anti-hypertensives | Hu N, 2022 | Exposed vs. not exposed | 21-Dec | AHRQ,NOS | cohort, case-control |
| **Class II** |  |  |  |  |  |
| Trauma | Gu D, 2021 | Exposed vs. not exposed | 20-Apr | NA | cohort, case-control |
| Premorbid body mass index | Zeng P, 2019 | High values vs. low values | NA | NA | cohort, case-control |
| **Class III** |  |  |  |  |  |
| Farming occupation | Kang H, 2014 | Exposed vs. not exposed | 13-Sep | NA | case-control |
| Pesticides | Kang H, 2014 | Exposed vs. not exposed | 13-Sep | NA | cohort, case-control |
| Head injury | Watanabe Y, 2017 | Exposed vs. not exposed | 16-Dec | NA | cohort, case-control |
| Leisure time activity | Zheng X,2023 | Exposed vs. not exposed | 22-May | NA | cohort, case-control |
| Anti-diabetes | Duan,Q,2023 | Exposed vs. not exposed | 21-Sep | NOS | case-control |
| Diabetes mellitus | Wannarong T, 2020 | Exposed vs. not exposed | 20-Jan | NOS | cohort, case-control |
| Kidney diseases | Duan,Q,2023 | Exposed vs. not exposed | 21-Sep | NOS | cohort, case-control |
| **Class IV** |  |  |  |  |  |
| Heavy metals | Duan,Q,2023 | Exposed vs. not exposed | 21-Sep | NOS | cohort, case-control |
| Metals | Wang M, 2014 | Exposed vs. not exposed | 13-Sep | a system | case-control |
| Lead | Meng E, 2020 | Exposed vs. not exposed | 19-Apr | NOS | cohort, nested case-control, case-control |
| Annual PM2.5 exposure | Gong Y, 2023 | Exposed vs. not exposed | 22-Oct | NOS | cohort, case-control |
| Competitive organized sports | Blecher R, 2019 | Exposed vs. not exposed | 17-Nov | NA | cohort, case-control |
| Vigorous physical activity | Zheng X,2023 | Exposed vs. not exposed | 22-May | NA | cohort, case-control |
| Occupational-related activity | Zheng X,2023 | Exposed vs. not exposed | 22-May | NA | cohort, case-control |
| Unclassified physical activity | Zheng X,2023 | Exposed vs. not exposed | 22-May | NA | case-control |
| Military personnel | Tai H, 2017 | Exposed vs. not exposed | 16-May | NOS | cohort, case-control |
| Heavy physical work | Gunnarsson L,2018 | Exposed vs. not exposed | 17-Feb | NA | cohort, case-control |
| Chemicals | Gunnarsson L, 2018 | Exposed vs. not exposed | 17-Feb | NA | cohort, case-control |
| Environmental andOccupational solvents | Zhang G,2023 | Exposed vs. not exposed | 22-Dec | NOS | cohort, case-control |
| ELF-MF | Jalilian H, 2021 | Exposed vs. not exposed | 2019 | NA | cohort, case-control |
| Stroke | Duan,Q,2023 | Exposed vs. not exposed | 21-Sep | NOS | case-control |
| ω-3 Polyunsaturated fatty acid intake | Fitzgerald K, 2014 | Intake_1 g/day increase | NA | NA | cohort |
| Carotenoids | Fitzgerald K, 2013 | 2,500 μg/day increase | NA | NA | cohort |
| Acetaminophen | Chang M, 2020 | Exposed vs. not exposed | 20-Feb | NOS | case-control |
| Living in urban | Duan,Q,2023 | Exposed vs. not exposed | 21-Sep | NOS | cohort, case-control |
| Smoking | Kim K, 2024 | NA | 23-Jan | NOS | cohort, case-control |
| **NS** |  |  |  |  |  |
| Occupation in industry | Zhu,Q,2023 | Exposed vs. not exposed | 22-Jun | NOS | case-control |
| Annual PM10 exposure | Gong Y, 2023 | Exposed vs. not exposed | 22-Oct | NOS | case-control |
| Rural residence | Kang H, 2014 | Exposed vs. not exposed | 13-Sep | NA | case-control |
| Work with electricity | Gunnarsson L,  2018 | Exposed vs. not exposed | 17-Feb | NA | cohort, case-control |
| Alcohol consumption | Duan,Q,2023 | Ever vs never | 21-Sep | NOS | case-control |
| hypertension | Duan,Q,2023 | Exposed vs. not exposed | 21-Sep | NOS | cohort, case-control |
| NSAIDs | Duan,Q,2023 | Exposed vs. not exposed | 21-Sep | NOS | case-control |
| Welding | Gunnarsson L, 2018 | Exposed vs. not exposed | 17-Feb | NA | cohort, case-control |
| Electric shocks | Jalilian H, 2021 | Exposed vs. not exposed | 2019 | NA | cohort, case-control |
| Statins | Hu N, 2022 | Exposed vs. not exposed | 21-Dec | AHRQ,NOS | cohort, case-control |
| Aspirin | Chang M, 2020 | Exposed vs. not exposed | 20-Feb | NOS | case-control |
| High vitamin diet | Duan,Q,2023 | Exposed vs. not exposed | 21-Sep | NOS | case-control |
| Sport-related activity | Zheng X,2023 | Exposed vs. not exposed | 22-May | NA | cohort, case-control |
| AMI/IS | Duan,Q,2023 | Exposed vs. not exposed | 21-Sep | NOS | case-control |
| Cerebrovascular disease. | Zhu,Q,2023 | Exposed vs. not exposed | 22-Jun | NOS | case-control |
| Occupation in service industry. | Zhu,Q,2023 | Exposed vs. not exposed | 22-Jun | NOS | case-control |
| Coffee drinking | Duan,Q,2023 | Ever vs never | 21-Sep | NOS | case-control |

NA: not available; ELF-MF: exposure to extremely-low frequency magnetic fields; NSAIDs: nonsteroidal anti-infammatory drugs; AMI/IS: acute myocardial infarction/ischemic stroke; NOS: Newcastle-Ottawa Scale; AHRQ: Agency for Healthcare Research and Quality;
