# Supplementary material for: Environmental risk factors, protective factors, and biomarkers for amyotrophic lateral sclerosis: an umbrella review
Source: Front Aging Neurosci. 2025 Jun 13;17:1541779. doi: 10.3389/fnagi.2025.1541779 (PMC12202415; doi:10.3389/fnagi.2025.1541779)
Supplement: Supplementary file 6 [file Table_4.docx]

**Table S4. Characteristics of included meta-analyses evaluating associations between biomarkers and ALS risk.**

| **Biomarkers** | **Author, year** | **Comparison** | **Article retrieval time** | **Assessment tool of original study** | **Study designs** |
| --- | --- | --- | --- | --- | --- |
|  |  |  |  |  |  |
| **Class II** |  |  |  |  |  |
| CSF NFL | Sferruzza G, 2022 | High values vs. low values | 21-Mar | QUADAS-2 | case-control, cross-sectional |
| Serum NFL | Sferruzza G, 2022 | High values vs. low values | 21-Mar | QUADAS-2 | case-control, cross-sectional |
| Serum feritin | Cheng Y, 2021 | High values vs. low values | 20-Jan | NA | case-control, cross-sectional |
| Serum uric acid | Zhang F, 2018 | High values vs. low values | 17-Aug | NOS | case-control |
| **Class III** |  |  |  |  |  |
| Serum transferrin | Wang L, 2020 | High values vs. low values | 19-Mar | NOS | case-control, cross-sectional |
| CSF CHIT1 | Xu A, 2024 | High values vs. low values | 23-Apr |  | case-control |
| **Class IV** |  |  |  |  |  |
| CSF cystatin C | Zhu Y, 2018 | High values vs. low values | 17-May | NOS | case-control |
| CSF TNF-α | Chen X, 2018 | High values vs. low values | 18-Mar | NA | case-control |
| CSF MIP-1α | Chen X, 2018 | High values vs. low values | 18-Mar | NA | case-control |
| CSF MCP-1 | Chen X, 2018 | High values vs. low values | 18-Mar | NA | case-control |
| CSF IL-17 | Chen X, 2018 | High values vs. low values | 18-Mar | NA | case-control |
| CSF IL-15 | Chen X, 2018 | High values vs. low values | 18-Mar | NA | case-control |
| CSF G-CSF | Chen X, 2018 | High values vs. low values | 18-Mar | NA | case-control |
| CSF IL-2 | Chen X, 2018 | High values vs. low values | 18-Mar | NA | case-control |
| CSF NFH | Xu Z, 2016 | High values vs. low values | 16-May | QUADAS-2 | case-control |
| CSF TDP-43 | Gambino C,2023 | High values vs. low values | 22-Apr | QUADAS-2 | case-control |
| CSF homocysteine | Hu N,2023 | High values vs. low values | 22-Oct | AHRQ | cross-sectional |
| CSF t-tau | Thapa S, 2023 | High values vs. low values | 22-May | ROBINS-I | case-control |
| CSF CHI3L1 | Xu A, 2024 | High values vs. low values | 23-Apr | NOS | case-control |
| Serum 8-OHdG | Wang Z, 2019 | High values vs. low values | 18-Sep | NA | case-control, cross-sectional |
| Serum GSH | Wang Z, 2019 | High values vs. low values | 18-Sep | NA | case-control, cross-sectional |
| Serum AOPP | Wang Z, 2019 | High values vs. low values | 18-Sep | NA | case-control |
| Serum MDA | Wang Z, 2019 | High values vs. low values | 18-Sep | NA | case-control |
| Serum lead | Farace C, 2022 | High values vs. low values | 22-Feb | NA | case-control |
| Serum TNF-α | Hu Y, 2017 | High values vs. low values | 16-May | NA | case-control |
| Serum TNFR1 | Hu Y, 2017 | High values vs. low values | 16-May | NA | case-control |
| Serum IL-1β | Hu Y, 2017 | High values vs. low values | 16-May | NA | case-control |
| Serum IL-6 | Hu Y, 2017 | High values vs. low values | 16-May | NA | case-control |
| Serum IL-8 | Hu Y, 2017 | High values vs. low values | 16-May | NA | case-control |
| Serum IL-17 | Gautam, A. S,2023 | High values vs. low values | 22-Jan | NOS | case-control, cross-sectional |
| Serum VEGF | Hu Y, 2017 | High values vs. low values | 16-May | NA | case-control |
| Serum FBG | Cheng Y, 2021 | High values vs. low values | 20-Jan | NA | case-control, cross-sectional |
| Serum CK | Cheng Y, 2021 | High values vs. low values | 20-Jan | NA | case-control |
| Serum TSC | Cheng Y, 2021 | High values vs. low values | 20-Jan | NA | case-control, cross-sectional |
| Serum TIBC | Cheng Y, 2021 | High values vs. low values | 20-Jan | NA | case-control, cross-sectional |
| Serum creatinine | Liu J, 2020 | High values vs. low values | 19-Feb | NOS | case-control, cross-sectional |
| Serum folic | Hu N,2023 | High values vs. low values | 22-Oct | AHRQ | cross-sectional |
| Serum miR-206 | Liu H,2023 | High values vs. low values | 22-May | NA | case-control |
| Serum miR-338-3p | Liu H,2023 | High values vs. low values | 22-May | NA | case-control |
| Serum miR-133b | Liu H,2023 | High values vs. low values | 22-May | NA | case-control |
| Serum miR-133a | Liu H,2023 | High values vs. low values | 22-May | NA | case-control |
| **NS** |  |  |  |  |  |
| CSF VEGF | Chen X, 2018 | High values vs. low values | 18-Mar | NA | case-control |
| CSF lead | Farace C, 2022 | High values vs. low values | 22-Feb | NA | case-control |
| CSF p-tau | Thapa S, 2023 | High values vs. low values | 22-May | ROBINS-I | case-control |
| Serum NFH | Xu Z, 2016 | High values vs. low values | 16-May | QUADAS-2 | case-control |
| Serum iron | Wang L, 2020 | High values vs. low values | 19-Mar | NOS | case-control, cross-sectional |
| Serum HDL | Cheng Y, 2021 | High values vs. low values | 44946 | NA | case-control, cross-sectional |
| Serum LDL | Liu J, 2020 | High values vs. low values | 20-Jul | NOS | case-control, cross-sectional |
| Serum TC | Liu J, 2020 | High values vs. low values | 20-Jul | NOS | case-control, cross-sectional |
| Serum TG | Liu J, 2020 | High values vs. low values | 20-Jul | NOS | case-control, cross-sectional |
| Serum homocysteine | Hu N,2023 | High values vs. low values | 22-Oct | AHRQ | cross-sectional |
| Serum vitamin B12 | Hu N,2023 | High values vs. low values | 22-Oct | AHRQ | cross-sectional |
| Serum galectin | Ramos E,2022 | High values vs. low values | 22-Feb | NOS | case-control |
| Serum selenium | Zhou,J,2023 | High values vs. low values | 23-Jul | NOS | case-control |
| Serum vitamin D | Lanznaster D, 2020 | High values vs. low values | 19-Sep | ROBINS-I | case-control |
| Serum ApoA1 | Chalitsios C, 2024 | High values vs. low values | 24-Feb | NOS | cohort |
| Serum ApoB | Chalitsios C, 2024 | High values vs. low values | 24-Feb | NOS | cohort |

NA: not available; NFL: Neurofilaments light chain; TNF: tumor necrosis factor; MIP: Macrophage Inflammatory Proteins; MCP: Monocyte Chemoattractant Protein; IL: interleukin; G-CSF: Granulocyte colony-stimulating factor; NFH: Neurofilaments heavy chain; TDP-43:an RNA-binding protein; 8-OHdG: 8-hydroxyguanosine; GSH: glutathione; AOPP: Advanced Oxidation Protein Product; MDAmalondialdehyde; TNFR1, TNF receptor 1; VEGF: Vascular endothelial growth factor; FBG: fasting blood glucose; CK: creatine kinase; TSC: transferrin saturation coefficient; TIBC: total iron binding capacity; miR: microRNA; HDL: High-density lipoprotein; LDL: Low-density lipoprotein; TC: Total cholesterol; TG: Triglyceride; ApoA1: apolipoprotein A1; ApoB: apolipoprotein B; t-tau: total tau; p-tau: phosphorylated-tau; CHIT1: chitotriosidase; CHI3L1: chitinase 3-like 1; NOS: Newcastle-Ottawa Scale; ROBINS-I: Risk Of Bias In Non-randomised Studies - of Interventions; AHRQ: Agency for Healthcare Research and Quality; QUADAS-2: Quality Assessment of Diagnostic Accuracy Studies-2;
