# Supplementary material for: Environmental risk factors, protective factors, and biomarkers for amyotrophic lateral sclerosis: an umbrella review
Source: Front Aging Neurosci. 2025 Jun 13;17:1541779. doi: 10.3389/fnagi.2025.1541779 (PMC12202415; doi:10.3389/fnagi.2025.1541779)
Supplement: Supplementary file 7 [file Table_5.docx]

| **ITEMS** | **1** | **2** | **3** | **4** | **5** | **6** | **7** | **8** | **9** | **10** | **11** | **12** | **13** | **14** | **15** | **16** | **Final rating** |
| --- | --- | --- | --- | --- | --- | --- | --- | --- | --- | --- | --- | --- | --- | --- | --- | --- | --- |
| Blecher 2019 | Y | N | Y | N | N | Y | Y | Y | N | Y | Y | Y | N | Y | N | Y | Critically low |
| Chang 2020 | Y | N | Y | Y | N | Y | Y | N | Y | N | Y | N | Y | N | Y | Y | Low |
| Chalitsios 2024 | Y | Y | Y | N | Y | Y | Y | N | Y | N | Y | N | Y | Y | Y | Y | Low |
| Duan 2023 | Y | Y | Y | Y | Y | Y | Y | N | Y | N | Y | Y | N | Y | Y | Y | Low |
| Fitzerald 2014 | Y | N | N | N | N | N | N | N | N | N | Y | Y | Y | Y | N | Y | Critically low |
| Fitzerald 2013 | Y | N | N | N | N | N | N | N | N | N | Y | N | Y | N | N | Y | Critically low |
| Gong 2023 | Y | Y | Y | Y | Y | Y | Y | Y | Y | Y | Y | Y | Y | Y | Y | Y | High |
| Gu 2021 | Y | N | Y | N | Y | Y | Y | Y | N | N | Y | Y | Y | Y | Y | Y | Critically low |
| Gunnarsson 2018 | Y | N | Y | N | N | N | N | N | Y | Y | Y | N | Y | Y | Y | Y | Critically low |
| Hu 2022 | Y | N | N | Y | Y | Y | Y | Y | Y | N | Y | N | Y | N | Y | Y | Low |
| Jalilian 2021 | Y | N | N | Y | Y | N | Y | Y | Y | N | Y | N | Y | Y | Y | Y | Low |
| Kang 2014 | Y | N | Y | Y | Y | N | Y | Y | N | N | Y | N | Y | Y | Y | Y | Critically low |
| Kim 2024 | Y | Y | Y | Y | Y | Y | Y | N | Y | N | Y | Y | Y | N | Y | Y | Moderated |
| Meng 2020 | Y | N | Y | N | Y | Y | Y | Y | Y | Y | Y | Y | Y | Y | Y | Y | Critically low |
| Tai 2017 | Y | N | Y | N | N | Y | Y | Y | Y | N | Y | Y | Y | Y | Y | Y | Critically low |
| Thapa 2023 | Y | N | Y | Y | Y | Y | Y | N | Y | N | Y | Y | Y | Y | Y | Y | Low |
| Wang 2014 | Y | N | Y | Y | N | N | Y | N | Y | N | Y | Y | Y | Y | Y | Y | Low |
| Wannarrong 2020 | Y | N | Y | Y | Y | Y | Y | Y | Y | N | Y | Y | N | Y | Y | Y | Critically low |
| Watanabe 2017 | Y | N | Y | Y | N | N | Y | Y | N | N | Y | Y | Y | Y | Y | Y | Critically low |
| Zeng 2019 | Y | N | N | N | N | N | N | Y | N | N | N | N | N | N | Y | Y | Critically low |
| Zhang 2023 | Y | N | Y | Y | N | Y | Y | Y | Y | N | Y | Y | N | Y | Y | Y | Critically low |
| Zheng 2023 | Y | Y | Y | Y | Y | Y | Y | Y | Y | Y | Y | Y | N | Y | Y | Y | Low |
| Zhu 2023 | Y | Y | Y | Y | Y | Y | Y | N | Y | N | Y | Y | Y | Y | Y | Y | High |
| Chen 2018 | Y | N | N | Y | Y | N | Y | N | N | N | Y | Y | N | Y | Y | Y | Critically low |
| Cheng 2021 | Y | N | Y | Y | N | N | Y | N | N | N | Y | Y | N | Y | Y | N | Critically low |
| Farace 2022 | Y | N | Y | N | Y | N | Y | N | N | N | N | Y | Y | Y | Y | Y | Critically low |
| Gambino 2023 | Y | N | Y | Y | Y | Y | Y | N | Y | Y | Y | N | N | N | Y | Y | Critically low |
| Gautam 2023 | Y | Y | Y | Y | Y | Y | Y | Y | Y | N | Y | N | N | N | Y | Y | Low |
| Hu 2017 | Y | N | N | Y | Y | Y | Y | N | N | N | Y | Y | N | Y | Y | Y | Critically low |
| Hu 2023 | Y | N | N | Y | Y | Y | Y | Y | Y | N | Y | N | N | Y | Y | Y | Critically low |
| Liu 2020 (lipid) | Y | Y | N | N | Y | Y | Y | N | Y | N | Y | Y | Y | Y | Y | Y | Low |
| Liu 2020 | Y | Y | N | Y | Y | Y | Y | N | Y | N | Y | Y | Y | Y | Y | Y | Moderated |
| Lanznaster 2020 | Y | Y | Y | Y | Y | Y | Y | N | Y | N | Y | N | N | N | Y | Y | Low |
| Liu 2023 | Y | N | N | N | Y | Y | Y | N | N | N | Y | Y | N | Y | Y | Y | Critically low |
| Ramos-Martínez 2022 | Y | Y | N | Y | Y | Y | Y | N | Y | Y | Y | Y | Y | Y | Y | Y | Moderated |
| Sferruzza 2022 | Y | N | N | Y | Y | N | Y | N | Y | N | Y | Y | N | Y | Y | Y | Critically low |
| Wang 2020 | Y | N | Y | Y | Y | Y | Y | Y | Y | N | Y | Y | Y | Y | Y | Y | Low |
| Wang 2019 | Y | N | N | Y | Y | N | N | N | N | N | Y | Y | Y | Y | Y | Y | Critically low |
| Xu 2016 | Y | N | N | Y | N | N | Y | N | Y | N | Y | N | N | N | Y | Y | Critically low |
| Xu 2024 | Y | Y | Y | Y | Y | Y | Y | N | Y | N | Y | Y | N | Y | Y | Y | Low |
| Zhang 2018 | Y | N | Y | Y | Y | Y | Y | N | Y | N | Y | Y | Y | Y | Y | Y | Low |
| Zhou 2023 | Y | Y | Y | Y | Y | N | Y | N | Y | N | Y | N | N | Y | Y | Y | Low |
| Zhu 2018 | Y | Y | Y | Y | Y | Y | Y | N | Y | N | Y | Y | Y | Y | Y | Y | Moderated |

**Table S5. Quality assessment of included meta-analyses focusing on environmental factors and biomarkers with AMSTAR 2.**
